# Revision of the western Palaearctic species of *Aleiodes* Wesmael (Hymenoptera, Braconidae, Rogadinae). Part 1: Introduction, key to species groups, outlying distinctive species, and revisionary notes on some further species

**DOI:** 10.3897/zookeys.639.10893

**Published:** 2016-12-12

**Authors:** Cornelis van Achterberg, Mark R. Shaw

**Affiliations:** 1 Research Associate, Department of Terrestrial Zoology, Naturalis Biodiversity Center, Postbus 9517, 2300 RA Leiden, The Netherlands; 2 Honorary Research Associate, National Museums of Scotland, Chambers Street, Edinburgh EH1 1JF, Scotland, U.K.

**Keywords:** Aleiodes, host range, biology, distribution, Europe, phenology

## Abstract

Seven new species of the genus *Aleiodes* Wesmael, 1838 (Braconidae: Rogadinae) are described and illustrated: *Aleiodes abraxanae* **sp. n.**, *Aleiodes angustipterus* **sp. n.**, *Aleiodes artesiariae* **sp. n.**, *Aleiodes carminatus* **sp. n.**, *Aleiodes diarsianae* **sp. n.**, *Aleiodes leptofemur* **sp. n.**, and *Aleiodes ryrholmi* **sp. n.** A neotype is designated for each of *Aleiodes circumscriptus* (Nees, 1834) and *Aleiodes pictus* (Herrich-Schäffer, 1838), and both species are redescribed and illustrated. *Aleiodes ochraceus* Hellén, 1927 (not *Aleiodes ochraceus* (Curtis, 1834)) is renamed as *Aleiodes curticornis* **nom. n.** & **stat. rev.**, and redescribed and illustrated. *Aleiodes bistrigatus* Roman, 1917, *Aleiodes nigriceps* Wesmael, 1838, and *Aleiodes reticulatus* (Noskiewicz, 1956), are re-instated as valid species. A lectotype is designated for *Aleiodes bistrigatus* Roman. An illustrated key is given to some distinctive species and the residual species groups along which further parts of an entire revision of western Palaearctic species of *Aleiodes* and *Heterogamus* will be organised. Biology, host associations and phenology are discussed for the keyed species (in addition to the above, *Aleiodes albitibia* (Herrich-Schäffer, 1838), *Aleiodes apiculatus* (Fahringer, 1932), *Aleiodes arcticus* (Thomson, 1892), *Aleiodes cantherius* (Lyle, 1919), *Aleiodes esenbeckii* (Hartig, 1834), *Aleiodes jakowlewi* (Kokujev, 1898), *Aleiodes modestus* (Reinhard, 1863), *Aleiodes nigricornis* Wesmael, 1838, *Aleiodes pallidator* (Thunberg, 1822), *Aleiodes praetor* (Reinhard, 1863), *Aleiodes seriatus* (Herrich- Schäffer, 1838) *sensu lato*, *Aleiodes testaceus* (Telenga, 1941), *Aleiodes ungularis* (Thomson, 1892), and *Aleiodes varius* (Herrich-Schäffer, 1838)) which are dealt with in full here (with the exception of *Aleiodes seriatus* *s.l.* which is, however, included in the key). The experimental methodology covering the revision as a whole, which involves some behavioural investigation, is outlined.

## Introduction

As defined by van Achterberg (1991), the large genus *Aleiodes* Wesmael, 1838, has a world-wide distribution and in the western Palaearctic region it forms a moderately prominent element of the braconid fauna. Despite the relatively large size and/or abundance of several species, and the frequency with which they are reared by lepidopterists, there are no reliable identification keys to the western Palaearctic species.

Van Achterberg (1991), for the W. Palaearctic and Afrotropical region, recognised and keyed three subgenera of *Aleiodes* (*Aleiodes* (including *Heterogamus* Wesmael, 1838), *Chelonorhogas* Enderlein, 1912 and *Neorhogas* Szépligeti, 1906). Subsequently, from a morphological phylogenetic analysis of the genus *Aleiodes* as defined by van Achterberg (1991), Fortier and S.R. Shaw (1999) proposed 18 monophyletic species groups, one of which was further divided by Fortier (2006). Zaldívar-Riverón et al. (2008), in a molecular phylogenetic study of the subfamily Rogadinae, defined a tribe Aleiodini to include only two genera, the resurrected *Heterogamus* and *Aleiodes*, within which were placed *Cordylorhogas* Enderlein, 1920, *Hemigyroneuron* Baker, 1917 and *Pholichora* van Achterberg, 1991. Within this differently defined *Aleiodes* two main clades were recovered, one corresponding to *Chelonorhogas* in van Achterberg’s (1991) sense, and no evidence was found to support the monophyly of most of the species groups proposed by Fortier and S.R. Shaw (1999); although a few proposals (e.g. the *pilosus*-group (*Tetrasphaeropyx* Ashmead, 1888) later recognised by Fortier (2006)) may indeed concern monophyletic assemblages embedded within *Aleiodes*. The conclusion that most of the species groups proposed by Fortier and S.R. Shaw (1999) contain unrelated elements has been further corroborated by more recent molecular work (Butcher et al. 2012, Quicke and M.R. Shaw unpublished), and many characters used by Fortier and S.R. Shaw (1999) for defining species groups, such as pectination of the claws, are homoplastic. In the present work, which is restricted to the W. Palaearctic region, we not only reject the classification proposed by Fortier and S.R. Shaw (1999) but also, indeed, choose to leave the definition of formal species groups aside. For the purposes of the key, we are using informal species groups that can be defined by the characters given in the key, but our concept of the groups differ from those used by Fortier and S.R. Shaw (1999); in particular our *circumscriptus*- and *apicalis*-groups are used in a much wider context but, along with our *bicolor*-group, they do appear each to be monophyletic, at least within the W. Palaearctic (Quicke and M.R. Shaw unpublished). It should also be understood that our use of *Aleiodes seriatus*-agg. applies to a small group of cryptic species, not corresponding to the wider concept of a certainly disparate *seriatus*-group proposed by Fortier and S.R. Shaw (1999). Despite the findings of Zaldívar-Riverón et al. (2008), we do not use the subgeneric name *Chelonorhogas* for our *apicalis*-group partly because the characters by which we recognise the W. Palaearctic elements break down when applied to the World fauna, but also because there are some indications (Butcher et al. 2012) that it may be a basal grade group, paraphyletic with respect to the subgenus Aleiodes as recognised by Zaldívar-Riverón et al. (2008). Further analysis of *Aleiodes* in the context of the wider Rogadinae on a world basis is in hand (Quicke et al. in prep.).

Over the past 45 years, during a programme aimed at investigating host relations of Ichneumonoidea in which Rogadinae and some other cyclostome braconid groups have been strong foci, the second author has reared many western European species of *Aleiodes* from their Lepidoptera hosts, and also received donations of specimens reared by a large number of lepidopterists. Investigation of *Aleiodes* host ranges has also involved some experimentation using short-term cultures by the second author, and in some cases this has been motivated by, and crucial for, elucidating species-level taxonomy. The large amount of collected data will be used for a revision of the western Palaearctic species of the genera *Aleiodes* and *Heterogamus*, also covering the host range, phenology and other aspects of biology of as many species as our data permit.

In this first paper we give a key to the species groups that will be dealt with in further parts, and to some of the more distinctive species which are then dealt with in full here. Some species that urgently need valid names are newly described and/or redescribed to clarify the confused nomenclature and status of some nominal species. One valid species (*Aleiodes ochraceus* Hellén, 1927) is renamed as *Aleiodes curticornis* (nom. n. & stat. rev.), because Hellén’s name is a junior homonym; and moreover not a synonym (as proposed by Papp 1985) of *Aleiodes gastritor* (Thunberg, 1822). Some parts of the genus appear to have radiated relatively recently and even in the well-studied British fauna it is probable that further biological research will reveal that some of the taxa currently recognised as single species are in fact aggregates of biologically distinct entities resistant to morphological separation. An example is given in this paper with the *Aleiodes pictus*-aggregate, and a relevant speciation hypothesis has been suggested by M.R. Shaw (1994, 2002), M.R. Shaw and Horstmann (1997) and Stigenberg and M.R. Shaw (2013). We outline the experimental methods involved in the short-term culture experiments involved in elucidating that complex (which will apply also to other parts of the revision), and the ways in which both natural rearings and experimental results are and will be presented. Overview information on the general developmental biology of *Aleiodes* will be given elsewhere (M.R. Shaw, in prep.), though particular points of interest will be mentioned under the species concerned and, to facilitate that, a brief description of the “normal” oviposition sequence is given in this paper.

## Specimens, methods and presentation of records

Unless stated otherwise the following protocols apply here and to ensuing parts of this work. Distributions are based only on material studied by us, unless otherwise stated. Literature records (e.g. as in Shenefelt 1975 and Yu et al. 2012) indicate much wider distributions for many of the species but, while this may often be true, we have in most cases no means of verifying the records. Indeed, we have noted a high level of previous misidentification in the material we have been able to examine, as is usual during taxonomic revision of poorly known groups. Country records that are not (at the time of writing) given in Fauna Europaea are indicated with an asterisk (*). In cases in which we have seen large numbers of specimens the distributional data are given only in summary form. For Britain this is usually just as Watsonian Vice County numbers (Dandy 1969) in order within countries but, when very numerous paratypes are involved (e.g. as in *Aleiodes leptofemur* sp. n.), the British Isles data are given as the names of Watsonian Vice Counties. For The Netherlands and other countries the province and locality are mentioned when possible. Host records are similarly based only on material that we have examined personally, although the host-ranges given here are likely to be a suitable basis from which to evaluate the probability that other host records (e.g. as in Shenefelt 1975 and Yu et al. 2012) are accurate. Host determinations taken from specimen labels are subject to the usual possibilities of host misidentification (including the possibility that overlooked “mummies” (= the structure within which the *Aleiodes* pupates, formed of the hardened host larval skin) of radically different hosts were undetected in mass-rearing programmes of particular host species), although host mummies have often been retained with specimens permitting a re-evaluation of host determination in many cases, quite often leading to a different conclusion. Rather than transcribing often obsolete or misspelt names from data labels, we have updated nomenclature when appropriate. Some of the records that we believe to be particularly anomalous or to require extra interpretation are discussed further. However, the majority of the reared specimens have resulted from special rearing efforts under carefully controlled conditions (M.R. Shaw 1997) undertaken by one of us (MRS), and much material supporting these records is deposited in the National Museums of Scotland, Edinburgh (NMS); in additional cases the relevant depository is given. The host records are given quantitatively for reasons outlined by M.R. Shaw (1994): the number immediately following each host name indicates the number of examined specimens reared from that host, and the number separated by a colon that sometimes follows plural records indicates the number of localities from which the records result, if it is fewer than three. The majority of our host records are of British rearings, and the names of hosts that are included only as a result of non-British rearings are followed by the name of their country of origin. All rearing records given in quantitative form are from wild-collected hosts. Experimental investigation of host range has been undertaken for several British species, and the results are given separately for each *Aleoides*/host pair in the summary form N: x \ y \\ p \ q + r, where N = the number of female parasitoids involved, x = the number of that host species offered, y = the number of hosts accepted, p = the number of accepted hosts that survived (mortality includes cannibalised hosts and dead hosts that couldn’t be dissected), q = hosts that produced mummies of the *Aleiodes* (in a few cases this includes hosts that were dissected to reveal healthy parasitoid larvae with gut content), r = produced healthy Lepidoptera pupae. A concise summary of host range, to suggest searching environment as well as host taxa, is given for all species for which information is sufficient.

Except for the limited time it took to perform experiments and/or service rearing containers indoors, all livestock (including wild collected caterpillars harbouring parasitoids; Lepidoptera cultures; *Aleiodes* mummies awaiting emergence; adult *Aleiodes*, from whatever source; experimentally parasitized and control caterpillars) was kept in an unheated, well-ventilated and fully shaded detached outdoor shed that held outdoor shade temperatures (Reading, UK and Edinburgh, UK) generally to within 0.5 °C of ambient (M.R. Shaw 1997), except that adult *Aleiodes* to be used for experiments were generally removed to cooler conditions when the shed temperature reached 16–20 °C. They were kept individually in corked 2.5 × 7.5 cm sterilised (household bleach) glass tubes (hereafter “tube(s)”) and dilute (initially 1:3) honey:water droplets were touched onto the insides of the tubes, and replenished or diluted as required to maintain *ad libitum* access to honey dilute enough to be imbibed. As far as possible, honey was sourced from areas remote from arable agriculture, and, in cases when the adult parasitoid had overwintered, a paste of pollen (obtained from health food shops) in dilute honey was additionally offered. Fresh tubes were used as soon as any sign of mould arose, and under these conditions females of most species could routinely be kept alive for at least 2 months; often considerably longer (regularly 4–6 months) and usually for well over a year in the case of species that overwinter as diapausing adults. Lepidoptera cultures used in experiments were obtained as eggs from captive females. Trials involving known or putative hosts were conducted by introducing a single female parasitoid to a single active host caterpillar of appropriate size (generally 2^nd^ instar) in a clean tube without vegetation; putative hosts were judged rejected after 10 minutes (prolonged to 5 contacts if these had not occurred in the time). Occasionally more than one species, or a range of sizes, were offered simultaneously, but in these cases rejections were scored only if all hosts simultaneously offered were rejected. Sometimes runs of parasitized hosts could be extended by offering hosts in proecdysis, as these are often accepted, but rejections were not scored using hosts that were subactive in this way. Similarly, because *Aleiodes* species are synovigenic, for scored trials no female was offered hosts after four ovipositions on that day (although most species are probably capable of up to at least 8 ovipositions on a reasonably warm day), but runs of fully acceptable hosts were sometimes extended beyond that. In addition to egg depletion, it was often clear that the supply of venom used to induce pre-oviposition paralysis was subject to temporary exhaustion, sometimes more so than eggs. Parasitized and (separately) control hosts were reared on carefully searched, clean, wild food plants as counted cohorts in 14 × 8 × 6 or 18 × 12 × 6 cm plastic boxes bottom-lined with 4–6 sheets of absorbent tissue (white lavatory roll); food and tissue was changed at least weekly, when each caterpillar was rigorously accounted for. All mummies were removed when found, allowed to dry in open air, and then placed in tubes to await adult emergence (inspected at least daily except in winter).

Mating trials were conducted by introducing a female to a fed male already present in a clean tube (not the other way round); generally, fresh tubes were used for each pair, and trials were done with newly emerged females (if possible before she had fed). The results of mating trials are given only impressionistically, as no way was found to quantify them satisfactorily.

The collections used for our revision contain the majority of recently collected material of *Aleiodes* from the western Palaearctic region; collections with type material are separately listed under the description of the species. The following collections and acronyms are used: AAC (A.A. Allen Collection, Dawlish), ALC (A. Lozan Collection, Institute of Entomology, České Budĕjovice), BMNH (Natural History Museum, London), BZL (Oberösterreichisches Landesmuseum, Biologiezentrum, Linz), CC (M. Čapek Collection, Moravian Museum, Brno), CMIM (C. Morley Collection, Ipswich Museum, Ipswich), CNC (Canadian National Collection of Insects, Ottawa), FC (J.V. Falcó Collection, Valencia), FMNH (Finnish Museum of Natural History, Helsinki), FRAH (Forest Research, Alice Holt Lodge, Farnham), KBIN (Koninklijk Belgisch Instituut voor Natuurwetenschappen, Brussels), JLC (J. Lukáš Collection, Bratislava), MCZ (Museum of Comparative Zoology, Harvard University, Cambridge, U.S.A.), MHHN (Muséum National d’Histoire Naturelle, Paris), MMUM (Manchester Museum, University of Manchester, Manchester), MSC (M. Schwarz Collection, Linz-Ansfelden), MTMA (Hungarian Natural History Museum, Budapest), NMI (National Museum of Ireland, Dublin), NMS (National Museums of Scotland, Edinburgh), NNHM (National Natural History Museum, Oslo), NRS (Swedish Natural History Museum, Stockholm), OUM (Oxford University Museum of Natural History, Oxford), PAN (Museum and Institute of Zoology, Polish Academy of Sciences, Warsaw & Łomna-Las), RMNH (Naturalis Biodiversity Center, Leiden), SDEI (Senkenberg Deutches Entomologisches Institut, Müncheberg), SMNS (Staatliches Museum für Naturkunde, Stuttgart), SYKE (Finnish Environment Institute, Friendship Park Research Centre, Kuhmo), UMZC (University Museum of Zoology, Cambridge), USNM (U.S. National Museum of Natural History, Washington D.C.), UWIM (University of Wyoming Insect Museum, Laramie), WAE (W.A. Ely Collection, Rotherham), ZILU (Zoological Institute, Lund University, Lund), ZISP (Zoological Institute, Academia NAUK, St. Petersburg), ZJUH (Zhejiang University, Hangzhou), ZMB (Zoologisches Museum, Humboldt Universität, Berlin), ZMC (Zoological Museum, Copenhagen), ZMUO (Zoological Museum, University of Oulu, Oulu), ZMUU (Zoological Museum of Uppsala University, Uppsala), and ZSSM (Zoologische Staatssammlung, München). In addition we have seen specimens from various smaller and private collections, which are cited in significant cases.

The number of antennal (i.e. flagellar + 2) segments is frequently an important aid to species recognition and of interest also because in some species the female has more segments on average than the male (while other species are more normal in that the male has the greater number). We give counts of antennal segments for the specimens we have examined, but for some species (especially when the segments did not need to be counted for determination) sometimes only for the first hundred or so of the specimens examined of each sex.

GenBank accession numbers are given for DNA sequences from specimens in the NMS collection bearing a “MRS Aleiodes DNA [number]”, in this text simplified to MRS[number] followed in parentheses by the country of origin then GenBank accession number and gene fragment. Not all sequenced samples have been accessioned, but for each species for which we have sequences we give several if available. Further, the DNA data of many taxa and putative taxa have been produced over a long number of years under several auspices: many sequences have been used in other studies of various kinds, often not taxonomic, and often under provisional or unpublished names. Thus many of those previously submitted to GenBank and BOLD had inappropriate names applied, but the names associated with MRS vouchers and accession numbers in this paper supplant previous identifications.

**Figures 1–6.**
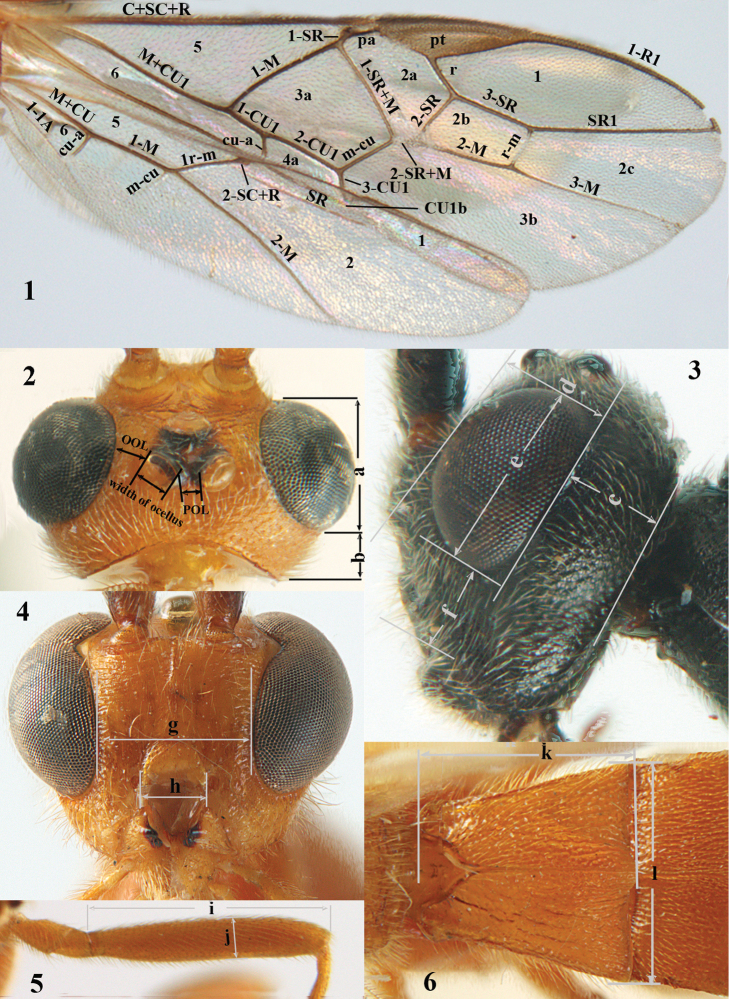
Terminology and measurements used in this paper. **1** wing venation: pa = parastigma, pt = pterostigma, 1 = marginal cell, 2a, b, c = first, second and third submarginal cell, respectively, 3a, b = first and second discal cell, respectively, 4a = first subdiscal cell, 5 = basal cell, 6 = subbasal cell **2** head dorsal: a = length of eye, b = length of temple **3** head lateral: c = width of temple, d = width of eye, e = height of eye, f = width of malar space (measured as actual true distance in its own plane) **4** head anterior: g = width of face, h = width of hypoclypeal depression **5** fore femur lateral: i = length, j = width **6** first metasomal tergite dorsal: k = length of tergite (measured from adductor), l = apical width of tergite.

For the recognition of braconid subfamilies, see van Achterberg (1990, 1993, 1997), for the identification of *Aleiodes* Wesmael, see van Achterberg (1991) and Chen and He (1997), and for the terminology used in this paper see Figs 1–6 and in van Achterberg (1988, 1993; note, however, that in the present work the distance between eye and lateral ocellus is measured differently). For additional references see Yu et al. (2012).

## Taxonomy

### Aleiodes

Taxon classificationAnimaliaHymenopteraBraconidae

Wesmael, 1838

Figs 1 , 7–8 , 9–19 , 20–21 , 22–34 , 35 , 36–47 , 48–49 , 50–62 , 63–64 , 65–75 , 76–77 , 78–88 , 89–98 , 99–100 , 101–112 , 113 , 114–124 , 125–126 , 127–137 , 138 , 139–151 , 152–153 , 154–164 , 165–166 , 167–177 , 178–179 , 180–189 , 190 , 191–202 , 203–204 , 205–214 , 215–216 , 217–229 , 230–231 , 232–242 , 243–244 , 245–256 , 257 , 258–259 , 260–271 , 272–273 , 274–284 , 285 , 286–287 , 288–301 , 302–304 , 305–315 , 316 , 317–327 , 328 , 329–340 , 341–342 , 343–352 , 353–354 , 355–365 , 366–367 , 368–378

Aleiodes Wesmael, 1838: 194; Shenefelt 1975: 1163–1185; Marsh 1979: 177–178; Papp 1985a: 143–164 & 1985b: 347–349; Shaw and Huddleston 1991: 95–96 (biology); van Achterberg 1991: 24. Type species (designated by Viereck 1914): Aleiodes heterogaster Wesmael, 1838 [examined; = Aleiodes albitibia (Herrich-Schäffer, 1838)].
Petalodes Wesmael, 1838: 123; Tobias 1971: 218 (transl. 1975: 86–87); Shenefelt 1975: 1209–1211; Tobias 1976: 90; Marsh 1979: 179; van Achterberg 1991: 24. Type species (by monotypy): Petalodes unicolor Wesmael, 1838 [examined; = Aleiodes compressor (Herrich-Schäffer, 1838)].
Schizoides Wesmael, 1838: 94. Unavailable name.
Nebartha Walker, 1860: 310; Shenefelt 1975: 1216; Marsh 1979: 179; van Achterberg 1991: 24. Type species (by monotypy): Nebartha macropodides Walker, 1860 [examined].
Tetrasphaeropyx Ashmead, 1889: 634; Shenefelt 1975: 1260; Marsh 1979: 181; Fortier 2009: 19 (as subgenus; revision). Type species (by monotypy): Rogas pilosus Cresson, 1872 [examined].
Neorhogas Szépligeti, 1906: 605; Shenefelt 1975: 1205; van Achterberg 1991: 24. Type species (by monotypy): Neorhogas luteus Szépligeti, 1906 [examined; = Aleiodes praetor (Reinhard, 1863)].
Chelonorhogas Enderlein, 1912b: 258; Shenefelt 1975: 1187; van Achterberg 1991: 24. Type species (by monotypy): Chelonorhogas rufithorax Enderlein, 1912 [examined; not Aleiodes rufithorax (Cameron, 1911) = Aleiodes convexus van Achterberg, 1991].
Leluthinus Enderlein, 1912c: 96; Shenefelt 1975: 1202–1203; van Achterberg 1991: 24. Type species (by monotypy): Leluthinus lividus Enderlein, 1912 [examined].
Aleirhogas Baker, 1917b: 383, 411; Shenefelt 1974: 1185–1186; van Achterberg 1991: 24. Type species (designated by Viereck 1922): Rhogas (Aleirhogas) schultzei Baker, 1917 [examined].
Heterogamoides Fullaway, 1919: 43; Shenefelt 1975: 1188; van Achterberg 1991: 24. Type species (by monotypy): Heterogamoides muirii Fullaway, 1919 [examined].
Cordylorhogas Enderlein, 1920: 153; Shenefelt 1975: 1195; van Achterberg 1991: 31. Type species (by monotypy): Cordylorhogas trifasciatus Enderlein, 1920 [examined].
Hyperstemma Shestakov, 1940: 10; Shenefelt 1975: 1200; van Achterberg 1991: 24. Type species (by monotypy): Hyperstemma chlorotica Shestakov, 1940 [examined].
Rhogas auctt.; Tobias 1971: 215–217 (transl. 1975: 83–86); Shenefelt 1975: 1215–1256; Tobias 1976: 81–89; Marsh 1979: 179–181; Tobias 1986: 74–84.

#### Diagnosis.

Propodeum with a long median carina dorsally (Figs 11, 52); ovipositor sheath slightly expanded towards apex or parallel-sided and comparatively wide as far as visible (Figs 12, 31, 152, 301); second metasomal tergite with a median carina anteriorly (Figs 193, 291, 320, 331), but absent in part of the genus; hind trochantellus of ♀ normal, at most 2.6 × as long as wide (Figs 55, 104, 183, 194); vein r of fore wing 0.2–0.8 × vein 3-SR (Figs 1, 9, 22, 65, 205), if 0.6–0.8 × (Fig. 343) then precoxal area of mesopleuron granulate or coriaceous, without rugae and second metasomal tergite without triangular area medio-basally.

#### Biology.

Very large genus of koinobiont, synovigenic endoparasitoids; in the western Palaearctic of Drepanidae (including Thyratirinae), Erebidae (including Hypeninae, Lymantriinae, Arctiinae, Hypenodinae), Geometridae, Hesperiidae, Lasiocampidae, Lycaenidae, Noctuidae, Nolidae, Notodontidae, Nymphalidae (Satyrinae), Pterophoridae, Sphingidae, Ypsolophidae and Zygaenidae. This list includes only taxa of which we have been able to verify hosts, either by our own rearings or by examination of host remains; there are other host groups recorded in the literature, but we regard many of them as almost certainly erroneous and seek confirmation of others. The caterpillars are killed by the endoparasitoid and “mummified” — i.e. turned into a partly shrunken and hardened structure that is more or less tanned (Figs 8, 244, 259, 287, 342, 354), in most cases before their final instar, and the parasitoid pupates and eventually emerges as an adult from this mummy. Almost all *Aleiodes* species are strictly solitary (in Europe only two species are gregarious, but neither is treated in this part).

#### Oviposition.

The oviposition behaviour of *Aleiodes* species is based on the following sequence, from which one or more steps may habitually be eliminated by particular species: (a) antennation of the host, often also investigation using fore and sometimes mid tarsi, during which the host often curls and may be drawn in towards the ventral/mesosomal region of the parasitoid; (b) a rapid sting (usually less than 0.5 second), executed more or less between the parasitoid’s front legs and usually accompanied by a brief fluttering of the wings; (c) waiting motionless by, but often not in physical contact with, the host while temporary paralysis caused by the injected venom takes effect (about 20 to exceptionally 90 seconds); oviposition (a single insertion of the ovipositor, usually about 30–80 seconds duration but regularly much shorter or much longer in certain species); (d) a period (usually about 20–100 seconds) of post-oviposition association, when the parasitoid stands over the host and the host is intermittently antennated, during which time the host recovers from paralysis; (e) abrupt and energetic departure, often by flight. Sluggish hosts are generally unattractive, but superparasitism is frequent if (e) is prevented or if the two come into contact again. In most species host feeding was seen only infrequently or not at all in well-fed parasitoids, but it became commoner in aged females; it was always non-destructive and concurrent (i.e using the same host individual as for oviposition) but took place from separate *ad hoc* wounds made using the ovipositor, usually before but occasionally after oviposition itself. In most species, first instar hosts are oviposited into only with difficulty and even then they frequently die from the trauma, second and early third instars are the most suitable, and from late in the third instar onwards hosts are consistently ignored (a rough guide is that if the host exceeds the length of the parasitoid it will usually be of no interest). In the majority of investigated species the egg floats freely in the haemocoel.

#### Distribution.

Cosmopolitan.

#### Notes.

Two papers with descriptions of the same *Aleiodes* species appeared in 1838. Most likely Herrich-Schäffer’s paper was published earlier (the introduction is dated April, 1838) than Wesmael’s paper. Baron de Stassart stated in his presidential report (Bulletins de l’Académie royale des sciences, des lettres et des beaux-arts de Belgique 5: 328) dated May 6^th^, 1838, that the 11^th^ volume of the Nouveau Memoires was in press.

### Key to West Palaearctic species groups, and outlying distinctive species, of the genus *Aleiodes* Wesmael

**Table:** 

1	Hind trochantellus of female moderately elongate, its ventral length 2.4–4.5 × its width (a), hind wing narrow (b), its vein 1r-m strongly reclivous (c) **and** vein r of fore wing 0.8–3.0 × vein 3-SR (d); second submarginal cell of fore wing about as long as high or distinctly shorter (e)	**genus *Heterogamus* Wesmael, 1838**
	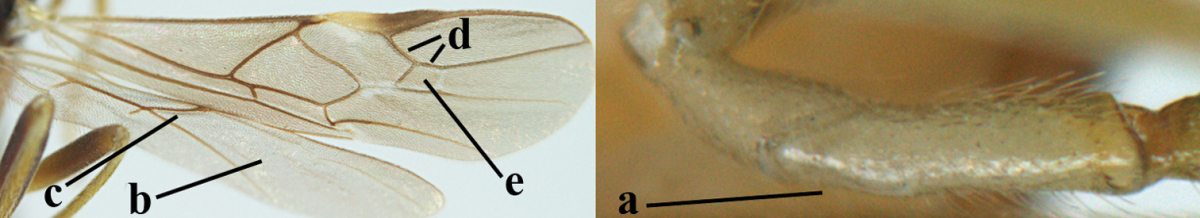	
–	Hind trochantellus of female usually moderately robust (aa), **if** ventrally 2.4–2.8 × as long as wide, then hind wing wider (bb) and its vein 1r-m moderately reclivous (cc) **or** vein r of fore wing shorter than 0.6 × vein 3-SR (dd); second submarginal cell of fore wing often longer than high (ee); genus *Aleiodes* Wesmael, 1838	**2**
	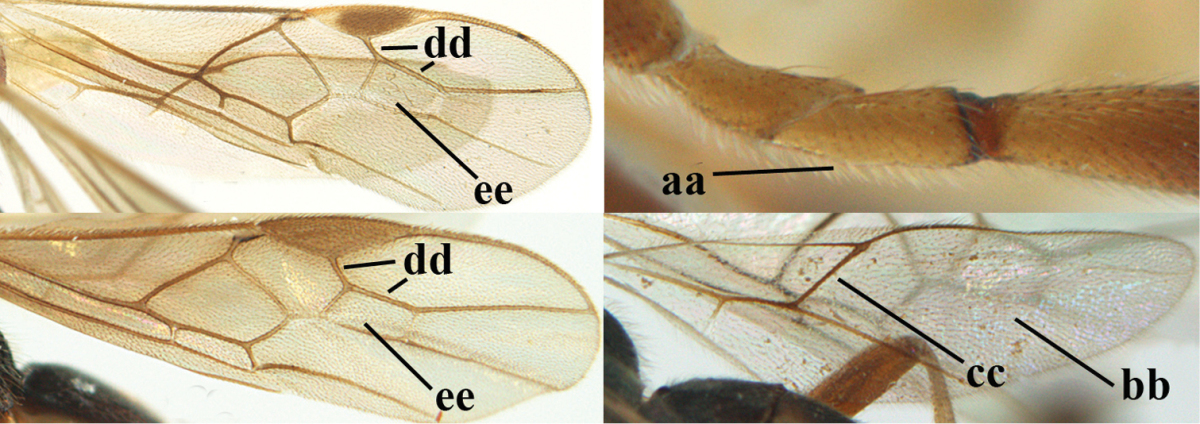	
2	Ovipositor sheath largely glabrous (except apically and ventrally) (a); marginal cell of hind wing narrowed near basal 0.6 and slightly widened apically (b); lateral carina of scutellum strong (c) and lunula rather narrow, but widened medially (d); [ovipositor with small teeth ventrally and with wide dorsal flange (e)]; parasitoid of Sphingidae	***Aleiodes praetor* (Reinhard, 1863)**
	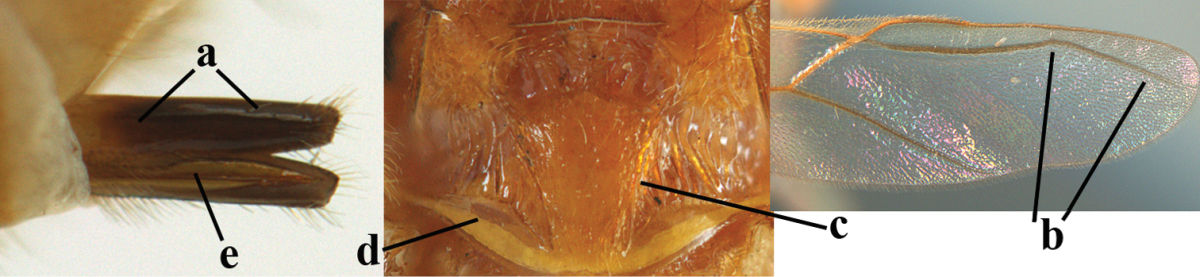	
–	Ovipositor sheath distinctly setose (aa), but sometimes mainly ventrally so; marginal cell of hind wing subparallel-sided (upper bb), evenly widened (lower bb) apically or somewhat narrowed and distinctly widened apically (bbb); lateral carina of scutellum absent (cc) or **if** present (ccc) then lunula wide (dd) or narrow (ddd); parasitoids of other families	**3**
	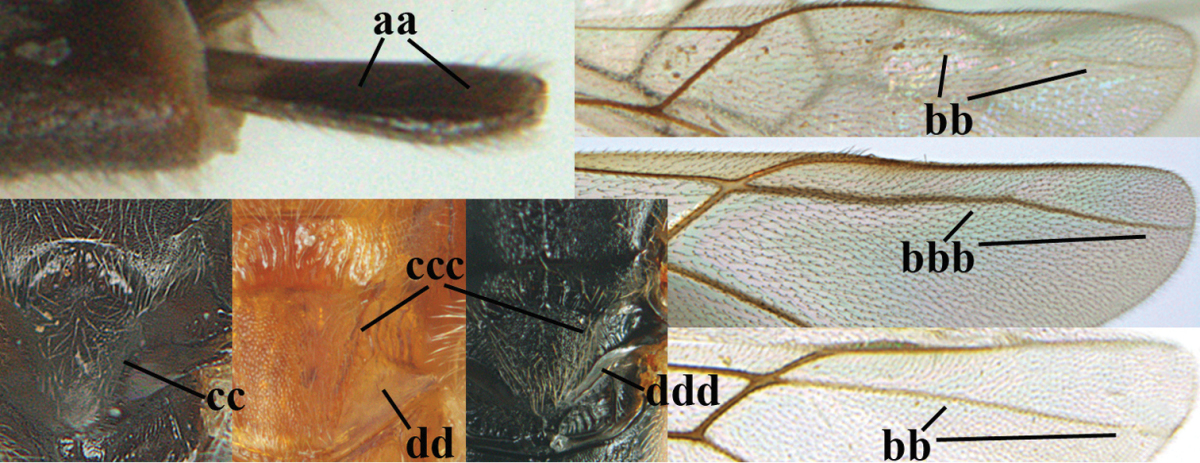	
3	Apical half of marginal cell of hind wing distinctly gradually widened, its maximum width 1.6 × its width near hamuli or wider (a), **if** largely parallel-sided (aaa) then tarsal claws with coarse blackish pecten (b); second metasomal tergite with wide smooth triangular area medio-basally (c); occipital carina usually reduced ventrally, not reaching hypostomal carina (d); mesopleuron partly smooth and shiny (at least between punctures), but largely densely sculptured in *Aleiodes krulikowskii* and some males of *Aleiodes ruficornis*; (usually macropterous, the known brachypterous specimens also belong here)	**Aleiodes apicalis-group**
	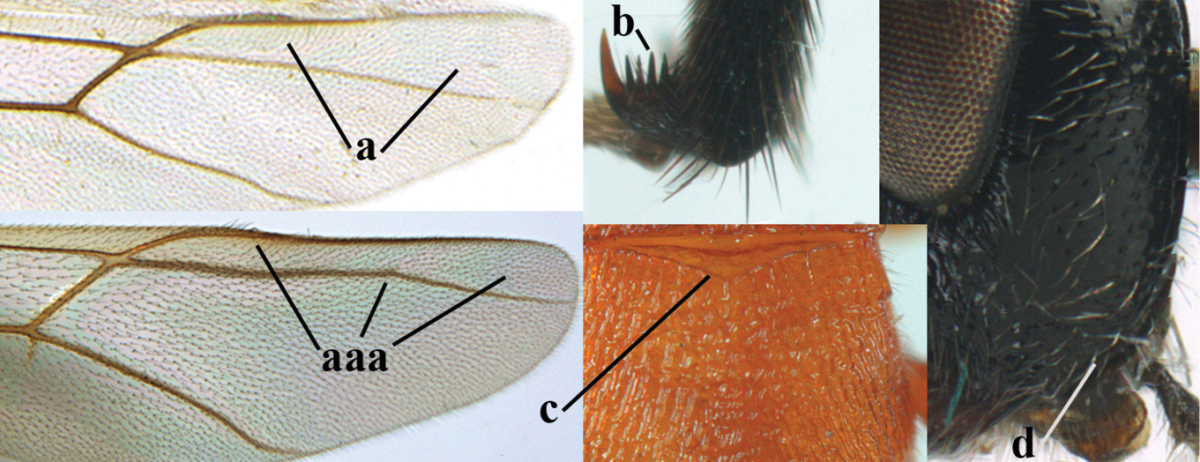	
–	Apical half of marginal cell of hind wing parallel-sided or slightly widened and its maximum width less than 1.8 × its width near hamuli (aa), **if** 1.7–2.7 × (aaa), then mesopleuron largely coriaceous or granulate and tarsal claws at most yellowish pectinate (bb); second tergite without triangular area medio-basally (cc) or this area is narrow or minute (ccc); occipital carina usually complete ventrally, reaching hypostomal carina (dd); mesopleuron usually extensively coriaceous or finely granulate, but medially coarsely sculptured in *Aleiodes bicolor*-group and a few members of *Aleiodes circumscriptus*-group, rarely largely shiny; (only macropterous specimens known)	**4**
	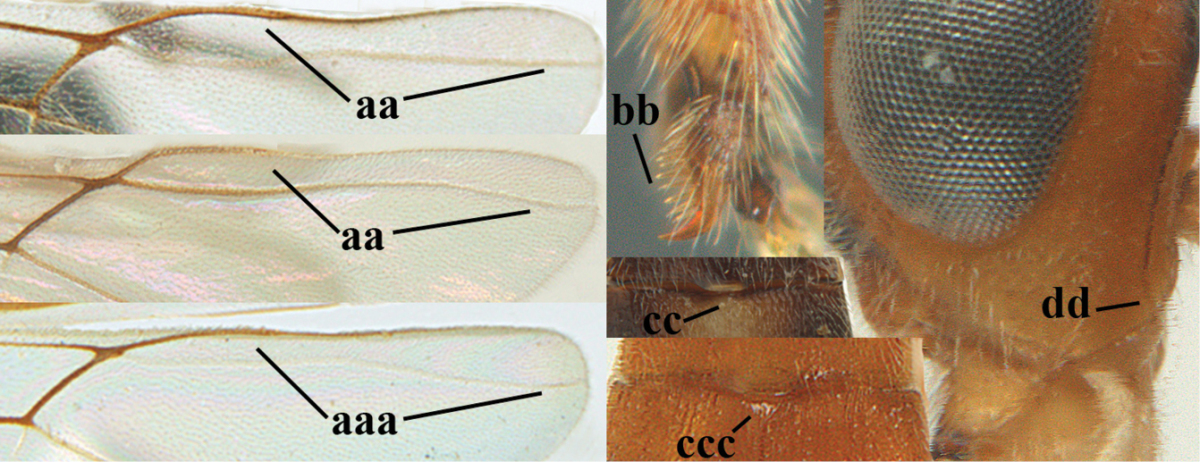	
4	Mesopleuron largely (and often strongly) shiny (a), but may be partly superficially granulate in *Aleiodes testaceus* and *Aleiodes ungularis*, and in *Aleiodes modestus* mesopleuron shiny mainly anteriorly of speculum (aaa); maximum width of hypoclypeal depression 0.3–0.6 × minimum width of face (b)	**5**
	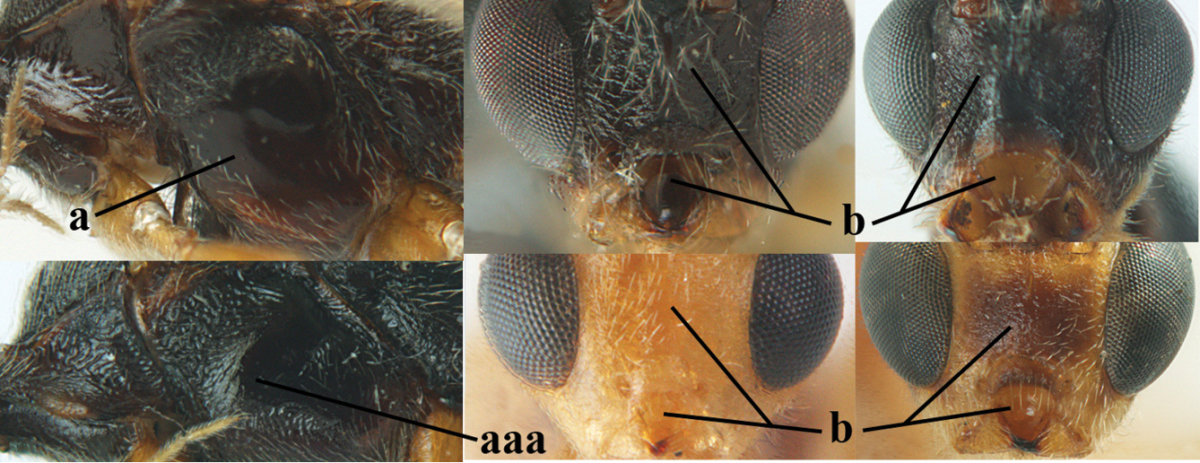	
–	Mesopleuron largely rather matt and distinctly sculptured (aa; at least coriaceous or granulate, though sometimes only weakly so); maximum width of hypoclypeal depression usually 0.3–0.4 × minimum width of face (bb)	**8**
	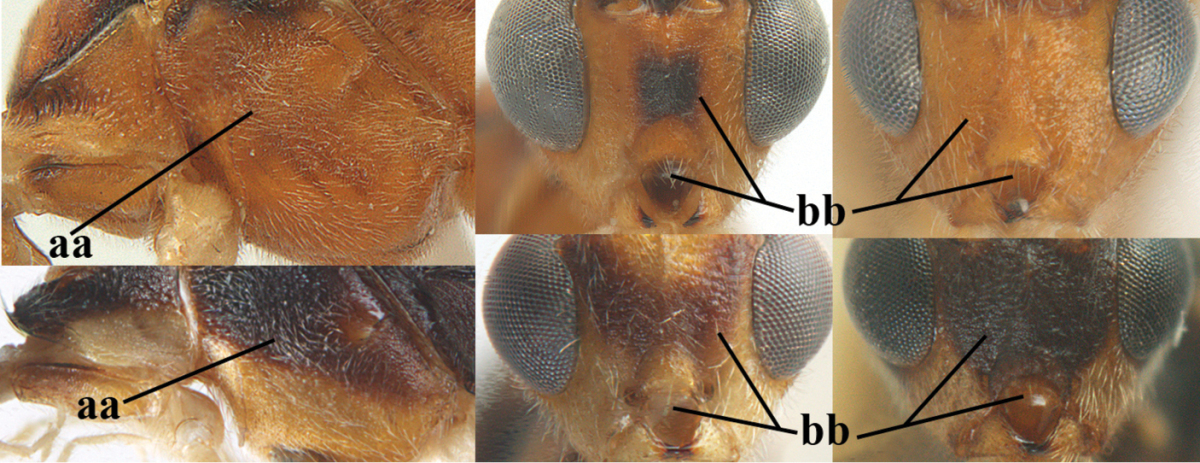	
5	Inner side of hind tibia with comb of whitish bristles apically (a); mesosoma (except propodeum and metapleuron) largely yellowish or yellowish orange (b); precoxal area impressed medially and finely crenulate (c); metasoma dark brown or blackish medially and largely pale yellow laterally (d)	***Aleiodes ungularis* (Thomson, 1892)**
	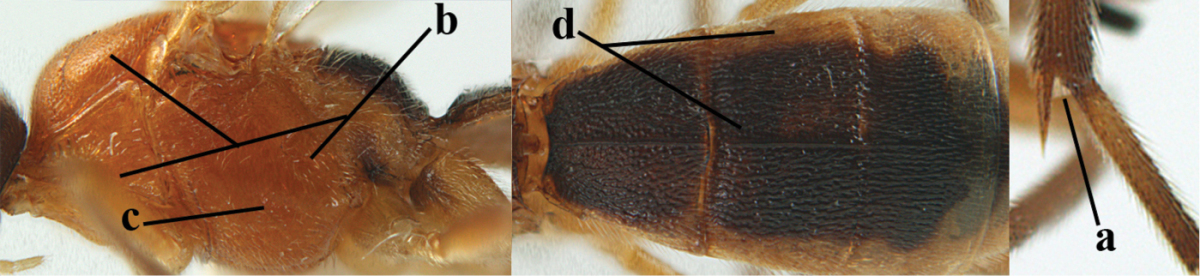	
–	Inner side of hind tibia without comb apically, normally only with bristly setae (aa); mesosoma largely black (bb) except in *Aleiodes testaceus*; precoxal area not impressed and smooth (cc) or rugose (ccc); metasomal tergites practically completely yellowish (dd) or brownish (in *Aleiodes albitibia* sometimes with a large yellow or ivory central patch on second tergite), if blackish or dark brown then also laterally so (ddd)	**6**
	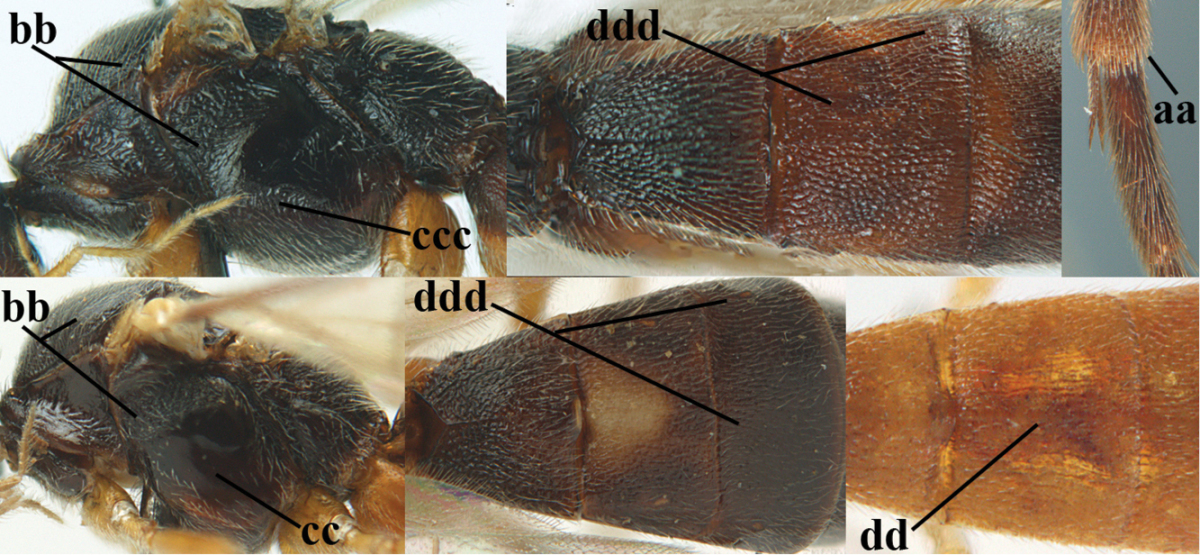	
6	OOL 0.4–0.5 × diameter of posterior ocellus (a); pterostigma dark brown (b); inner hind tibial spur 0.40–0.50 × hind basitarsus (c); third antennal segment robust (d); vein 1r-m of hind wing about as long as vein 1-M (e); area in front of anterior ocellus without tubercle (f); inner side of basal half of hind tibia whitish and contrasting with darker apical half (g), rarely largely dark brown; third tergite about as long as second tergite and curved medio-posteriorly in dorsal view (h)	***Aleiodes albitibia* (Herrich-Schäffer, 1838)**
	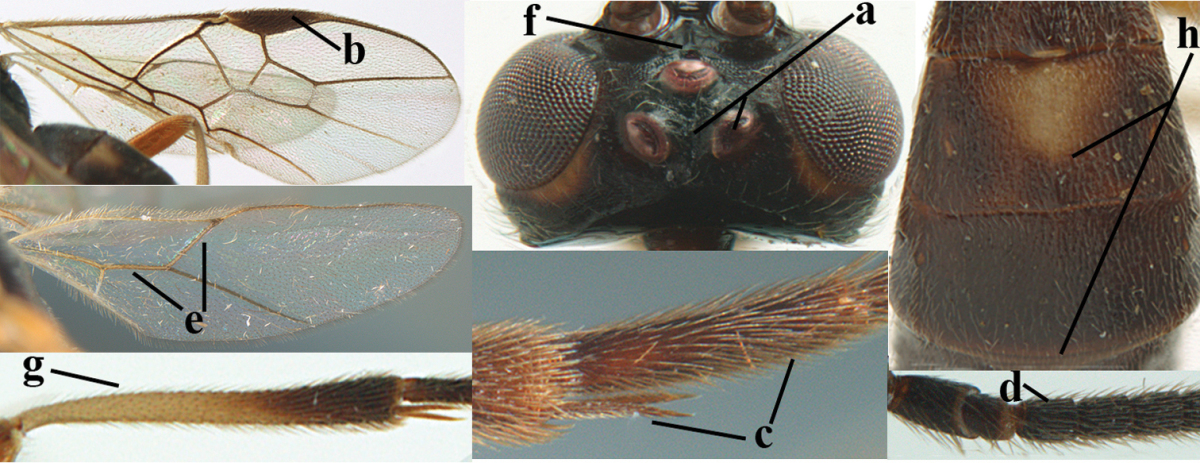	
–	OOL about equal to diameter of posterior ocellus (aa); pterostigma largely yellow (bb), yellowish brown or brown; inner hind tibial spur 0.25–0.30 × hind basitarsus (cc); third antennal segment rather slender (dd); vein 1r-m of hind wing distinctly shorter than vein 1-M (ee); area in front of anterior ocellus with a minute smooth tubercle (ff); inner side of basal half of hind tibia yellowish, similar to apical half (gg); third tergite 0.8 × as long as second tergite and truncate medio-posteriorly in dorsal view (hh)	**7**
	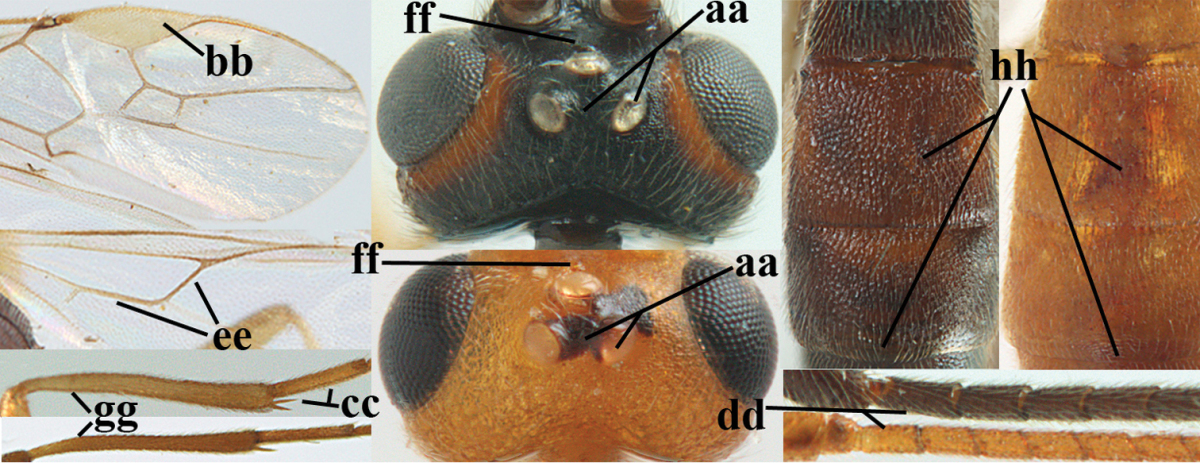	
7	Antennal segments of female 30–35 (of male 34–37); vein r of fore wing 0.7–0.9 × vein 3-SR (a); vein 1-SR of fore wing rather long (b); ventral margin of clypeus thick (c); maximum width of hypoclypeal depression 0.30–0.35 × minimum width of face (d); head and mesosoma largely yellowish brown (e), but mesopleuron dorsally and propodeum usually more or less dark brown; length of malar space of female 0.3–0.4 × height of eye in lateral view (f); third metasomal tergite with more or less developed diverging striae laterally (g)	***Aleiodes testaceus* (Telenga, 1941)**
	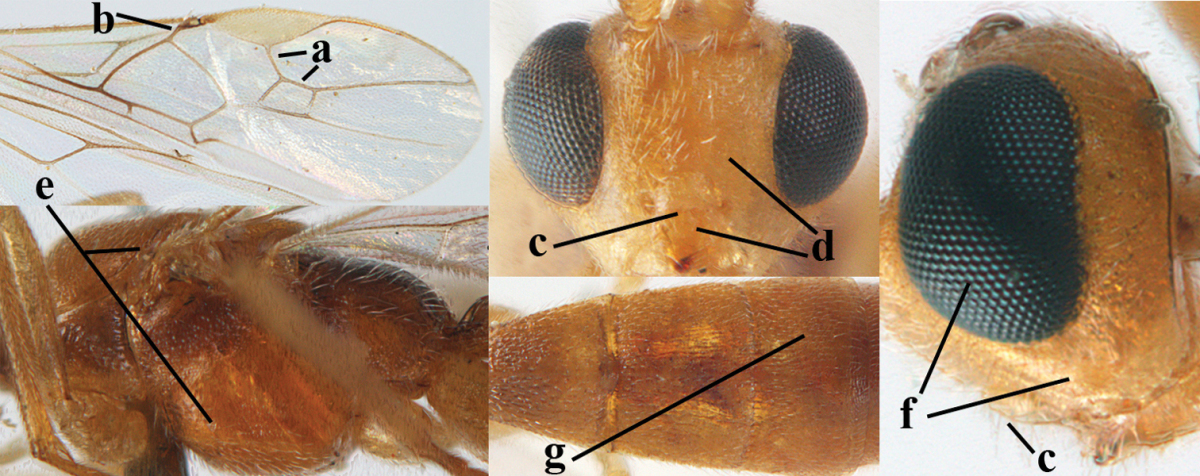	
–	Antennal segments of both sexes (37–)40–45(–47); vein r of fore wing 0.5–0.6 × vein 3-SR (aa); vein 1-SR of fore wing shorter (bb); ventral margin of clypeus thin (cc); maximum width of hypoclypeal depression about 0.5 × minimum width of face (dd); head and mesosoma largely blackish (ee); length of malar space of female 0.5–0.6 × height of eye in lateral view (ff); third tergite without distinct striae (gg)	***Aleiodes modestus* (Reinhard, 1863)**
	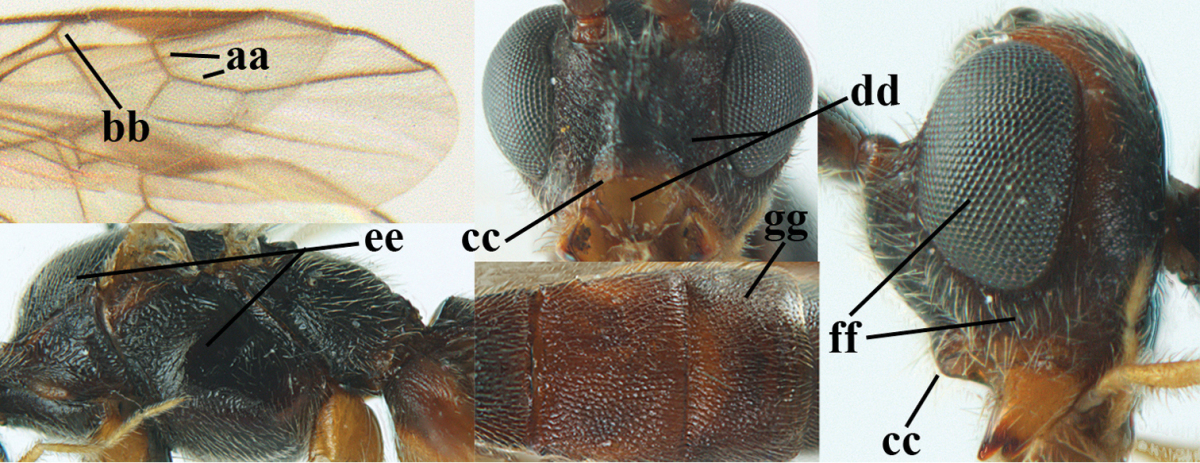	
8	Antennal segments of ♀ 50–71 **and** head usually entirely brownish yellow (a), of ♂ 52–68 (but males of *Aleiodes pallidator* practically unknown); scapus brownish yellow or reddish brown (b) and robust in lateral view (c); [fourth metasomal tergite largely superficially granulate; first tergite lamelliform protruding latero-anteriorly]	**9**
	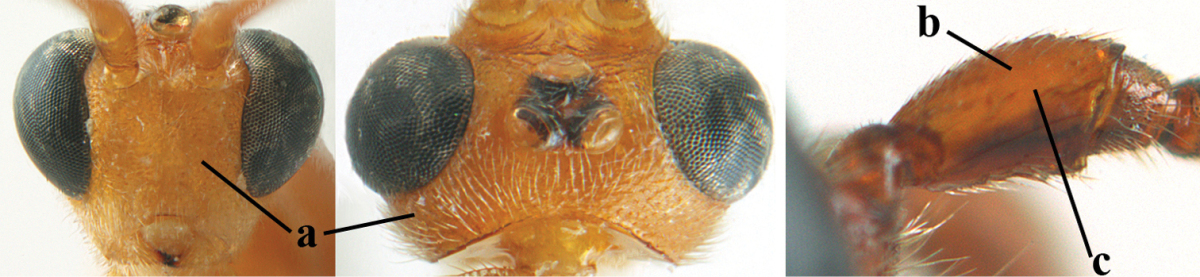	
–	Antennal segments of ♀ 28–51, of ♂ 31–48, **if** antenna with 45–55 segments then head largely blackish medio-dorsally (aa) and/or scapus largely black or dark brown (bb) and less robust in lateral view (cc)	**11**
	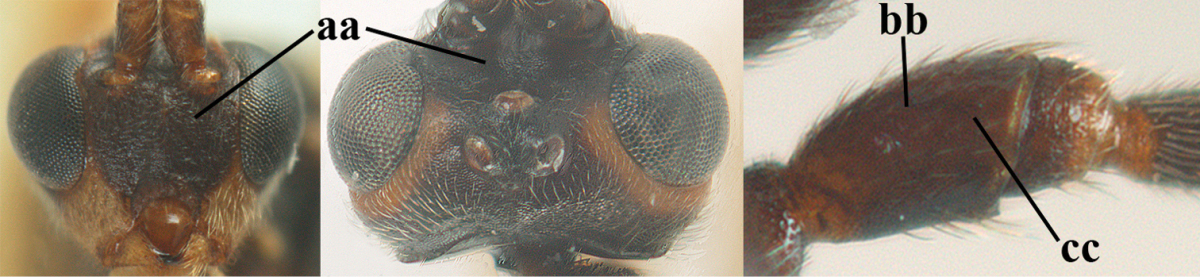	
9	Vein 2-CU1 of fore wing 0.7–1.2 × as long as vein 1-CU1 (a); vein 1-SR weakly angled with vein 1-M (b) and vein 1-M rather curved (c); vein r of fore wing long and subvertical (d); antennal segments of ♀ 56–62; length of fore wing 6–10 mm; [tarsal claws small (e)]	***Aleiodes esenbeckii* (Hartig, 1834)**
	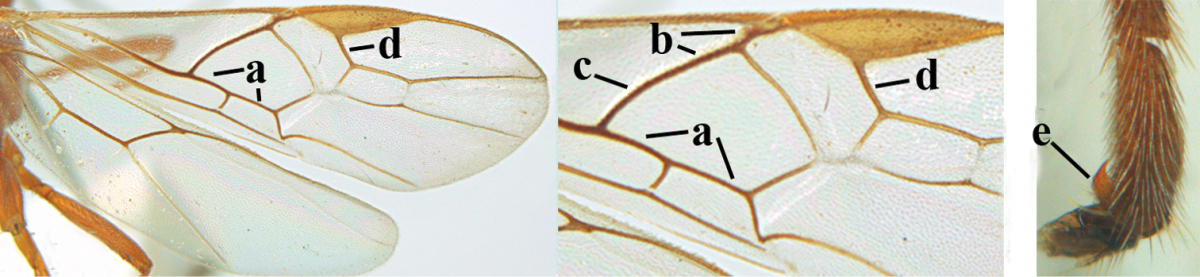	
–	Vein 2-CU1 of fore wing 1.63.0 × longer than vein 1-CU1 (aa); vein 1-SR distinctly angled (bb) or linearly connected to vein 1-M (bbb) and vein 1-M nearly straight (cc); vein r of fore wing medium-sized and oblique (dd); antennal segments of ♀ 51–71, if with 54–55 segments then length of fore wing 5–7 mm	**10**
	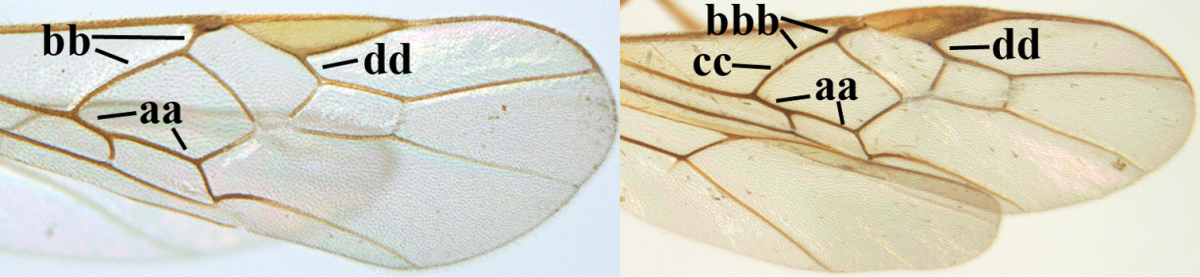	
10	Antennal segments of ♀ 66–71; length of malar space of ♀ 0.4 × height of eye (a); vein 2-CU1 of fore wing 1.6–1.8 × vein 1-CU1 (b); occipital carina reduced ventrally (c)	***Aleiodes varius* (Herrich-Schäffer, 1838)**
	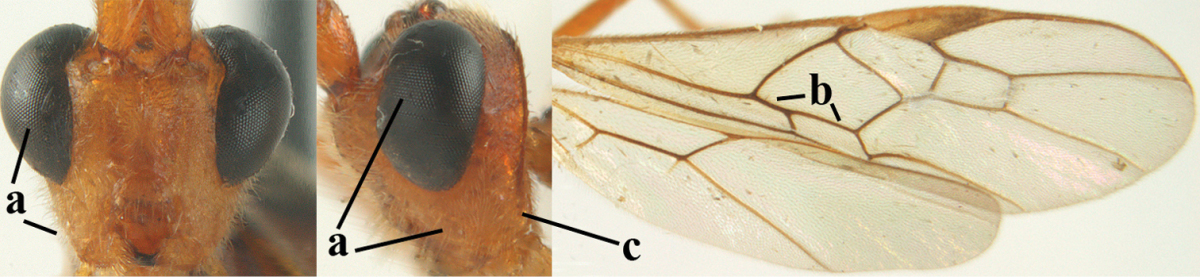	
–	Antennal segments of ♀ 50–55; length of malar space of ♀ 0.3 × height of eye (aa); vein 2-CU1 of fore wing 2.2–3.0 × vein 1-CU1 (bb); occipital carina complete ventrally, reaching hypostomal carina (cc)	***Aleiodes pallidator* (Thunberg, 1822)**
	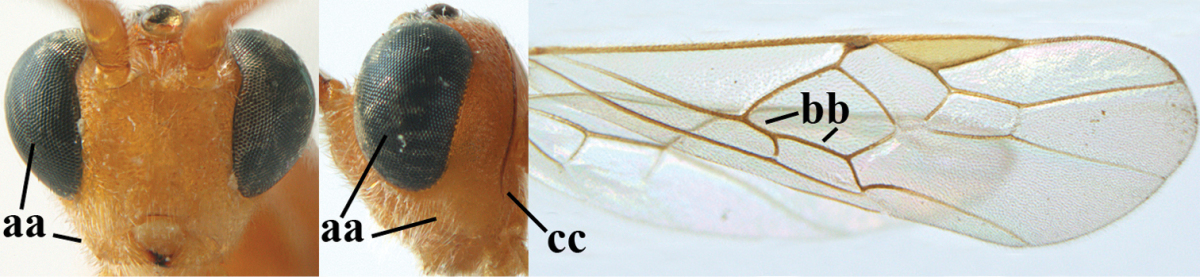	
11	First metasomal tergite lamelliform protruding latero-anteriorly (a) **and** hind trochantellus of female slender, its ventral length 2.2–2.9 × its width (b); fore wing rather narrow (c); speculum of mesopleuron often rugose and with satin sheen, largely reticulate or granulate (d)	**12**
	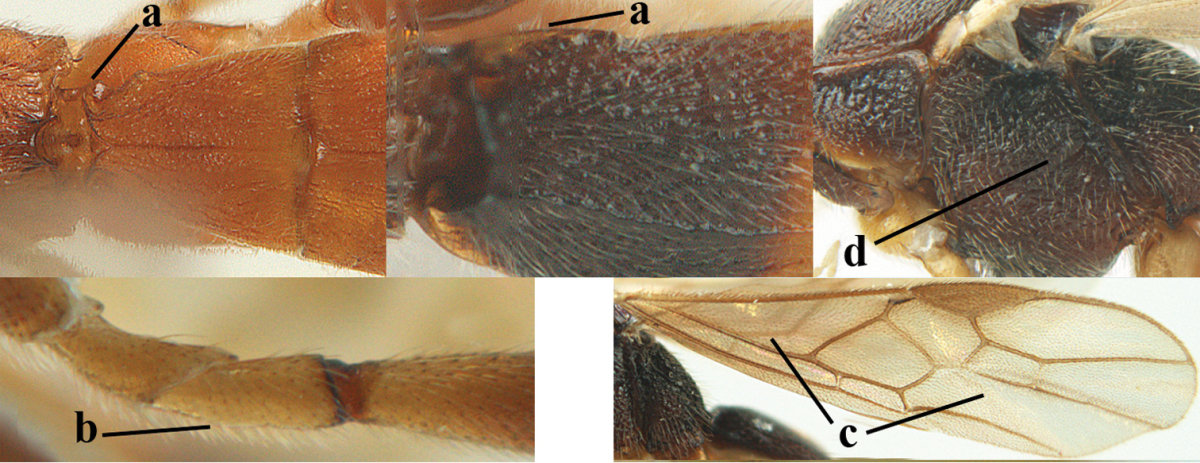	
–	First tergite less lamelliform protruding latero-anteriorly (aa); **if** lamella present up to spiracle and rather protruding then hind trochantellus of female moderately robust, its ventral length less than 2.4 × its width (bb), **if** rarely up to 2.6 × then fore wing moderately wide (cc) and speculum of mesopleuron shiny and (partly) smooth or granulate (dd)	**15**
	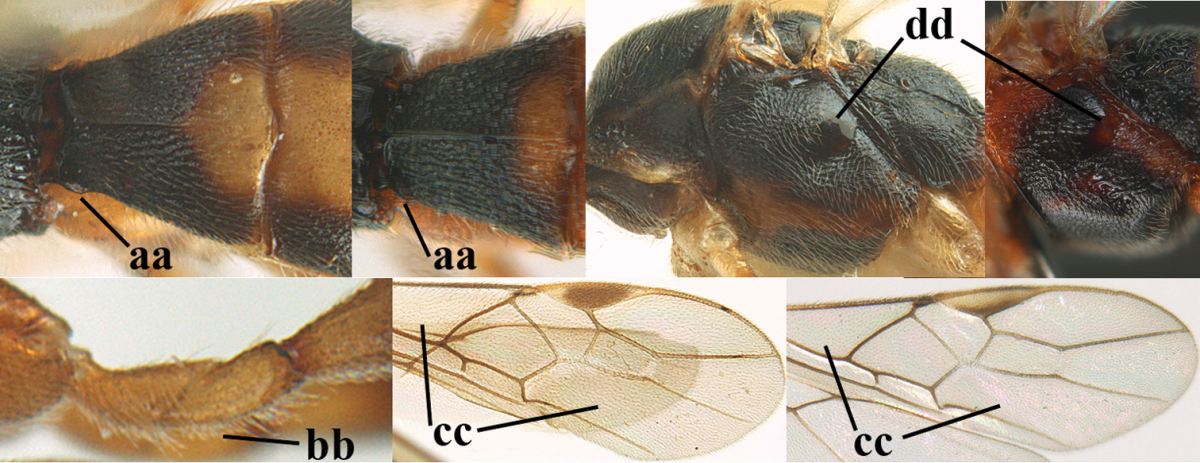	
12	Tarsal claws with distinct fine pecten (a); scapus and pedicellus of ♀ at least partly blackish, contrasting with yellowish middle of antenna (b); length of malar space of ♀ 0.25–0.30 × height of eye in lateral view (c; of ♂ 0.30 times); [fourth metasomal tergite of ♀ black latero-posteriorly (of ♂ brownish yellow); antenna of ♀ in dorsal view bicoloured, first–eighth and 48th–49th segments more or less dark brown, remainder of antenna yellowish, of ♂ entire antenna yellowish]	***Aleiodes apiculatus* (Fahringer, 1932)**
	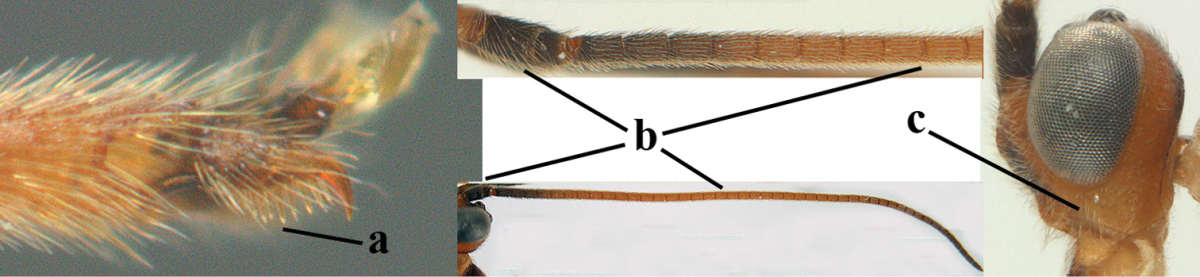	
–	Tarsal claws only bristly setose, without distinct pecten (aa); scapus and pedicellus of ♀ similarly coloured as medial fifth of antenna or paler (bb); length of malar space of ♀ 0.30–0.50 × height of eye in lateral view (cc; of ♂ 0.25 times, but males of *Aleiodes angustipterus* unknown)	**13**
	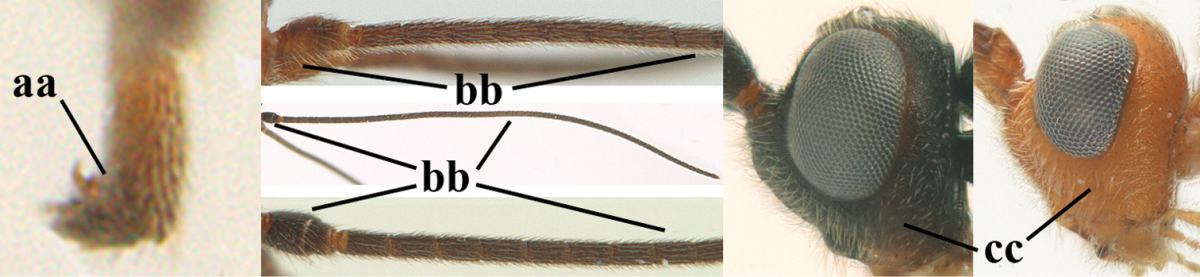	
13	Antennal segments of ♀ 49–52; hind tibia infuscate subapically, contrasting with yellowish apex of tibia (a); second tergite comparatively long (b); fourth tergite with distinct sharp lateral crease (c) and basally rugulose or rugose (d); pterostigma bicoloured, with its basal third pale yellow (e); [vein m-cu of fore wing straight and angled to vein 2-CU1; antenna of ♀ sometimes with a narrow white or pale yellowish submedial band]	***Aleiodes jakowlewi* (Kokujev, 1898)**
	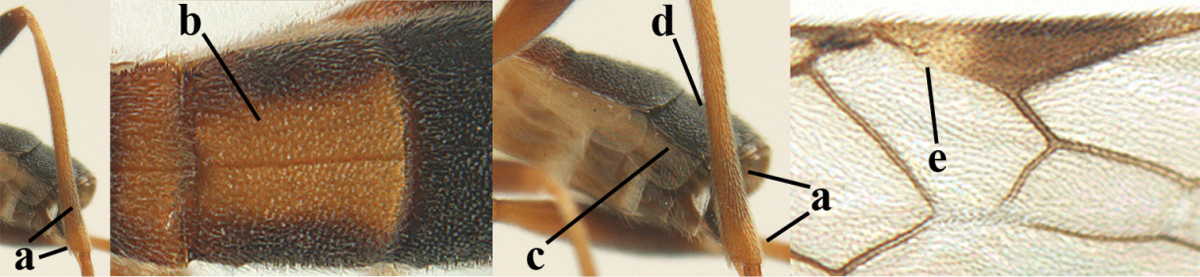	
–	Antennal segments of ♀ 34–40; hind tibia subapically and apically similarly coloured and brownish yellow (aa); second tergite comparatively short (bb); fourth tergite partly without distinct sharp lateral crease (cc), partly retracted and tergite largely smooth (dd); pterostigma unicoloured yellowish brown or dark brown (ee)	**14**
	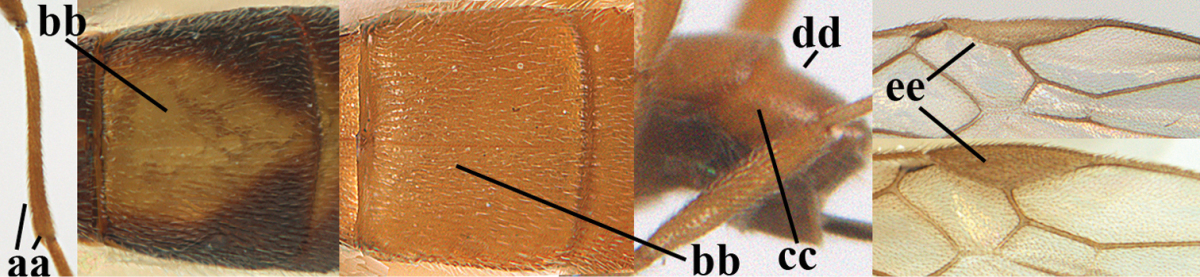	
14	Length of malar space of ♀ 0.4 × height of eye in lateral view (a); vein m-cu of fore wing slightly curved towards vein 2-CU1, meeting it at about 140° (b); pterostigma dark brown (c); fore wing distinctly infuscate (d); inner side of hind tibia without apical comb (e); speculum sculptured (f); occipital carina complete or narrowly interrupted dorsally (g)	***Aleiodes angustipterus* sp. n.**
	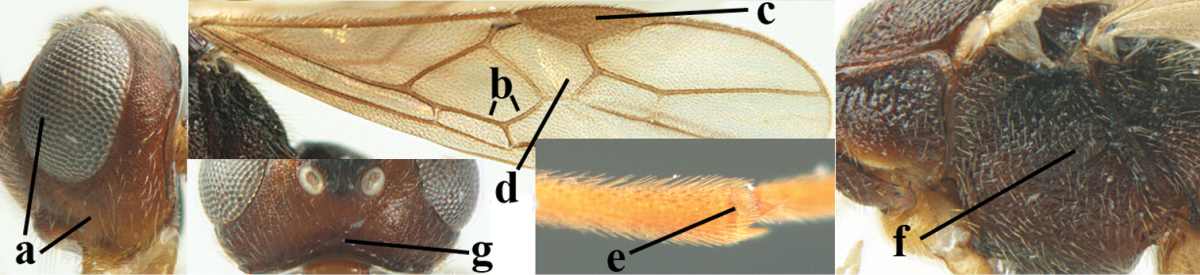	
–	Length of malar space of ♀ 0.5 × height of eye in lateral view (aa); vein m-cu of fore wing straight and angled to vein 2-CU1 (bb); pterostigma yellowish (cc); fore wing subhyaline (dd); inner side of hind tibia with weakly developed apical comb (ee); speculum partly smooth and shiny (ff); occipital carina widely interrupted dorsally (gg)	***Aleiodes carminatus* sp. n.**
	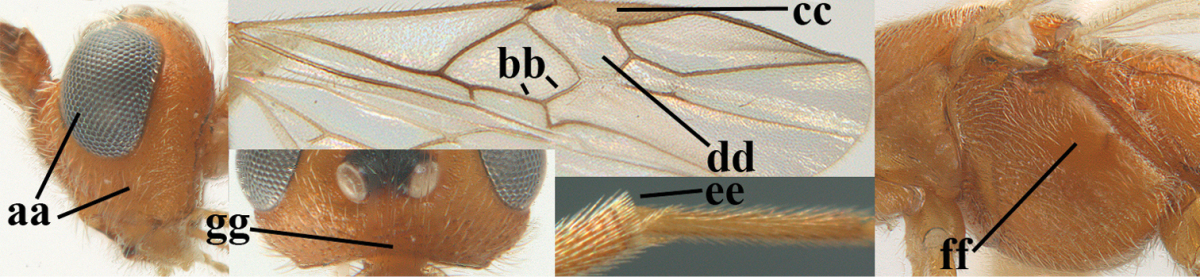	
15	Fourth metasomal tergite curved posteriorly in dorsal view (a) and following tergites more or less retracted (b); vein r of fore wing 0.6–0.8 × vein 3-SR (c), precoxal sulcus largely granulate or coriaceous (d); trochanters, trochantelli and pterostigma largely black(ish) (e); [vein m-cu of hind wing distinct; formerly *Tetrasphaeropyx* Ashmead, 1889. Associated with Macariini (Geometridae)]	**16**
	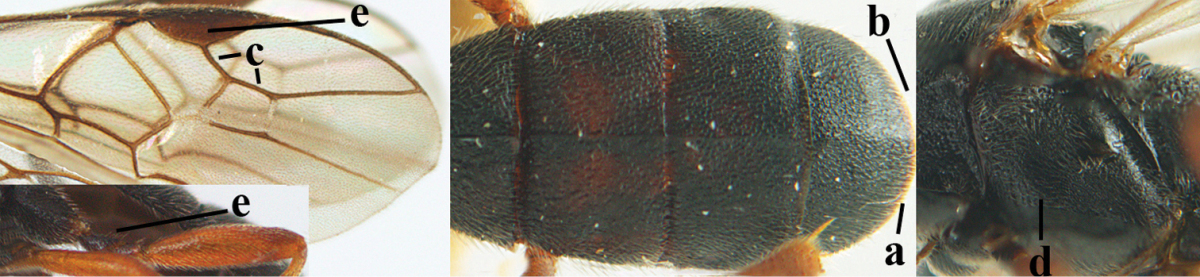	
–	Fourth tergite subtruncate medio-posteriorly in dorsal view (aa) and following segments at least partly exposed (bb); vein r of fore wing 0.2–0.6 × vein 3-SR (cc), **if** 0.6–0.8 × (ccc) then precoxal area coarsely rugose medially (dd) and third tergite enlarged and flattened (*Aleiodes hergeri* Papp); trochanters, and trochantelli usually brownish yellow (ee) and colour of pterostigma variable (ee, eee)	**18**
	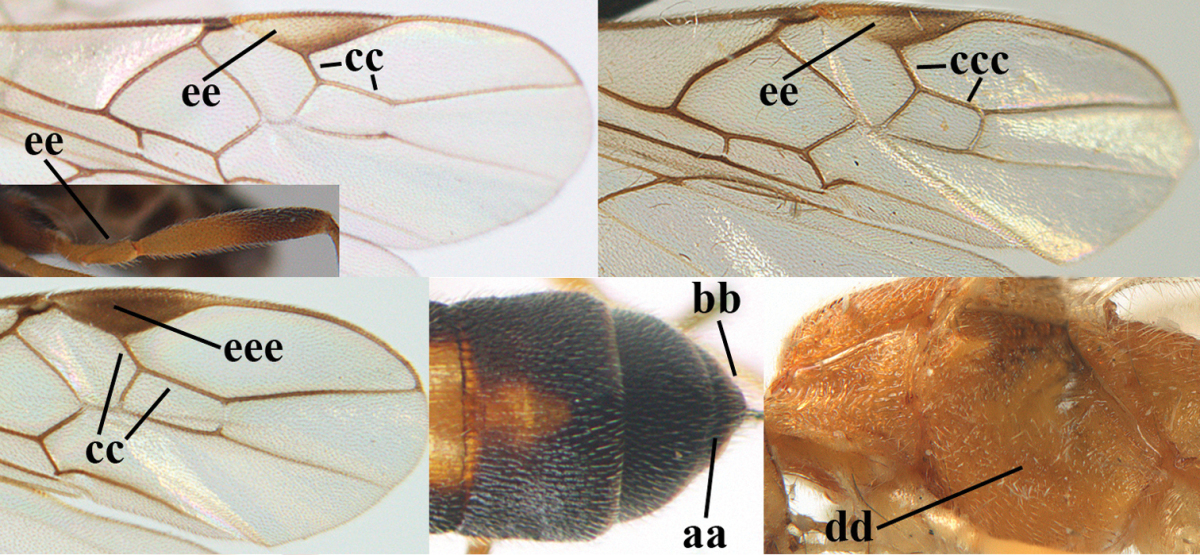	
16	Mesoscutum (a), orbita (b) and malar space (c) largely yellowish brown; all femora and tibiae black or dark brown (d); fore and hind femora slender (e); vein 1-SR of fore wing angled with vein 1-M (f)	***Aleiodes artesiariae* sp. n.**
	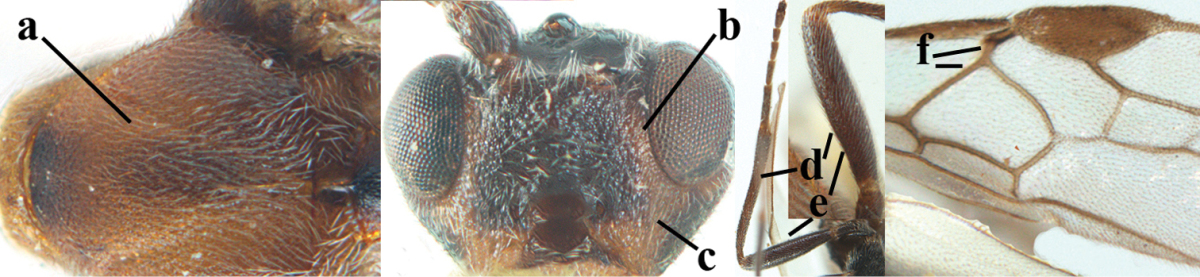	
–	Mesoscutum (aa), orbita (bb) and malar space (cc) black; all femora and tibiae reddish or yellowish brown (dd); fore and hind femora less slender (ee); vein 1-SR of fore wing sublinear with vein 1-M (ff)	**17**
	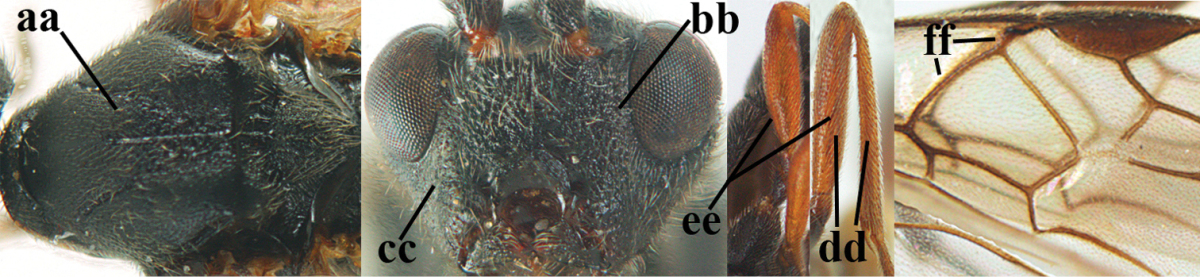	
17	Vein M+CU1 of fore wing apically at about same level as vein 2-CU1 (a); vein r of fore wing 0.6–0.9 × vein 3-SR (b); length of fore wing 3.4–3.7 mm; arctic and alpine sp.	***Aleiodes arcticus* (Thomson, 1892)**
	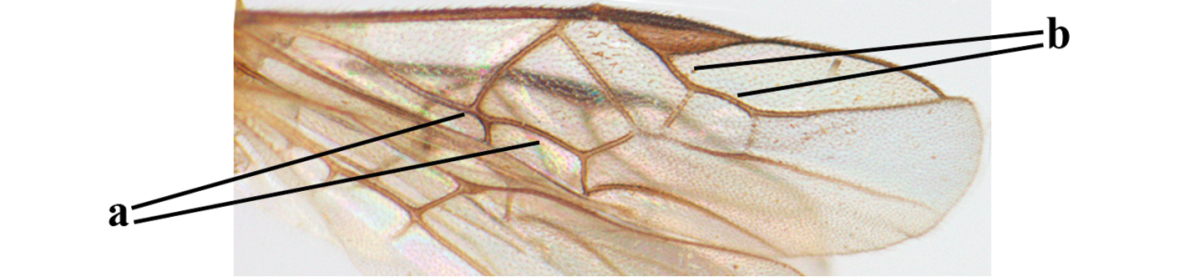	
–	Vein M+CU1 of fore wing apically above level of vein 2-CU1 (aa); vein r of fore wing 0.9–1.1 × vein 3-SR (bb); length of fore wing 3.9–4.7 mm; lowland sp.	***Aleiodes reticulatus* (Noskiewicz, 1956), stat. rev.**
	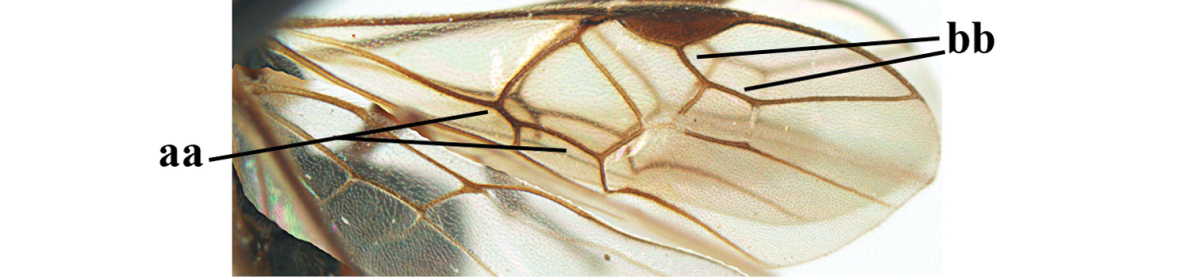	
18	Head subglobose in dorsal view (a) and high in anterior view (b); dorsal face of propodeum long and (slightly) angularly protruding postero-laterally (c); fore femur stout (d); [antennal segments of ♀ 28–35, stout; strongly sexually dimorphic, male with large ocelli and slender antennal segments and antenna with 39–41 segments; body completely yellowish; length of antenna of ♀ 0.9–1.1 × fore wing, longer in ♂; second submarginal cell of fore wing rather narrow]	***Aleiodes curticornis* nom. n.**
	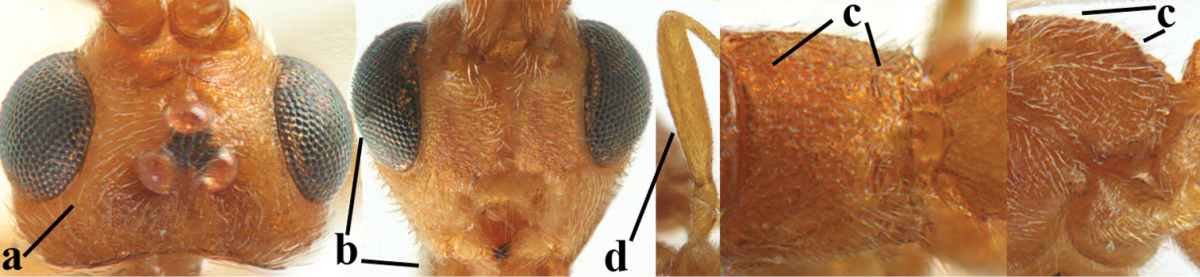	
–	Head transverse in dorsal view (aa) and lower in anterior view (bb); dorsal face of propodeum shorter and rounded posteriorly (cc), **if** more elongate then fore femur slenderer (dd)	**19**
	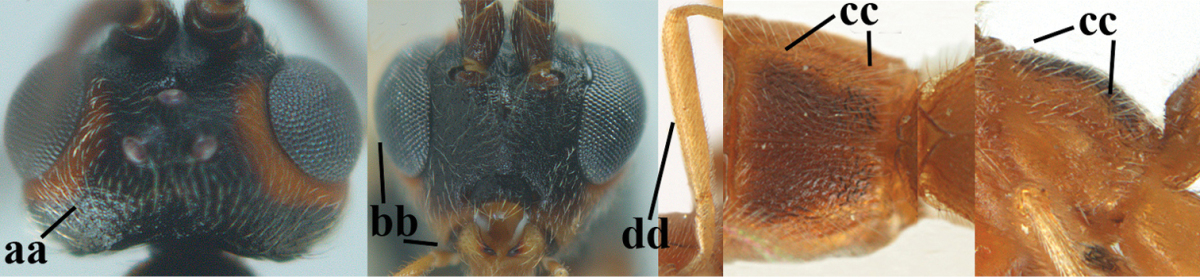	
19	Fourth metasomal tergite with a more or less sharp lateral crease for its whole length (a), **if** weak or absent then length of malar space of female 0.5–0.6 × height of eye in lateral view (b); precoxal area distinctly (and usually coarsely) rugose medially (c); epicnemial area (d) and propodeum (e) coarsely rugose; fourth metasomal tergite at least basally distinctly sculptured (f)	***Aleiodes bicolor*-group**
	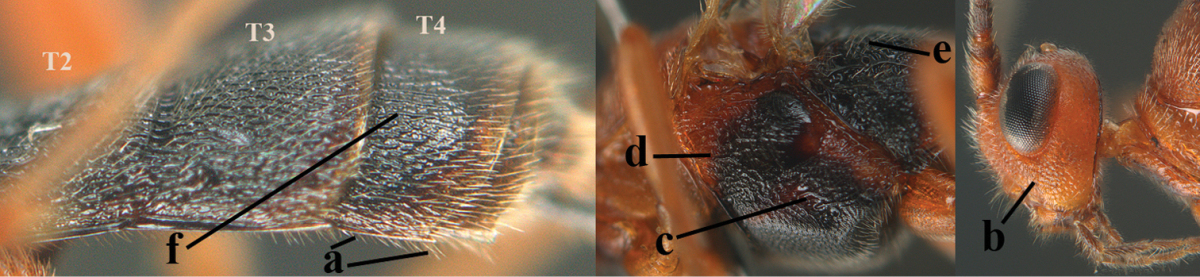	
–	Fourth tergite gently folded laterally, without acute lateral crease or this only anteriorly developed (aa), although rarely present as a simple, non-lamelliform crease to apex of tergite; length of malar space of female less than 0.5 × height of eye in lateral view (bb); precoxal area (cc) and epicnemial area less rugose (dd); propodeum (ee) usually with few rugae or completely coriaceous; fourth tergite usually mainly smooth with some superficial sculpture (ff)	**20**
	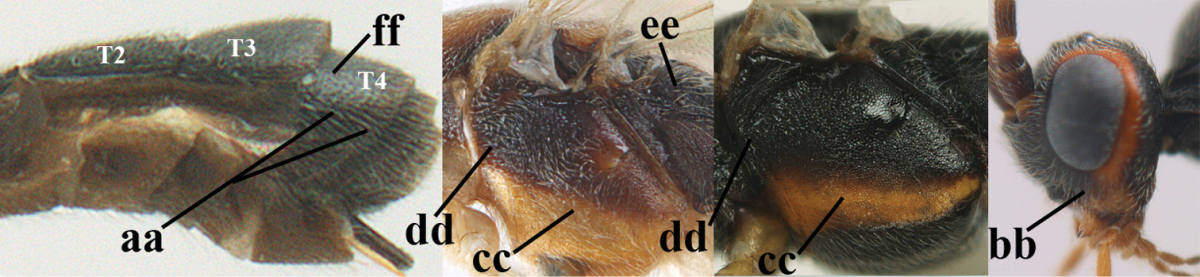	
20	Inner apex of hind tibia with distinct comb (a); surroundings of veins 1-M and 1-SR of fore wing more or less infuscate and darker than surroundings (b); metasoma usually richly patterned (c); fourth tergite of ♀ pale (ivory-)yellowish latero-posteriorly (d), in ♂ usually infuscate; base of hind tibia usually narrowly dark brown (e); [antennal segments of ♀ (35–)44–50, of ♂ (42–)48–54; length of malar space 0.2–0.4 × height of eye in lateral view (f); temple narrow and directly narrowed behind eyes (g); length of hind femur of ♀ 5.1–6.5 × its width (of ♂ up to 8 times)]	***Aleiodes seriatus* (Herrich-Schäffer, 1838) s.l.**
	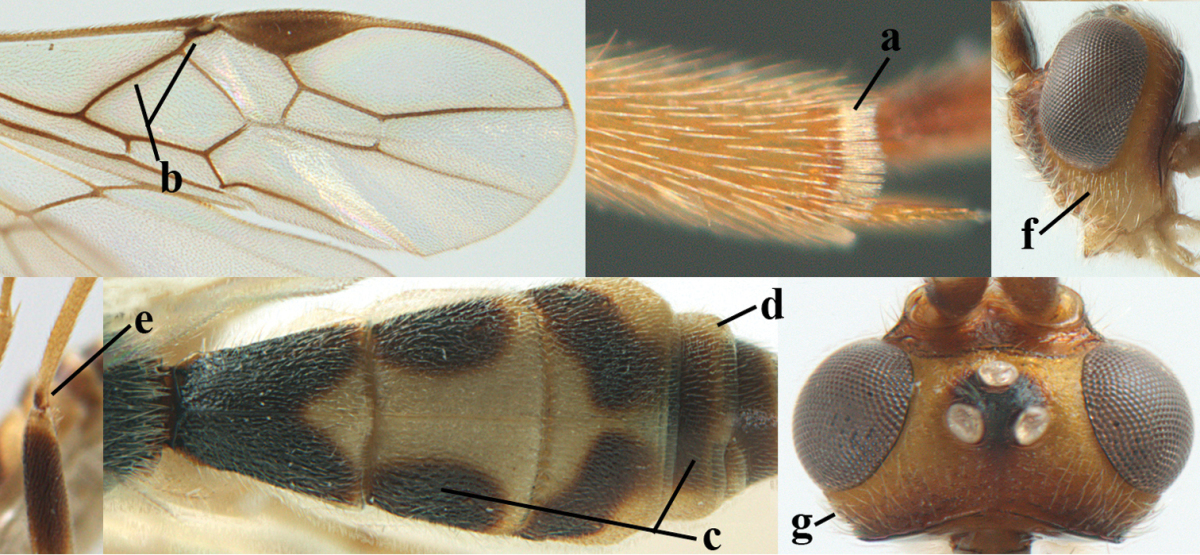	
–	Inner apex of hind tibia without distinct comb (aa); surroundings of veins 1-M and 1-SR of fore wing subhyaline and similar to surroundings (bb); metasoma less patterned (cc); fourth tergite of ♀ dark brown or yellowish brown latero-posteriorly (dd); base of hind tibia usually yellowish brown (ee); [malar space (ff) and head shape variable]; ***Aleiodes circumscriptus***-group	**21**
	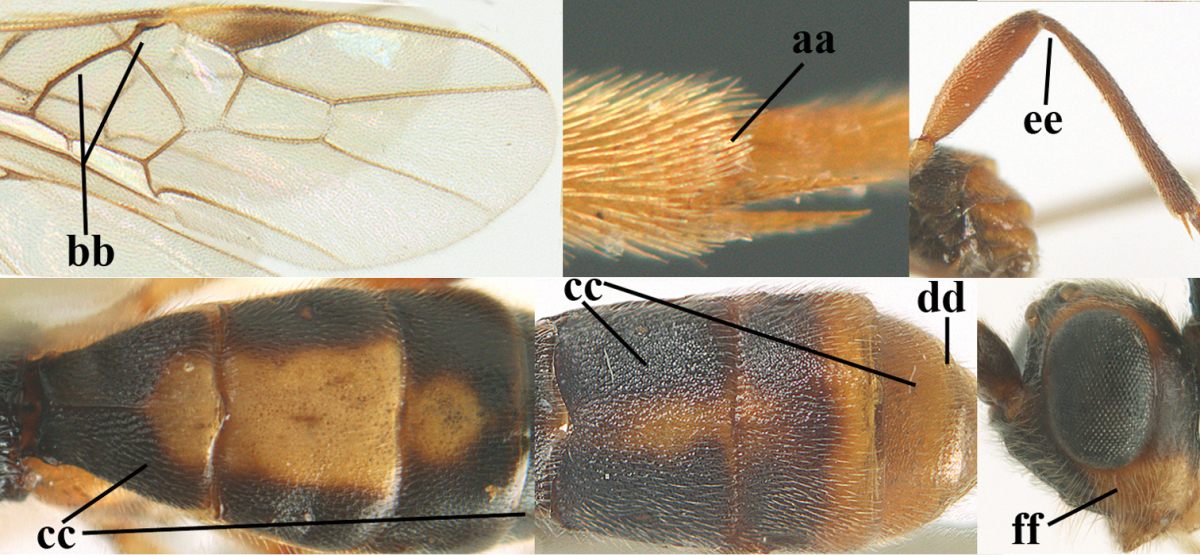	
21	Antennal segments of ♀ 42–49, of ♂ 42–46; temple directly narrowed behind eyes in dorsal view (a), mesosoma black(ish) dorsally (especially mesoscutum and scutellum (b), but sometimes notaulic area or scutellum brownish) **and** apical half of metasoma largely blackish (c); hind femur usually orangeish brown (d); [second tergite with (pale) yellowish elliptical patch medially (e). If body pale yellowish, antenna with 47–51 segments, head coarsely sculptured dorsally and fourth and following tergites largely under enlarged and flattened third tergite (f), cf. *Aleiodes hergeri* Papp, 1989]	**22**
	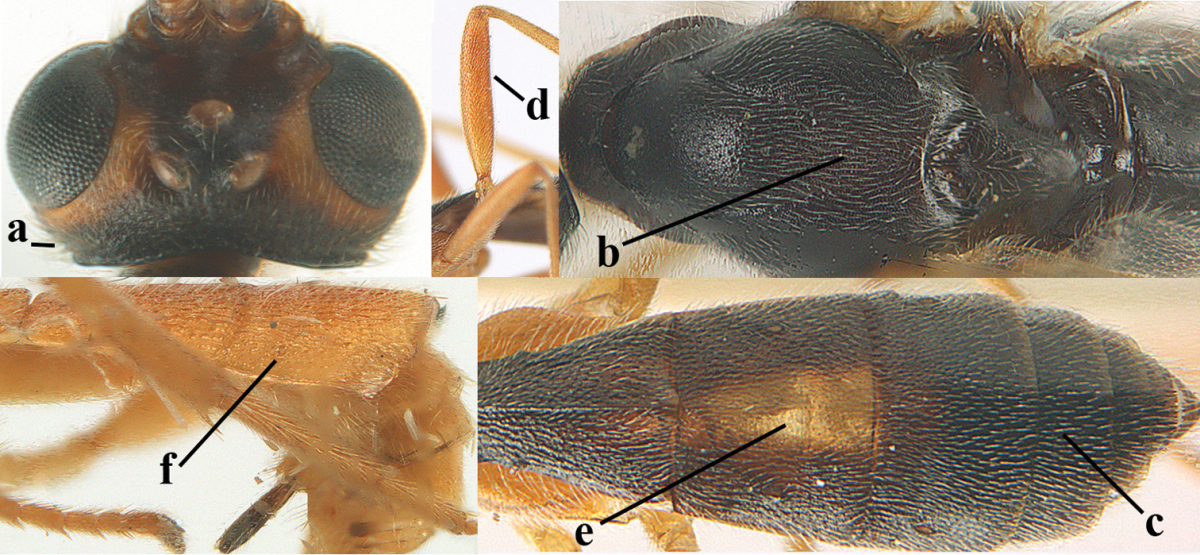	
–	Antennal segments of ♀ 27–46, of ♂ 31–48; **if** antennal segments of ♀ 42–46 then temple gradually narrowed behind eyes in dorsal view (aa) **or** mesosoma partly dorsally (bb) and apical half of metasoma yellowish brown (cc); hind femur variable, often yellowish or partly strongly darkened (dd)	**23**
	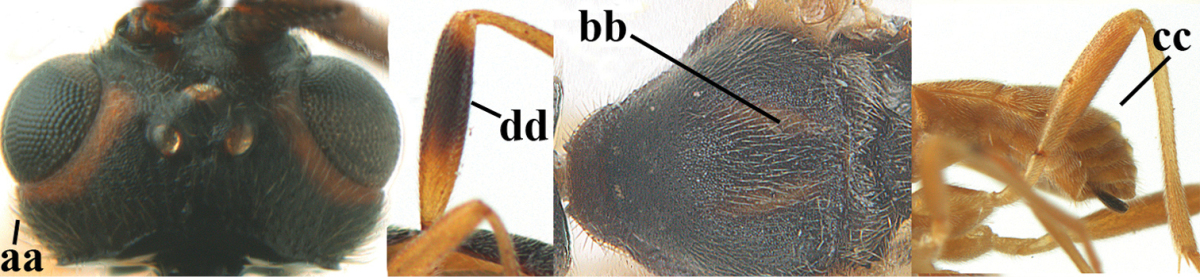	
22	Fore femur of ♀ less slender, 5.4–5.7 × as long as wide (a) and hardly sculptured, but of males slenderer; scapus and pedicellus (yellowish) brown ventrally (b); precoxal area frequently with some rugae or rugulae (c); propodeum distinctly transversally rugose medially and median carina largely absent on posterior half of propodeum or irregular (d); posterior half of pterostigma of ♀ largely dark brown (e); ivory part of malar space usually reaching clypeus, sometimes extending to lower part of inner orbit (f); mesosternum more or less blackish or dark brown (g), rarely completely reddish; [antennal segments of ♀ 42–47, of ♂ 42–46]	***Aleiodes circumscriptus* (Nees, 1834) s.s.**
	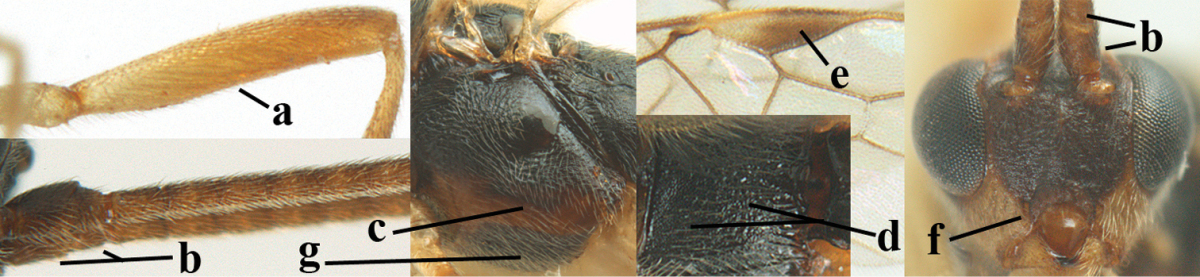	
–	Fore femur slender, (6.0–)6.7–7.4 × as long as wide (aa) and very finely sculptured; scapus and pedicellus more or less infuscate or black ventrally (bb); precoxal area usually without rugae (cc); propodeum largely coriaceous medially and median carina at least anteriorly present on posterior half of propodeum and regular (dd); posterior half of pterostigma of ♀ more or less yellowish (ee), but usually apical third laterally darkened; pale yellowish part of malar space usually not reaching clypeus (ff); mesosternum frequently reddish or brownish (gg); [antennal segments of ♀ 44–49, of ♂ 43–47]	***Aleiodes nigricornis* Wesmael, 1838**
	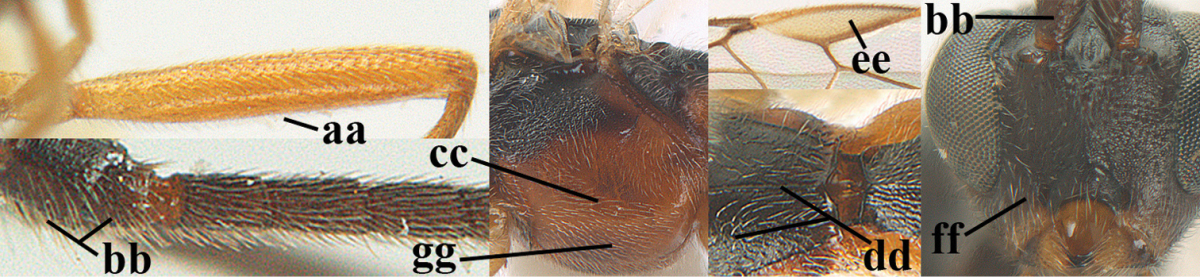	
23	Hind femur often partly, mesonotum dorsally and/or head largely dark brown or blackish (a); OOL and POL of ♀ 1.2–1.7 × diameter of posterior ocellus (b), but less in *Aleiodes cantherius* and *ryrholmi* (bbb); antennal segments of ♀ 36–45, of ♂ 34–45; metasoma with medial ivory patch (c), but less developed or absent in *Aleiodes diarsianae* and *nigriceps* (ccc)	**24**
	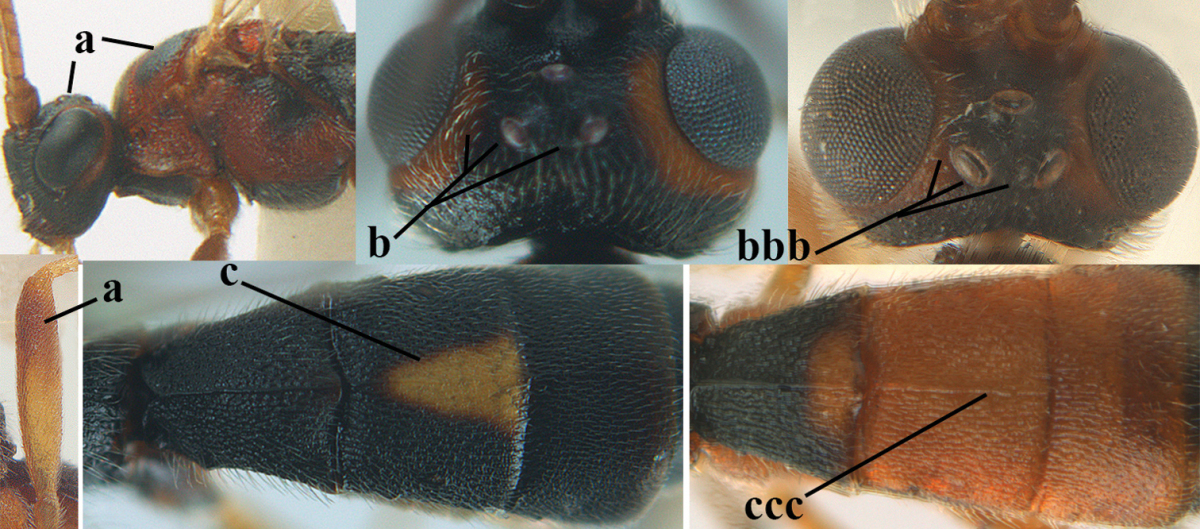	
–	Hind femur completely, mesonotum dorsally and head largely yellowish (aa); **if** these parts are strongly darkened then OOL and POL of ♀ about equal to diameter of posterior ocellus (bb) and/or antennal segments of ♀ (31–)33–37, rarely up to 40, of ♂ 34–39(–41) and metasoma without ivory patch, brownish yellow, brown or dark brown (cc)	**30**
	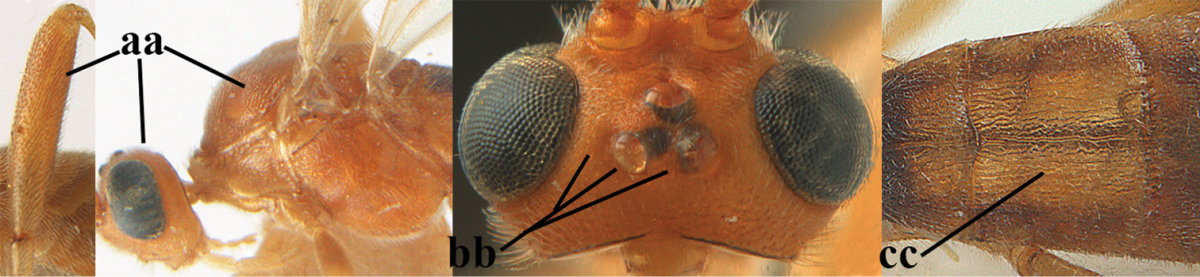	
24	Head behind eyes directly narrowed in dorsal view (a); ocelli large (b); apex of metasoma of ♀ brownish yellow (c); temple short (d)	**25**
	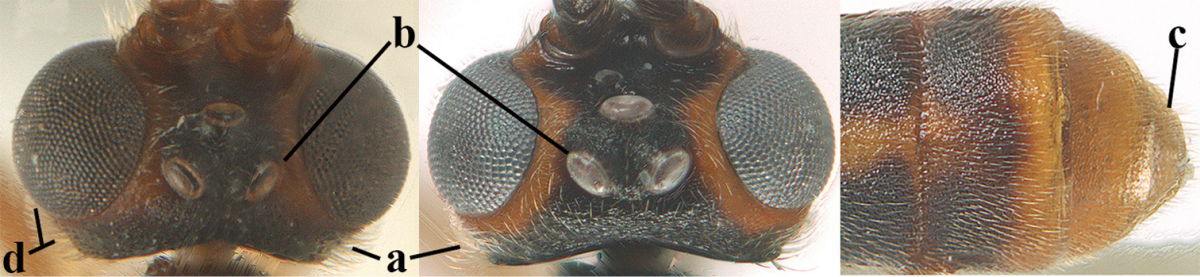	
–	Head behind eyes gradually (roundly) narrowed in dorsal view (aa); ocelli medium-sized (bb); apex of metasoma of ♀ black or dark brown (cc); temple medium-sized (dd)	**26**
	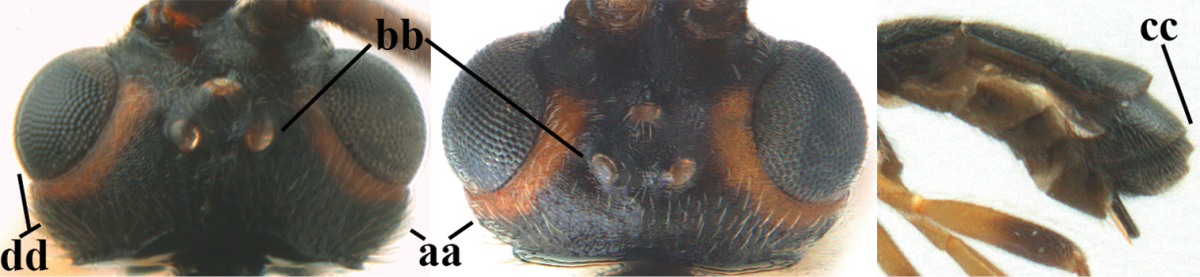	
25	Face yellowish brown (a); ocelli smaller, POL 0.8 × as wide as diameter of posterior ocellus (b); pale area of second tergite wide (c); mesoscutum with pair of yellowish brown stripes (d; but in males sometimes only vaguely indicated); medio-posterior depression of metanotum wide (e); palpi pale yellowish (f); vein cu-a of fore wing subvertical (g); face mainly transversely rugose (h)	***Aleiodes cantherius* (Lyle, 1919)**
	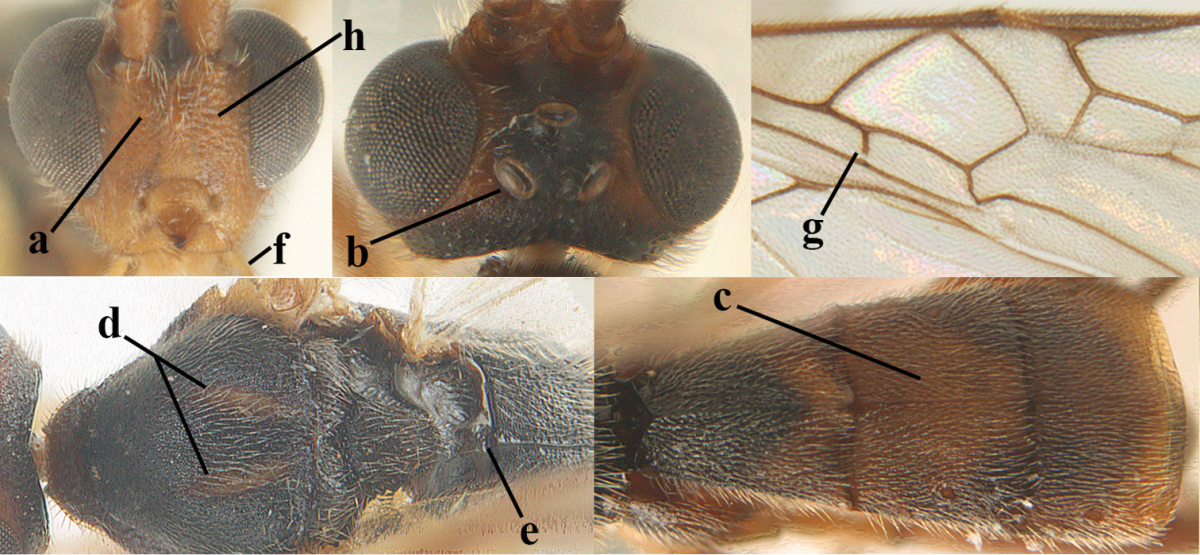	
–	Face black (aa); ocelli larger, POL 0.6 × as wide as diameter of posterior ocellus (bb); pale area of second tergite narrow (cc); mesoscutum entirely black (dd); medio-posterior depression of metanotum rather narrow (ee); palpi mainly dark brown (ff); vein cu-a of fore wing inclivous (gg); face superficially rugulose (hh)	***Aleiodes ryrholmi* sp. n.**
	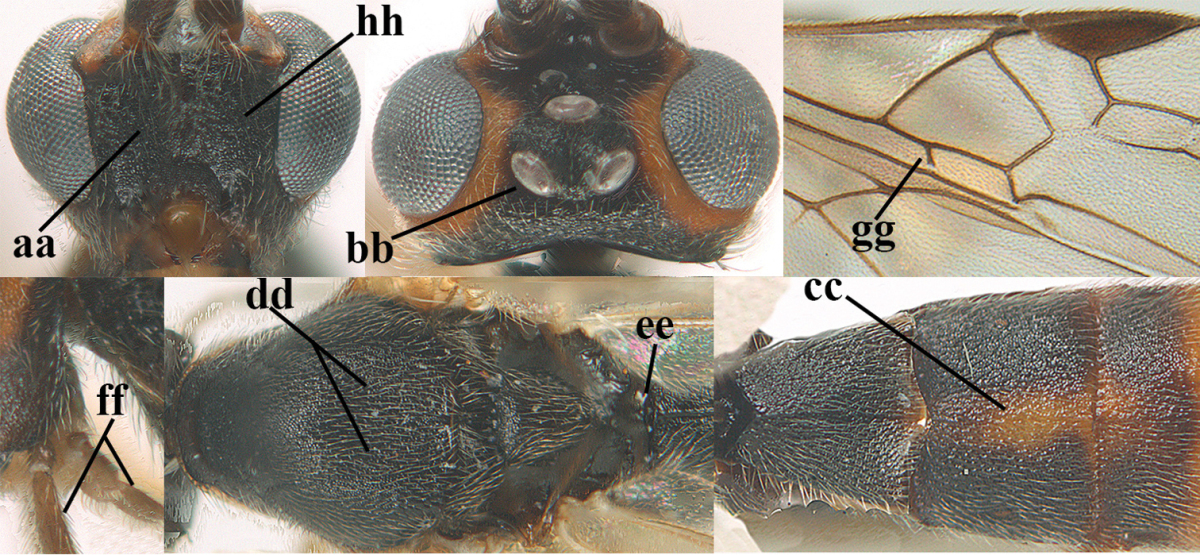	
26	Length of fore femur 6.4–8.0 × its maximum width (a) and hind femur (centrally and subapically) parallel-sided (b); hind femur slender basally (c); mesosternum usually black(ish) (d); temple normal (e); scapus ventrally and usually basal half of antenna (dark) brown (f), rarely yellowish; hind femur basally largely yellowish and slightly infuscate subapically (g), paler than ventral side of scapus; **if** hind femur is distinctly infuscate then often also extreme base of hind tibia infuscate (h); [face usually black or dark brown medially and near eyes yellowish brown, but sometimes completely black or rarely completely yellowish; antennal segments of ♂ 35–40(–41), usually 36–38, less than of ♀, which has usually 37–39 segments]	***Aleiodes leptofemur* sp. n.**
	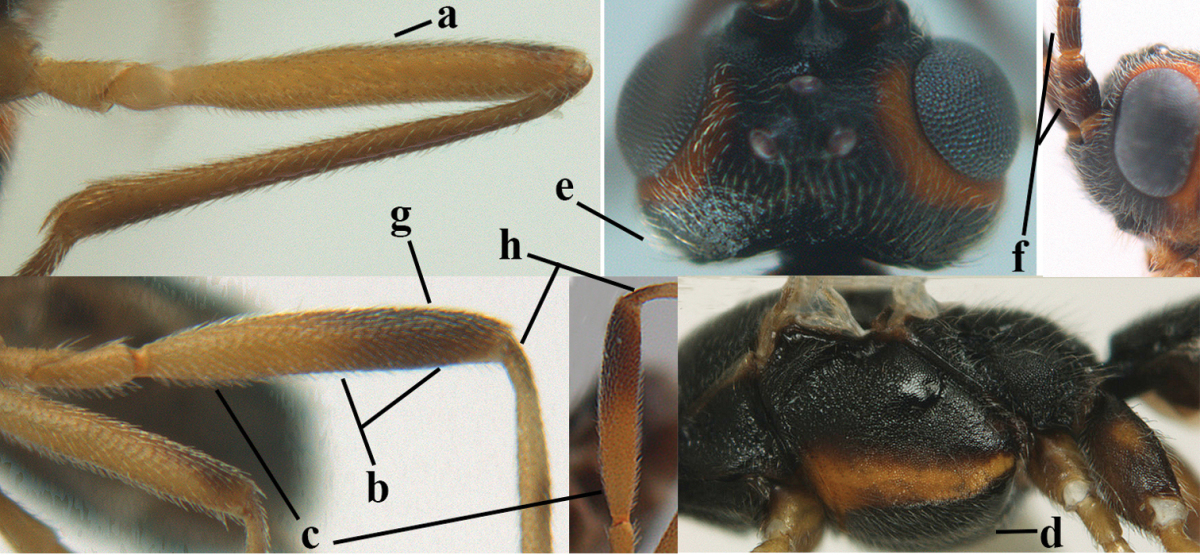	
–	Length of fore femur 5.4–6.4(–8.0) × its maximum width (aa) and hind femur more or less weakly swollen (bb); **or** fore femur more than 6.4 × (aaa) **and** hind femur comparatively wide basally (cc), antenna with more than 40 segments and mesosternum yellowish or orange-brown (dd) or temple slightly wider (ee), or scapus ventrally and basal half of antenna yellowish brown (ff); scapus usually yellowish ventrally, **if** dark brown or blackish then scapus similarly coloured as hind femur subapically; apical half of hind femur (partly) conspicuously dark brown (gg); base of hind tibia yellowish (hh); [face usually completely black or yellowish; POL 1.2–1.7 × diameter of posterior ocellus]	**27**
	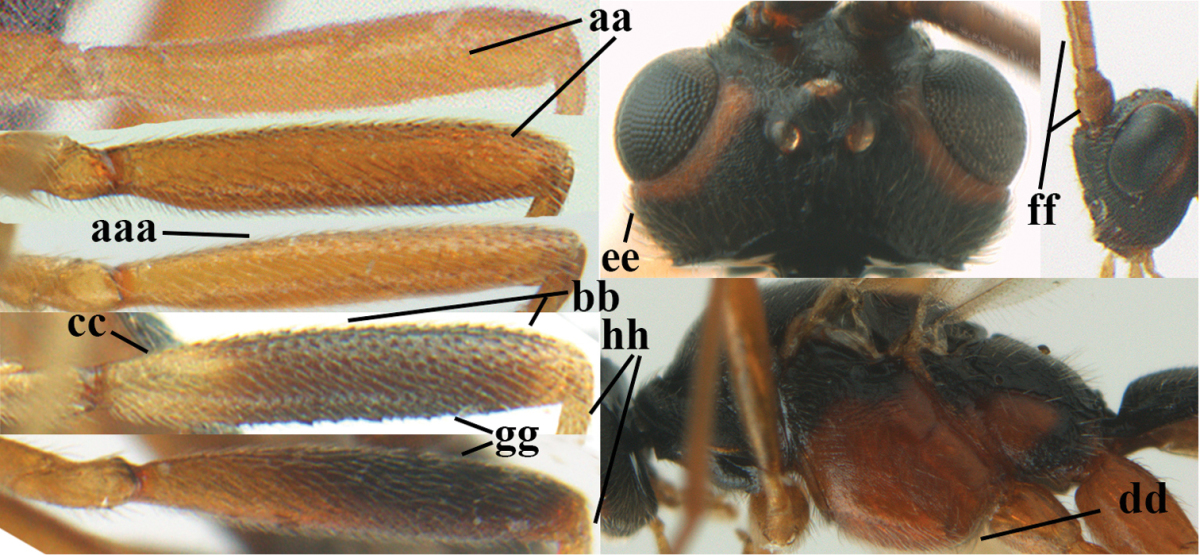	
27	Width of hypoclypeal depression of ♀ 0.35–0.40 × minimum width of face (a); antennal segments of ♀ (38–)39–43, of ♂ (38–)39–44; mesoscutum anteriorly and pronotum medio-anteriorly usually black or dark brown (b); medially mesopleuron usually without distinct rugulae or with a few (c); mesosternum yellowish, orange-brown or reddish (d), **if** darkened then not sharply defined; second metasomal tergite of ♀ with well differentiated median carina (e); lateral margins of third and fourth metasomal tergites of ♂ often completely yellowish, but sometimes darkened; [dark part of hind femur often extended to its apex]	***Aleiodes nigriceps* Wesmael, 1838**
	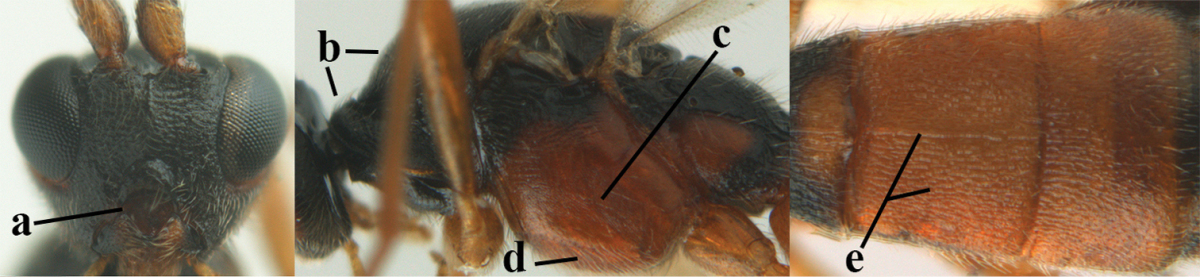	
–	Width of hypoclypeal depression of ♀ 0.30–0.35 × minimum width of face (aa); antennal segments of ♀ (34–)36–40, of ♂ (36–)37–41; mesoscutum anteriorly and pronotum medio-anteriorly often yellowish (bb); medially mesopleuron of females with several rugulae or rugae (cc), but often lacking in males; colour of mesosternum variable, often strongly darkened or black and this usually sharply defined in N. European specimens (dd; more often orange-brown in S. European and Turkish specimens and in some males only indistinctly darkened); second metasomal tergite of ♀ with less differentiated median carina (ee); lateral margins of third and fourth tergites of ♂ almost always dark brown	**28**
	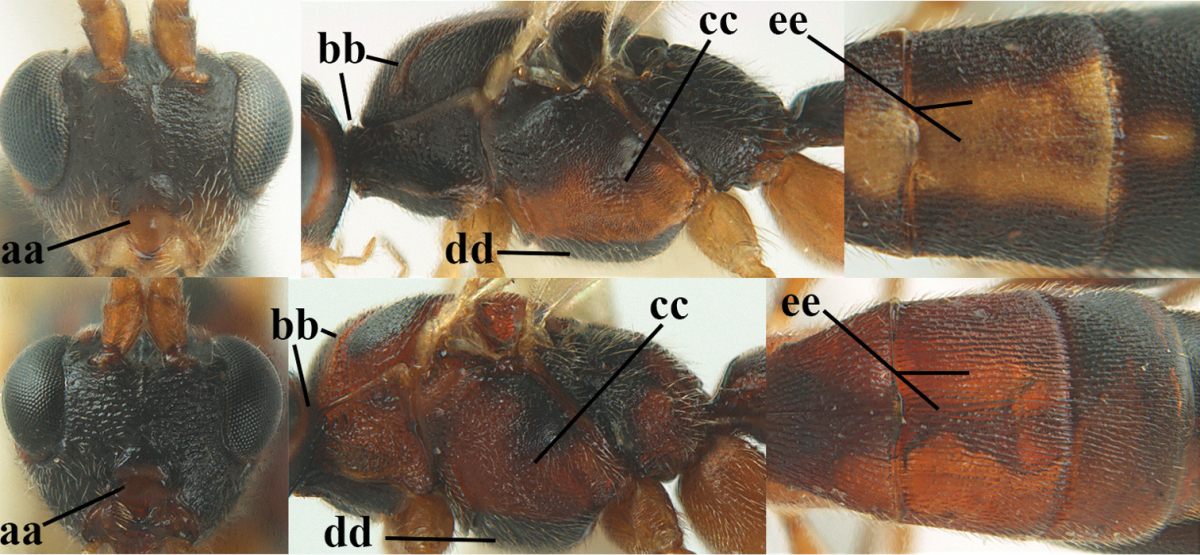	
28	Third and fourth antennal segments of ♀ slenderer (a); ocelli smaller (b); rugosity of face of female less developed (c); palpi slenderer (d); subapical antennal segments of ♀ moderately slender (e); first tergite slenderer (f); propodeum mainly coriaceous and with some rugulae or rugae (g); malar space usually partly or completely and temple near eye yellowish brown (h); fore and hind tarsi slenderer (hind tarsus: i); fore wing subhyaline or somewhat infuscate; mummy slender and light brownish	***Aleiodes pictus* (Herrich-Schäffer, 1838) s.s.**
	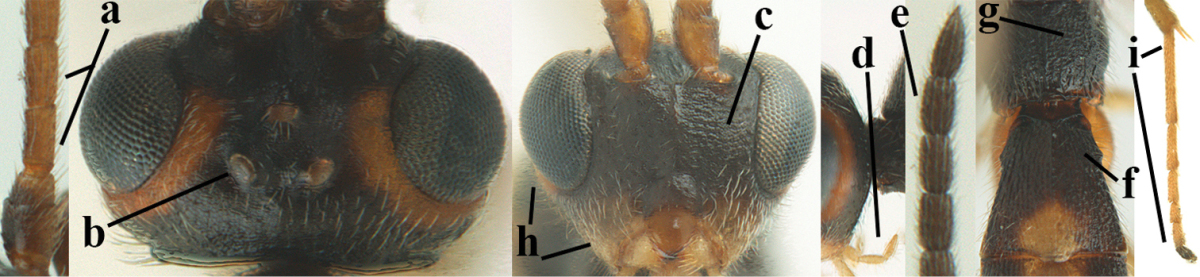	
–	Third and fourth antennal segments of ♀ stout (aa); ocelli somewhat larger (bb); larger part of face of female with distinct rugae (cc); palpi less slender (dd; especially third and fourth maxillary palp segments of ♂ widened); subapical antennal segments of ♀ submoniliform (ee); first tergite robust (ff); propodeum coarsely rugose (gg), but anteriorly less so; malar space and temple near eye usually dark reddish brown or blackish (hh); fore and hind tarsi less slender (hind tarsus: ii); fore wing usually slightly infuscate; mummy swollen and blackish; (unknown of *Aleiodes bistrigatus*)	**29**
	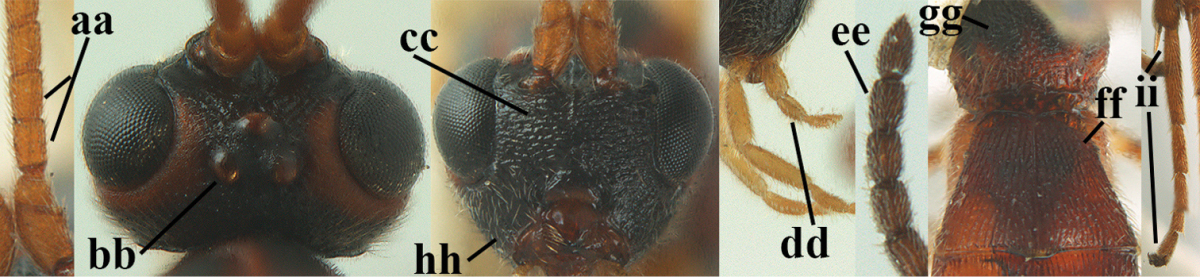	
29	Length of eye in dorsal view about 1.6 × temple (a); 4^th^–7^th^ antennal segments of both sexes slightly slenderer (b); temple behind eye slightly wider (c); number of antennal segments of ♂ usually less than of ♀, about 38 segments	***Aleiodes bistrigatus* Roman, 1917, stat. rev.**
	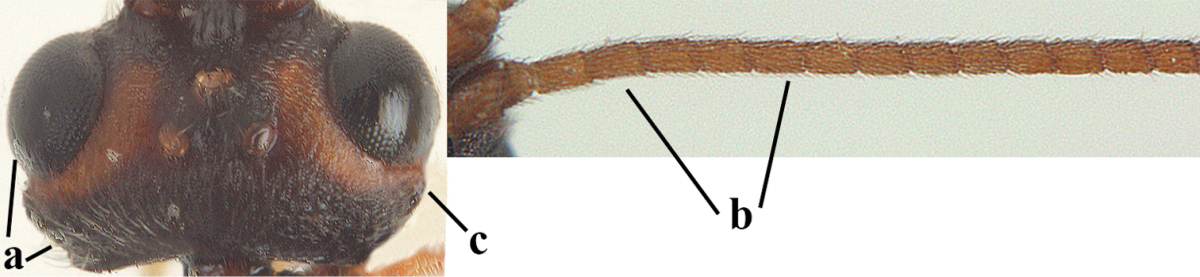	
–	Length of eye in dorsal view 2.2–2.5 × temple (aa); 4^th^–7^th^ antennal segments of both sexes stout (bb); temple behind eyes narrower (cc); number of antennal segments of ♂ usually more than of ♀, 40–45 segments	***Aleiodes diarsianae* sp. n**.
	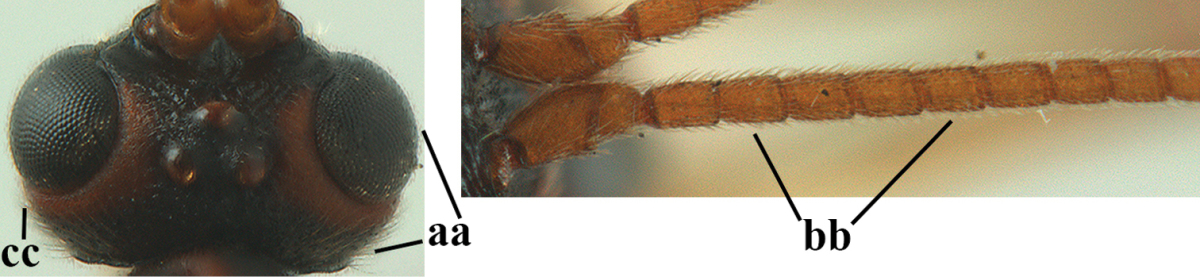	
30	Pterostigma of both sexes blackish or dark brown medially (a), border between dark and pale part well limited, contrasting with each other (b); temples linearly narrowed (c), head trapezoid in anterior view (d), **and** hind femur rather slender (e); OOL about equal to diameter of ocellus or less (f); antennal segments of ♀ 41–45, of ♂ 40–44; [vein 2-SR of fore wing yellowish; propodeum and first tergite usually yellowish in S. England, almost always moderately darkened in N. England and Scotland; vertex may be distinctly rug(ul)ose; mesopleuron shiny and only superficially granulate; rather long face and malar space; stemmaticum of male black and of female usually partly brownish yellow; a predominantly yellowish orange species]	***Aleiodes abraxanae* sp. n.**
	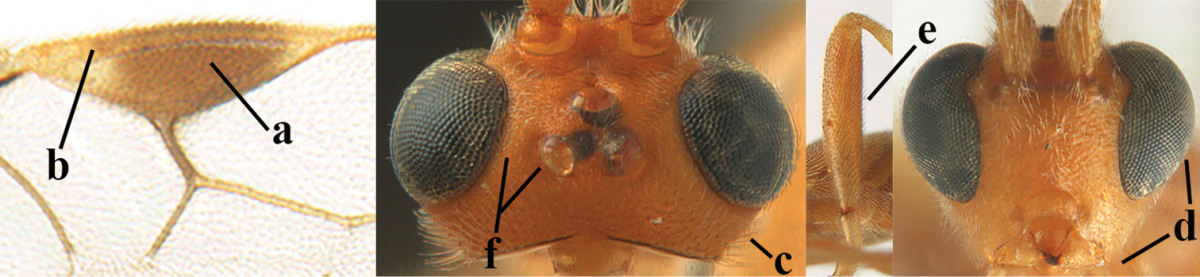	
–	Pterostigma of ♀ often largely or completely yellowish (aa), **if** distinctly infuscate (aaa) then border not well delimited, vague, not or less contrasting with its pale base (bb), antennal segments of ♀ 28–34, temples gradually (roundly) narrowed (cc), hind femur widened (ee) **or** OOL larger than diameter of ocellus (ff) and/or head nearly globular in anterior view (dd); antenna of ♀ often with less than 41 segments; [antennal segments of ♀ 28–47, of ♂ 30–47; mostly largely yellowish or orange species, but some very dark specimens should also run here]	remainder of ***Aleiodes circumscriptus*-group**
	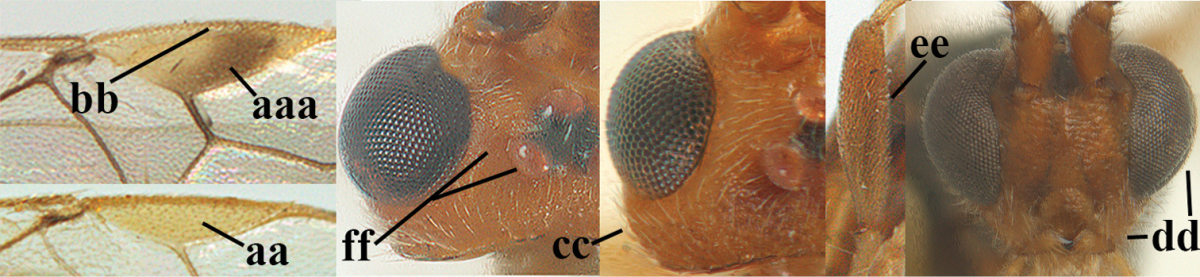	

### Biology and descriptions

#### Aleiodes abraxanae sp. n.

Taxon classificationAnimaliaHymenopteraBraconidae

http://zoobank.org/1BE207D8-E7B5-493F-B103-7F5EE6896569

Figs 7–8 , 9–19

Aleiodes abraxanae van Achterberg in Lozan et al. 2010: 19. Nomen nudum.
Rogas circumscriptus auct. p.p. (not Nees 1834).
Aleiodes armatus auct. p.p. (not Wesmael 1838).

##### Type material.

Holotype, ♀ (NMS, Edinburgh), “[**England**], Otmoor N. R., Oxon., H[ost]: *Abraxas grossulariata* [on] *Prunus spinosa*, HLC [= host larva collected] 13.v.[19]79, PLE [parasitoid larva evident = mummification in the case of *Aleiodes*] 11.vi.[19]79, PIE [= parasitoid imago emerged] 4.vii.[19]79, M.R. Shaw”. Paratypes (74 ♀, 34 ♂): 52 ♀, 25 ♂ reared from larvae of the geometrid *Abraxas grossulariata* (Linnaeus) collected in v/vi, em (v)vi/vii as follows: 14 ♀, 4 ♂ (NMS, RMNH) **England**, Oxford, Otmoor, 1972, 1973, 1979, M.R. Shaw; 2 ♀, 3 ♂ (NMS, BMNH) England, West Sussex, Littlehampton, 1978, 1979, A.A. Allen; 1 ♂ (NMS) England, West Sussex, Hove, 1982, A.R. Cronin; 3 ♀, 1 ♂ (NMS, AAC) England, Surrey, Salfords, 1976, A.A. Allen; 1 ♀, 1 ♂ (NMS, RMNH) England, Berks, Maidenhead Thicket, 1979, M.R. Britton; 4 ♀, 4 ♂ (NMS, RMNH) England, Bucks, Butlers Hangings, 1979, M.R. Shaw; 2 ♀ (NMS) England, Bucks, Milton Keynes, 1984, J.P. Brock; 1 ♀ (BMNH) England, Cambridge, 1913, L. Doncaster; 2 ♀ (NMS) England, Westmorland, Beetham, 1991, M.R. Shaw; 11 ♀, 1 ♂ (NMS, BMNH) **Scotland**, Fife, St Andrews, 1935, 1936, 1938, D.J. Jackson; 1 ♀ (NMS) Scotland, Stirling, D.J. Jackson; 8 ♀, 7 ♂ (NMS, RMNH) Scotland, Orkney, Mainland, Waulkmill Bay, 2009, K.P. Bland; 1 ♂ (NMS) Scotland, Orkney, Mainland, Caldale Bottom, 2009, K.P. Bland; 2 ♀ (NMS) Scotland, Orkney, Mainland, Redland, 2009, K.P. Bland; 1 ♂ (NMS) Scotland, Orkney, Hoy, Nowt Bield, 2009, K.P. Bland; 1 ♂ (NMS) Scotland, Orkney, Hoy, Enegars, 2004, S. Gauld; 2 ♀ (SDEI) **Germany**, Sachsen-Anhalt, Wolfen, 1957, B. Stehlik; additionally 1 ♀ (NMS) from the Otmoor locality, host larva collected 8.x.1978, mummified 4.vi.1979, emerged 3.vii.1979, M.R. Shaw. Non-reared specimens: 1 ♂ (NMS) **England**, Cambridge, Chippenham Fen, 9.vii.1983, M.R. Shaw; 1 ♀ (NMS) England, Hunts, Monks Wood, 31.viii.2005, G.R. Broad; 1 ♂ (NMS) England, East Gloucester, Eastleach, 8.viii.2006, M.R. Shaw; 1 ♀ (BMNH) East Cornwall, Botusfleming, Marshall collection; 4 ♀ (BMNH) England, Oxford, Stanton St. John, 19.viii. 1968 (1) and 4.ix.1968 (3), J.P. Brock; 1 ♂ (BMNH) England, Herts, Whetstone, 24.vii.1961, P.H. Ward; 1 ♀, 1 ♂ (BMNH) England, Northampton, Spratton, x.1975 and vii.1976 respectively, I. & P. Gauld; 2 ♀ (BMNH) British Isles, Harwood coll.; 1 ♀ (BMNH) presumed British, A. Matthews in Lyle coll.; 3 ♀ (BMNH) presumed British, Stephens coll.; 1 ♀ (CMIM) England, Dorset, Weymouth 24.vi.1899, Peachell; 1 ♀ (CMIM) England, West Suffolk, Old Newton; 1 ♂ (CMIM) England, East Suffolk, Monk’s Soham, 18.vii.1933; 2 ♀, 1 ♂ (AAC) England, South Devon, Shaldon, 7.viii.1978 (1 ♀) and 6.viii.1979 (1 ♀, 1 ♂), A.A. Allen; 2 ♀ (NMS, RMNH) **Wales**, Anglesey, Llangristiolus, 27.viii–25.ix.1982, S.A. & D.C. Wilkinson; 1 ♀ (NMS) **Scotland**, West Ross, Sheildaig, viii. 1991, I. MacGowan; 2 ♂ (NMS) Scotland, South Uist, Loch Eynort, vi.1988, D. Whiteley; 2 ♀ (ALC, RMNH) **Czech Republic**, South Bohemia, Šumava Boubinský Pralés, virgin forest, 1000–1300 m, light trap, 22–24.vii.2003, I. Jaroš & K. Spitzer; 1 ♀ (ALC), Czech Republic, South Bohemia, Šumava Mts, 740 m, peat bogs near Mrtvý, light trap, 21–24.viii.2001, I. Jaroš & K. Spitzer; 1 ♂ (NMS) **Finland**, Satakunta, Pori, 1991, K. Ruohomäki; 1 ♀ (NMS) **Sweden**, Gotland, Sundre, Barrshage, vii/viii.2004, N. Ryrholm.

##### Molecular data.

MRS391 (Sweden JF962827, CO1), MRS636 (Scotland HQ551278/HQ551264, CO1), MRS637 (Scotland HQ551262, CO1 + KU682263, 28S), MRS694 (Scotland HQ551277, CO1).

##### Biology.

Univoltine and presumed monophagous parasitoid of *Abraxas grossulariata*, overwintering in the living host larva and killing it in early summer in its penultimate instar. Mummy (Fig. 8) black and moderately swollen. In Britain very widespread and present in most well-established colonies of the host, on its various foodplants (rearing records from *Prunus spinosa*, *Ribes uva-crispa*, *Ribes nigrum*, *Calluna vulgaris* and *Euonymus japonicus*). It has not been reared from collections of the congeneric *Abraxas sylvata* (Scopoli) which hibernates as a pupa and would not provide a means for the parasitoid to overwinter. The essentially univoltine host overwinters as a small caterpillar, within which the parasitoid overwinters as an early (probably first) instar larva. The mummies of penultimate instar hosts are made in exposed situations on very narrow stems etc. in about early June and, no doubt aided by their almost black and hence heat-absorbing colour, adult emergence follows quickly; the mummies otherwise being subject to high levels of pseudohyperparasitism. The adult females are unwilling to mate soon after their emergence in the morning but become highly receptive in early evening, offering themselves to males at the first contact which sometimes happens before the males are fully aware of them, and repeated copulation bouts with the same or another male frequently ensue over the next tens of minutes. The new generation of hosts is not available for several weeks, but the adult females are long-lived (confirmed in captivity), and have been collected from June well into the autumn (as late as October). The host has been in severe decline in Britain in recent years, apart from its strong presence on *Calluna* in parts of Scotland, which remain also a stronghold for the otherwise declining parasitoid. When surveying for the presence of the parasitoid, undersized hosts collected in mid to late May are the most likely to yield results.

**Figures 7–8.**
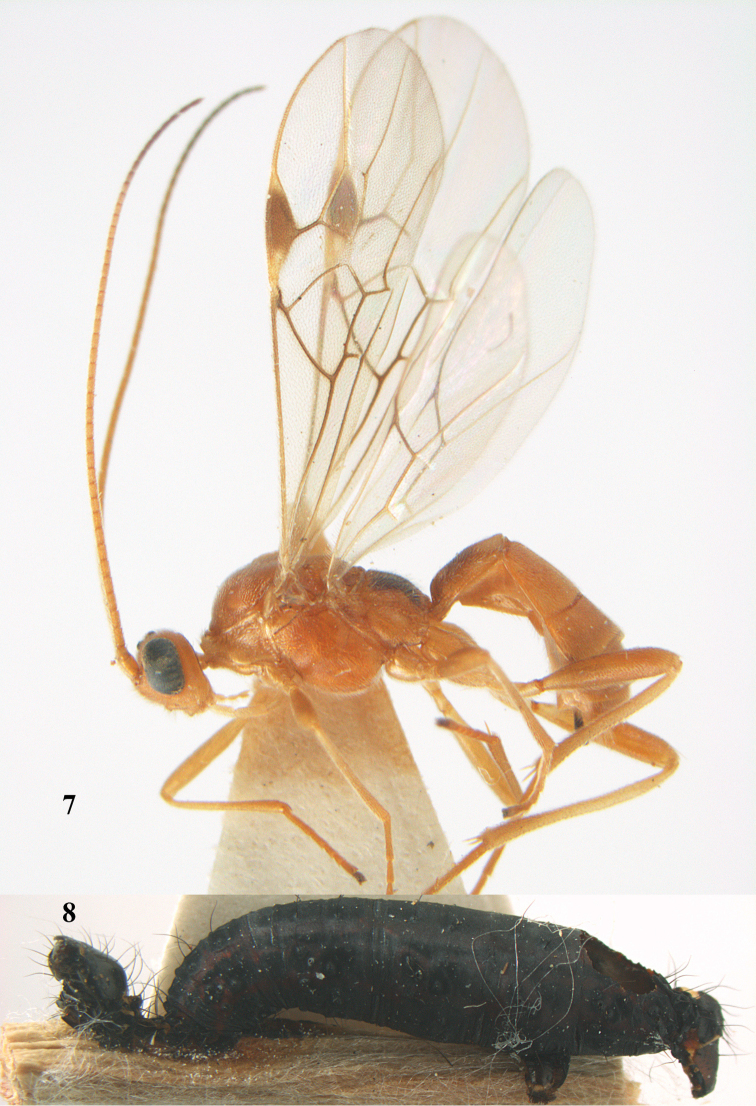
*Aleiodes abraxanae* sp. n., ♀, holotype. **7** habitus lateral **8** mummy of *Abraxas grossulariata* (Linnaeus).

##### Diagnosis.

**Figures 9–19.**
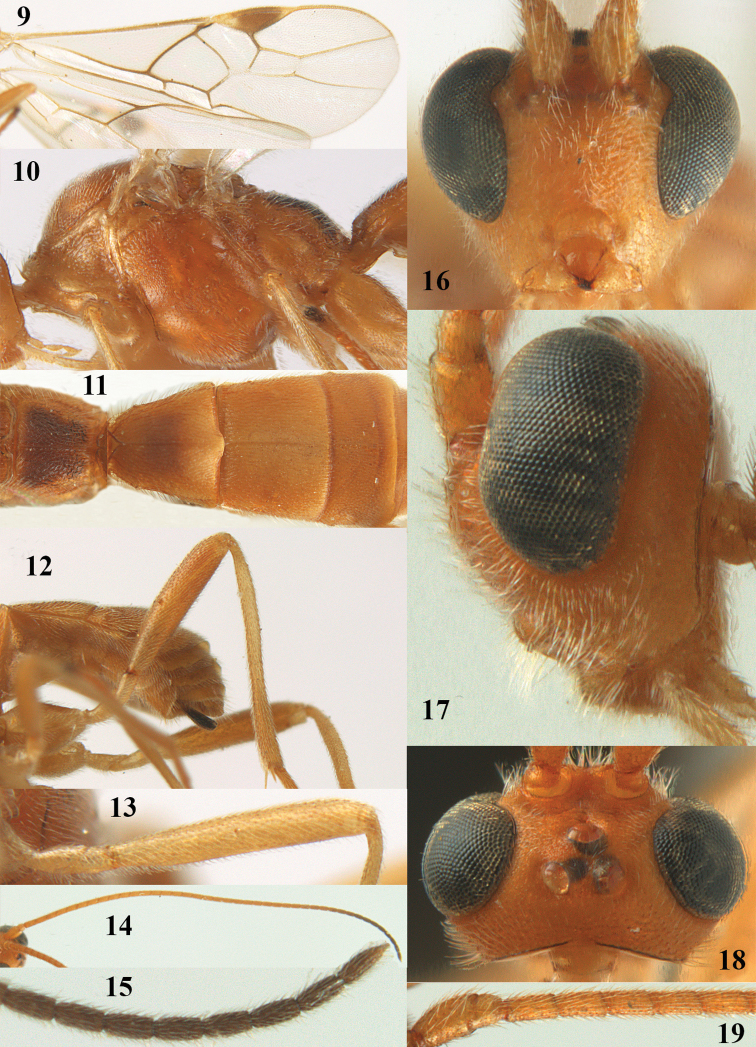
*Aleiodes abraxanae* sp. n., ♀, holotype. **9** wings **10** mesosoma lateral **11** propodeum and anterior half of metasoma dorsal **12** hind leg lateral **13** fore femur lateral **14** antenna **15** apical segments of antenna **16** head anterior **17** head lateral **18** head dorsal **19** basal segments of antenna.

Pterostigma of both sexes blackish or dark brown medially, border between dark and pale part sharp, contrasting with each other (Figs 7, 9); temples directly strongly narrowed (Fig. 18) and comparatively wide in lateral view (Fig. 17); OOL about equal to diameter of ocellus; propodeum and first tergite yellowish or medially brown (Fig. 11); vein 2-SR of fore wing yellowish as vein 1-R1 (Fig. 9); head moderately transverse (Fig. 18); antennal segments of ♀ 41–45, of ♂ 40–44; subapical antennal segments slender (Fig. 15); vertex may be distinctly rug(ul)ose and mesopleuron only coriaceous medially (Fig. 10); body entirely brownish yellow, at most propodeum and first tergite medially brown. Similar to *Aleiodes hellenicus* Papp, 1985, but *Aleiodes hellenicus* has pterostigma of both sexes completely pale yellowish and precoxal area usually with rugae medially.

##### Description.

Holotype, ♀, length of fore wing 5.1 mm, of body 6.2 mm.

*Head.* Antennal segments of ♀ 43, length of antenna 1.3 × fore wing, its subapical segments about 2.3 × as long as wide; frons only coriaceous, matt; OOL 0.9 × diameter of posterior ocellus and coriaceous; vertex coriaceous, matt; clypeus convex, coriaceous; ventral margin of clypeus thick and depressed (Fig. 16); width of hypoclypeal depression 0.4 × minimum width of face (Fig. 16) and face coriaceous; length of eye 3.8 × temple in dorsal view and temple directly narrowed behind eye; occiput behind stemmaticum coriaceous and occipital carina interrupted by somewhat less than width of ocellus (Fig. 18); clypeus partly above lower level of eyes (Fig. 16); length of malar space 0.3 × height of eye in lateral view; eyes distinctly protruding (Figs 16, 18).

*Mesosoma.* Mesoscutal lobes largely coriaceous, matt, but medio-posteriorly longitudinally rugose; notauli narrow and smooth, posteriorly lost in rugose area; prepectal carina medium-sized, reaching anterior border; precoxal area of mesopleuron and metapleuron coriaceous, matt; mesopleuron above precoxal area (except smooth and shiny speculum) coriaceous, but dorsally rugose; mesosternal sulcus narrow and shallow, impressed and without carina posteriorly; mesosternum angulate posteriorly; scutellum slightly convex, coriaceous, and carinate laterally; propodeum evenly convex and rugose but anteriorly weakly so, median carina complete, without tubercles.

*Wings.* Fore wing: r 0.4 × 3-SR (Fig. 9); 1-CU1 horizontal, 0.35 × as long as 2-CU1; r-m 0.7 × 2-SR, and 0.5 × 3-SR; second submarginal cell medium-sized (Figs 7, 9); cu-a vertical, not parallel with CU1b, straight (Fig. 9); 1-M slightly curved posteriorly. Hind wing: apical half of marginal cell parallel-sided; 2-SC+R short and longitudinal; m-cu present but unpigmented.

*Legs.* Tarsal claws setose; hind coxa coriaceous, largely matt; hind trochantellus 2.6 × longer than wide; length of fore and hind femora 6.1 and 4.9 × their width, respectively (Figs 12–13); inner apex of hind tibia without comb; length of inner hind spur 0.25 × hind basitarsus.

*Metasoma.* First tergite nearly as long as wide posteriorly, moderately convex and latero-posteriorly lamelliform; first and second tergites densely and finely longitudinally rugose, robust (Fig. 11), with distinct median carina; medio-basal area of second tergite obsolescent; second suture shallow and crenulate; basal half of third tergite finely rugose, remainder of metasoma largely superficially coriaceous; fourth and apical third of third tergite without sharp lateral crease; ovipositor sheath densely setose.

*Colour.* Brownish yellow; apical fifth of antenna and dorsally propodeum dark brown; ovipositor sheath black; palpi, tegulae, apical 0.4 of first tergite and more or less second tergite pale yellowish; veins (but distally from 2-SR yellowish) and pterostigma (except yellow base and apex) dark brown; border between dark and pale part of pterostigma sharp, contrasting with each other (Figs 7, 9); wing membrane subhyaline.

*Variation.* Length of fore wing 4.4–5.3 mm; antennal segments of ♀ 41(10), 42(18), 43(30), 44(6), and 45(1), of ♂ 40(3), 41(7), 42(8), 43(4), 44(4); stemmaticum of male black and of female brownish yellow; basal 0.2–0.5 of pterostigma pale yellow, rarely largely yellow and only medially darkened; first tergite yellowish or infuscate medially.

##### Etymology.

Named after the generic name of its host: *Abraxas* Leach.

##### Distribution.

*British Isles (England, Scotland, Wales), *Czech Republic, *Finland, *Germany, *Sweden.

##### Note.

The males of this species have on average about one antennal segment less than females.

#### Aleiodes albitibia

Taxon classificationAnimaliaHymenopteraBraconidae

(Herrich-Schäffer, 1838)

Figs 20–21 , 22–34

Rogas albitibia Herrich-Schäffer, [April] 1838: 156; Shenefelt 1975: 1217; van Achterberg 1991: 24 (as senior synonym of Aleiodes heterogaster).
Aleiodes albitibia ; van Achterberg 1991: 24; Belokobylskij et al. 2003: 398.
Aleiodes heterogaster Wesmael, [May] 1838: 96; Shenefelt 1975: 1176; Papp 1991: 97 (examined).
Rhogas heterogaster ; Fahringer 1932: 258–259.
Rogas heterogaster ; Hammond and Smith 1957: 181; Tobias 1986: 82 (transl.: 136).

##### Type material.

Redescribed ♀ and holotype of *Aleiodes heterogaster* (KBIN), “[**Belgium**], Campine, 1833”, “*Aleiodes heterogaster* mihi, det. C. Wesmael”, “Belgique, Charleroi/teste Papp, J., 1983”, “Holotypus *Aleiodes heterogaster* Wesm., 1838 / Papp, 1983”. The type series of *Rogas albitibia* is lost.

##### Additional material.

*Austria, **British Isles** (**England**: V.C.s 3, 11, 15, 22, 58, 59, 61, 69; **Wales**: V.C. 49; **Scotland**: V.C.s 72, 77, 80, 88, 92, 96, 97, 98, 99; **Ireland**: (V.C.s H1, H19, H20), *Czech Republic, **Finland**, **France**, **Germany**, **Hungary**, **Netherlands** (DR: Wijster, LI: Asselt; Castelre, GE: Heerde; Putten; Tongeren, NB: Tilburg (Kaaistoep), NH: Muiderberg, OV: Buurse (Schipbeek)), **Poland**, *Spain, **Sweden**, **Switzerland**. Specimens in NMS, BMNH, BZL, CNC, OUM, RMNH, SDEI, USNM, ZSSM, I. Kakko collection, and WUIM.

##### Molecular data.

MRS210 (Scotland EU979574, CO1), MRS383 (Sweden JF962835/ KU682238, CO1), MRS753 (Sweden KU682248, CO1).

##### Biology.

Univoltine parasitoid of arboreal notodontids, overwintering in a highly distinctive mummy (Fig. 21). Specimens (in NMS unless indicated) reared from wild collected arboreal Notodontidae identified as *Notodonta dromedarius* (Linnaeus) (12 [5 are OUM, 2 are USNM]; T.H. Ford, M.J. Morgan, M.R. Shaw, A.W. Stelfox, W.A. Watson), *Eligmodonta ziczac* (Linneaus) (2:1; M.R. Shaw), *Pheosia tremula* (Clerck) (8 [1 is AAC]; A.A. Allen, B.T. Parsons, M.R. Shaw), *Pheosia gnoma* (Fabricius) (2 [BMNH]; G. Graham-Smith). Host range experiments had the following outcomes: *Eligmodonta ziczac* 2:32\10\\8+2; *Pterostoma palpina* (Clerck) 2:10\0\\-; *Ptilodon capucina* (Linnaeus) 1:5\0\\-; *Clostera pigra* (Hufnagel) 2:9\0\\-; *Phalera bucephala* (Linnaeus) 2:2\0\\-. The developmental biology of this species is rather unusual in several respects. On approaching the host (*Eligmodonta ziczac* in all the following observations, which are based on two female *Aleiodes albitibia*) the female seems to depend on its fore and middle tarsi more than its antennae for host assessment, although antennation does occur (possibly as much to desensitise the host as to investigate it). The host is not paralysed during attack: the female more or less pounces on the host once accepted and aligns herself along the host’s body, which she grasps with her front and middle legs during oviposition, and fans her wings for short bursts repeatedly during the oviposition process (this may have indicated that venom was being injected, but if so it did not appear to have a significantly paralysing action), which in some cases lasted as long as 10–15 minutes – in these cases usually with more than one insertion. In fact, probably because the host was not temporarily parasitised and so was not sluggish, self-superparasitism happened very easily. The metasoma is only weakly curled downwards during oviposition, after which the female left the host without the usual post-oviposition period of association. When young, the females host-fed non-destructively on occasion. Development of the parasitoid larva was extremely rapid at ca 22–25 °C, with mummification ensuing after as few as 7–10 days from oviposition. The unusual and highly distinctive mummy (Fig. 21) is very shiny and dark mahogany brown in colour. It consists of the caudal portion of the host (usually from abdominal segment 3 onwards), strongly delimited from the anterior portion which usually shrivels up and becomes detached, leaving a sharp rim. The parasitoid pupates in a capsule which is formed in the most caudal half of this structure (usually in abdominal segments 5–8), dorsally raised, thinly lined with silk, and sealed from a more ventral and anterior inflation of the host’s cuticle that is of approximately equal volume but fully open at its anterior end. During the mummification process this area fills with liquid and bubbles, but fluids do not spread to the substrate and the mummy does not become stuck down. Once dried and hardened, the whole structure looks as though it contains two pupating parasitoids, and indeed some authors have been misled by this (Hammond and Smith 1957). It is possible that the empty chamber may serve to decoy pseudo-hyperparasitoids, though this seems unlikely to be its main function. The mummy forms on the host’s food plant, usually on a leaf surface from which it is easily dislodged, and overwinters in the leaf litter. *Aleiodes albitibia* occurs particularly in wet, bushy places: bearing in mind that its hosts (evidently rather restricted within Notodontidae) feed on trees and shrubs (*Salix*, *Populus*, *Alnus* and *Betula*) that often overhang water, the form of the mummy may also be an adaptation to prevent submersion and perhaps also results in dispersion by water, as it floats easily and is not wetted. The winter is passed in the mummy, and *Aleiodes albitibia* is univoltine, with a flight period in Britain of roughly JuneAugust.

**Figures 20–21.**
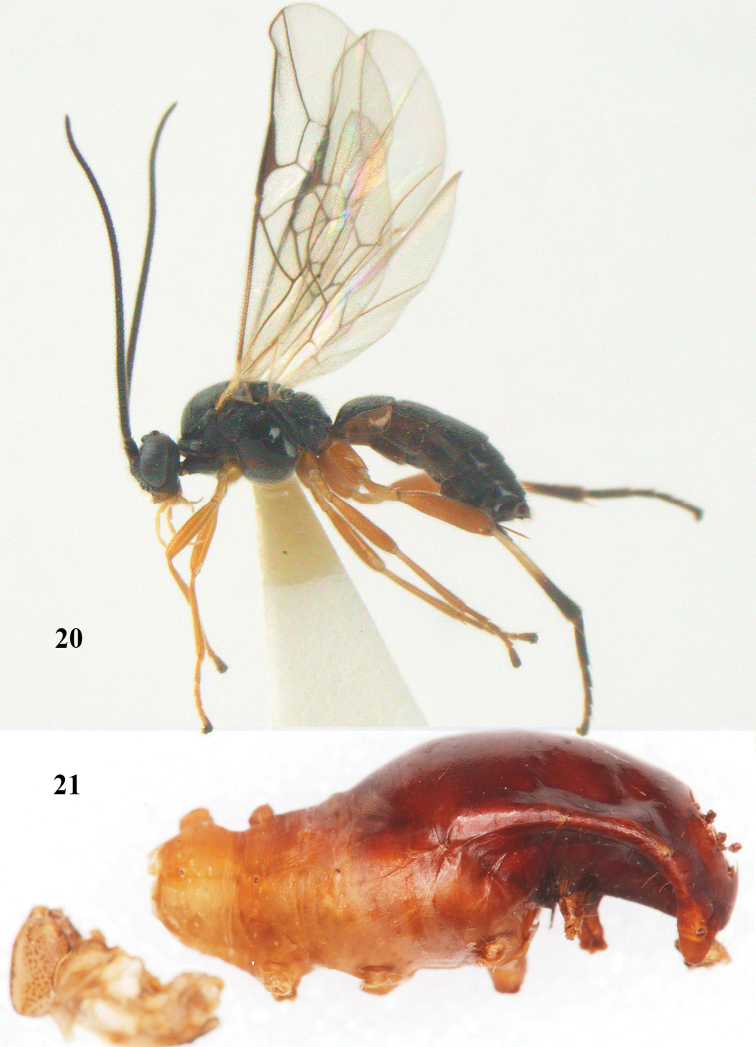
*Aleiodes albitibia* (Herrich-Schäffer), ♀, England. **20** habitus lateral **21** mummy of *Notodonta dromedarius* (Linnaeus).

##### Diagnosis.

Third antennal segment robust (Fig. 34); area in front of anterior ocellus without tubercle; OOL 0.4–0.5 × diameter of posterior ocellus (Fig. 32); mesopleuron strongly shiny and precoxal area not impressed (Fig. 24); pterostigma dark brown; vein 1r-m of hind wing about as long as vein 1-M (Fig. 23); inner hind tibial spur 0.4–0.5 × hind basitarsus; inner side of basal half of hind tibia whitish, rarely largely dark brown; inner side of hind tibia with bristly setae and no comb apically; third tergite curved medio-posteriorly in dorsal view (Fig. 25) and longer than second; metasomal tergites largely black (also laterally so), sometimes with a large yellow or ivory central patch on second tergite.

**Figures 22–34.**
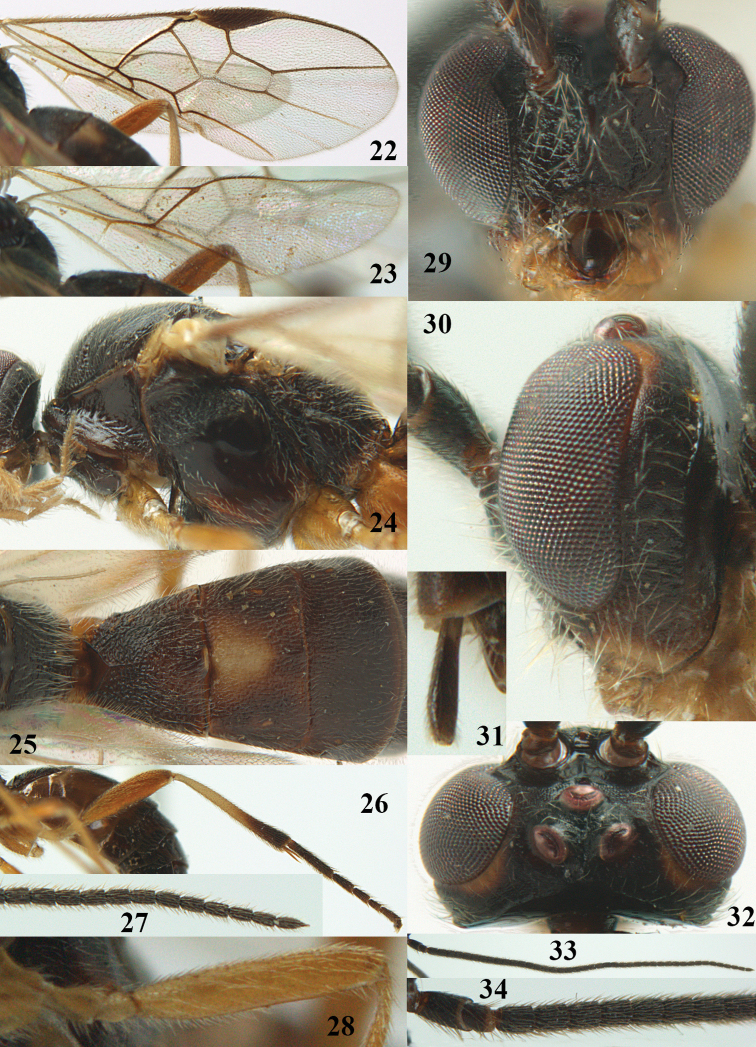
*Aleiodes albitibia* (Herrich-Schäffer), ♀, Switzerland. **22** fore wing **23** hind wing **24** mesosoma lateral **25** propodeum and anterior half of metasoma dorsal **26** hind leg lateral **27** apical segments of antenna **28** fore femur lateral **29** head anterior **30** head lateral **31** ovipositor sheath lateral **32** head dorsal **33** antenna **34** basal segments of antenna.

##### Description.

Holotype of *Aleiodes heterogaster*, length of fore wing 5.4 mm, of body 5.6 mm.

*Head*. Antennal segments of ♀ 46, long setose, length of antenna 1.2 × fore wing, its subapical segments distinctly longer than wide; frons weakly depressed, finely rugose medially, remainder superficially micro-granulate; OOL 0.4 × diameter of posterior ocellus, and finely granulate; vertex finely granulate, with some rugulae posteriorly, rather dull; clypeus normal, micro-granulate; ventral margin of clypeus thick and not protruding forwards; width of hypoclypeal depression 0.5 × minimum width of face (Fig. 29); length of eye 2.8 × temple in dorsal view (Fig. 32); occiput behind stemmaticum granulate with some rugulae, narrow; clypeus near lower level of eyes; length of malar space 0.2 × length of eye in lateral view; occipital carina widely interrupted medio-dorsally and ventrally (Fig. 30).

*Mesosoma*. Mesoscutal lobes largely granulate and with punctulation, matt; prepectal carina complete, rather weak; precoxal area of mesopleuron largely smooth (but in other specimens usually micro-granulate with some punctulation); mesopleuron above precoxal area strongly shiny and smooth; metapleuron largely coriaceous; scutellum granulate and finely punctate, no distinct carina; propodeum evenly convex, short, anteriorly granulate, medially and posteriorly rugose, median carina complete, without tubercles.

*Wings*. Fore wing: r 0.5 × 3-SR (Fig. 22); 1-CU1 horizontal, 0.35 × 2-CU1; r-m 0.5 × 3-SR; second submarginal cell rather long (Fig. 22); cu-a inclivous, curved posteriorly; 1-M straight posteriorly. Hind wing: marginal cell subparallel-sided, its apical width 1.0 × width at level of hamuli (Fig. 23); 2-SC+R shortly longitudinal (but in other specimens subquadrate); m-cu absent; M+CU:1-M = 5:3; 1r-m about as long as 1-M.

*Legs*. Tarsal claws yellowish setose; hind coxa sparsely punctulate, and granulate; hind trochantellus robust; length of fore femur, hind femur and basitarsus 5.4, 3.7 and 6.6 × their width, respectively (Figs 26, 28); length of inner hind spur 0.5 × hind basitarsus, as long as outer spur.

*Metasoma*. First tergite robust (Fig. 25); first and second tergites rather coarsely longitudinally (reticulate-)rugose, robust, with distinct median carina; medio-basal area of second tergite minute, triangular; second suture shallow; third tergite as long as second tergite and largely granulate, anteriorly with some rugulae; remainder of metasoma smooth, compressed; fourth and apical half of third tergite without sharp lateral crease; ovipositor sheath slender and rather shiny.

*Colour*. Black; malar area, narrow stripe along eyes dorsally, fore and middle legs (but telotarsi infuscate), hind coxa, trochanter trochantellus and femur, palpi and tegulae yellowish; pterostigma and most veins dark brown; basal 0.6 of hind tibia ivory; remainder of hind tibia and tarsus blackish.

*Variation*. Antennal segments of ♀: 43(8), 44(14), 45(14), 46(3), 47(3), 49(1); of ♂: 39(1), 40(7), 41(7), 42(4), 43(3), 44(2). Second metasomal tergite may be largely yellowish or ivory (except laterally), or only with pale basal patch; hind tibia may be largely dark brown; base of pterostigma and first tergite medio-apically completely black (typical *Aleiodes heterogaster*) or yellowish (typical *Aleiodes albitibia*); width of hypoclypeal depression 0.4–0.5 × minimum width of face; mesopleuron usually with faint brownish longitudinal streak ventrally.

##### Notes.

Males average about four fewer antennal segments than females. As is the case for the vast majority of *Aleiodes* species, only one parasitoid develops in each host (*pace* Hammond and Smith 1957).

#### Aleiodes angustipterus sp. n.

Taxon classificationAnimaliaHymenopteraBraconidae

http://zoobank.org/7186C4F3-1A0F-4128-870D-25B7E20DE304

Figs 35 , 36–47

##### Type material.

Holotype, ♀ (RMNH, Leiden), “**Nederland** (Dr.), Wijster, opposite Biol. Station, 12–19.viii.1977, C. v. Achterberg”. Paratypes (15 ♀): 1 ♀ (NMS), **England**, Cumbria, Whitbarrow, Howe, MV light, 24.viii.1995, M.R. Shaw; 1 ♀ (NMS), England, Norfolk, Scarning, TF981120, 6.vii–1.ix.1988, A.P. Foster/NCC; 1 ♀ (NMS), England, Norfolk, Sutton, TQ373235, water trap, 21.viii–4.ix.1986, A.P. Foster/NCC; 2 ♀ (NMS), **Wales**, Anglesey, Fedw Fawr, SH6081, MV light, 11.viii.2003, M.R. Shaw; 1 ♀ (NMS), Wales, Gwent, Magor Marsh, ST425865, water trap 8–21.vii.1988, P. Holmes/NCC; 1 ♀ (Tullie House Museum, Carlisle), **Scotland**, Dumfriesshire, Gretna, Springfield, 17.viii.1939, J. Murray; 1 ♀ (BMNH), **Jersey**, Trinity, Howard Davis Farm, Rothamsted trap 18.iv–3.xii.2004, A. Vautier/P. Gould; 1 ♀ (FMNH), **Finland**, U. Vantaa. 6690:384. ex larva *Hypenodes humidalis* 27.v.1974, cocoon [in which the mummy formed?] 19.vi.1974, em. 3.vii.1974, E.O. Peltonen; 1 ♀ (BZL), **Greece**, Thráki NW, Mt. Menikio, 12.viii.2010, J. Halada; 1 ♀ (NMS), **Lithuania**, Cerkelia peat bog, 3.ix.2006, A. Lozan; 1 ♀ (M. Riedel Collection), **Russia**, E. Siberia, 10 km E Irkutsk, 8.viii.2005, Berlov; 1 ♀ (NMS), **Portugal**, Azores, ca 2008 [per D.L.J. Quicke, no further data]; 1 ♀ (MRS), **China**, Yangtze River near Fengdu, 15.vii.2002, M.R. Shaw; 1 ♀ (RMNH), **Japan**, Kusakai, Kawai V., Iwate, 3–4.viii.1981, A. Takasu.

##### Molecular data.

MRS172 (China KU682231, CO1), MRS279 (Wales KU682232, CO1), MRS280 (Wales KU682233, CO1), MRS822 (Azores KU682246, CO1).

##### Biology.

No males have been seen, suggesting that this species might be thelytokous. Only a single reared specimen examined, from *Hypenodes humidalis* Doubleday (Erebidae: Hypenodinae). From the specimen labelling, the mummy appears to be formed in the host cocoon (but this has not been examined) and the adult emerged the same year. From this, and the flight data (vii-viii), it is surmised that it is a univoltine species, overwintering in the partly fed host larva. *Hypenodes humidalis* occurs in both acidic and alkali marshy areas, and the larva feeds on plant debris certainly including dead or dying *Molinia caerulea* (G.M. Haggett, personal communication). Indeed, when known the collecting sites of *Aleiodes angustipterus* have mostly been wet grasslands, including fens and bogs, but at least one specimen was collected in woodland on a limestone hill (Whitbarrow) which may suggest a wider host range.

##### Diagnosis.

**Figure 35.**
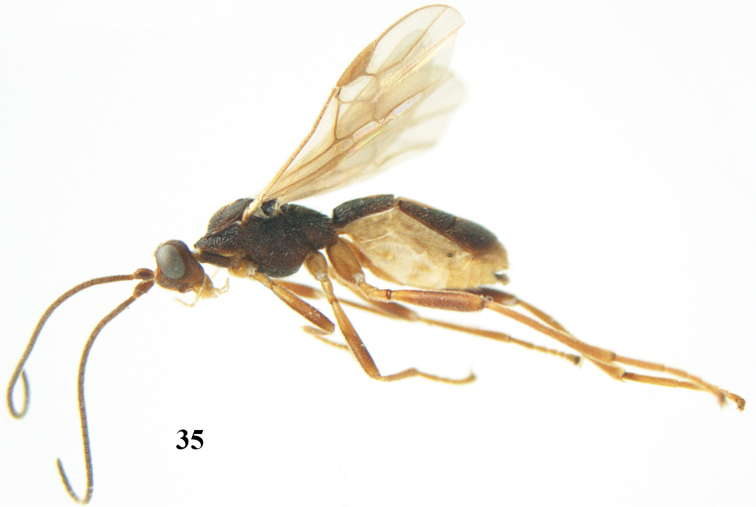
*Aleiodes angustipterus* sp. n., ♀, holotype, habitus lateral.

**Figures 36–47.**
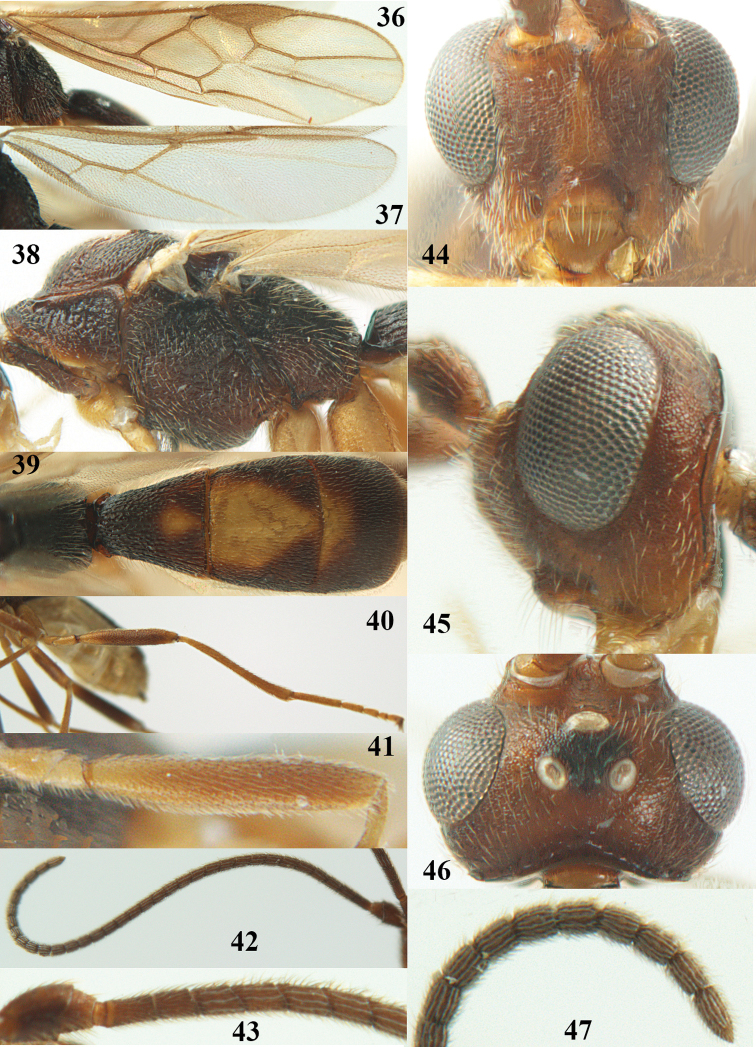
*Aleiodes angustipterus* sp. n., ♀, holotype. **36** fore wing **37** hind wing **38** mesosoma lateral **39** propodeum and anterior half of metasoma dorsal **40** hind leg lateral **41** fore femur lateral **42** antenna **43** basal segments of antenna **44** head anterior **45** head lateral **46** head dorsal **47** apical segments of antenna.

Head subglobular (Fig. 46) and body slender; antenna of ♀ without a pale submedial band; antennal segments of ♀ 36–40; eye rather small (Fig. 45); OOL 1.2 × posteior ocellus; speculum of mesopleuron rugose or reticulate and dull as is remainder of mesopleuron (Fig. 38); propodeum slightly elongate (Fig. 38); fore wing narrow (Fig. 36); pterostigma brown; hind coxa distinctly shorter than first tergite; hind femur 6–7 × as long as its maximum width; hind trochantellus slender (Fig. 40); dorsal carinae of first metasomal tergite lamelliform protruding basally; second tergite with small smooth triangular area medio-basally and tergite rather short (Fig. 39); third tergite weakly sculptured; fourth tergite partly or entirely without sharp lateral crease, fourth and following tergites partly retracted and largely smooth. Morphologically similar to *Aleiodes jakowlewi* from Finland, Sweden and N. Russia, but *Aleiodes jakowlewi* has the hind coxa about as long as first tergite and second tergite comparatively long (hind coxa distinctly shorter than first tergite in *Aleiodes angustipterus* (Fig. 35) and second tergite comparatively short (Figs 35, 39)); fourth tergite with distinct sharp lateral crease and basally rugulose (fourth tergite partly without distinct sharp lateral crease, partly retracted and largely smooth); third tergite strongly sculptured (third tergite weakly sculptured); pterostigma dark brown with basal third pale (pterostigma dark brown); eye comparatively large (eye comparatively small); antenna of ♀ sometimes with a narrow white or pale yellowish submedial band (antenna of ♀ without a pale submedial band); antennal segments of female 49–52 (36–40).

##### Description.

Holotype, ♀, length of fore wing 3.2 mm, of body 4.1 mm.

*Head.* Antennal segments of ♀ 36, length of antenna 1.1 × fore wing, its subapical segments about 1.4 × as long as wide; frons granulate, rather shiny; OOL and POL 1.2 and 1.5 × width of posterior ocellus, respectively; vertex superficially granulate-coriaceous, rather shiny; clypeus convex and coriaceous; ventral margin of clypeus thick and depressed (Fig. 44); width of hypoclypeal depression 0.4 × minimum width of face (Fig. 44) and face coriaceous with some rugulae; length of eye 2.4 × temple in dorsal view and temple directly narrowed behind eye; head subglobular (Fig. 46); occiput behind stemmaticum coriaceous with satin sheen; occipital carina complete and dorsally arched (Fig. 46); clypeus partly above lower level of eyes (Fig. 44); length of malar space 0.4 × height of eye in lateral view; eyes somewhat protruding (Figs 44, 46).

*Mesosoma.* Mesoscutal lobes coriaceous-rugulose, matt, but medio-posteriorly longitudinally rugose and anteriorly low; notauli narrow and crenulate, but sculpture largely lost; prepectal carina medium-sized, reaching anterior border; precoxal area of mesopleuron, area below it and mesosternum largely reticulate-rugose; remainder of mesopleuron (including speculum) rugose or rugulose and matt (Fig. 38); metapleuron rugose, matt; mesosternal sulcus deep and sparsely crenulate; mesosternum rounded posteriorly; scutellum slightly convex, rugulose, and laterally with irregular carina; propodeum flattened, without tubercles and coarsely rugose, median carina incomplete, posterior 0.3 absent.

*Wings.* Fore wing: r 0.4 × 3-SR (Fig. 36); 1-CU1 horizontal, 0.5 × as long as 2-CU1; r-m 0.8 × 2-SR, and 0.5 × 3-SR; second submarginal cell medium-sized (Fig. 36); cu-a vertical, not parallel with CU1b, straight; 1-M straight and 1-SR angled with 1-M. Hind wing: apical half of marginal cell slightly widened; 2-SC+R short; m-cu obsolescent.

*Legs.* Tarsal claws with yellow bristles; hind coxa rugulose and with spaced oblique rugae, with satin sheen and 0.7 × as long as first tergite; hind trochantellus 2.4 × longer ventrally than wide; length of fore and hind femora 5.8 and 6.1 × their width, respectively (Figs 40–41); inner apex of hind tibia without distinct comb; length of inner hind spur 0.25 × hind basitarsus.

*Metasoma.* First tergite 1.1 × as long as wide posteriorly, convex anteriorly and dorsal carinae lamelliform protruding basally; first and second tergites longitudinally rugose, robust (Fig. 39), with distinct median carina; medio-basal area of second tergite minute; second suture narrow and crenulate; basal half of third tergite largely superficially coriaceous, with some fine longitudinal elements; third tergite with complete sharp lateral crease, absent on following tergites; ovipositor sheath densely setose and apically acute.

*Colour.* Dark brown; head (except stemmaticum), mesoscutum and scutellum medially, tegulum, legs (but femora largely infuscate), patch on posterior third of first tergite, large triangular patch on second tergite (Fig. 39) and anterior patch of third tergite brownish yellow; fourth–seventh tergites yellow; mouthparts, humeral plate and metasoma ventrally pale yellow; ovipositor sheath black; veins and pterostigma dark brown; wing membrane infuscate.

*Variation.* Antennal segments of ♀: 36(2), 37(4), 38(2), 39(3), 40(2). The male is unknown. Pale patches of first and third tergites sometimes absent; hind femur 6.1–7.0 × as long as wide and hind trochantellus 2.4–3.0 × longer ventrally than wide. Central antennal segments vary from 1.2–1.5 × as long as wide, but in one specimen about 2.2 times – although its metasoma (at least posteriorly) is female, it seems possible that this individual is an intersex.

##### Etymology.

From “angustus” (Latin for “narrow”) and “pteron” (Greek for “wing”), because of the narrow wings.

##### Distribution.

*British Isles (England, Scotland, Wales, Jersey), *Finland, *Greece, *Lithuania, *Netherlands, *Portugal (Azores), *Russia (Siberia), *China (Chongqing), *Japan (Honshu).

##### Note.

CO1 sequences obtained from the paratypes from Azores and China group closely with those from Britain, and this seldom-collected species appears to have a very wide distribution.

#### Aleiodes apiculatus

Taxon classificationAnimaliaHymenopteraBraconidae

(Fahringer, 1932)

Figs 48–49 , 50–62

Rogas apicalis Reinhard, 1863: 266 (not Brullé 1832) (examined).
Rhogas apiculatus Fahringer, 1932: 284 (replacement name).
Aleiodes apiculatus ; Shenefelt 1975: 1165; Papp 1991: 101 (as synonym of Aleiodes pallidator); Belokobylskij et al. 2003: 398.
Rogas (Aleiodes) negativus Tobias, 1961: 123; Belokobylskij 2000: 60 (as synonym of Aleiodes apiculatus (Fahringer, 1932); paratype in BMNH examined).
Aleiodes negativus ; Shenefelt 1975: 1165.

##### Type material.

Holotype of *Aleiodes apiculatus*, ♀ (ZMB), “Type”, “Coll. H. Rhd”, “**Germania**, [surroundings of] Bautzen”, “Holotypus *Rogas apiculatus* Reinh., 1863, ♀, Papp, 1983”, “*Aleiodes pallidator* Thunb., ♀, det. Papp, J., 1984/ var. apiculatus (Fahr.)”. Paratype of *Aleiodes negativus*, ♀ (BMNH) from **Russia** (Siberia: Tuvinskaya ASSR).

##### Additional material.

*England (V.C.s 9, 17, 20, 23, 24, 29, 31), *Poland. Specimens in NMS, BMNH, RMNH, AAC.

##### Molecular data.

MRS028 (England EF115455, CO1 + EF115440, 28S), MRS064 (England KU682218, CO1), MRS079 (England KU682222, CO1), MRS407 (England KU682239, CO1).

##### Biology.

This rather poorly-known species is a probably monophagous parasitoid of *Euproctis similis* (Fuessly) (Erebidae: Lymantriinae), from which we have seen 5 rearings (England, Poland; A.A. Allen, S.D. Beavan, M.R. Shaw, L. Sukovata) in addition to a reared paratype of *Aleiodes negativus* from the same host. Although evidently not obligatorily so (see below), it is probably largely univoltine, and the winter is passed inside the diapausing host larva. The host is arboreal, and when parasitised shows strong climbing behaviour just before being mummified, such that mummies are formed in exposed positions. It was readily reared from *Euproctis similis* in culture, but quantitative data are not available owing to high overwintering mortality. The notes that follow relate to a single, virgin, female. This female showed great interest in an egg mass of its host, antennating the dense covering of setae left by the female moth, and probing also with the ovipositor but probably without attempting to oviposit except into fully eclosed larvae as they exited from the felted covering. Neither legs nor antennae were used to manipulate such hosts, and the process was achieved with a single insertion of the ovipositor. In subsequent trials, second instar hosts were offered naked, and it was clear that there was an injection of a temporarily paralysing venom (detected by a clear jerk of the wings) before actual oviposition took place, although the ovipositor was usually not removed in the interim. As with the emerging first instars, the use of antennae or legs to hold the host was minimal so usually the ovipositor was all that was in contact with the host, pinning it against the substrate, and oviposition usually took about (often just over) 2 minutes, without a period of post-oviposition assessment or at most with only a minimal one. The long setae of third instar hosts were a good deal harder for the female to penetrate, but some ovipositions into this instar also occurred.

In culture *Aleiodes apiculatus* proved to be, like its host, partly plurivoltine. The host invariably overwinters as a partly grown larva in a densely spun hibernaculum, and the adult moths appear in the following vi/vii. In captivity, a small proportion of host larvae (available from about vii onwards) from the resulting eggs fed up rapidly and produced a second generation of the moth, while the majority developed only slowly and entered hibernation in the autumn (often not until the end of ix) while still relatively small, joined at that time by offspring of the second generation. The parasitoid invariably overwinters as a small larva within these diapausing hosts. From overwintering hosts mummification takes place in about (v–)vi the following year, and emergence of the adult parasitoids in about (vi–)vii, to oviposit into the young hosts that appear soon after. In host individuals with the accelerated growth pattern the host was mummified in about viii and the adult parasitoids emerged in ix (N = 4). At this time host larvae, from both generations, are still available prior to constructing their hibernacula. The cohort of hosts with accelerated growth that produced a second generation during the culture experiments arose in control groups as well as among the parasitised hosts, so this behaviour was not the result of having been parasitised: rather, it seems likely that only the growth of host individuals independently destined for a second generation would have provoked similar early development by the parasitoid. Hosts bearing the parasitoid entered winter diapause on average an estimated 8–10 days sooner than unparasitised ones. For the hibernaculum, the parasitised hosts constructed a weak outer web, moulted, and then made a much denser inner chamber isolated from the exuvium, while unparasitised controls usually moulted before commencing construction of a single chamber. Parasitised hosts (N = 10) broke diapause in spring over a period of 22 days, on average 8.0 days later than controls (N = 9) which emerged from their hibernacula over a period of 11 days (see also *Aleiodes pallidator* which exhibits similar behaviour).

Despite the possibility of plurivoltinism revealed in culture experiments the capture dates, in Britain (vi–)vii–ix(–x), suggest that a single generation of rather long-lived individuals is the norm. It appears to have colonised Britain only recently; the first specimens known to us were collected in 1999 in Berkshire, since when it has been taken in MV traps in the SE corner of England fairly regularly. It is unlikely to have been long-overlooked in Britain, as its rather common and attractive host larva is conspicuous, readily identified, often reared and, when mummified by this parasitoid, often easily seen in a sun-exposed position.

##### Diagnosis.

Head transverse in dorsal view and directly narrowed ventrally in anterior view; eye rather large; OOL 0.5 × width of posterior ocellus; scapus and pedicellus of ♀ at least partly blackish, contrasting with yellowish middle of antenna and antenna of ♀ in dorsal view bicoloured, first–fifth[–eighth] and few apical segments more or less dark brown, remainder of antenna yellowish, antenna of ♂ entire yellowish; antennal segments of ♀ 46–49; length of malar space of ♀ 0.25–0.30 × height of eye in lateral view (Fig. 60; of ♂ 0.30 times); speculum of mesopleuron granulate and with satin sheen; fore wing rather narrow (Fig. 50); vein m-cu of fore wing straight and angled to vein 2-CU1 (Fig. 50); pterostigma dark brown with its basal half largely pale yellow; hind femur about 4.5 × as long as its maximum width; hind trochantellus about 2.6 × as long ventrally as wide (Fig. 53); hind tibia completely brownish yellow (Fig. 53); tarsal claws with distinct fine pecten (Fig. 54); dorsal face of propodeum medium-sized and rounded posteriorly (Figs 51, 52), first metasomal tergite lamelliform protruding latero-anteriorly (Fig. 62); second metasomal tergite rather stout and with minute smooth triangular area medio-basally (Fig. 52); third tergite finely sculptured; apical half of third and fourth tergite without sharp lateral crease and superficially granulate and with satin sheen; fourth metasomal tergite of ♀ black latero-posteriorly (Figs 52, 53; of ♂ brownish yellow).

**Figures 48–49.**
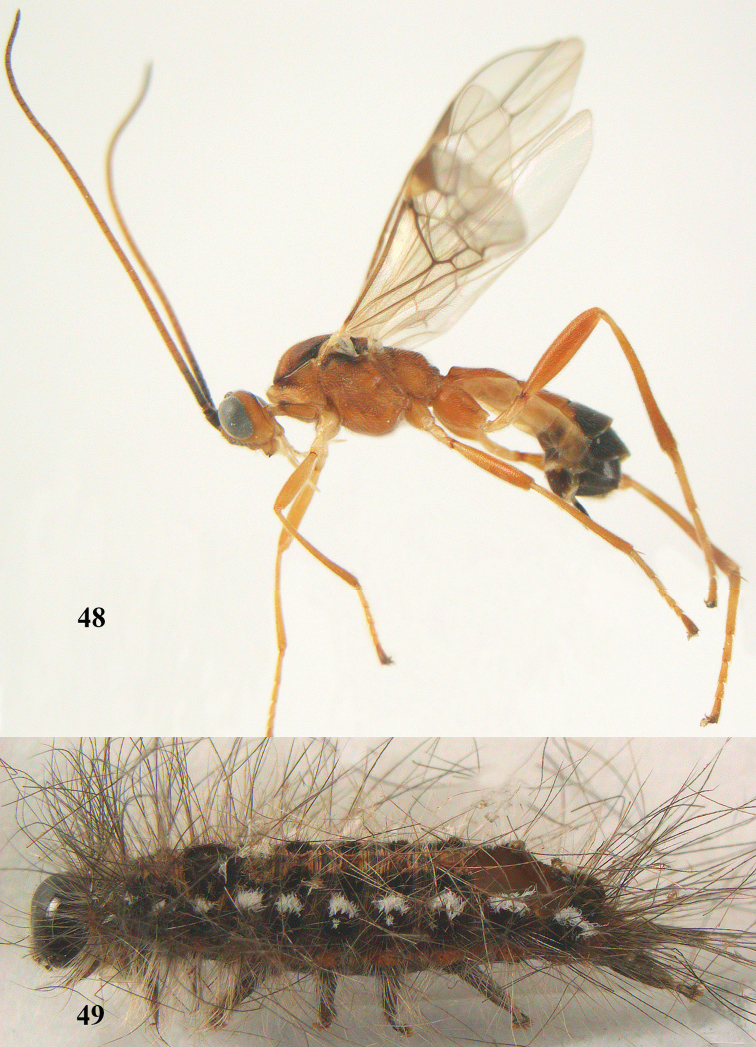
*Aleiodes apiculatus* (Fahringer), ♀, England. **48** habitus lateral **49** mummy of *Euproctis similis* (Fuessly).

**Figures 50–62.**
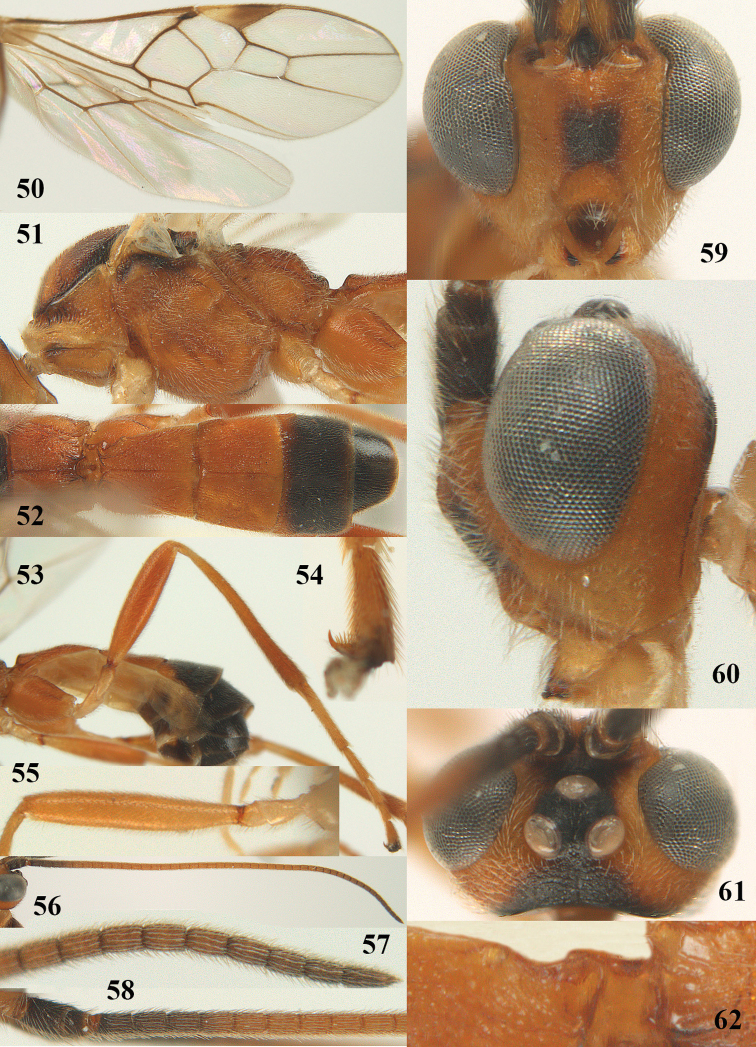
*Aleiodes apiculatus* (Fahringer), ♀, England. **50** wings **51** mesosoma lateral **52** propodeum and anterior half of metasoma dorsal **53** hind leg lateral **54** outer hind claw lateral **55** fore femur lateral **56** antenna **57** apical segments of antenna **58** basal segments of antenna **59** head anterior **60** head lateral **61** head dorsal **62** base of first tergite dorsal.

##### Description.

Redescribed ♀ (NMS) from England, length of fore wing 5.9 mm, of body 6.8 mm.

*Head.* Antennal segments of ♀ 47, length of antenna 1.2 × fore wing, its subapical segments 1.6 × as long as wide; frons granulate, with satin sheen and some rugae; OOL and POL 0.5 and 0.6 × width of posterior ocellus, respectively; stemmaticum strongly protruding; vertex rugulose-granulate, with satin sheen; clypeus convex and punctulate-coriaceous; ventral margin of clypeus thick and convex (Fig. 59); width of hypoclypeal depression 0.4 × minimum width of face (Fig. 59) and face mainly granulate with transverse rugulae; length of eye 3.4 × temple in dorsal view and temple moderately narrowed behind eye; occiput behind stemmaticum rugulose-granulate; occipital carina reduced medio-dorsally and complete ventrally, without crenulae and dorsally curved (Fig. 61); clypeus above lower level of eyes (Fig. 59); length of malar space 0.30 × height of eye in lateral view; eyes rather protruding (Figs 59–61).

*Mesosoma.* Length of mesosoma 1.7 × its height; mesoscutal lobes finely granulate, matt, but medio-posteriorly irregularly rugose and anteriorly high; notauli medium-sized and crenulate; prepectal carina medium-sized, remaining separate far from anterior border; precoxal area of mesopleuron and area above it distinctly rugose; remainder of mesopleuron (including speculum) granulate and with satin sheen (Fig. 51); metapleuron distinctly granulate and with satin sheen; mesosternal sulcus shallow and sparsely crenulate; mesosternum rather angulate latero-posteriorly but rounded medially; scutellum slightly convex, finely granulate, and antero-laterally with carina; propodeum weakly convex, without tubercles, anteriorly granulate, medially coarsely rugose and posteriorly with longitudinal carinae, median carina complete.

*Wings.* Fore wing: r 0.3 × 3-SR (Fig. 50); 1-CU1 slightly oblique, 0.5 × as long as 2-CU1; r-m 0.6 × 2-SR, and 0.45 × 3-SR; second submarginal cell rather long (Fig. 50); cu-a slightly inclivous, not parallel with CU1b, straight (Fig. 50); 1-M straight posteriorly and 1-SR angled to 1-M. Hind wing: marginal cell parallel-sided, but slightly narrowed submedially; 2-SC+R medium-sized, slender; m-cu short and only slightly pigmented; M+CU:1-M = 4:3; 1r-m 0.7 × 1-M.

*Legs.* Tarsal claws rather small and with distinct fine pecten (Fig. 54); hind coxa finely granulate, with satin sheen and 0.8 × as long as first tergite; hind trochantellus 2.6 × longer ventrally than wide; length of fore and hind femora 6.0 and 4.6 × their width, respectively; inner apex of hind tibia without distinct comb; length of inner hind spur 0.35 × hind basitarsus.

*Metasoma.* First tergite 1.1 × as long as wide posteriorly, stout, convex anteriorly and latero-anteriorly distinctly lamelliform; first and second tergites densely coarsely longitudinally rugose (Fig. 52), third tergite basally rugulose and with distinct median carina up to middle of third tergite; medio-basal area of second tergite minute; second suture rather wide and crenulate; remainder of third tergite granulate and following tergites shiny and superficially granulate; apical half of third and fourth tergites without sharp lateral crease; ovipositor sheath densely setose and apically truncate.

*Colour.* Brownish yellow; scapus and pedicellus of ♀ at least partly blackish, contrasting with yellowish middle of antenna and antenna of ♀ in dorsal view bicoloured, first–fifth[–eighth] and 2–3 apical segments more or less dark brown, remainder of antenna yellowish; malar space, mandible, palpi, tegulae, pronotum anteriorly, basal half of pterostigma, trochanters and trochantelli, fore and middle coxae, and ventral half of metasoma ivory or pale yellow; face medially, frons and vertex medially, stemmaticum, occiput dorsally, mesoscutum laterally narrowly, scutellar sulcus, axilla, scutellum posteriorly, metanotum, third (except antero-lateral corner)–sixth tergites, fourth–sixth sternites and ovipositor sheath black or dark brown; telotarsi slightly infuscate; veins and apical half of pterostigma dark brown; wing membrane slightly infuscate.

*Variation.* Antennal segments of ♀ 46(1), 47(10), 48(4), 49(1); ♂ 41(1), 42(2), 43(8), 44(3), 45(6), 46(2). Length of fore wing 5–6 mm, of body 5–7 mm. Males are brownish yellow, but stemmaticum black and antenna apically, occiput dorsally, mesoscutum laterally, scutellum posteriorly, metanotum, propodeum medially, first tergite except posteriorly and second tergite laterally somewhat infuscate; malar space, palpi, tegulae, pronotum, fore and middle coxae, trochanters and trochantelli, first tergite medio-apically and middle of second tergite pale yellowish.

##### Notes.

Similar to *Aleiodes pallidator* (Thunberg), but the latter differs by having the tarsal claws only bristly setose, the hind trochantellus ventrally 2.2 × as long as wide, the antennal segments of ♀ with 51–57 segments; the stemmaticum less protuberant, the pterostigma yellow and the body of ♀ entirely brownish yellow. The extent of dark colouration is highly variable, and is often poorly developed in the British population. In males especially, the colour (including scape and even stemmaticum) can be rather uniform orange to light honey-brown. Because it can lack the colour characters usually plain in females, the male of this species can superficially resemble some of the relatively large orange species with big ocelli and antennal segments in the range 41–48 that fall into the residual *circumscriptus*-group not dealt with in this paper. Good recognition characters for male *Aleiodes apiculatus* include its somewhat bristly antenna and legs, its enlarged fifth tarsal segment (especially in the fore leg), its relatively strongly sculptured second metasomal tergite with weak mediolateral depressions, its weakly pectinate claws, and the stronger (though weak) development of a comb at the apex of the hind tibia. The synonymy with *Aleiodes negativus* (Tobias) is accepted; the examined females of *Aleiodes apiculatus* have the antenna with 46–49 segments (the holotype has 49 segments). The examined paratype of *Aleiodes negativus* (BMNH) was reared from *Euproctis similis* and has 47 antennal segments. According to Tobias (1961) *Aleiodes negativus* female types (including the holotype) should have 35–38 antennal segments and the male types 42–43 segments; most likely the antennal counts for the female types given by Tobias result from a *lapsus* or typographical error and should be 45–48.

#### Aleiodes arcticus

Taxon classificationAnimaliaHymenopteraBraconidae

(Thomson, 1892)

Figs 63–64 , 65–75

Rogas arcticus Thomson, 1892: 1679; Tobias 1986: 83 (transl.: 138) (examined).
Rhogas arcticus ; Fahringer 1932: 285.
Aleiodes arcticus ; Shenefelt 1975: 1165–1166; Papp 1985a: 155 (lectotype designation), 1991: 96; Belokobylskij et al. 2003: 398.

##### Type material.

Lectotype, ♀ (ZIL), “Lpl” [= Lapland, North Sweden]).

##### Additional material.

3 ♀, 2 ♂ (G. Várkonyi personal coll., NMS) **Finland**, Ks. Salla 752.61, Värriö H, ex *Pygmaena fusca*, collection dates (of host larva) between 15.vi and 28.vi.1995, G. Várkonyi; 1 ♂ (G. Várkonyi personal coll.) Finland: Ks. Salla 752.61 Värriö H 21.vi.1995, G. Várkonyi; 1 ♀ (BMNH) Finland, Kuusano, Mäntytunturi, on snow, 29.vi.1935, G.J. Kerrich; 1 ♂ (NMS) S. **Norway**, Jotunheimen, Giendersheim, 1000–1500 m, 7.vii.[1966], J.E. & R.B. Benson; 2 ♂ (BMNH) S. Norway, Buskerud Fylke Geilo, 1000–1100 m, 16–24.vi.1965, J.E. & R.B. Benson; 1 ♂ (RMNH) Norway, Oppdal, Kongsvoll, Vestbekken, 28.vi.1978, J.O. Solem; 1 ♀ (BMNH) **Sweden**, Torne Lappmark, Tornehamn, 4.vii.1954, J.E. & R. B. Benson; 1 ♀, 1 ♂ (BMNH, NMS) **Switzerland**, Grisons, Müstairtal, Funt da S. Charl, 2400 m, 9.vi 1960 and 3.vii.1960, J.E. & R.B. Benson; 1 ♂ (BMNH) Switzerland, Grisons, Engadine National Park, 2380 m, 1.vii.1960, J.E. & R.B. Benson; 1 ♂ (BMNH) Switzerland, Valais, Arolla, 8000 ft, 9.vii.1935, J.E. & R.B. Benson; 1 ♂ (BMNH) Switzerland, Valais, near Verbier, 8000–8500 ft, 27.vi.1959, J.E. & R.B. Benson.

##### Biology.

This boreoalpine species is univoltine, passing the winter as a mummy. The only known host is the ennomine (Macariini) geometrid *Pygmaena fusca* (Thunberg) (5:1; G. Várkonyi/Finland), which feeds on *Empetrum* and *Vaccinium* (G. Várkonyi personal communication) and probably occurs throughout the range of the parasitoid. The small mummy (Fig. 64) is short, broad and dorsally elevated.

**Figures 63–64.**
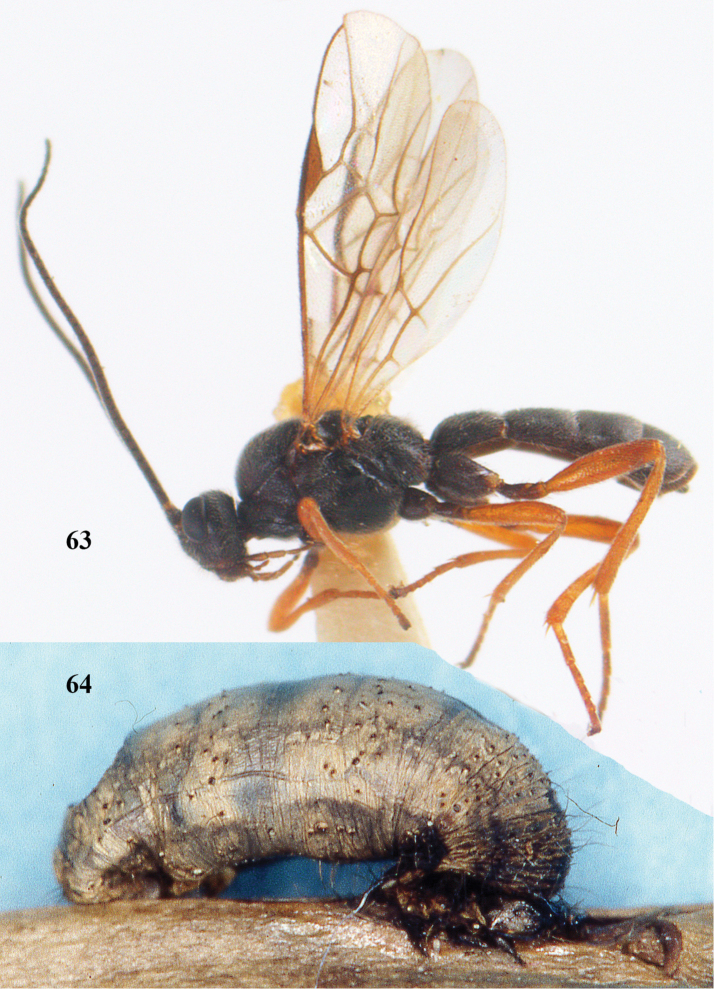
*Aleiodes arcticus* (Thomson), ♀, Switzerland. **63** habitus lateral **64** mummy of *Pygmaena fusca* (Thunberg) from Finland.

##### Diagnosis.

**Figures 65–75.**
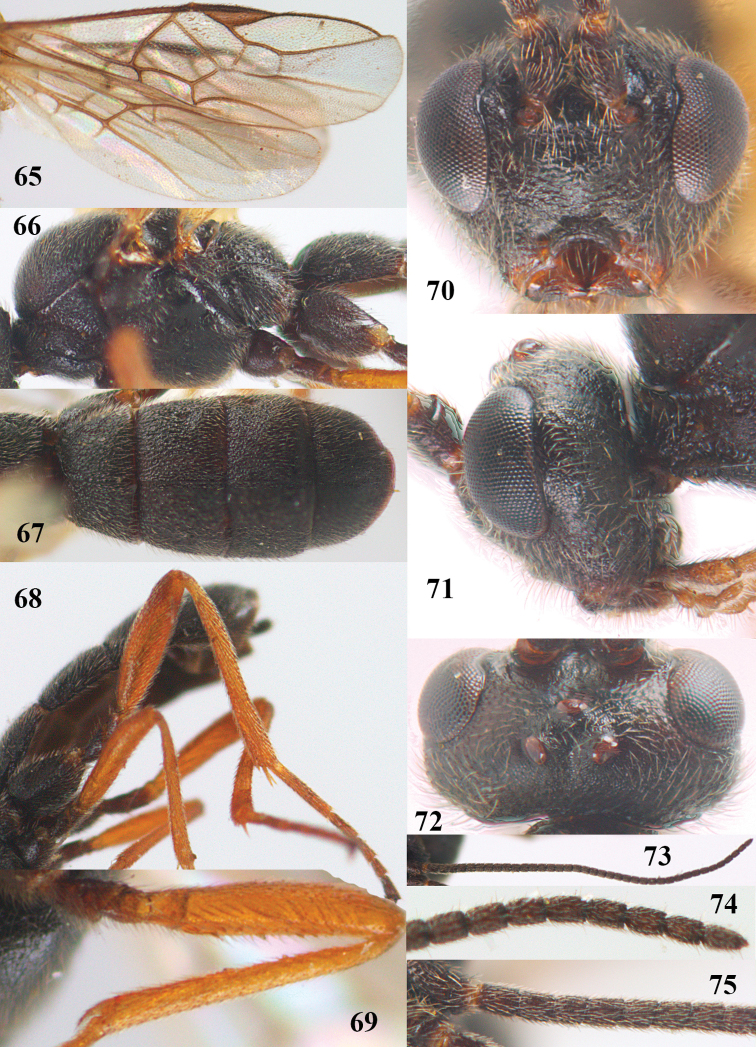
*Aleiodes arcticus* (Thomson), ♀, Switzerland. **65** wings **66** mesosoma lateral **67** metasoma dorsal **68** hind leg lateral **69** fore femur lateral **70** head anterior **71** head lateral **72** head dorsal **73** antenna **74** apical segments of antenna **75** basal segments of antenna.

Maximum width of hypoclypeal depression 0.3–0.4 × minimum width of face (Fig. 70); OOL 1.9 × diameter of posterior ocellus; mesoscutum, orbita and malar space black; precoxal sulcus largely granulate; trochanters, trochantelli and pterostigma largely black(ish); mesoscutum without a longitudinal carina on mesoscutum medio-posteriorly; apical half of marginal cell of hind wing parallel-sided or slightly widened; vein M+CU1 of fore wing apically at about same level as vein 2-CU1 (Fig. 65); vein r of fore wing 0.6–0.9 × vein 3-SR (Figs 63, 65); vein 1-SR of fore wing linear with vein 1-M (Fig. 65); all femora and tibiae reddish or yellowish brown; fore and hind femora moderately stout (Figs 68–69); fourth metasomal tergite curved posteriorly in dorsal view (Fig. 67), lateral crease distinct and following tergites more or less retracted; length of fore wing 3.4–3.9 mm.

##### Description.

Redescribed ♀ (BMNH) from Müstairtal (Switzerland), length of fore wing 3.7 mm, of body 4.6 mm.

*Head.* Antennal segments 40, length of antenna as long as fore wing, its subapical segments about 1.7 × as long as wide; frons mainly superficially granulate and with some rugulae anteriorly, weakly shiny; OOL 1.9 × diameter of posterior ocellus and granulate as vertex, with satin sheen; clypeus moderately convex, narrow and coriaceous; ventral margin of clypeus thick and depressed (Fig. 70); width of hypoclypeal depression 0.3 × minimum width of face (Fig. 70) and face mainly coriaceous with some rugulae dorsally and long setae; length of eye 1.1 × temple in dorsal view and temple subparallel-sided behind eye; occiput behind stemmaticum granulate and occipital carina absent ventrally and narrowly interrupted dorsally; clypeus below lower level of eyes (Fig. 70); length of malar space 0.5 × height of eye in lateral view; eyes somewhat protruding (Figs 70–72).

*Mesosoma.* Mesoscutal lobes largely granulate-coriaceous, matt and medio-posteriorly rugose, middle lobe without a longitudinal carina; notauli narrow, shallow and very finely crenulate; prepectal carina narrow lamelliform medio-ventrally, not reaching anterior border of mesopleuron; precoxal area of mesopleuron granulate; mesopleuron above precoxal area (except large smooth and shiny speculum) granulate, but dorsally finely rugose; medially metapleuron granulate and with some rugae, rather shiny; mesosternal sulcus narrow and rather deep, with longitudinal carina posteriorly; mesosternum rounded posteriorly; scutellum moderately convex, mainly granulate and largely non-carinate laterally; propodeum rather directly lowered posteriorly and granulate-rugose, median carina complete, without tubercles.

*Wings.* Fore wing: r 0.7 × 3-SR (Fig. 65); 1-CU1 subhorizontal, 0.1 × as long as 2-CU1; r-m 0.9 × 2-SR, and 0.8 × 3-SR; second submarginal cell rather small (Fig. 65); vein M+CU1 of fore wing apically at about same level as vein 2-CU1 (Fig. 65); vein 1-SR of fore wing linear with vein 1-M; cu-a subvertical, not parallel with CU1b, straight; 1-M nearly straight posteriorly. Hind wing: apical half of marginal cell slightly widened apically (Fig. 65); 2-SC+R short and longitudinal; m-cu present, pigmented.

*Legs.* Tarsal claws setose; hind coxa granulate-coriaceous, with satin sheen; hind trochantellus twice longer ventrally than wide; length of fore and hind femora 5.5 and 4.0 × their width, respectively (Figs 68–69); inner apex of hind tibia without comb; length of inner hind spur 0.4 × hind basitarsus.

*Metasoma.* First tergite 0.7 × as long as wide posteriorly, convex and latero-posteriorly non-lamelliform; first–second tergites finely and densely irregularly rugulose and with median carina (Fig. 67); medio-basal area of second tergite absent; second suture narrow, deep and finely crenulate; third tergite with median carina (except posteriorly), third–fourth tergites very finely rugulose-coriaceous; fourth tergite convex medially and apically; fourth tergite with sharp lateral crease; remainder of metasoma largely retracted; ovipositor sheath truncate apically and moderately setose.

*Colour.* Black (including coxae); palpi basally, tegulae, pterostigma, veins, trochanters and trochantelli dark brown; remainder of palpi and legs yellowish brown; wing membrane slightly infuscate.

*Variation*. Antennal segments of ♀ 39(1), 40(2), 41(0), 42(1), of ♂ 38(1), 39(2), 40(2); length of fore wing 3.4–3.7 mm; maximum width of hypoclypeal depression 0.3–0.4 × minimum width of face; vein r of fore wing 0.6–0.8 × vein 3-SR; median carina of middle mesoscutal lobe absent or weakly indicated; legs (except basally) vary from largely yellowish brown to largely dark brown with base of hind femur and tibiae paler than remainder of legs; second submarginal cell of fore wing rather variable in shape, but some are as trapezoidal as in *Aleiodes reticulatus*, with which this species is closely related.

##### Notes.

Recorded as British by Morley (1916), but in error as the three specimens (CMIM) on which the record was based have been examined and prove to belong to *Aleiodes similis* (Curtis). A series from Austria (Hohe Tauern, various altitudes ca 2300 m (MSC)) and also specimens from similar elevations in Switzerland (BMNH) have a habitus similar to *Aleiodes arcticus* but differ considerably from our concept of *Aleiodes arcticus* in being more coarsely rugose (including mesopleuron), the head being longer and behind the eyes narrower, outer orbits brownish (i. e. lighter in colour than the rest of the temple), antennal segments longer, second cubital cell usually longer. It is considered to be a different species placeable in the *bicolor*-group, but with apical tergites retracted and hind coxa short.

#### Aleiodes artesiariae sp. n.

Taxon classificationAnimaliaHymenopteraBraconidae

http://zoobank.org/536DFEB6-65AF-46FC-9F9E-DE22816FD463

Figs 76–77 , 78–88

##### Type material.

Holotype, ♀ (NMS, Edinburgh), “France, Olonne, ex *Macaria artesiaria*, mummy coll[ected] on *Salix repens*, vi.1984, N. Hall”.

##### Biology.

Apart from the host (determined as a result of adults of *Macaria artesiaria* (Denis & Schiffermüller) (Geometridae) being reared from caterpillars morphologically corresponding to the mummy and collected at the same time), nothing is known of the biology of this species. The holotype was excavated (fully formed but dead) from the mummy more than a year after it had been collected in apparently freshly made condition on a twig of its foodplant.

##### Diagnosis.

**Figures 76–77.**
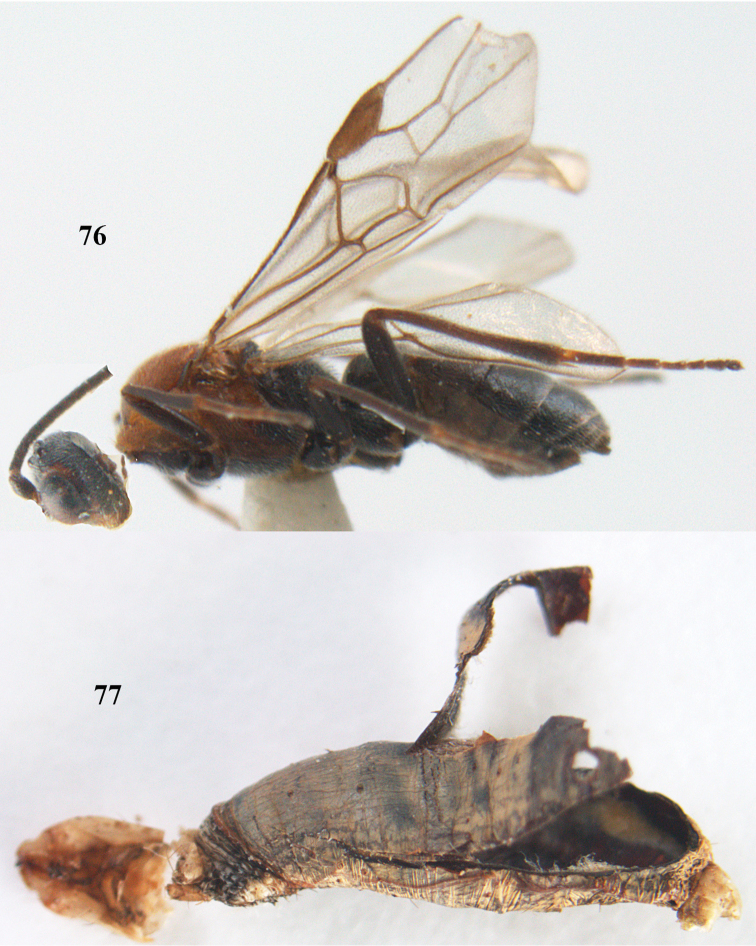
*Aleiodes artesiariae* sp. n., ♀, holotype. **76** habitus lateral **77** mummy of *Macaria artesiaria* (Denis & Schiffermüller) after extraction of adult.

**Figures 78–88.**
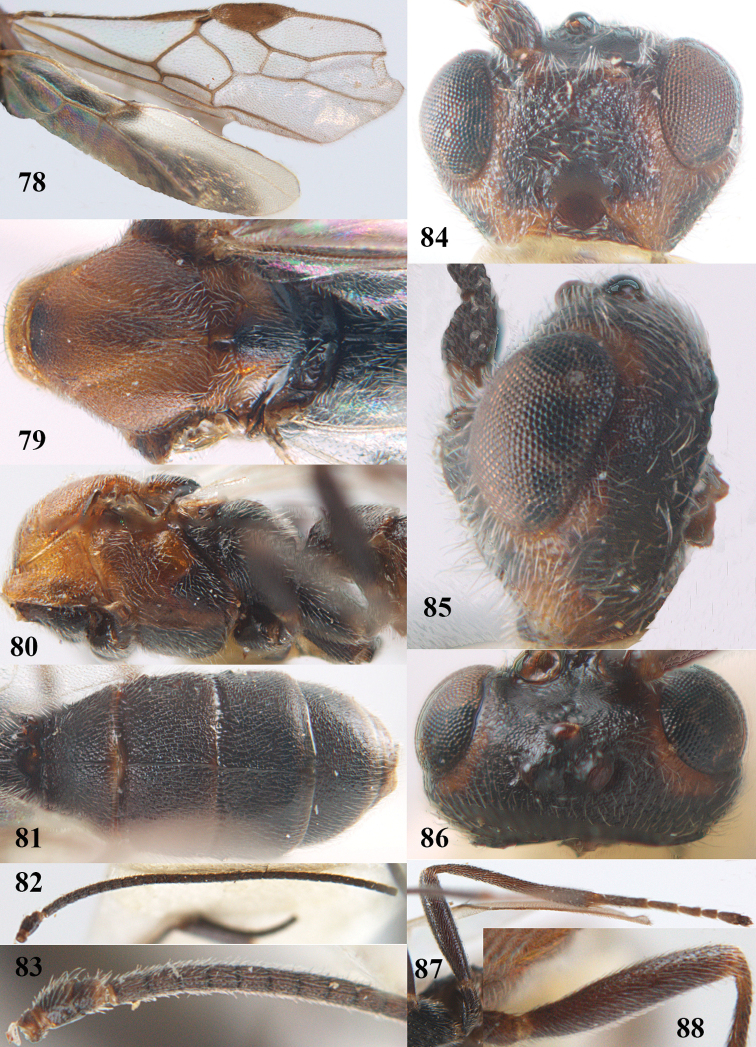
*Aleiodes artesiariae* sp. n., ♀, holotype. **78** wings **79** mesosoma dorsal **80** mesosoma lateral **81** metasoma dorsal **82** antenna (incomplete) **83** basal segments of antenna **84** head anterior **85** head lateral **86** head dorsal **87** hind leg lateral **88** fore femur lateral.

Maximum width of hypoclypeal depression 0.3 × minimum width of face (Fig. 84); OOL 2.4 × diameter of posterior ocellus; mesoscutum, orbita and malar space yellowish brown; precoxal sulcus largely granulate; trochanters, trochantelli and pterostigma largely dark brown or black(ish); mesoscutum without a longitudinal carina medio-posteriorly; apical half of marginal cell of hind wing parallel-sided or slightly widened; vein M+CU1 of fore wing apically above level of vein 2-CU1 (Fig. 78); vein r of fore wing about 0.7 × vein 3-SR (Figs 76, 78); vein 1-SR of fore wing angled with vein 1-M (Fig. 78); all femora and tibiae dark brown; fore and hind femora moderately stout (Figs 87–88); fourth metasomal tergite curved posteriorly in dorsal view (Fig. 81), lateral crease distinct and following tergites more or less retracted; length of fore wing about 3 mm. Very similar to *Aleiodes reticulatus* (Noskiewicz) but the latter differs by the black mesoscutum, the reddish or yellowish brown femora and tibiae, the less slender fore and hind femora (Figs 313–314 vs 87–88) and by having vein 1-SR of fore wing linear with vein 1-M (Fig. 305 vs 78).

##### Description.

Holotype, ♀, length of fore wing 2.9 mm, of body 3.6 mm.

*Head.* Antennal segments 33+ (incomplete), length of antenna at least as long as fore wing, its subapical segments somewhat longer than wide; frons mainly superficially granulate, moderately shiny; OOL 2.4 × diameter of posterior ocellus and granulate as vertex, with satin sheen; clypeus moderately convex, narrow and coriaceous, 0.4 × width of face; ventral margin of clypeus thick and depressed (Fig. 84); width of hypoclypeal depression 0.3 × minimum width of face (Fig. 84) and face mainly coriaceous with some rugulae dorsally and long setae; length of eye 1.8 × temple in dorsal view and temple sub-parallel-sided behind eye and narrowed posteriorly; occiput behind stemmaticum granulate and occipital carina absent ventrally and complete (but irregular) dorsally; clypeus partly up to lower level of eyes (Fig. 84); length of malar space 0.4 × height of eye in lateral view; eyes slightly protruding (Figs 84–86).

*Mesosoma.* Mesoscutal lobes largely granulate-coriaceous, matt and medio-posteriorly rugose, middle lobe without a longitudinal carina; notauli narrow, shallow and very finely crenulate; prepectal carina narrow lamelliform medio-ventrally, not reaching anterior border of mesopleuron; precoxal area of mesopleuron granulate; mesopleuron above precoxal area (except large smooth and shiny speculum) granulate, but dorsally rugose; medially metapleuron granulate, rather shiny; mesosternal sulcus narrow and rather deep, without longitudinal carina posteriorly; mesosternum rather angulate posteriorly; scutellum moderately convex medially and depressed laterally, mainly granulate and largely non-carinate laterally; lunula moderately wide; propodeum rather directly lowered posteriorly and granulate-rugose, median carina complete, without tubercles.

*Wings.* Fore wing: r 0.7 × 3-SR and linear with 3-SR (Fig. 78); 1-CU1 oblique and widened, 0.25 × as long as 2-CU1; r-m 0.8 × 2-SR, and 0.7 × 3-SR; second submarginal cell rather small and square (Fig. 78); vein M+CU1 of fore wing apically above level of vein 2-CU1 (Fig. 78); vein 1-SR of fore wing angled with vein 1-M; cu-a subvertical, not parallel with CU1b, straight; 1-M straight posteriorly. Hind wing: apical half of marginal cell slightly widened apically (Fig. 78); 2-SC+R medium-sized and longitudinal; m-cu present and slightly pigmented.

*Legs.* Tarsal claws setose; hind coxa granulate-coriaceous, with satin sheen; hind trochantellus 2.2 × longer ventrally than wide; length of fore and hind femora 5.4 and 5.2 × their width, respectively (Figs 87–88); inner apex of hind tibia without comb; length of inner hind spur 0.35 × hind basitarsus.

*Metasoma.* First tergite 0.7 × as long as wide posteriorly, convex and non-lamelliform latero-posteriorly and basally; first–second tergites finely and densely irregularly rugulose and with median carina (Fig. 81); medio-basal area of second tergite absent; second suture narrow, deep and finely crenulate; third tergite superficially coriaceous and with median carina (except posteriorly), third–fourth tergites very finely rugulose-coriaceous; fourth tergite convex medially and apically, shiny and with sharp lateral crease; remainder of metasoma largely retracted; ovipositor sheath truncate apically and moderately setose.

*Colour.* Black (including coxae, middle and hind trochanters); palpi, tegulae, pterostigma, veins, first and second tergites and remainder of legs dark brown; malar space, orbita, mesoscutum (but middle lobe somewhat infuscate medio-anteriorly), scutellum laterally, pronotum, mesopleuron (except postero-ventrally) yellowish brown; wing membrane slightly infuscate.

##### Etymology.

From the specific epithet of its host.

##### Distribution.

*France.

#### Aleiodes bistrigatus

Taxon classificationAnimaliaHymenopteraBraconidae

Roman, 1917 stat. rev.

Figs 89–98

Aleiodes circumscriptus var. bistrigatus Roman, 1917: 9; Shenefelt 1975: 1171; Papp 1991: 109 (as synonym of Aleiodes borealis) (examined).

##### Type material.

Lectotype here designated, ♀ (NRS), “Färöar [= **Faroe Isl., Denmark**], Klinck”, “Triangisvaag”, “♀ Aleiodes circumscriptus var. bistrigatus Roman, C. van Achterberg, 1984. Lectotype”, “178, 84”, “Riksmuseum Stockholm”, “NHRS-HEVA 000003802”. Paralectotypes: 3 ♂ (NRS) with same locality labels as lectotype.

##### Additional material.

None.

##### Biology.

Unknown.

##### Diagnosis.

Apical half of hind femur (partly) dark brown, darker than hind trochanter and trochantellus; face with distinct rugae; antenna of female with 39 segments and third segment stout, 4^th^–7^th^ segments moderately stout (but less than in *Aleiodes diarsianae*; Fig. 164), of male with 38 segments; OOL 1.7 × diameter of posterior ocellus (Fig. 97); clypeus 0.4 × as wide as face (Fig. 95); mesopleuron black dorso-posteriorly (Fig. 90); malar space and inner orbita dark brown and temple near eye (= external orbita) reddish-brown; first tergite strongly widened apically; eye (of male) elongate in lateral view; middle third of hind femur yellowish brown or dark brown; vertex moderately setose, rather shiny and blackish posteriorly; mesosternum dark brown. Close to *Aleiodes borealis* (Thomson, 1892), but this species has less antennal segments (♀: 32–34 segments), palpi and legs more or less infuscate and the clypeus wider (about 0.5 × width of the face). The shape of the subbasal antennal segments is similar to that of series from Scandinavia mentioned under *Aleiodes diarsianae*, but this series has the temples directly narrowed behind the eyes and the males have 42–47 antennal segments and females 44–45. Similar to the *Aleiodes pictus*-aggregate (e.g. *Aleiodes pictus* (Herrich-Schäffer, 1838) and *Aleiodes nigriceps* Wesmael, 1838), but these are usually smaller and less robust species, having the face without distinct rugae or only a few rugae dorsally, the mesosternum usually widely orange-brown (and the mesopleuron usually without rugae in *Aleiodes nigriceps*), the malar space partly or completely yellowish brown, the first tergite less widened apically, the fore and hind tarsi comparatively slender, the eye normal in lateral view and the antenna less robust.

**Figures 89–98.**
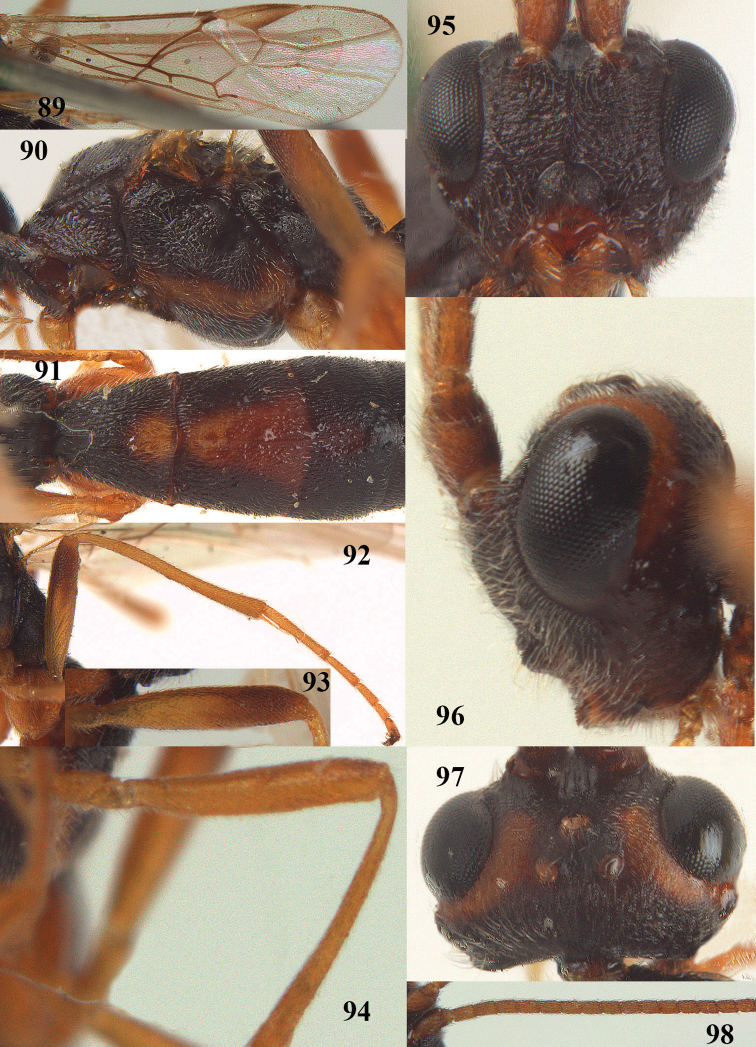
*Aleiodes bistrigatus* Roman, ♀, lectotype. **89** wings **90** mesosoma lateral **91** basal half of metasoma dorsal **92** hind leg lateral **93** hind femur lateral **94** fore femur lateral **95** head anterior **96** head lateral **97** head dorsal **98** basal segments of antenna.

##### Description.

Lectotype, ♀, length of fore wing 4.0 mm, of body 5.5 mm.

*Head.* Antennal segments of ♀ 39, length of antenna 1.3 × fore wing, its subapical segments about 1.9 × as long as wide and third segment stout (Fig. 98); frons coriaceous and posteriorly rugulose, with satin sheen; OOL 1.7 × diameter of posterior ocellus and coriaceous; vertex rugulose-coriaceous, rather shiny; clypeus slightly convex, indistinctly sculptured; ventral margin of clypeus rounded and depressed (Fig. 95); width of hypoclypeal depression 0.38 × minimum width of face and face distinctly transversely rugose (Fig. 95); length of eye 1.6 × temple in dorsal view and temple gradually narrowed behind eye (Fig. 97); occiput behind stemmaticum coriaceous and with some rugulae, occipital carina interrupted dorsally; clypeus partly above lower level of eyes and 0.4 × as wide as face (Fig. 95); length of malar space 0.6 × length of eye in lateral view; eyes slightly protruding (Figs 95–97).

*Mesosoma.* Mesosoma 1.7 × as long as high; mesoscutal lobes coriaceous, matt, but medio-posteriorly longitudinally rugose; notauli complete and moderately wide, weakly crenulate and posteriorly widened and rugose; prepectal carina medium-sized and lamelliform, reaching anterior border; precoxal area of mesopleuron very coarsely rugose, connected to rugosity of dorso-anterior part of mesopleuron; speculum nearly smooth and shiny (Fig. 90); metapleuron granulate, matt and posteriorly rather tuberculate; mesosternal sulcus narrow and deep; mesosternum rounded posteriorly; scutellum elongate, slightly convex, coriaceous and laterally largely carinate; propodeum rather flat dorsally, laterally and apically rather rugose, anteriorly only weakly so, median carina complete, but posteriorly irregular.

*Wings.* Fore wing: r 0.3 × 3-SR (Fig. 89); 1-CU1 horizontal, 0.5 × as long as 2-CU1; r-m 0.65 × 2-SR, and 0.40 × 3-SR; second submarginal cell medium-sized (Fig. 89); cu-a vertical, not parallel with CU1b, straight; 1-M slightly curved posteriorly and not continuous with 1-SR. Hind wing: apical half of marginal cell parallel-sided or nearly so; 2-SC+R short and longitudinal; m-cu present; 1r-m distinctly oblique and 0.7 × 1-M.

*Legs.* Tarsal claws setose; hind coxa coriaceous but partly superficially rugulose, largely matt; hind trochantellus twice longer ventrally than wide; length of fore and hind femora 5.4 and 5.0 × their width, respectively (Figs 93–94); inner apex of hind tibia without comb; length of inner hind spur 0.35 × hind basitarsus.

*Metasoma.* First tergite 0.9 × as long as wide posteriorly and latero-posteriorly narrowly lamelliform, moderately convex and flattened posteriorly, dorsope comparatively wide (Fig. 91); first–third tergites densely and distinctly longitudinally rugose, robust (Fig. 91), with distinct median carina; medio-basal area of second tergite absent; second tergite 1.5 × as long as third tergite; second suture moderately impressed and crenulate; remainder of metasoma largely superficially coriaceous; fourth and apical fifth of third tergite without sharp lateral crease; ovipositor sheath (except dorsally) densely setose.

*Colour.* Black or brownish black; antenna yellowish brown, but scapus dorsally and apical seventh of antenna dark brown; palpi, temple near eyes, legs (except infuscate subapical part of hind femur), tegulae, longitudinal stripe on mesopleuron, mesoscutum posteriorly, metasoma baso-ventrally, first tergite medio-apically, middle of second tergite and third tergite medio-basally largely yellowish; veins and pterostigma (except yellowish basal third and centrally) dark brown; border between dark and pale part of pterostigma diffuse (Fig. 89); wing membrane subhyaline.

*Variation.* The male paralectotypes are very similar to the lectotype; one has a complete antenna with 38 segments and most of the hind femur darkened. One paralectotype has the hind coxa completely yellowish and the mesopleuron less coarsely rugose, but other paralectotypes have the mesopleuron coarsely sculptured and the hind coxa largely infuscate.

##### Note.

Possibly a Faroe Islands endemic.

#### Aleiodes cantherius

Taxon classificationAnimaliaHymenopteraBraconidae

(Lyle, 1919)

Figs 99–100 , 101–112

Rogas cantherius Lyle, 1919: 153–154 (examined).
Aleiodes cantherius ; Shenefelt 1975: 1169; Papp 1991: 112 (as possible synonym of Aleiodes nigricornis).

##### Type material.

Lectotype here designated, ♀ (BMNH), “2504” [on card], “cotype”, “[**England**,] New Forest, 4.v.1914, ex *Semiothisa liturata*, G.T. Lyle”, “G.T. Lyle Coll., B.M. 1930-579”, “*Rhogas cantherius* Lyle”. Paralectotypes: 4 ♀ + 3 ♂ (BMNH, CMIM), topotypic and from same host, but one non-reared paralectotype from Harwood collection.

##### Additional material.

*Austria, **British Isles** (**England**: V.C.s 11, 17, 19, 22, 24, 25, 28, 56), *Germany, *Netherlands (Breda; Melissant; Wageningen), *Russia, Slovakia, *Sweden. . Specimens in NMS, BMNH, RMNH, ZISP, FRAH, CC, CMIM, SDEI.

##### Molecular data.

MRS777 (Sweden KU682249, CO1)), MRS787 (Sweden KU682253, CO1).

##### Biology.

A parasitoid of conifer-feeding *Macaria* species (Geometridae), overwintering as a mummy. Specimens (in NMS unless specified) reared from *Macaria liturata* (Clerck) (22 [6 are BMNH, 3 CMIM, 2 SDEI, 1 FRAH]; P.E. Hatcher, G.T. Lyle, M.R. Shaw/England); *Macaria signaria* (Hübner) (2:2 [CC]; M. Čapek/Slovakia). Additionally 5 reared specimens, fortunately accompanied by the host mummy, had been labelled as reared from other conifer-feeding geometrids (*Bupalus piniaria* (Linnaeus) (2, ZISP), *Eupithecia indigata* (Hübner) (2, FRAH), *Hylaea fasciaria* (Linnaeus) (1, ZISP)) but in all cases examination of the host remains established that the host was in fact more consistent with a species of *Macaria*. Plurivoltine, overwintering in a mummy constructed on a conifer needle. The adult occurs in the field from May until well into September, and it is clear from the rearing data that its conifer-feeding *Macaria* hosts are likely to be sought across all of their foodplants (*Abies cephalonica*, *Larix decidua*, *Pinus strobus*, *Pinus sylvestris* and *Pseudotsuga menzieseii* are indicated on the data labels overall). No experimentation has been undertaken.

##### Diagnosis.

Antennal segments of ♀ 39–43, of ♂ 40–43; head strongly narrowed behind eyes (Fig. 110), yellowish anteriorly and mainly dark brown dorsally; OOL 0.6 × diameter of posterior ocellus; length of malar space of ♀ 0.2–0.3 × height of eye in lateral view (Fig. 109); scapus in lateral view rather oblique apically; occipital carina interrupted dorsally and complete ventrally (Fig. 109); eye 3.5–6.5 × as long as temple in dorsal view (Fig. 110); mesosternum and precoxal sulcus superficially granulate and with satin sheen; vein 2-CU1 of fore wing about 3 × vein 1-CU1 (Fig. 101); vein 1-SR narrow and linearly connected to vein 1-M and vein 1-M straight (Fig. 101); hind femur stout (Fig. 104); fourth metasomal tergite largely (superficially) coriaceous and shiny; length of fore wing 4–5 mm.

**Figures 99–100.**
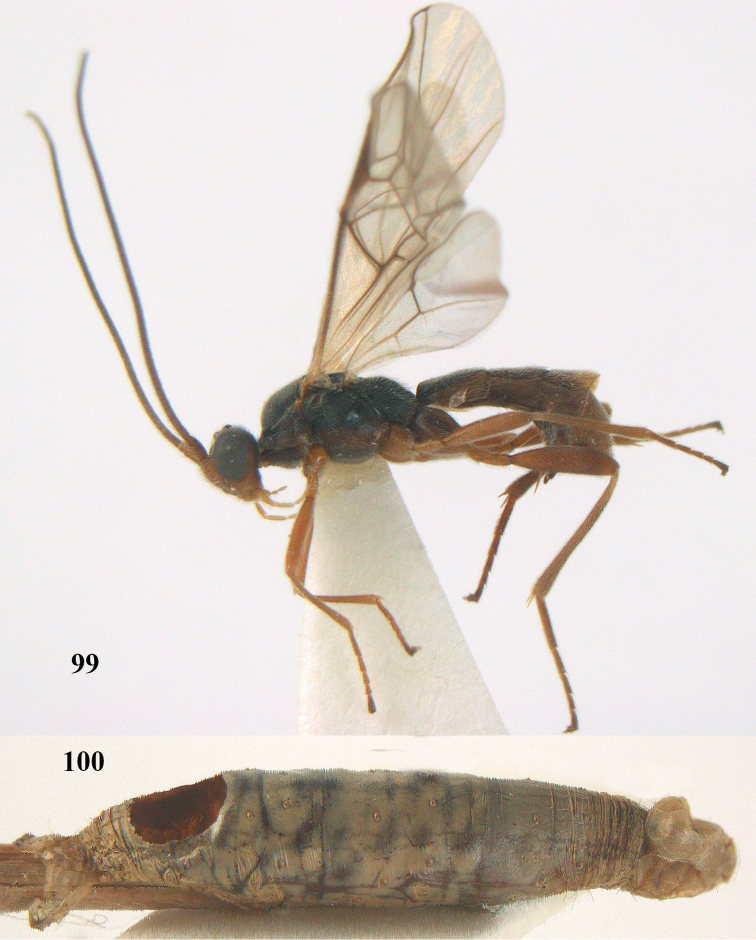
*Aleiodes cantherius* (Lyle), ♀, England. **99** habitus lateral **100** mummy of *Macaria* sp.

**Figures 101–112.**
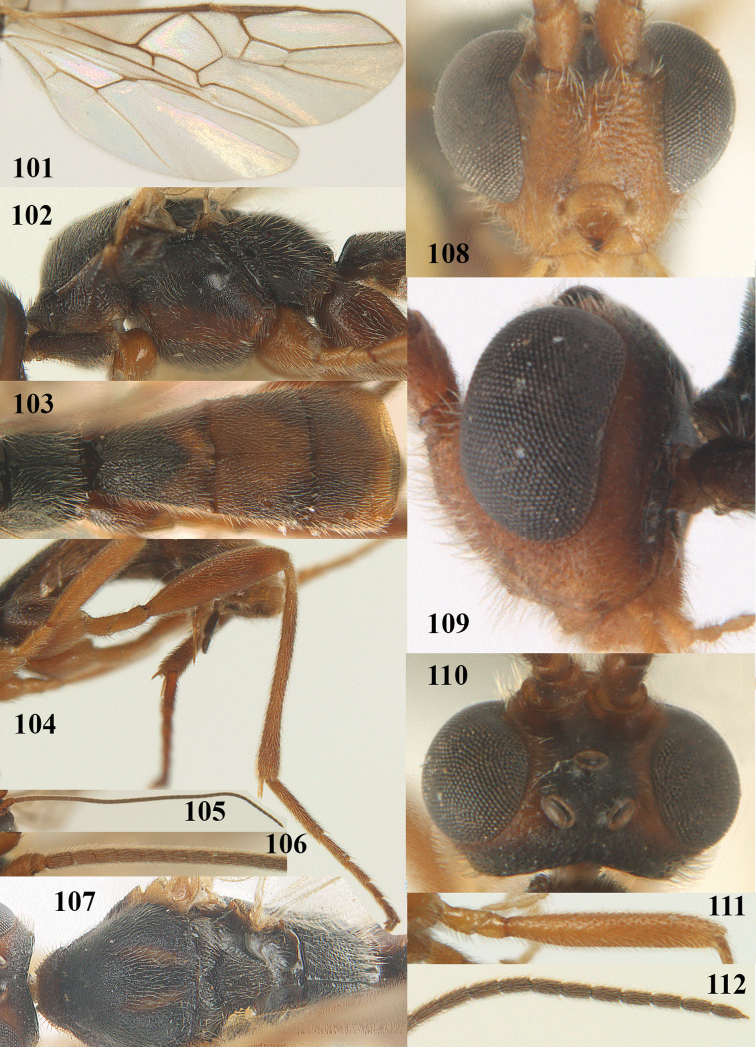
*Aleiodes cantherius* (Lyle), ♀, England. **101** wings **102** mesosoma lateral **103** propodeum and metasoma dorsal **104** hind leg lateral **105** antenna **106** basal segments of antenna **107** mesosoma dorsal **108** head anterior **109** head lateral **110** head dorsal **111** fore femur lateral **112** apical segments of antenna.

##### Description.

Redescribed ♀ (NMS) from Santon Downham (England), length of fore wing 4.6 mm, of body 5.0 mm.

*Head.* Antennal segments 43, length of antenna 1.3 × fore wing, its subapical segments about 2.3 × as long as wide (Fig. 112) and scapus in lateral view rather oblique apically; frons granulate and rather shiny; OOL and POL 0.6 and 0.8 × diameter of posterior ocellus, respectively and granulate; vertex granulate, dull; clypeus rather high, convex, coriaceous; ventral margin of clypeus thick (Fig. 108); width of hypoclypeal depression 0.3 × minimum width of face (Fig. 108) and face mainly transversely rugose and granulate; length of eye 6.5 × temple in dorsal view and temple directly narrowed behind eye; occiput behind stemmaticum mainly granulate and occipital carina interrupted medio-dorsally and complete ventrally (Fig. 109); clypeus above lower level of eyes (Fig. 108); length of malar space 0.3 × height of eye in lateral view; eyes protruding (Fig. 109).

*Mesosoma.* Mesoscutal lobes very finely coriaceous, with satin sheen, but medio-posteriorly with some rugae; notauli narrow, shallow and largely smooth; prepectal carina rather lamelliform medio-ventrally, nearly reaching anterior border of mesopleuron and latero-ventrally curved; precoxal area of mesopleuron granulate; mesopleuron above precoxal area (except large smooth and shiny speculum) superficially granulate, but dorsally rugulose; medially metapleuron superficially granulate, rather shiny; mesosternal sulcus narrow and rather deep, micro-crenulate, without carina posteriorly; mesosternum rather angulate posteriorly; scutellum finely coriaceous and non-carinate laterally; dorsal face of propodeum medium-sized, convex and rugulose, but posteriorly with some carinae and smooth in between and anteriorly mainly granulate, median carina complete, without tubercles.

*Wings.* Fore wing: r 0.5 × 3-SR (Fig. 101); 1-CU1 horizontal, 0.4 × as long as 2-CU1; r-m 0.7 × 2-SR, and 0.4 × 3-SR; second submarginal cell elongate (Fig. 101); 1-SR slightly angled to 1-M and slender; cu-a rather inclivous, not parallel with CU1b, straight; 1-M slightly curved. Hind wing: apical half of marginal cell parallel-sided or nearly so (Fig. 101); 2-SC+R longitudinal; m-cu present as fold, unpigmented; M+CU:1-M = 3:2; 1r-m 0.7 × 1-M.

*Legs.* Tarsal claws yellowish setose; hind coxa superficially finely coriaceous, rather shiny; hind trochantellus 2.4 × longer ventrally than wide; length of fore and hind femora 6.2 and 4.0 × their width, respectively (Figs 104, 111); inner apex of hind tibia without comb; length of inner hind spur 0.3 × hind basitarsus.

*Metasoma.* First tergite 1.2 × as long as wide posteriorly, flattened and latero-anteriorly narrowly lamelliform; first–second tergites and base of third tergite densely finely longitudinally rugose and with median carina; second tergite stout, 0.8 × longer than wide basally and 1.2 × as long as third tergite (Fig. 103); medio-basal area of second tergite minute; second suture deep and distinctly crenulate; remainder of metasoma largely superficially coriaceous and rather shiny; apical half of third and fourth tergite without sharp lateral crease; ovipositor sheath largely densely setose and apically truncate.

*Colour.* Black or dark brown; palpi, pronotum postero-dorsally and tegulae pale yellowish; scapus and pedicellus ventrally (but dorsally more or less darkened), orbita, two stripes on mesoscutum, legs (but hind coxa more or less dark brown), first tergite medio-apically, second tergite (except postero-lateral corners), third and following tergites mainly yellowish brown (Figs 99, 103); pterostigma and veins dark brown, but base of pterostigma and vein 1-R1 of fore wing brownish yellow; wing membrane slightly infuscate.

*Variation.* Length of fore wing 3.6–4.5 mm, of body 3.8–4.6 mm; antennal segments of ♀ 39(1), 40(2; one is lectotype), 41(6), 42(2), 43(4), of ♂ 39(1), 40(6), 41(8), 42(3), 43(3); specimens have a characteristic pair of more or less obscure dorsal orange brown marks on the otherwise dark mesoscutum. Males examined have the metasoma dark brown apically, hind tibia (except ivory base) and tarsus more or less infuscated.

##### Note.

The two sexes have about the same number of antennal segments.

#### Aleiodes carminatus sp. n.

Taxon classificationAnimaliaHymenopteraBraconidae

http://zoobank.org/5C58514A-72B5-46E9-998D-4B491A436068

Figs 113 , 114–124

##### Type material.

Holotype, ♀ (NMS, Edinburgh), “[**France**:] Corsica: Corte, Val de Restonica (Hôtel Colonna), 500 m, [at] light, 29.vii–3.viii.[20]01, M.R. Shaw”, “MRS *Aleiodes* DNA 102”. Paratypes (11 ♀ + 23 ♂): 1 ♀ + 1 ♂ (NMS, RMNH), same data as holotype; 1 ♀ (BMNH), “[**Spain**:] Mallorca, Sa Roca, P.N. de s’Albufera, MV light, 2–27.ix.2013, M.R. Honey BMNH(E) 2013-158”; 1 ♀ (NMS), “Spain: Zaragoza Prov., Los Monegros, Retuerta de Pina, 30TYL 27.94, J. Blasco-Zumeta, 5104, 8.viii.[19]92, NMSZ1997.026, swept from *Suaeda vermiculata*”; 2 ♀ + 1 ♂ (NMS, RMNH), id., but 28.vi.1992 and swept from *Suaeda vera*, ♂ swept from *Osyris alba*; 3 ♀ (NMS, RMNH), id., but 12.ix.1991 (1) or 10.ix.1993 (2) and collected at light; 2 ♂ (NMS, RMNH), id., but 10.vii.1993, at light; 9 ♂ (NMS, RMNH), id., but 10.ix.1993; 3 ♂ (NMS, RMNH), id., but 20.ix.1993; 2 ♂ (NMS, RMNH),, id., but ?1991; 2 ♂ (NMS), id., but 20.vii.1993; 1 ♂ (NMS), id., but 20.viii.1993; 2 ♀ (FC), “Esp.: Valencia, El Saler (Casal d’Esplai), T.M., 20–27.vii.1992 & 17–24.viii.1992, J.V. Falcó y F. Luna”; 2 ♂ (FC), “Esp.: Valencia, Moncada-TM blanca, 6–13.vii.1992 & 13–20.vii.1992, M.J. Verdú”; 1 ♀, (NMS), “[Spain:] Canary Islands, Tenerife, Anco Viejo, La Sabinita, 20.iii.1999, R.R. Askew”.

##### Molecular data.

MRS055 (Corsica JF962818, CO1), MRS098 (Corsica KU682224, CO1), MRS102 (Corsica KU682225, CO1).

##### Biology.

Unknown. This species is active at night and occurs in open habitats suggesting that its hosts live in low vegetation, but its voltinism is unclear.

##### Diagnosis.

Head weakly transverse (Fig. 122); body slender and entirely brownish yellow; antenna of ♀ (except scapus) dark brown; antennal segments of ♀ 34–37, of ♂ 35–40; eye rather small (Figs 121–123)); OOL equal to width of posterior ocellus; length of malar space of ♀ 0.5 × height of eye in lateral view (Fig. 123); speculum of mesopleuron smooth and shiny or superficially granulate; propodeum slightly elongate (Fig. 115); fore wing rather narrow (Fig. 114); vein m-cu of fore wing straight and angled to vein 2-CU1 (Fig. 114); pterostigma pale yellowish basally; hind coxa distinctly shorter than first tergite; fore wing subhyaline; apex of hind tibia with comb at inner side (Fig. 119); hind femur 5 × as long as its maximum width; hind trochantellus slender (Fig. 117); dorsal carinae of first metasomal tergite lamelliform protruding basally; second tergite without triangular area medio-basally (Fig. 116); third tergite weakly sculptured; fourth tergite without sharp lateral crease, fourth and following tergites partly retracted and largely smooth. Resembles *Aleiodes testaceus* (Telenga, 1941), but *Aleiodes testaceus* has no apical comb of the hind tibia (present in *Aleiodes carminatus*); surrroundings of precoxal sulcus largely smooth and shiny (mainly granulate and moderately shiny); veins 1-SR and r of fore wing longer (shorter); antenna yellowish brown basally (dark brown) and length of malar space 0.3–0.4 × height of eye in lateral view (0.5 ×). Resembles superficially *Aleiodes curticornis* nom. n., but *Aleiodes curticornis* has no apical comb of the hind tibia (present in *Aleiodes carminatus*); fore femur, third and penultimate antennal segments robust (slender) and antenna yellowish brown basally (dark brown). The presence of a hind tibial comb distinguished it from all the pale members of the *Aleiodes circumscriptus* group not treated in this paper.

**Figure 113.**
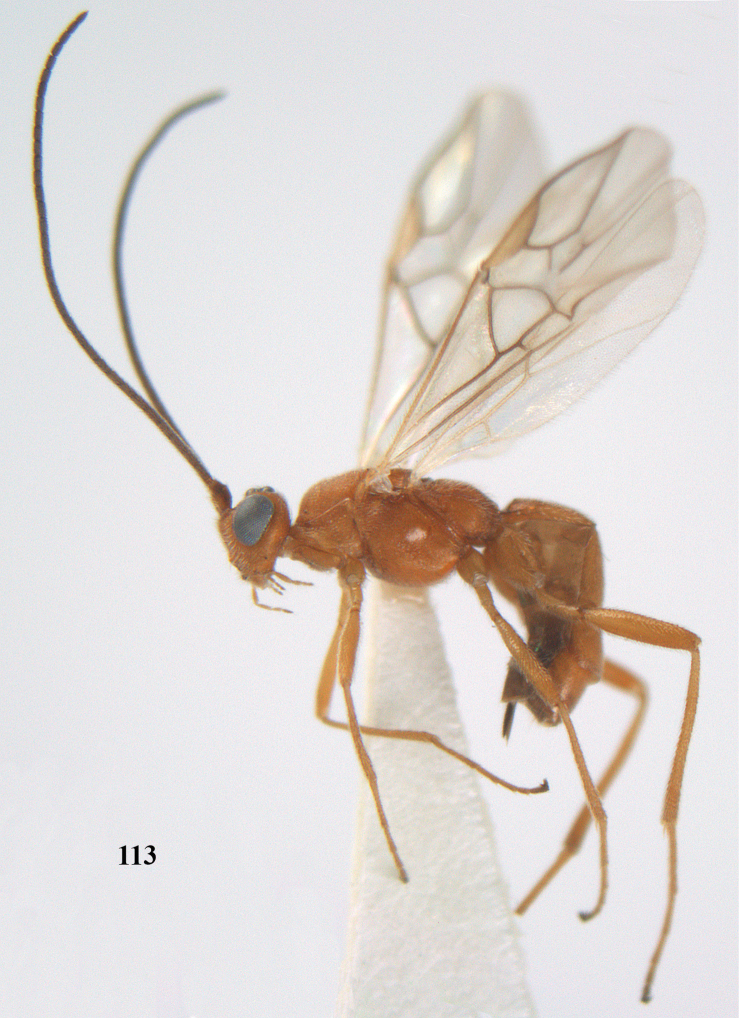
*Aleiodes carminatus* sp. n., ♀, holotype, habitus lateral.

**Figures 114–124.**
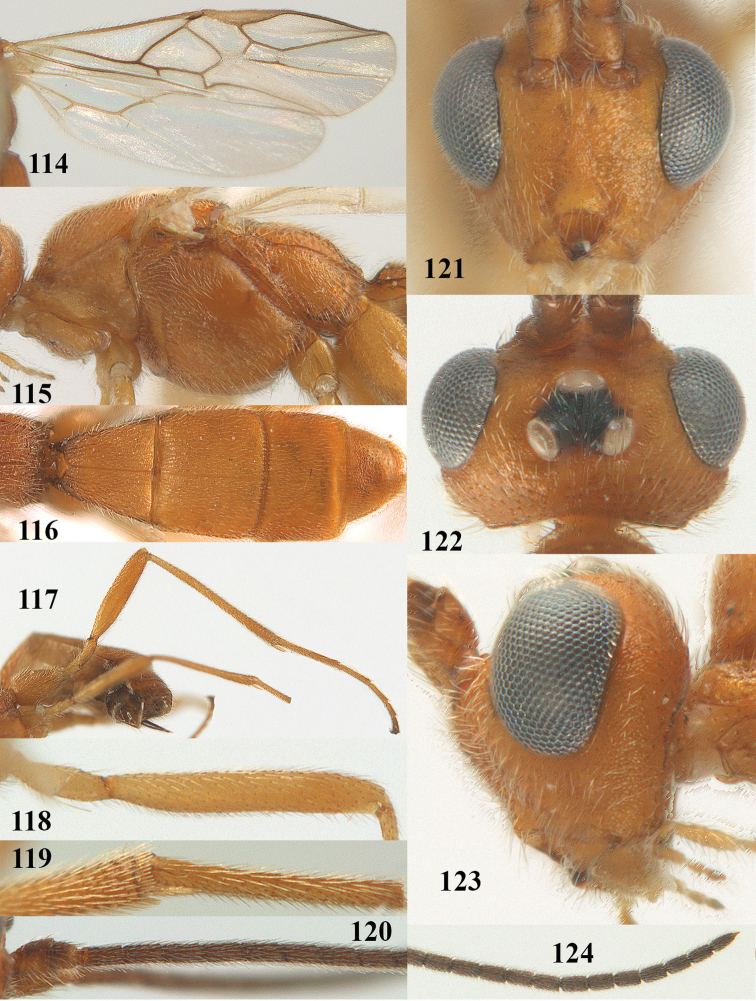
*Aleiodes carminatus* sp. n., ♀, holotype. **114** wings **115** mesosoma lateral **116** propodeum and metasoma dorsal **117** hind leg lateral **118** fore femur lateral **119** inner side of hind tibial apex lateral **120** basal segments of antenna **121** head anterior **122** head dorsal **123** head lateral **124** apical segments of antenna.

##### Description.

Holotype, ♀, length of fore wing 3.4 mm, of body 3.9 mm.

*Head.* Antennal segments of ♀ 35, length of antenna 1.2 × fore wing, its subapical segments about 1.7 × as long as wide; frons rugulose-granulate, with satin sheen; OOL and POL 1.0 and 0.8 × width of posterior ocellus, respectively; vertex granulate, rather dull and distinctly depressed near ocelli; clypeus convex and coriaceous; ventral margin of clypeus thick and depressed (Fig. 121); width of hypoclypeal depression 0.3 × minimum width of face (Fig. 121) and face coriaceous with superficial rugulae; length of eye 2.4 × temple in dorsal view and temple roundly narrowed behind eye; occiput behind stemmaticum coriaceous with satin sheen; occipital carina widely interrupted medio-dorsally and ventrally weak and irregular (Figs 122–123); clypeus partly above lower level of eyes (Fig. 121); length of malar space 0.5 × height of eye in lateral view; eyes protruding (Fig. 122).

*Mesosoma.* Mesoscutal lobes coriaceous-granulate, with satin sheen, but medio-posteriorly longitudinally rugose and anteriorly steep; notauli obsolescent; prepectal carina medium-sized, reaching anterior border; precoxal area of mesopleuron (except posteriorly) and mesopleuron antero-dorsally distinctly rugose; remainder of mesopleuron (but speculum partly smooth and shiny) granulate and dull; metapleuron largely granulate, matt; mesosternal sulcus shallow and largely smooth; mesosternum rounded posteriorly; scutellum flat, granulate, and laterally with distinct carina, lunula narrow and parallel-sided; propodeum convex, without tubercles, rugulose anteriorly and remainder rugose, median carina complete.

*Wings.* Fore wing: r 0.7 × 3-SR (Fig. 114); 1-CU1 horizontal, 0.4 × as long as 2-CU1; r-m 0.7 × 2-SR, and 0.5 × 3-SR; second submarginal cell medium-sized (Fig. 114); cu-a slightly inclivous, nearly parallel with CU1b, straight; 1-M nearly straight and 1-SR distinctly angled with 1-M. Hind wing: apical half of marginal cell slightly widened; 2-SC+R short; m-cu absent.

*Legs.* Tarsal claws with yellow setae; hind coxa rugulose and with spaced oblique rugae, with satin sheen and 0.8 × as long as first tergite; hind trochantellus 2.8 × longer ventrally than wide (Fig. 117); length of fore and hind femora 6.6 and 4.8 × their width, respectively (Figs 117–118); inner apex of hind tibia with distinct comb (Fig. 119); length of inner hind spur 0.2 × hind basitarsus.

*Metasoma.* First tergite as long as wide posteriorly, convex anteriorly and dorsal carinae lamelliform protruding basally; first and second tergites longitudinally striate, robust (Fig. 116), with distinct median carina; medio-basal area of second tergite absent; second suture narrow and crenulate; third tergite largely longitudinally rugulose, but smooth posteriorly; third tergite with complete sharp lateral crease but this absent from following tergites; ovipositor sheath ventrally densely setose and remainder smooth, shiny and apically acute.

*Colour.* Yellowish brown; antenna (except scapus and pedicellus ventrally), ovipositor sheath and most of ventral part of metasoma dark brown; stemmaticum black; tegulae, pronotum partly and legs brownish yellow; veins brown; pterostigma pale yellowish, but slightly darkened laterally; wing membrane subhyaline.

*Variation.* Antennal segments of ♀: 34(5), 35(3), 36(2), 37(2), of ♂: 35(1), 36(1), 37(8), 38(3), 39(1), 40(2). In many specimens fore wing 2-SR is strikingly longer than r-m, but in others this is less distinctive. Hind femur sometimes brown, 4.5–4.9 × as long as wide and hind trochantellus 2.6–2.9 × longer ventrally than wide; occipital carina ventrally sinuate and reduced or complete; colour of body varies from nearly completely yellowish brown to largely brown. The female from Canary Islands is the darkest specimen examined with metasoma (except medial pale patch) and hind leg largely brown.

##### Etymology.

From “carmino” (Latin for “comb”), because of the comb on the hind tibia.

##### Distribution.

*France (Corsica), *Spain (mainland, Balearic and Canary Islands).

##### Note.

Males have on average about 2–3 more antennal segments than females.

#### Aleiodes circumscriptus

Taxon classificationAnimaliaHymenopteraBraconidae

(Nees, 1834)

Figs 125–126 , 127–137

Rogas circumscriptus Nees, 1834: 216 (syntypes lost).
Aleiodes circumscriptus ; Shenefelt 1975: 1170–1171 (p.p.); Papp 1991: 113 (p.p.); Belokobylskij et al. 2003: 398.

##### Type material.

Neotype here designated, ♀ (NMS, Edinburgh), “[**Scotland**], Rowardennan, Stirlings., *Hypena proboscidalis* [on] *Urtica*, 2.ix.[19]89, mum. 17.iv.[19]90, em. 10.vi.[19]90, M.R. Shaw (♀ 2 in 1990 Expts)”.

##### Additional material.

Widespread in western Europe: *Austria, **Belgium**, **British Isles** (**England**: V.C.s 1, 3, 4, 11, 12, 14, 17, 20, 22, 23, 25, 26, 27, 28, 30, 31, 32, 33, 58, 61, 62, 63, 64; **Wales**: V.C. 52; **Scotland**: V.C.s 72, 77, 84, 86, 87, 89, 99, 111; **Ireland**: Co. Cork), **Bulgaria**, **Czech Republic**, *Finland, **Germany**, **Hungary**, **Italy**, *Lichtenstein, **Netherlands** (FL: Lelystad (Oostvaardersplassen), FR: Ried, GE: Heerde; Tongeren; Brummen (Voorstonden), LI: Kerkrade; St. Pietersberg; Tegelen; Wrakelberg, NB: Bergen op Zoom, ZH: Asperen; Waarder; Lexmond), **Norway**, **Spain**, **Slovakia**, *Sweden. Specimens in NMS, BMNH, OUM, BZL, RMNH, MTMA, ZSSM, ZISP, World Museum Liverpool, CNC, USNM, UWIM, M. Riedel collection, H. Schnee collection, MSC, JLC WAE, and I. Kakko collection.

##### Molecular data.

MRS062 (England EU979579, CO1 + KU682264, 28S), MRS073 (England KU682256, CO1), MRS074 (England KU682220, CO1).

##### Biology.

Plurivoltine parasitoid of larvae of *Hypena proboscidalis* (Linnaeus, 1758) (Erebidae: Hypeninae), overwintering in the host larva. Mummy (Fig. 15) brown and moderately slender. Specimens (in NMS unless indicated) reared from *Hypena proboscidalis* (Linnaeus) (15 [1 BMNH, 1 OUM, 1 AAC, 3 H. Schnee collection]; A.A. Allen, G.M. Haggett, A. Hawkins, R.J. Heckford, S. Ratering, M.R. Shaw). It may be an absolute specialist on *Hypena proboscidalis*; related species that similarly feed on *Urtica* (*Hypena obsitalis* (Hübner) and *Hypena obesalis* (Treitschke)) overwinter as adults and would not (by themselves) be capable of supporting the parasitoid’s annual life cycle: indeed, it has been absent from several large collections of *Hypena obesalis* made in various localities in the Alps (M.R. Shaw). The oviposition sequence (observations from two females, and *Hypena proboscidalis*) is abnormal in that there is no separate pre-oviposition sting inducing temporary paralysis, nor is there a post-oviposition period of association. The host is scarcely antennated, but quickly recognised and pounced upon or snatched with the front two pairs of legs, and held aligned with the parasitoids’s body while the ovipositor is inserted for a prolonged period – sometimes there are several insertions, with self-superparasitism then often occurring. The egg is strongly attached to internal organs (gut and malpighian tubules both observed) at its narrow end. Because of subsequent disease in the stock, it is not possible to give quantitative results, but it appeared that enthusiastic attack on fourth instar hosts resulted in oviposition but no development, and that only earlier instar hosts were suitable. Mummification takes place on the host’s food plant, usually in a semi-concealed position.

##### Diagnosis.

Antennal segments of female 42–47, of male 42–46; fore femur of ♀ 5.4–5.7 × as long as wide (Fig. 131) and hardly sculptured, but of ♂ slenderer; scapus and pedicellus (yellowish) brown ventrally; temples directly narrowed behind eyes; precoxal area frequently with some rugae or rugulae; propodeum distinctly transversally rugose medially and median carina largely absent on posterior half of propodeum or irregular; posterior half of pterostigma of female largely dark brown; ivory part of malar space usually reaching clypeus (Fig. 134); mesosternum more or less blackish or dark brown, rarely completely reddish; hind femur of ♀ rather reddish brown, but may be largely infuscate in ♂; OOL about equal to diameter of posterior ocellus (Fig. 136); vein 1-CU1 of fore wing horizontal and vein cu-a short (Fig. 127); antenna dark brown or black (but scape below usually paler than face; Fig. 134), rarely completely yellowish brown; mesosoma black(ish) dorsally, especially mesoscutum and scutellum (but notaulic area may be brownish posteriorly); metasoma largely blackish with (pale) yellowish elliptical patch medially (Fig. 129). Similar to *Aleiodes nigricornis* Wesmael, 1838, which (like many *Aleiodes circumscriptus*) has the mesoscutum usually without light markings, but *Aleiodes nigricornis* has the fore femur more slender (6.7–7.4 × as long as wide) and very finely sculptured, the scapus and the pedicellus more or less infuscate or black ventrally, the precoxal area usually without rugae, the propodeum largely coriaceous medially and the median carina at least anteriorly present on posterior half of propodeum and regular, the posterior half of the pterostigma of female more or less yellowish, but usually apical third laterally darkened, the pale yellowish part of the malar space usually not reaching the clypeus and the mesosternum usually reddish or brownish.

**Figures 125–126.**
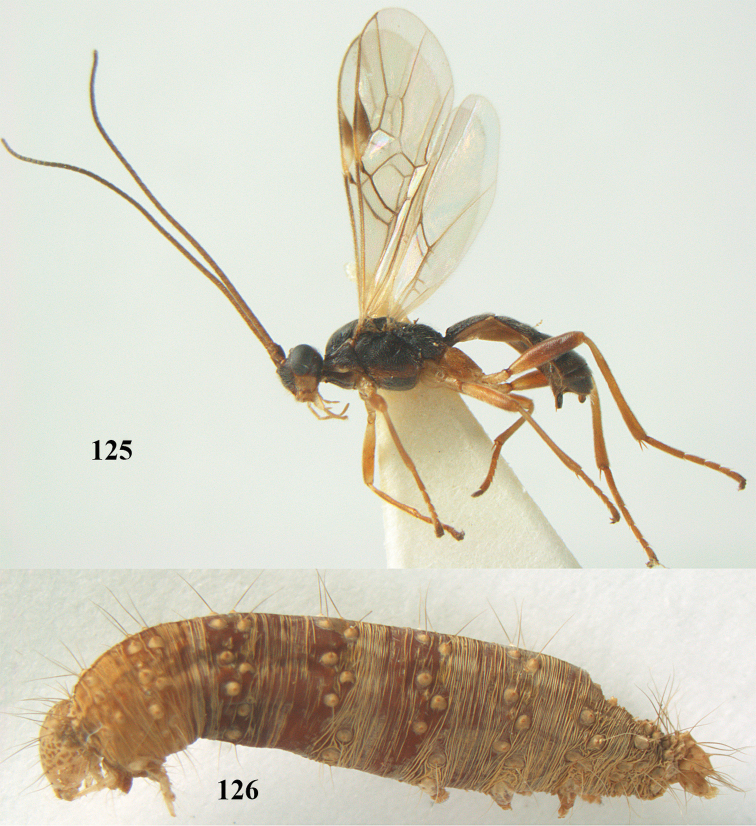
*Aleiodes circumscriptus* (Nees), ♀, neotype. **125** habitus lateral **126** mummy of *Hypena proboscidalis* (Linnaeus).

**Figures 127–137.**
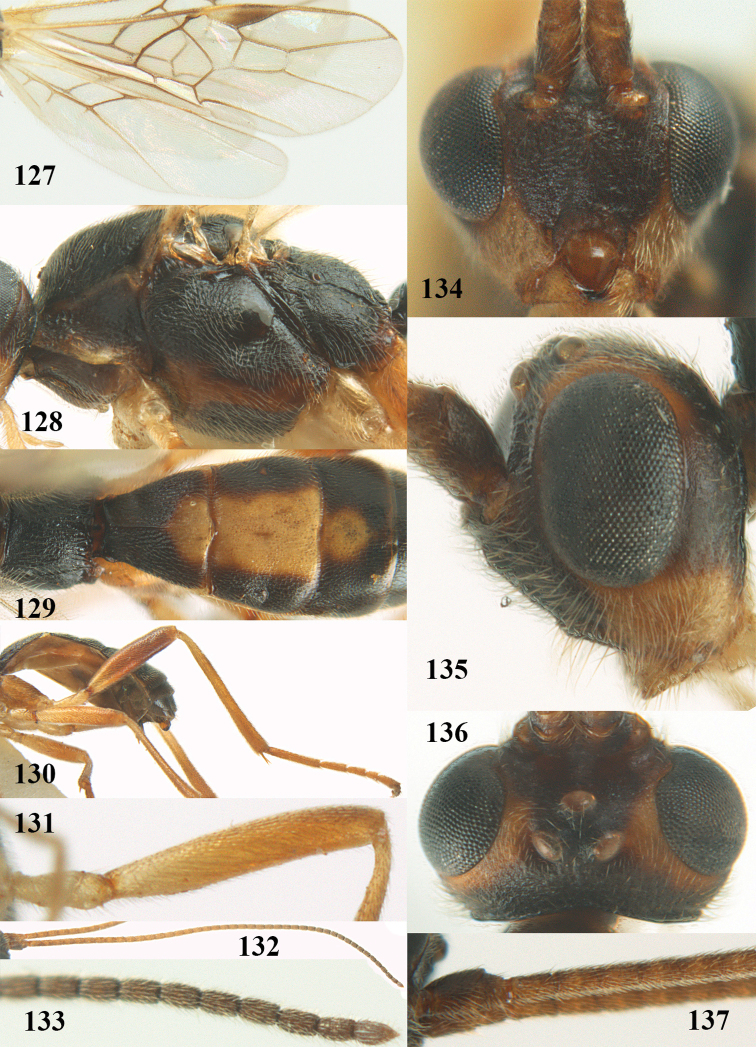
*Aleiodes circumscriptus* (Nees), ♀, neotype. **127** wings **128** mesosoma lateral **129** propodeum and metasoma dorsal **130** hind leg lateral **131** fore femur lateral **132** antenna **133** apical segments of antenna **134** head anterior **135** head lateral **136** head dorsal **137** basal segments of antenna.

##### Description.

Neotype, ♀, length of fore wing 4.9 mm, of body 5.3 mm.

*Head.* Antennal segments 46, length of antenna 1.3 × fore wing, its subapical segments about 1.6 × as long as wide; frons coriaceous and posteriorly rugulose, weakly shiny; OOL equal to diameter of posterior ocellus and coriaceous; vertex coriaceous, with satin sheen; clypeus moderately convex, coriaceous; ventral margin of clypeus thick and depressed (Fig. 134); width of hypoclypeal depression 0.4 × minimum width of face (Fig. 134) and face mainly coriaceous with some rugae dorsally; length of eye 3.2 × temple in dorsal view and temple directly narrowed behind eye; occiput behind stemmaticum coriaceous and occipital carina nearly complete, interrupted dorsally by somewhat less than width of ocellus (Fig. 136); clypeus partly above lower level of eyes (Fig. 134); length of malar space 0.4 × height of eye in lateral view; eyes moderately protruding (Fig. 136).

*Mesosoma.* Mesoscutal lobes largely coriaceous, matt, but medio-posteriorly with a few longitudinal rugae; notauli narrow, shallow and crenulate, but posterior half absent; prepectal carina lamelliform medio-ventrally, reaching anterior border; precoxal area of mesopleuron coriaceous and with some rugae medially; mesopleuron above precoxal area (except large smooth and shiny speculum) coriaceous, but dorsally rugose; medially metapleuron coriaceous, matt; mesosternal sulcus narrow and rather deep, with carina posteriorly; mesosternum rather angulate posteriorly; scutellum nearly flat, coriaceous and largely non-carinate laterally; propodeum rather flat and coriaceous but posteriorly with some rugae, median carina present but absent on posterior half, without tubercles.

*Wings.* Fore wing: r 0.2 × 3-SR (Fig. 127); 1-CU1 horizontal, 0.5 × as long as 2-CU1; r-m 0.6 × 2-SR, and 0.4 × 3-SR; second submarginal cell medium-sized (Fig. 127); cu-a vertical, not parallel with CU1b, straight; 1-M nearly straight posteriorly. Hind wing: apical half of marginal cell parallel-sided or nearly so (Fig. 127); 2-SC+R short and longitudinal; short stub of m-cu present, unpigmented.

*Legs.* Tarsal claws setose; hind coxa superficially coriaceous, with satin sheen; hind trochantellus 2.3 × longer than wide; length of fore and hind femora 5.7 and 4.3 × their width, respectively (Figs 130–131); inner apex of hind tibia without comb; length of inner hind spur 0.3 × hind basitarsus.

*Metasoma.* First tergite as long as wide posteriorly, flattened and latero-posteriorly lamelliform; first tergite coriaceous and finely irregularly longitudinally rugose; second tergite robust (Fig. 129), without distinct median carina, with satin sheen and superficially rugulose; medio-basal area of second tergite absent; second suture shallow and largely crenulate; basal half of third tergite indistinctly rugulose, remainder of metasoma largely superficially coriaceous and rather shiny; fourth and apical third of third tergite without sharp lateral crease; ovipositor sheath largely densely setose.

*Colour.* Black or brownish black; antenna brown, but scapus dorsally and laterally dark brown; palpi, malar space up to eyes, mandible, tegulae, fore and middle coxae, trochanters and trochantelli, bases of fore and middle femora, medio-apical fifth of first tergite, medially second tergite and medio-basal patch of third tergite pale yellowish (Fig. 129); orbita (except latero-ventrally) brownish yellow (Figs 134–136) and remainder of head dark brown; mesopleuron ventrally yellowish brown with darker mesosternum; hind femur (except basally) fuzzy brown (Fig. 130), remainder of legs brownish yellow; veins and pterostigma (except yellow basal 0.4 and apex) dark brown; border between dark and pale part of pterostigma fairly sharp, contrasting with each other (Fig. 127); wing membrane subhyaline.

*Variation.* Length of fore wing 4.5–5.0 mm; antennal segments of ♀: 42(1), 43(1), 44(17), 45(46), 46(28), 47(4); of ♂: 42(13), 43(30), 44(38), 45(25), 46(1); notauli absent posteriorly or shallowly impressed; mesoscutum sometimes with weak diffuse reddish colouration posteriorly, along notaulic courses; orbita sometimes completely yellowish; mesosternum varying from (frequently) almost black, and then strongly contrasting with the reddish lower third of the mesopleuron, to reddish brown; median carina of propodeum sometimes traceable to posterior margin.

##### Note.

Males have on average about one fewer antennal segment than females.

#### Aleiodes curticornis nom. n. & stat. rev.

Taxon classificationAnimaliaHymenopteraBraconidae

Figs 138 , 139–151

Aleiodes ochraceus Hellén, 1927: 24, 32 (not Rogas ochraceus Curtis, 1834); Shenefelt 1975: 1179; Papp 1985a: 154 (as possible synonym of Aleiodes gastritor (Thunberg, 1822); Koponen and Tobias 1989: 24 (lectotype deposition) (examined).
Rhogas ochraceous ; Fahringer 1932: 305.

##### Type material.

Lectotype of *Aleiodes ochraceus* Hellén here designated (FMNH), ♀, “[**Finland**,] Jomala”, “Hellén”, “829”, “Coll. Hellén: *Aleiodes ochraceus* Hellén”, “http://id.luomus.fi./GL3421”; one ♀ paralectotype (topotypic, GL3420) and one ♂ paralectotype (Nystad, GL3419).

##### Additional material.

*Austria, **Finland**, **France** (*mainland and *Corsica), *Hungary, **Italy** (*mainland and *Sicily), *Romania, *Spain, *Slovakia, *Slovenia, *Turkey. Specimens in NMS, BMNH, RMNH, MTMA, FMNH, ZSSM, FC, JLC.

##### Molecular data.

MRS056 (Corsica JF962825, CO1), MRS336 (Italy JF973341, CO1), MRS338 (Italy KU682235, CO1]), MRS342 (Italy KU682236, CO1), MRS343 (Italy JF962826/KU682237, CO1).

##### Biology.

Unknown. Most specimens have been collected at night in July and August in open situations. It may be univoltine and have hosts in low vegetation.

##### Diagnosis.

Head subglobose (Fig. 150); antennal segments of ♀ 28–35, and stout (Fig. 147), of ♂ 39–42; antenna of ♀ 0.8–1.1 × as long as fore wing, longer in ♂; OOL of ♀ 1.2 × diameter of posterior ocellus; occiput coriaceous-rugose or -rugulose (Fig. 150); second submarginal cell of fore wing rather narrow (Fig. 139); dorsal face of propodeum long and (slightly) angularly protruding postero-laterally (Fig. 142); fore femur stout (Fig. 145); hind femur rather wide (Fig. 143); sexes strongly dimorphic, the ♂ having larger ocelli, and slender and more numerous antennal segments; body completely yellowish brown, except black stemmaticum.

**Figure 138.**
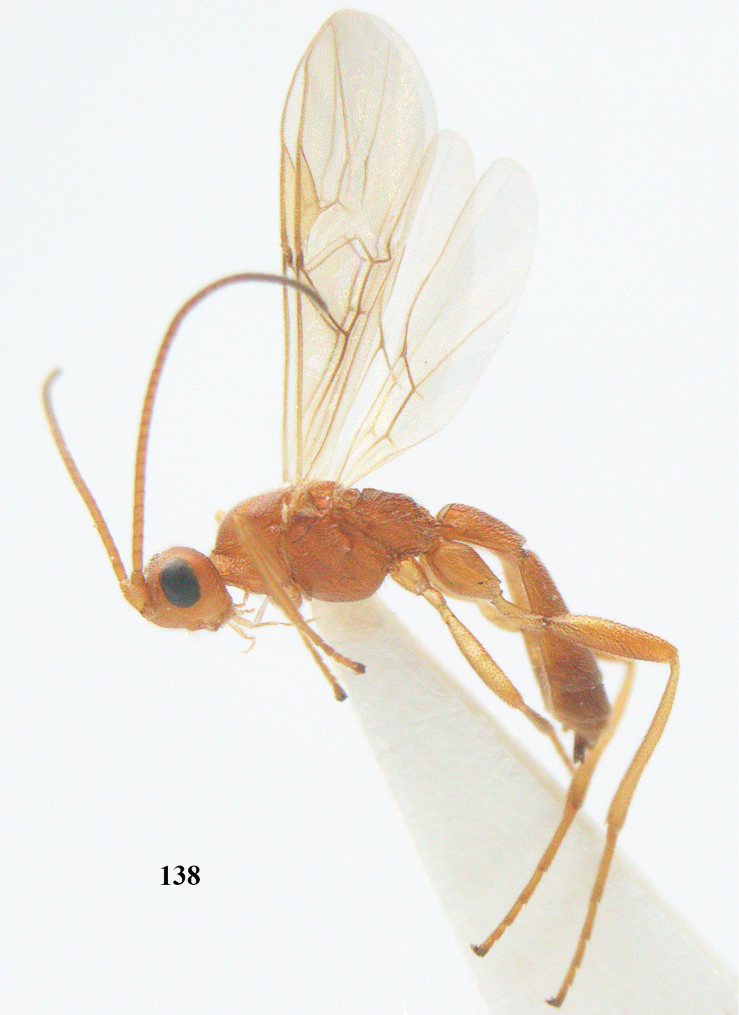
*Aleiodes curticornis* nom. n., ♀, Italy, Tyrol, near Laudes, habitus lateral.

**Figures 139–151.**
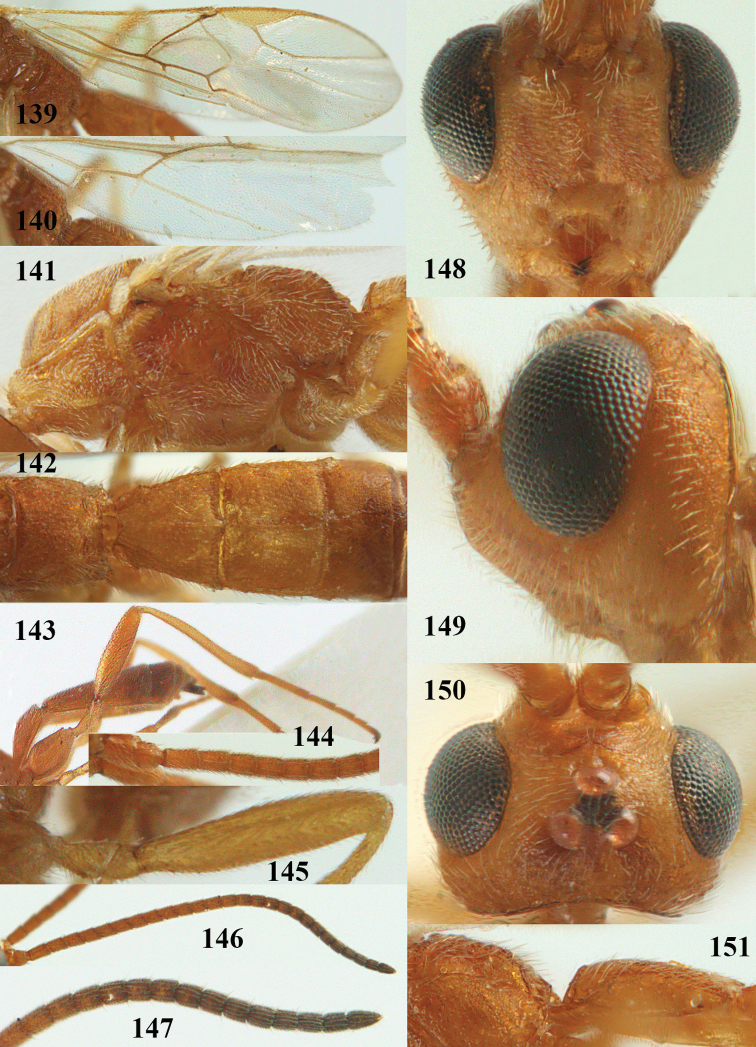
*Aleiodes curticornis* nom. n., ♀, Turkey, Agri, but 143 of ♀ from Tyrol. **139** fore wing **140** hind wing **141** mesosoma lateral **142** propodeum and metasoma dorsal **143** hind leg lateral **144** basal segments of antenna **145** fore femur lateral **146** antenna **147** apical segments of antenna **148** head anterior **149** head lateral **150** head dorsal **151** propodeum and first tergite lateral.

##### Description.

Redescribed ♀ (RMNH) from Agri (Turkey), length of fore wing 3.2 mm, of body 3.7 mm.

*Head.* Antennal segments 28, length of antenna 0.8 × as long as fore wing, its subapical segments slightly longer than wide (Fig. 147); head subglobose in dorsal view (Fig. 150); frons granulate-rugulose and slightly shiny; OOL 1.2 × diameter of posterior ocellus and very finely rugulose as is vertex, with satin sheen; clypeus moderately convex and coriaceous; ventral margin of clypeus thick and depressed (Fig. 148); width of hypoclypeal depression 0.4 × minimum width of face (Fig. 148) and face mainly transversely rugulose and with medium-sized setae; length of eye 2.2 × temple in dorsal view and temple subparallel-sided behind eye; occiput behind stemmaticum rugulose and occipital carina present ventrally and widely interrupted dorsally; clypeus near lower level of eyes (Fig. 148); length of malar space 0.5 × height of eye in lateral view (Fig. 149); eyes somewhat protruding (Figs 148–150).

*Mesosoma.* Pronotum medio-anteriorly distinctly convex; mesoscutal lobes largely coriaceous, matt and medio-posteriorly rugulose, notauli narrow and sparsely finely crenulate and posteriorly absent; prepectal carina narrow lamelliform medio-ventrally, not reaching anterior border of mesopleuron; precoxal area of mesopleuron finely rugose; mesopleuron above precoxal area (except partly smooth and shiny speculum) superficially granulate, but dorsally rugose; medially metapleuron granulate and matt; mesosternal sulcus narrow and rather shallow, without carina posteriorly; mesosternum rounded posteriorly; scutellum slender, moderately convex, mainly granulate and non-carinate laterally; dorsal face of propodeum largely rugose, long and (slightly) angularly crest-like or tuberculate protruding postero-laterally, median carina present but irregular and similar to surrounding sculpture.

*Wings.* Fore wing: r 0.5 × 3-SR (Fig. 139); 1-CU1 horizontal, 0.4 × as long as 2-CU1; r-m 0.6 × 2-SR, and 0.4 × 3-SR; second submarginal cell rather slender (Figs 138–139); vein M+CU1 of fore wing apically at same level as vein 2-CU1 (Fig. 139); vein 1-SR of fore wing short and linear with vein 1-M (Fig. 139); cu-a subvertical, not parallel with CU1b, straight; 1-M nearly straight posteriorly. Hind wing: apical half of marginal cell slightly widened apically (Fig. 140); 2-SC+R narrow and longitudinal; m-cu present, but unpigmented.

*Legs.* Tarsal claws setose; hind coxa coriaceous, with some oblique striae and satin sheen and about reaching apex of first tergite; hind trochantellus 2.4 × longer ventrally than wide; length of fore and hind femora 4.5 and 4.2 × their width, respectively (Figs 143, 145); inner apex of hind tibia without comb; length of inner hind spur 0.4 × hind basitarsus.

*Metasoma.* First tergite 0.9 × as long as wide posteriorly, rather flattened medially and latero-posteriorly non-lamelliform; first–second tergites longitudinally rugose and with median carina (Fig. 142); medio-basal area of second tergite absent; second suture narrow, deep and finely crenulate; third tergite without median carina; third–fourth tergites finely coriaceous; fourth tergite flat medially and apically truncate; fourth tergite without sharp lateral crease; remainder of metasoma largely retracted; ovipositor sheath truncate apically and moderately setose.

*Colour.* Yellowish brown; palpi, tegulae, pterostigma, veins (but parastigma and part of basal veins dark brown) and legs yellow; stemmaticum and ovipositor sheath black; wing membrane subhyaline.

*Variation.* Sexual dimorphism is unusually pronounced in this species, in respect of the large ocelli and the slenderer and much higher number of antennal segments of the male. Antennal segments of ♀ 28(2), 30(6), 31(2), 32(1), 33(1), 34(3), 35(1) and of ♂ 39(2), 40(1), 41(5), 42(1); antenna of ♀ 0.8–1.1 × as long as fore wing; stemmaticum black or brown; hind femur of ♀ moderately robust (Fig. 143) to rather swollen.

#### Aleiodes diarsianae sp. n.

Taxon classificationAnimaliaHymenopteraBraconidae

http://zoobank.org/04A7F1AC-F831-4ED3-BABC-287C8C42BBF9

Figs 152–153 , 154–164

##### Type material.

Holotype, ♀ (NMS), “[**U.K.**], **Wales**: Anglesey, Fedw Fawr, ex indet. Noctuid swept at night ex *Calluna* etc., 23.v.[19]97, mum. 29.v.[19]97, em. 16.vi.[19]97, died 1.ii.[19]98, M.R. Shaw, ♀ *Aleiodes diarsianae* in 1997 expts.”, “Host remains compatible with *Diarsia* sp., possibly *brunnea* or *mendica* det M.R. Shaw, 2013”. Paratypes (3 ♀ + 55 ♂): 42 ♂ (NMS, RMNH, BMNH) progeny of the holotype, 29 cultured in the noctuid *Diarsia mendica* (Fabricius), oviposition in range 26.vii–3.viii.1997, mummification 18–27.v.1998, emergence 25.vi–4.vii.1998 and 13 in *Diarsia rubi* (Vieweg), oviposition 23–30.viii.1997, mummification 4.iv–9.v.1998, emergence 7–25.vi.1998; 1 ♂ (NMS) **England**: Westmorland, Arnside Knott, ex Diarsia ?brunnea (Denis & Schiffermüller) on *Calluna*, coll. 5.v.1984, mum. 16.v.1984, em. 19.vi.1984, M.R. Shaw; 1 ♀ + 1♂ (NMS) **Scotland**: Orkney, ex *Diarsia brunnea* on *Calluna*, coll. v.1977, em. vi.1977, R.I. Lorimer; 1 ♀ (NMS) Scotland, East Perth, Drumderg, NO2055, ix.2012, A. Huff; 1 ♂ (NMS) Scotland, South Aberdeen, Glen Tanar 16.vii–4.viii.1986, I. MacGowan; 1 ♂ (NMS) Scotland, South Aberdeen, Braemar, Morrone Birkwood, 12.vii–6.viii.1984, B.D. Batty; 1 ♂ (NMS) Scotland, Easterness, Loch Garten, vi.1984, J.A. Owen; 3 ♂ (NMS) Scotland, Easterness, river Nethy shingle bank, NJ0214, 19.vi–5.vii.1999 (1 ♂) and 5–19.vii.1999 (2 ♂), M. Edwards); 2 ♂ (NMS) Scotland, Elgin, Bognacruie, NJ0415 19.vii–3.viii.1999 (1 ♂) and 3–23.viii.1999 (1 ♂), M. Edwards; 1 ♂ (NMS) Scotland, Elgin, Elchies, NJ2146, 27.vii–9.viii.1999, B. Hicks; 2 ♂ (NMS) Scotland, Shetland, HU335730, 15–19.vii.2004, C. Sullivan; 1 ♀ (ZSSM), **Netherlands**: Nijmegen, ex *Diarsia rubi*, Bauer; 1 ♀ (RMNH), **France**: Besse en Chande SSE, Puy de Dôme, 13.vi.1976, H. Teunissen.

##### Molecular data.

MRS030 (Wales JF962600, CO1), MRS135 (Scotland KU682257, CO1 + EU854345, 28S).

##### Biology.

Univoltine and possibly partly plurivoltine parasitoid of low feeding noctuid larvae (especially, perhaps exclusively, *Diarsia* spp.) on moorland vegetation such as *Calluna*, overwintering in the host larva. Mummy (Fig. 153) probably made in concealment, blackish and swollen. The above list of paratypes includes specimens reared in culture, with the following experimental outcomes: *Diarsia mendica* (Fabricius) 1:47\45\\30+6; *Diarsia rubi* (Vieweg) 1:47\45\\33+5. In both cases mortality was rather heavy in the overwintering young larvae, as also in control groups. In Britain a northern insect, apparently restricted to broadly moorland habitats where it is a parasitoid of low-feeding noctuid larvae, possibly exclusively in the genus *Diarsia*. When using univoltine hosts it is certainly capable of being univoltine, overwintering as a small larva within the overwintering young host, which is killed in its penultimate instar to form a characteristic swollen mummy more or less concealed near ground level (in culture, all those from *Diarsia rubi* were made on tissues lining the base of the container; in the case of *Diarsia mendica* a small proportion formed on food plant, but again in low situations). At least one suitable host (*Diarsia rubi*) is at least partly plurivoltine; it is not entirely clear how the parasitoid responds to this, but *Diarsia rubi* larvae parasitised in culture in late viii all overwintered, while about 10% of the control cohort fed up to become autumn moths, which may suggest a tendency towards univoltine constraint by the parasitoid. The adults are long-lived and females can probably persist in the field from midsummer right through the latter half of the summer; on the other hand, some of the male collection dates recorded above are late enough to suggest plurivoltinism.

**Figures 152–153.**
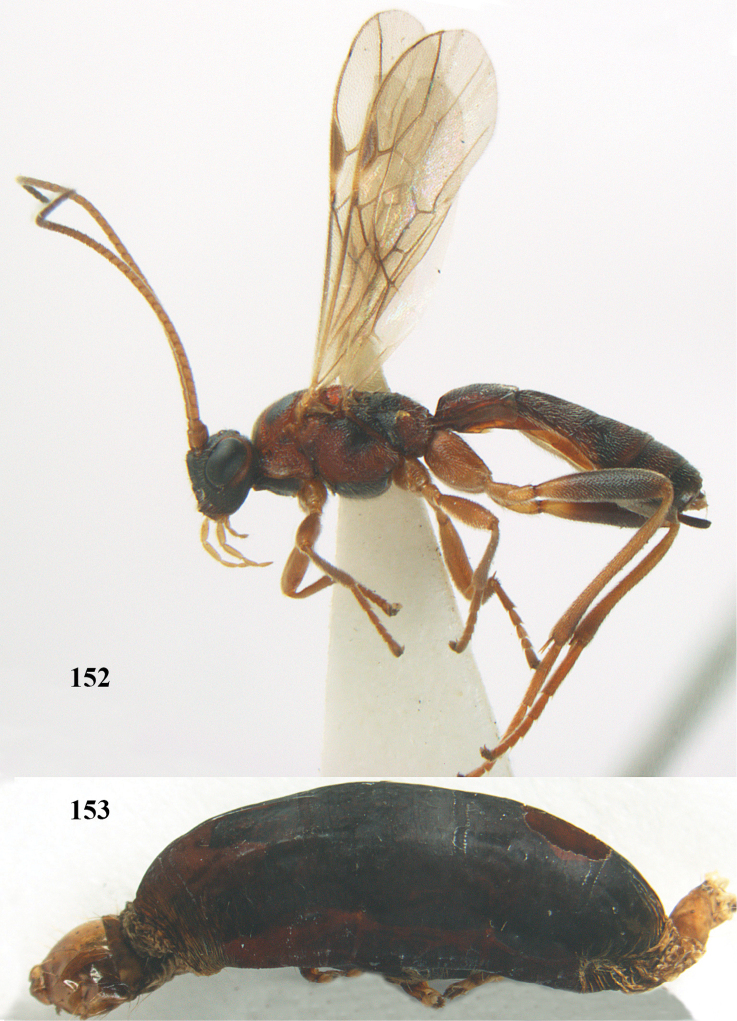
*Aleiodes diarsianae* sp. n., ♀, holotype. **152** habitus lateral **153** mummy of *Diarsia* sp.

##### Diagnosis.

Apical half of hind femur (partly) dark brown, darker than hind trochanter and trochantellus (Fig. 157); face with distinct rugae; antenna of ♀ with 36–40 segments and third segment stout, of ♂ with 40–46 segments, 4^th^–7^th^ antennal segments of both sexes stout (Fig. 164); OOL 1.4 × diameter of posterior ocellus (Fig. 163); clypeus 0.4 × as wide as face; mesosternum usually at least narrowly black posteriorly (Fig. 155); malar space and temple near eye dark reddish brown or dark brown; first tergite strongly widened apically (Fig. 156); eye elongate (of ♂) in lateral view; middle third of hind femur yellowish brown or dark brown; vertex moderately setose, rather shiny and more or less blackish or infuscate posteriorly; mesosternum variable, but dark brown in all British specimens; POL 1.2–1.7 × diameter of posterior ocellus; eye 2.2–2.5 × as long as temple in dorsal view (Fig. 163). Very similar to *Aleiodes bistrigatus* (Roman), but the latter has the temples less directly narrowed behind the eyes, 4^th^–7^th^ antennal segments less robust and fewer antennal segments in the males. Similar to the *Aleiodes pictus*-aggregate (i.e. *Aleiodes pictus* (Herrich-Schäffer, 1838) and *Aleiodes nigriceps* Wesmael, 1838) but these usually are smaller and less robust species, having the face without distinct rugae or only a few rugae dorsally, the mesosternum usually widely orange brown (and the mesopleuron usually without rugae in *Aleiodes nigriceps*), the malar space partly or completely and the temple near the eye (= external orbita) yellowish brown, the first tergite less widened apically, the fore and hind tarsi comparatively slender, the mummy slender and usually brownish (Figs 231, 273), the eye normal in lateral view and the antenna less robust. The holotype is much darker than at the start of its life (it lived in a humid tube for over 7 months); this happens to most experimental females.

**Figures 154–164.**
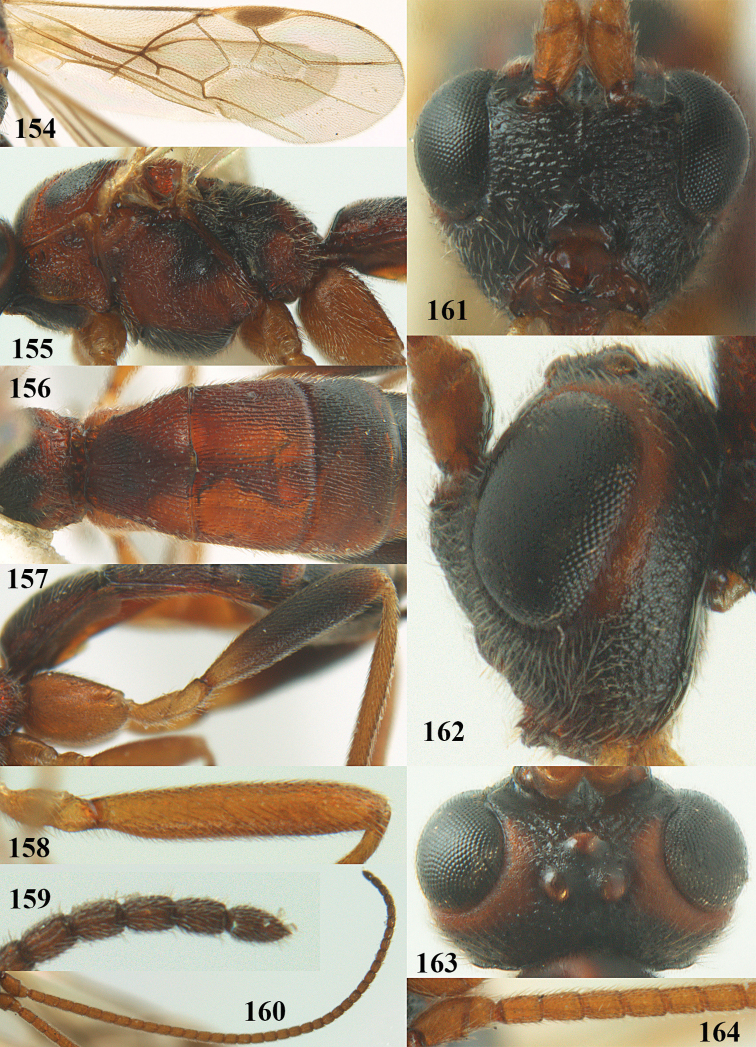
*Aleiodes diarsianae* sp. n., ♀, holotype. **154** wings **155** mesosoma lateral **156** propodeum and metasoma dorsal **157** hind leg lateral **158** fore femur lateral **159** apical segments of antenna **160** antenna **161** head anterior **162** head lateral **163** head dorsal **164** basal segments of antenna.

##### Description.

Holotype, ♀, length of fore wing 4.0 mm, of body 5.5 mm.

*Head.* Antennal segments of ♀ 40, length of antenna 1.3 × fore wing, its subapical segments about 1.5 × as long as wide and third segment stout (Figs 159–160, 164); frons coriaceous and posteriorly rugulose, with satin sheen; OOL 1.4 × diameter of posterior ocellus and coriaceous; vertex coriaceous, matt; clypeus slightly convex, indistinctly sculptured; ventral margin of clypeus rounded and depressed; width of hypoclypeal depression 0.36 × minimum width of face and face distinctly transversely rugose (Fig. 161); length of eye 2.5 × temple in dorsal view and temple rather directly narrowed behind eye (Fig. 163); occiput behind stemmaticum coriaceous and with some rugulae, occipital carina interrupted dorsally by somewhat less than width of ocellus (Fig. 163); clypeus partly above lower level of eyes and 0.4 × as wide as face (Fig. 161); length of malar space 0.5 × length of eye in lateral view; eyes moderately protruding (Figs 161–163).

*Mesosoma.* Mesoscutal lobes coriaceous, matt, but medio-posteriorly longitudinally rugose; notauli complete and moderately wide, weakly crenulate and posteriorly widened and rugose; prepectal carina medium-sized and lamelliform, reaching anterior border; precoxal area of mesopleuron largely widely rugose, mesopleuron above precoxal area (except nearly smooth and shiny speculum) largely rugose (Fig. 155); metapleuron coriaceous, matt and posteriorly rather tuberculate; mesosternal sulcus narrow and deep, absent and replaced by carina medio-posteriorly; mesosternum rounded posteriorly; scutellum elongate, slightly convex, coriaceous and laterally largely carinate; propodeum rather flat dorsally (depressed laterally and posteriorly, rather tuberculate latero-posteriorly) and strongly rugose but anteriorly weakly so, median carina complete, but posteriorly irregular.

*Wings.* Fore wing: r 0.3 × 3-SR (Fig. 154); 1-CU1 horizontal, 0.4 × as long as 2-CU1; r-m 0.65 × 2-SR, and 0.55 × 3-SR; second submarginal cell comparatively large (Figs 152, 154); cu-a weakly oblique, not parallel with CU1b, straight; 1-M slightly curved posteriorly. Hind wing: apical half of marginal cell parallel-sided or nearly so; 2-SC+R short and longitudinal; m-cu present and weakly pigmented (Fig. 154).

*Legs.* Tarsal claws setose; hind coxa coriaceous but partly superficially rugulose, largely matt; hind trochantellus 2.3 × longer than wide; length of fore and hind femora 5.1 and 4.3 × their width, respectively (Figs 157–158); inner apex of hind tibia without comb; length of inner hind spur 0.35 × hind basitarsus.

*Metasoma.* First tergite 0.8 × as long as wide posteriorly and latero-posteriorly narrowly lamelliform, moderately convex and flattened posteriorly, dorsope comparatively wide (Fig. 156); first–third tergites densely and distinctly longitudinally rugose, robust (Fig. 156), with distinct median carina; medio-basal area of second tergite absent; second suture moderately impressed and crenulate; remainder of metasoma largely superficially coriaceous; fourth and apical fifth of third tergite without sharp lateral crease; ovipositor sheath (except dorsally) densely setose.

*Colour.* Black or brownish black; antenna pale brown, but scapus dorsally and apical seventh of antenna dark brown; palpi, and tegulae pale yellowish (Fig. 152); orbita posteriorly and dorsally brownish yellow (Figs 162–163); mesosoma orange brown, but propleuron, mesoscutal lobes medially, metanotum laterally, anterior half of propodeum and metapleuron, mesopleuron dorso-posteriorly and mesosternum black; metasoma largely dark orange brown (Figs 152, 156); hind femur (except basally) fuzzy dark brown (Fig. 157) and remainder of legs yellowish brown; veins and pterostigma (except yellow basal 0.2 and apex) dark brown; border between dark and pale part of pterostigma sharp, contrasting with each other (Fig. 154); wing membrane subhyaline. This specimen had lived in a humid tube for 7 months, and its colour had deepened considerably over this time.

*Variation.* Length of fore wing 4.5–5.0 mm; antennal segments of ♀ 36(1), 38(3), 39(1), 40(1), of ♂ 40(2), 41(2), 42(4), 43(13), 44(16), 45(14), 46(3); mesosoma largely black to largely orange-brown; OOL of male slightly longer than diameter of posterior ocellus and apical half of antenna dark brown; fifth maxillary palp segment slender to moderately widened and rather long; first tergite (except medio-posteriorly) black (♂) or entirely dark reddish-brown (♀) and second tergite black or reddish laterally; in British females only posterior segments somewhat darkened; in British males first tergite more or less blackish in anterior half as well, but second and third tergites usually (almost) fully orange, sometimes with infuscation sublaterally on second tergite (especially anteriorly).; mesopleuron medially and propodeum rugose or superficially rugulose; few females seen, but in one very extensively orange specimen the legs are almost completely orange, with only slight infuscation in the apical half of hind femur. May be confused with *Aleiodes borealis* (Thomson, 1892), but this species has less antennal segments (♀: 32–34 segments), palpi and legs more or less infuscate and the clypeus wider (about 0.5 × width of the face).

We have seen 3 ♀ + 11 ♂ (NMS) from Sweden (Bohuslän and Västerbotten) and Finland (Kuusamo and Saarijärvi) that come close to *Aleiodes diarsianae*, but differ in being less robust (T1 less expanded apically; antennal segments longer in relation to width, especially basally), less strongly sculptured (fewer rugae on face; mesopleuron with only weak rugae), and having somewhat larger eyes. They also have slightly more antennal segments, at least in the female sex (the two females with intact antennae have 44 and 45 segments, the males have 42(1), 43(1), 44(1), 45(6), 47(1)), and the females have T1 more or less extensively blackish in anterior half, unlike the British *Aleiodes diarsianae* females seen, in which it is uniformly orange. CO1 sequences have been obtained for two localities (Västerbotten and Kuusamo; respectively MRS304 GenBank KU682234, and MRS692 GenBank KU682247): they form a well-isolated clade with *Aleiodes diarsianae* but differ from it by 8 fixed base-pairs. One of the Finnish males was reared from a noctuid mummy collected on a twig in a bog (N.R. Fritzén) later kindly identified from its CO1 sequence as *Coenophila subrosea* (Stephens) by Dr Katja Kramp (SDEI). Another male (in NMS) from Norway (Turtagrö, Sogn og Fjordane) has 42 antennal segments and probably belongs to the same species; it was reared from an unidentified dark noctuid mummy on *Betula nana* (K.P. Bland), which, like the Finnish one, is somewhat swollen but not as extensively so as in the considerable number of British *Aleiodes diarsianae* mummies we have seen. Both of these specimens, in common with some (but not all) males from Sweden, have the maxillary palp more swollen than seen in the British material. More material is needed to settle the status of these Fennoscandian populations, but we provisionally regard them as probably a different species near *Aleiodes diarsianae*.

##### Etymology.

Named after the generic name of its host: *Diarsia* Hübner.

##### Distribution.

*British Isles (England, Wales, Scotland), *France, *Netherlands.

##### Note.

Males have on average about 3–4 more antennal segments than females.

#### Aleiodes esenbeckii

Taxon classificationAnimaliaHymenopteraBraconidae

(Hartig, 1838)

Figs 165–166 , 167–177 , 178–179 , 180–189

Rogas esenbeckii Hartig, 1838: 255; Tobias 1986: 81 (transl.: 135) (examined).
Rhogas esenbeckii ; Kokujev (in Serebryanikova), 1901: 100.
Aleiodes esenbecki ; Papp 1991: 93 (as synonym of Aleiodes procerus).
Aleiodes esenbeckii ; Belokobylskij et al. 2003: 398.
Rhogas corsicus Szépligeti, 1906: 616 (examined).
Aleiodes corsicus ; Papp 1991: 93 (as synonym of Aleiodes procerus), 2004: 215 (holotype).
Rogas gastropachae Kokujev (in Serebryanikova), 1901: 100–101.
Aleiodes gastropachae ; Papp 1991: 93 (as synonym of Aleiodes procerus).
Phanomeris dendrolimi Matsumura, 1926: 41; Chen and He 1997: 50 (as synonym of Aleiodes esenbeckii).
Aleiodes dendrolimi ; Shenefelt 1975: 1172–1173.
Phanomeris dendrolimusi Matsumura, 1926: 32 (invalid emendation).
Phanomeris spectabilis Matsumura, 1926: 33; Chen and He 1997: 50 (as synonym of Aleiodes esenbeckii).
Rhogas metanastriae Rohwer, 1934: 47; Chen and He 1997: 50 (as synonym of Aleiodes esenbeckii).

##### Type material.

Holotype of *Rogas esenbeckii*, ♂ (ZSSM), “715, [**Germany**, Charlottenburg]”, “*esenbeckii* n.”, together with mummy of *Dendrolimus pini* (L.); holotype of *Rhogas corsicus*, ♀ (MTMA), “[**France**, Corsica,] Ajaccio”, “*praetor* Reinh.? (Corsica)”.

##### Additional material.

f. esenbeckii: **Austria**, *Croatia, **Czech Republic**, **France** (*mainland and Corsica) *Netherlands (Muiderberg), **Germany**, **Spain** (*Mallorca); f. dendrolimi: **China**, *Finland, **Russia**, **Switzerland**. Specimens in NMS, ZSSM, RMNH, BZL, MTMA, BMNH, SDEI, ZISP

##### Molecular data.

MRS180 (Finland EU979581,CO1 + EU854329, short 28S), MRS500 (Mallorca JF962845/KU682240, CO1).

##### Biology.

Apart from the examined holotype (see above) all the reared specimens we have seen (of form dendrolimi) were from Siberian populations of *Dendrolimus superans sibericus* (Rozhkov) (Lepidoptera: Lasiocampidae) (7). *Aleiodes esenbeckii* is a well-known parasitoid of *Dendrolimus* species. In the central part of its range the host species *Dendrolimus pini* (Linnaeus) is normally univoltine, but in southern European (Mediterranean) populations it is at least bivoltine (Vadim V. Zolotuhin, pers. comm.), while in northern Siberia the host *Dendrolimus superans sibericus* Tchetverikov usually has a 2-year life cycle. The parasitoid overwinters inside the diapausing host larva, and adapts its seasonality according to that of the host: Boldaruev (1958) reports that in Siberia *Aleiodes dendrolimi* spends over 20 months inside its host larva, which is mummified in late May to June following the second winter of the 2-year cycle, but in captivity (and also ocassionally in the wild) the host, and similarly the parasitoid, can be induced to develop within a year or even less. He also found that the adult parasitoid is long-lived and that females develop eggs only after a prolonged period of feeding on honeydew. Specimens seen from Europe have been collected from late May–October, no doubt reflecting local seasonality of the host.

##### Diagnosis.

Antennal segments of ♀ 56–62; head entirely brownish yellow apart from stemmaticum (Figs 173–175): f. esenbeckii) or blackish (Figs 185–187: part of f. dendrolimi); scapus in lateral view ratherly oblique apically; OOL 0.3 × diameter of posterior ocellus; occipital carina complete ventrally (Fig. 174); length of malar space 0.25 × height of eye in lateral view; vein 2-CU1 of fore wing 0.7–1.2 × vein 1-CU1 (Fig. 167); vein 1-SR angled to vein 1-M and vein 1-M distinctly curved (Fig. 167); vein r of fore wing gradually merging into vein 3-SR; tarsal claws small (Fig. 170); fourth metasomal tergite superficially coriaceous; length of fore wing 6–10 mm. Often confused with *Aleiodes varius* (as *Aleiodes procerus*), but differs by the relative lengths of veins 1- and 2-CU1 and by the number of antennal segments.

**Figures 165–166.**
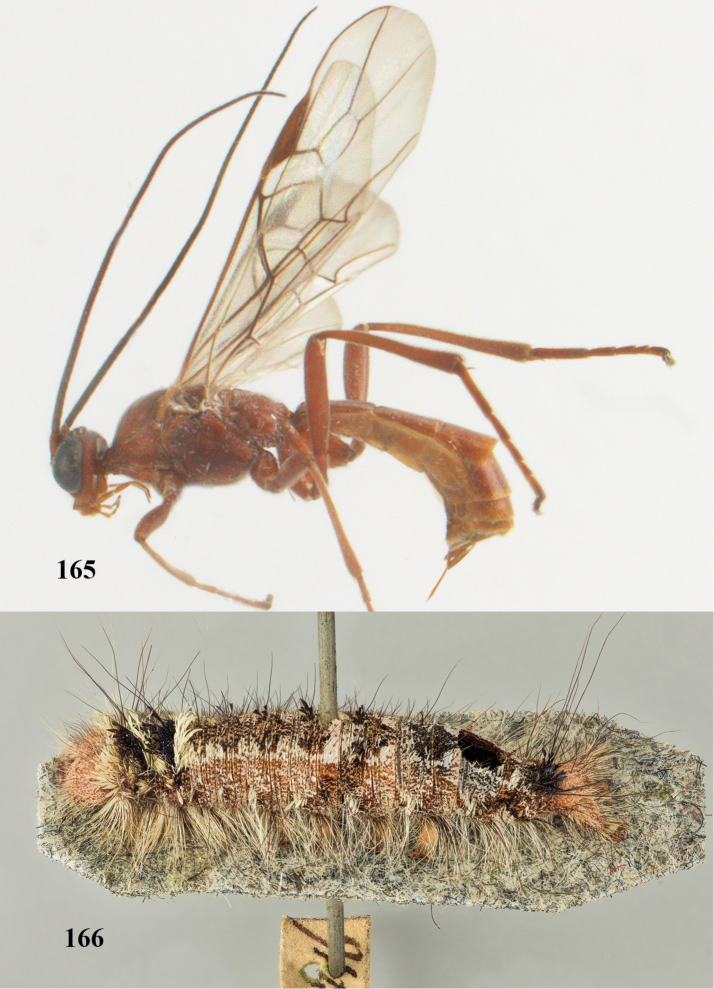
*Aleiodes esenbeckii* (Hartig), ♀, Spain, Mallorca. **165** habitus lateral **166** mummy of *Dendrolimus pini* (Linnaeus) of holotype of *Aleiodes esenbeckii* from Germany.

**Figures 167–177.**
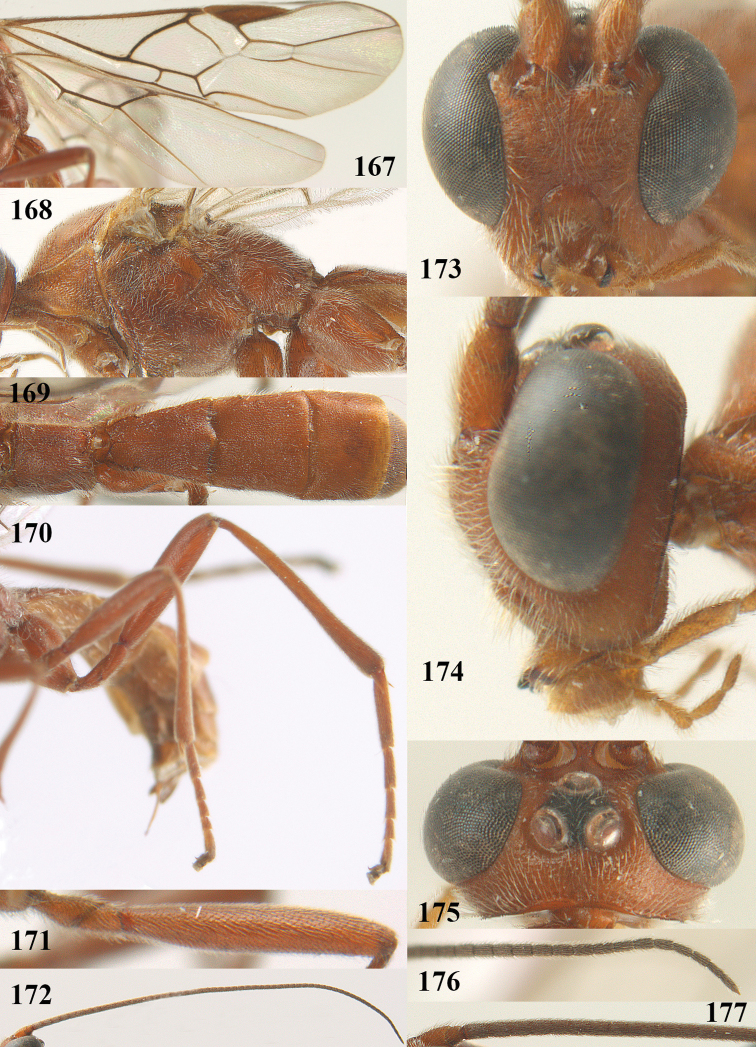
*Aleiodes esenbeckii* (Hartig), ♀, Spain, Mallorca. **167** wings **168** mesosoma lateral **169** propodeum and metasoma dorsal **170** hind leg lateral **171** fore femur lateral **172** antenna **173** head anterior **174** head lateral **175** head dorsal **176** apical segments of antenna **177** basal segments of antenna.

**Figures 178–179.**
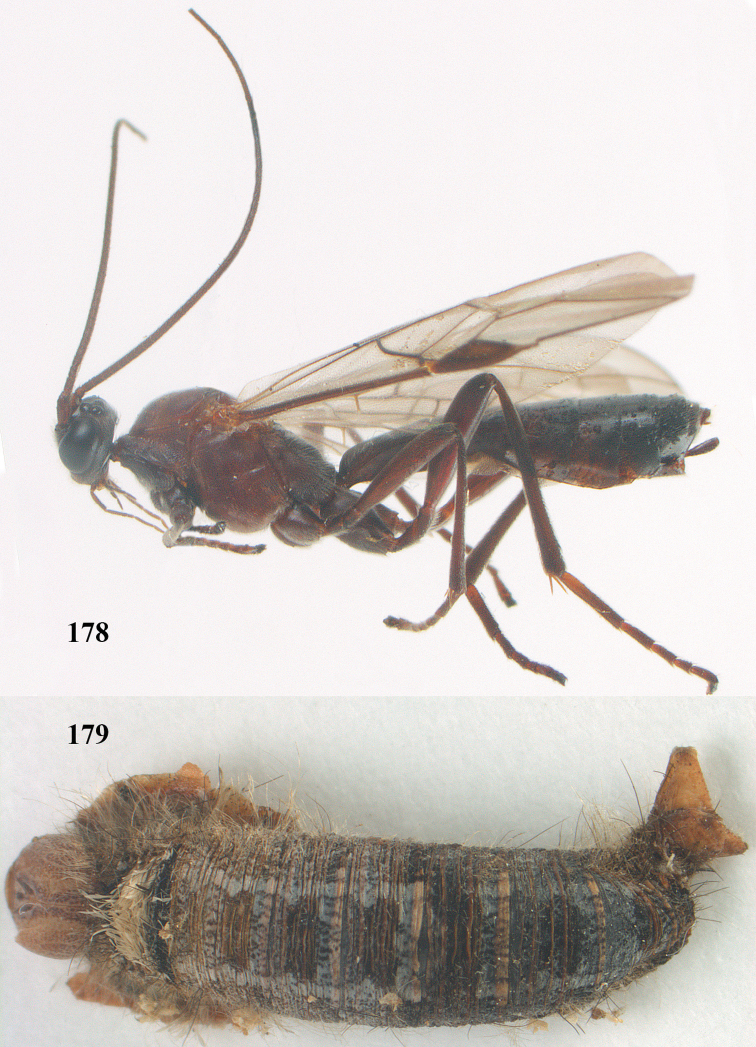
Aleiodes esenbeckii (Hartig) f. dendrolimi (Matsumura), ♀, Russia, Sakhalin. **178** habitus lateral **179** mummy of *Dendrolimus spectabilis* (Butler) from China.

**Figures 180–189.**
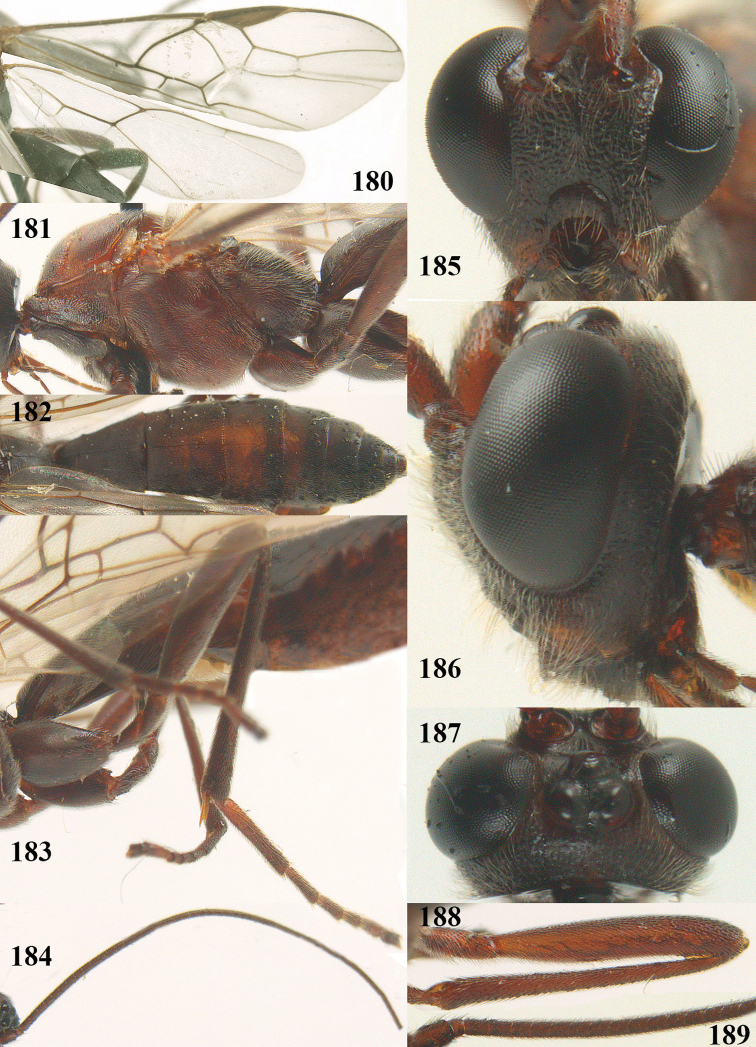
Aleiodes esenbeckii (Hartig) f. dendrolimi (Matsumura), ♀, Russia, Sakhalin. **180** wings **181** mesosoma lateral **182** propodeum and metasoma dorsal **183** hind leg lateral **184** antenna **185** head anterior **186** head lateral **187** head dorsal **188** fore femur lateral **189** basal segments of antenna.

##### Description.

Redescribed ♀ (NMS) from Mallorca (Spain), length of fore wing 7.6 mm, of body 9.5 mm.

*Head.* Antennal segments 59, length of antenna 1.3 × fore wing, its subapical segments about 1.9 × as long as wide and scapus in lateral view rather oblique apically; frons superficially granulate, rather shiny; OOL 0.3 × diameter of posterior ocellus and granulate; vertex superficially coriaceous, with satin sheen; clypeus rather high, convex dorsally and flattened ventrally, coriaceous and with long setae; ventral margin of clypeus thick and gradually depressed (Figs 173, 185); width of hypoclypeal depression 0.4 × minimum width of face (Figs 173, 185) and face mainly rugose with interspaces coriaceous; length of eye 4.7 × temple in dorsal view and temple directly narrowed behind eye; occiput behind stemmaticum finely rugose and occipital carina curved and interrupted medio-dorsally and complete ventrally (Figs 174, 186); clypeus entirely above lower level of eyes (Figs 173, 185); length of malar space 0.25 × height of eye in lateral view; eyes strongly protruding (Figs 173–175, 185–187).

*Mesosoma.* Mesoscutal lobes very finely coriaceous, with satin sheen; notauli narrow, shallow and mainly coriaceous; prepectal carina rather lamelliform medio-ventrally, almost reaching anterior border of mesopleuron and latero-ventrally curved; precoxal area of mesopleuron coriaceous, without fine rugae medially; mesopleuron above precoxal area (including shiny and granulate speculum) coriaceous, but dorsally finely rugose (cf. Fig. 181); medially metapleuron superficially granulate and shiny; mesosternal sulcus narrow and deep, without carina posteriorly; mesosternum angulate posteriorly; scutellum superficially coriaceous and carinate antero-laterally; dorsal face of propodeum rather long and coriaceous, posterior face short, hardly differentiated, with some short carinae and smooth in between, median carina complete and with weak tubercles postero-laterally.

*Wings.* Fore wing: r 0.3 × 3-SR (Fig. 167); 1-CU1 horizontal, 1.1 × as long as 2-CU1; r-m 0.9 × 2-SR, and 0.45 × 3-SR; second submarginal cell stout (Fig. 167); 1-SR rather angled to 1-M; cu-a somewhat reclivous and curved; 1-M slightly curved. Hind wing: marginal cell parallel-sided submedially and slightly widened apically (Fig. 167); 2-SC+R short and subquadrate; m-cu present as slightly pigmented vein; M+CU:1-M = 6:5; 1r-m 0.7 × 1-M.

*Legs.* Tarsal claws with fine brownish pecten basally; hind coxa finely coriaceous, with satin sheen; hind trochantellus 2.2 × longer ventrally than wide; length of fore and hind femora 5.7 and 4.5 × their width, respectively; inner apex of hind tibia without comb; length of inner hind spur 0.35 × hind basitarsus; hind basitarsus wider than following segments.

*Metasoma.* First tergite 1.3 × as long as wide posteriorly, flattened and latero-anteriorly widely lamelliform; first–second tergites densely finely irregularly rugulose and with fine median carina; second tergite as long as wide basally and 1.4 × as long as third tergite (Fig. 169); minute medio-basal area of second tergite present; second suture rather deep, widened medially and distinctly crenulate; third and following tergites superficially coriaceous and shiny; fourth tergite largely without sharp lateral crease; ovipositor sheath largely densely setose and apically truncate.

*Colour.* Yellowish brown; antenna (except yellow scapus and pedicellus) dark brown; stemmaticum black; hypopygium, middle and hind tarsi more or less infuscate; pterostigma and veins of middle third of wings dark brown (Fig. 167); other veins brownish yellow; wing membrane subhyaline.

*Variation.* Length of fore wing 6–10 mm, of body 7.5–11.5 mm; antennal segments of ♀ 58(1), 59(2), 60(3), (and of f. dendrolimi: 60(2), 61(2), 62(1), 63(1)), of ♂ 55(1), 56(2), 57(1), 58(2), 59(2), 61(1) (of f. dendrolimi: 54(1), 61(1)); latero-anterior lamella of first tergite rather wide or narrow; marginal cell of hind wing parallel-sided or slightly narrowed submedially; f. dendrolimi has head partly, palpi, mesosoma ventrally and posteriorly, metasoma and legs more or less dark brown or blackish; rarely nearly entire head black.

##### Notes.

Aleiodes esenbeckii f. dendrolimi differs morphologically only in colouration and occurs in the East Palaearctic region and in boreal Europe, perhaps reflecting a 2-year life cycle. The CO1 sequences (between Mallorcan f. esenbeckii and Finnish f. dendrolimi) are, however, divergent, differing by at least 32 base pairs in the barcode region (around 5%) (D.L.J. Quicke, pers. comm.), suggesting effective genetic isolation of at least these populations. For mediterranean specimens the name *Aleiodes corsicus* Szépligeti, 1906, is available. From limited data males appear to average about 3 fewer antennal segments than females, in both forms.

#### Aleiodes jakowlewi

Taxon classificationAnimaliaHymenopteraBraconidae

(Kokujev, 1898)

Figs 190 , 191–202

Rhogas (Aleiodes) jakowlewi Kokujev, 1898: 307.
Aleiodes jakowlewi ; Shenefelt 1975: 1176; Papp 1991: 112.
Rogas jakowlewi ; Tobias 1986: 82 (transl.: 136).

##### Material.

*Finland: 2 ♀ (NMS), Pohjois-Savo, Kangaslampi, Malaise trap, 26.vi–17.vii.2004 and 19.vii–1.viii.2005, N.M. Laurenne; 1 ♀ (I. Kakko collection) Finland, Loppi Topeno, 67410xx: 33468xx, 24.viii.2006, I. Kakko; *Sweden: 2 ♀, 1 ♂ (BMNH), Skåne, Röstanga 6.vii.1938, D.M.S & J.F. Perkins; *Slovakia: 1 ♀ (MTMA), Smolnieká Huta, 2.vii.1956, M. Čapek.

##### Molecular data.

MRS355 (Finland JF962849, CO1).

##### Biology.

Nothing is known of the biology of this predominantly boreal species.

##### Diagnosis.

**Figure 190.**
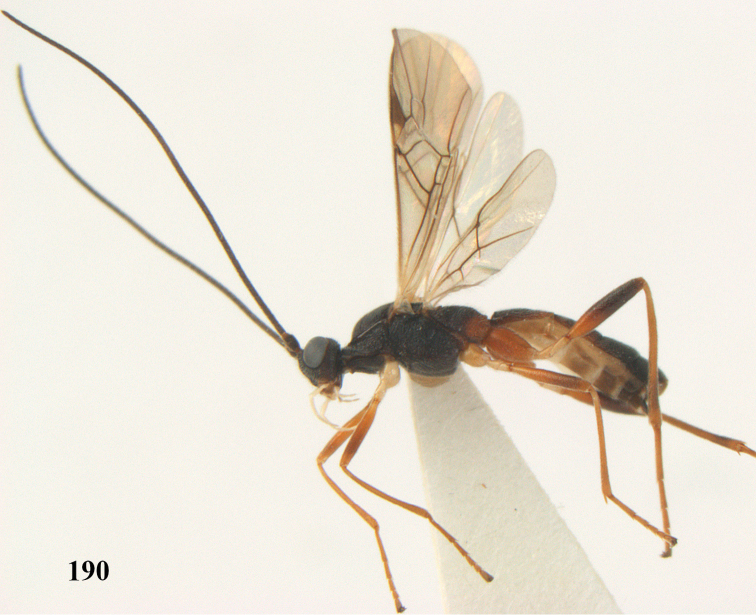
*Aleiodes jakowlewi* (Kokujev), ♀, Finland, Kangaslampi, habitus lateral.

**Figures 191–202.**
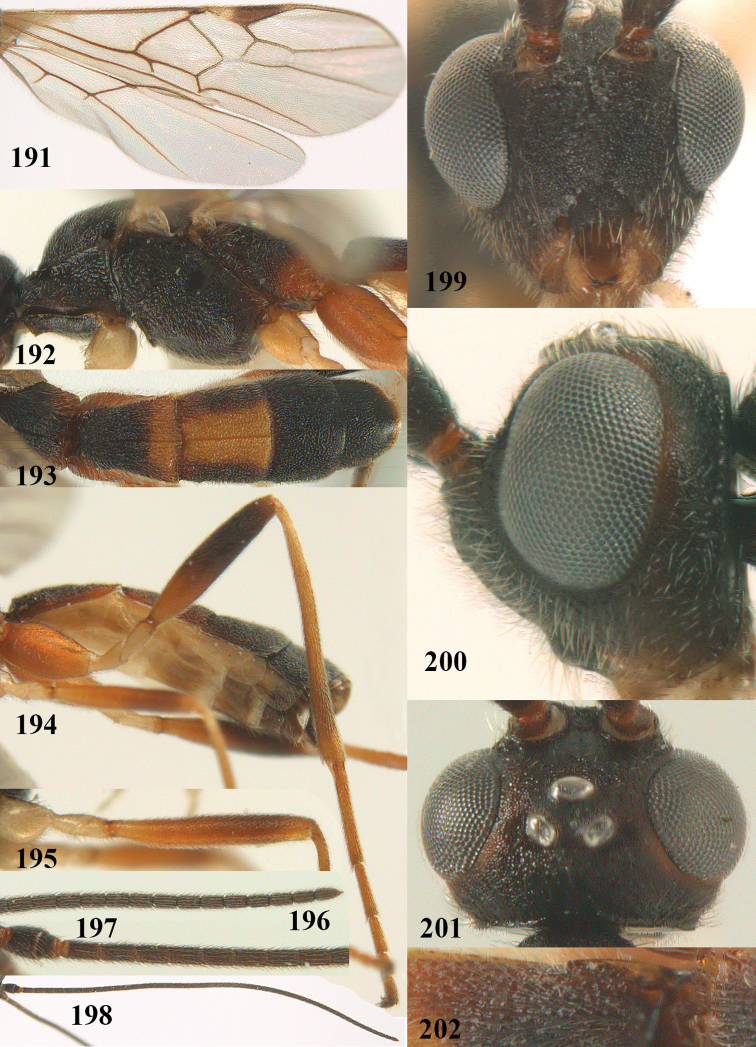
*Aleiodes jakowlewi* (Kokujev), ♀, Finland, Kangaslampi. **191** wings **192** mesosoma lateral **193** propodeum and metasoma dorsal **194** hind leg lateral **195** fore femur lateral **196** apical segments of antenna **197** basal segments of antenna **198** antenna **199** head anterior **200** head lateral **201** head dorsal **202** base of first metasomal tergite dorsal.

Head transverse in dorsal view and directly narrowed ventrally; eye rather large; antenna of ♀ sometimes with a narrow white or pale yellowish submedial band, scapus and pedicellus of ♀ similarly coloured as medial fifth of antenna; antennal segments of ♀ 49–52; OOL equal to width of posterior ocellus; length of malar space of ♀ 0.30–0.40 × (of ♂ 0.25 times) height of eye in lateral view (Fig. 200); speculum of mesopleuron rugose or reticulate and dull as remainder of mesopleuron; propodeum distinctly elongate (Fig. 190); fore wing narrow (Fig. 191); vein m-cu of fore wing straight and angled to vein 2-CU1 (Fig. 191); pterostigma dark brown with its basal third pale yellow; hind femur 4.9–5.5 × as long as its maximum width; hind trochantellus 2.2–2.7 × as long ventrally as wide (Fig. 194); hind tibia infuscate subapically, contrasting with yellowish apex of tibia (Fig. 194); tarsal claws only bristly setose, without distinct pecten; first metasomal tergite with lamella latero-anteriorly (Fig. 202), second metasomal tergite rather long; second tergite with small smooth triangular area medio-basally (Fig. 193); third tergite coarsely sculptured; fourth tergite with distinct sharp lateral crease and basally rugulose. Very similar to the East Palaearctic *Aleiodes parentalis* Belokobylskij, 2000; this species differs mainly by the wider hind femur, the subbasally yellowish first tergite (except two dark brown patches) and the more robust apical antennal segments.

##### Description.

Redescribed ♀ (NMS) from Kangaslampi (Finland), length of fore wing 4.5 mm, of body 5.5 mm.

*Head.* Antennal segments of ♀ 49, length of antenna 1.2 × fore wing, its subapical segments 1.7–1.8 × as long as wide; frons granulate, with satin sheen; OOL and POL 1.0 and 0.8 × width of posterior ocellus, respectively; vertex distinctly rugulose-granulate, with satin sheen; clypeus convex and coriaceous; ventral margin of clypeus thick and convex (Fig. 199); width of hypoclypeal depression 0.3 × minimum width of face (Fig. 199) and face mainly coarsely granulate with some rugulae; length of eye 3 × temple in dorsal view and temple directly narrowed behind eye; occiput behind stemmaticum rugulose-coriaceous; occipital carina complete, with short crenulae and dorsally curved (Figs 200–201); clypeus partly above lower level of eyes (Fig. 199); length of malar space 0.35 × height of eye in lateral view; eyes rather protruding (Figs 199–201).

*Mesosoma.* Length of mesosoma 1.8 × its height; mesoscutal lobes coriaceous, matt, but medio-posteriorly longitudinally rugose and anteriorly low; notauli narrow and crenulate; prepectal carina medium-sized, remaining separate from anterior border; precoxal area of mesopleuron and area above it largely rugose (Fig. 192); remainder of mesopleuron (including speculum) granulate and with satin sheen; metapleuron distinctly granulate and with satin sheen; mesosternal sulcus deep and sparsely crenulate; mesosternum rather angulate posteriorly; scutellum slightly convex, coriaceous, and laterally with carina; propodeum flattened, without tubercles and largely coarsely rugose, median carina complete.

*Wings.* Fore wing: r 0.5 × 3-SR (Fig. 191); 1-CU1 horizontal, 0.3 × as long as 2-CU1; r-m 0.6 × 2-SR, and 0.4 × 3-SR; second submarginal cell medium-sized (Fig. 191); cu-a inclivous, parallel with CU1b, straight (Fig. 191); 1-M straight and 1-SR angled to 1-M. Hind wing: marginal cell parallel-sided; 2-SC+R short; m-cu short and only weakly pigmented; M+CU:1-M = 15:10; 1r-m 0.7 × 1-M.

*Legs.* Tarsal claws with yellow bristles and small; hind coxa rugose-granulate, with satin sheen and 0.9 × as long as first tergite; hind trochantellus 2.7 × longer ventrally than wide; length of fore and hind femora 7.0 and 4.9 × their width, respectively (Figs 194–195); inner apex of hind tibia without distinct comb; length of inner hind spur 0.25 × hind basitarsus.

*Metasoma.* First tergite 1.2 × as long as wide posteriorly, stout, convex anteriorly and latero-anteriorly distinctly lamelliform; first–fourth tergites densely finely rugose (Fig. 193), with distinct median carina up to middle of third tergite; medio-basal area of second tergite minute; second suture medium-sized and crenulate; third and fourth tergites with complete sharp lateral crease; fifth and following tergites retracted; ovipositor sheath mainly densely setose and apically acute.

*Colour.* Dark brown; palpi, humeral plate, trochanters and trochantelli, fore and middle coxae, and ventral half of metasoma ivory or pale yellow; orbita posteriorly and tegula brown; legs (but hind femur (except basally) dark brown and fore and middle femora and hind tibia subapically infuscate), first tergite posteriorly, second tergite (except laterally) brownish yellow; ovipositor sheath black; veins and pterostigma (but basal third pale yellow) dark brown; wing membrane rather infuscate.

*Variation.* Antennal segments of ♀ 49(3), 50(1); of ♂ 48(1). In some females the antenna is distinctly white-banded (over about 23^rd^–27^th^ segments) but in others, even from the same locality, the antenna is completely brownish. The anterior ocellus is sometimes enlarged, but this too seems to be variable and is not always noticeable.

##### Note.

From limited data males appear to have fewer antennal segments than females.

#### Aleiodes leptofemur sp. n.

Taxon classificationAnimaliaHymenopteraBraconidae

http://zoobank.org/B3F2C302-894D-4DF9-89A0-3C9737CFC373

Figs 203–204 , 205–214

Aleiodes ?nigriceps ; Shaw 1983: 319, 321.
Aleiodes borealis ; Shaw 1994: 134, 136, 137.
Aleiodes borealis ; Papp and Rezbanyai-Reser 1996: 73, 96.
Aleiodes borealis ; Belokobylskij et al. 2003: 398.
Aleiodes nigriceps auctt. p.p. (not Wesmael 1838).

##### Type material.

Holotype, ♀ (NMS, Edinburgh), “[**England**:] Norfolk, Santon, ex *Stilbia anomala* on *Deschampsia flexuosa*, 18.xii.[20]01, mum. ?ii.[20]02, em. 12.v.[20]02, G. M. Haggett”, “MRS *Aleiodes* DNA 154”. Paratypes (475 ♀, 412 ♂) from **England** (West Cornwall, East Cornwall, South Devon, North Devon, Isle of Wight, North Somerset, North Wilts, South Wilts, Dorset, North Hants, South Hants, West Sussex, East Sussex, East Kent, West Kent, Surrey, South Essex, Herts, Middlesex, Berks, Oxford, Bucks, West Suffolk, East Norfolk, West Norfolk, Cambridge, Bedford, Hunts, Northampton, East Gloucester, West Gloucester, Warwick, Stafford, Leicester, Derby, Chester, South Lancaster, Mid-west York, North-west York, Westmorland), **Scotland** (Dumfries, Ayr, Lanark, Berwick, Haddington, Edinburgh, Linlithgow, Fife, Stirling, West Perth, East Perth, Elgin, Easterness, Westerness, Dunbarton, Kintyre, West Ross), **Wales** (Glamorgan, Merioneth, Caernarvon, Monmouth, Pembroke, Anglesey), **Isle of Man, Guernsey, Jersey, Andorra** (St. Julia), **Austria** (Niederösterreich: Raglitz; Poysdorf; Hainburg; Oberösterreich: Linz, Kirchschlag; Sensengebirge Bärenriedlau), **Belgium** (Mt. St. Pierre), **Bulgaria** (Rodopi: Galabovo), **Cyprus** (Yermasoyia Riv.; Yeroskipon; Cherkes; Limasol; Paramytha), **Czech Republic** (Sumava, 1000–1300m; Moravia: Mikulov), **France** (Orsay; Hautes-Alpes: Briançon), **Finland** (Kainuu: Kuhmo), **Germany** (Bonn: Rheinhöhenweg im Kottenforst; Schleswig-Holstein: Lübeck; Niedersachsen: Berkhof; Gottingen: Hann-Munden; Bonn; Rheinland, Köln-Flittard; Bavaria: Wiesen/Spessart; Lower Saxony: Harzburg; Mullingen; Alfeld; Oberhaverbeck; Hürth-Fischenich; Ahlem; Saxony: Zöbigker; Baden-Württemberg: Heidel/Jagdl; Goslar, Astfeld), **Gibraltar** (Botanical Garden), **Greece** (Lakonia, Parnon Oros, 1700 m; id., Taygetos, 1000–1200 m; Thessalia: Mt Olympos; Pisadia; Kozani). **Hungary** (Budapest: Budaliget; Budakeszi, Hársbokorhegy; Pécs, Tettye), **Ireland** (WI: Ballinclea; SK: Caragh Lake; DU: Phoenix Park; Carlow, Antrim), **Italy** (Tuscany: Gorgona Isl.; Sicily: Catania; Mt. Etna, Milo Fornazzo, 800 m; Bolzano, Sarntal; 1250 m; Südtirol: Ahrntal; Trentino: Riva s. Garda; Trentino: Tremalzo), **Netherlands** (DR: Wijster; Borger, FL: Lelystad (Oostvaardersplassen & Jagersbos), GE: Gortel; Ede; Epe; Heerde; Nunspeet; Tongeren, NB: Baarle-Nassau; Eindhoven; Geertruidenberg; Hoogerheide; Nijmegen, Berg en Dal; Vierlingsbeek; Etten-Leur; Hilvarenbeek; Rijen; Bergen op Zoom; Oss; Raamdonksveer; Tilburg (Kaaistoep), LI: Arcen; Geulle; Lemelerberg; Neercanne; Wrakelberg; Grubbenvorst; St. Pietersberg; Vilt; Wolder, NH: Weesp; Texel, Oudeschild, UT: Linschoten, ZH: Meijendel; Oostvoorne; Ouddorp; Lexmond; Melissant; Nieuwkoopse Plassen; Noordwijk; Rotterdam; Voorschoten; Waarder, FR: Ried; Terschelling, ZE: Haamstede; Westenschouwen), **Norway** (Oppland, Lom-Lia), **Slovakia** (B. Karpaty-Jaktar, Drietoma), **Spain** (Teruel, Tramacastilla; Navarra, Alsasua, 600 m; Mallorca, Porto Cristo; Zaragoza: Juslibol), **Sweden** (Skåne: Järahusen; Ystad; Böste; Spraggehusen; Ö. Väringe; Spukke; Härjedalen: Tänndalen; Duvberget; Halland: Åsa Närsbokrok), and **Switzerland** (GR: Sent Surains, Val Gronda, 1500 m). Paratypes in NMS, AAC, BMNH, BZL, M. Riedel collection, RMNH, H. Schnee collection, SYKE, MSC, ZJUH, MCZ and ZSSM.

##### Molecular data.

MRS154 (England KU682229, CO1), MRS156 (England JF962813, CO1), MRS157 (England KU682230, CO1), MRS515 (Netherlands KU682260, CO1).

##### Biology.

A parasitoid of a wide range of low feeding noctuid larvae, as listed below. Overwinters as a small larva in the host, which is killed before it is in its final instar. Mummy (Fig. 204) largely dark brown (summer generations paler) and slender, usually formed in a prominent position at least in spring. Specimens (in NMS unless indicated) reared from wild-collected Noctuidae identified as *Abrostola triplasia* (Linnaeus) (1; J.L. Gregory), *Ammoconia caecimacula* (Denis & Schiffermüller) (4:1; J. Connell/Austria), *Autographa gamma* (Linneaus) (2; G.E. King/Spain, E. Haeselbarth/Germany; 1 (RMNH), G. Peters/Germany), ?*Cerastis rubricosa* (Denis & Schiffermüller) (1; J.L. Gregory), *Cucullia chamomillae* (Denis & Schiffermüller) (2; A.A. Allen [1 is AAC]), ?*Diarsia rubi* (Vieweg) (1; T.H. Ford), *Dicestra trifolii* (Hufnagel) (1; G.M. Haggett), *Euplexia lucipara* (Linnaeus) (6:1; P. Baker), *Lacanobia oleracea* (Linnaeus) (2:1; P. Baker), *Melanchra pisi* (Linnaeus) (5; P. Baker, A. Lord, M.R. Shaw), *Mythimna ferrago* (Fabricius) (1; J. L. Gregory), ?*Mythimna impura* (Hübner) (3:1; M. R. Hall), ?*Mythimna littoralis* (Curtis) (1; F.D. Bennett), *Noctua comes* Hübner (14; J. Connell[?], D. Hackett[?], G.M. Haggett, E. Haeselbarth, R. Hinz, R.A. Softly), *Noctua fimbriata* (Schreber) (2; G.M. Haggett, R.A. Softly), *Noctua interjecta* Hübner (2; M.R. Hall, G.M. Haggett), *Noctua janthina* (Denis & Schiffermüller) (1; G.M. Haggett), *Noctua orbona* (Hufnagel) (3; G.M. Haggett), *Noctua pronuba* (Linnaeus) (5 [2 are BMNH]; R.A. Softly), *Orthosia gracilis* (Denis & Schiffermüller) (1; M.R. Shaw), *Paradiarsia glareosa* (Esper) (1; G.M. Haggett), *Phlogophora meticulosa* (Linnaeus) (10; M.R. Shaw, R.A. Softly, J. Voogd), *Shargacucullia verbasci* (Linnaeus) (24 [5 are OUM, 1 is AAC]; A.A. Allen, F.C. Woodforde, M.R. Shaw), *Standfussiana lucernea* (Linnaeus) (1; R.F. Logan), *Stilbia anomala* (Haworth) (4; G.M. Haggett), *Xestia agathina* (Duponchel) (12 [6 are ZSSM, 2 are AAC]; A.A. Allen, E. Bauer, A. Dobson, M.R. Shaw), *Xestia baja* (Denis & Schiffermüller) (1; J.L. Gregory), *Xestia castanea* (Esper) (8 [5 are ZSSM]; E. Bauer, K.P. Bland[?], M.R. Shaw), *Xestia xanthographa* (Denis & Schiffermüller) (31 [1 is ZSSM, 1 is RMNH]; E. Bauer, M.R. Britten, J. Connell, G.M. Haggett, M.R. Hall, N. Hall, R. Hinz, M.R. Shaw, R.A. Softly), and unidentified noctuids (81 [14 are BMNH], mostly collected as mummies). Specimens in NMS reared in culture experiments are included in the type material (but not in the above host list), and these experimental results (using females from the overwinter generation to parasitize summer hosts) are as follows: *Diarsia rubi* 1:16\16\\7+4 [several others were retarded but died]; *Dicestra trifolii* 5:28\28\\24+4; *Lacanobia oleracea* 6:28\22\\0+16 [several others were retarded but died]; *Melanchra persicariae* (Linnaeus) 4:22\13[several others pricked and paralysed without oviposition]\\0+13; *Orthosia cerasi* (Fabricius) 7:55\6\\5+1; *Orthosia gothica* (Linneaus) 6:40\1\\0+1; *Orthosia gracilis* 8:27\16\\14+2; *Orthosia incerta* (Hufnagel) 6:23\4\\2+1; *Phlogophora meticulosa* 9:49\44\\29+14: *Shargacucullia verbasci* 1:1\1\\1+0. In Britain adult flight times peak around May, July–August and October. However, in common with most *Aleiodes*, the adults are very long-lived and can be found in most months (but we have no British records from January to March). Both sexes come feely to light, but it is also active by day. The males tend to court non-conspecific females (at least of some species) with as much – though unsuccessful – vigour as with conspecifics. All host records are from Noctuidae feeding on low plants, but within that group this species has an unusually broad host range. Many (but not all) of the overwintering hosts, such as *Noctua* and *Xestia* species (parasitized from September to November), feed on Poaceae and grow slowly during mild periods in the winter, with mummification by the parasitoid in the host’s 3^rd^ or 4^th^ instar sometimes as early as February, but more often during March or April. The resulting adults again parasitize low-feeding noctuids, with a similarly broad range of hosts, but mostly on plants other than Poaceae. Adults of both sexes are often swept from trees and bushes (especially in late summer and autumn) but they are probably merely feeding on honeydew rather than seeking hosts: although some *Noctua* and *Xestia* species that feed through the winter on grasses do sometimes oviposit on tree leaves, with the resulting larvae feeding thereon until (easily!) displaced, their falling to the field layer usually happens in the first instar (MRS, personal observation) when they are probably still too small to be easily parasitized by *Aleiodes leptofemur*. It is rather remarkable that the host larvae, especially of the overwintering generation, regularly (perhaps invariably) climb out of their normal living space, to be mummified fully exposed high on stems of various kinds (very often on dead grass seed heads), on tree trunks, fence posts etc. Related common species such as *Aleiodes nigriceps*, *Aleiodes pictus* and *Aleiodes similis* (Curtis, 1834) [the latter to be treated in a subsequent part of this revision], which (at least overwinter) parasitize ecologically similar and closely related – or in many cases the same – noctuid species, do not cause their hosts to do this, but instead the hosts parasitized by these species seek concealment before mummification. Consequently, mummies of this group found exposed in nature are almost invariably those of *Aleiodes leptofemur*. It is an obvious suggestion that this helps the parasitoid to avoid idiobiont parasitism (pseudohyperparasitism) in the dangerous field layer, including by virtue of faster development to the relative safety of the adult stage (the spring-forming mummies are very dark and presumably absorb insolation energy well), but it does leave open the question why the other (related, and similarly plurivoltine) species mentioned above have not adopted the same habit.

**Figures 203–204.**
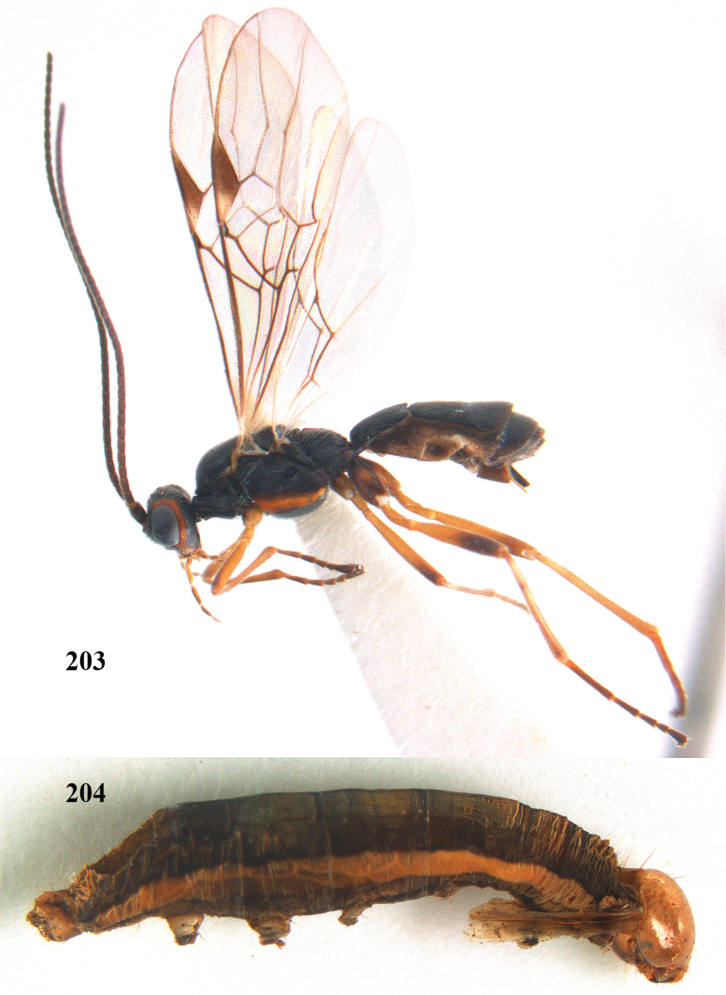
*Aleiodes leptofemur* sp. n., ♀, holotype. **203** habitus lateral **204** mummy of *Stilbia anomala* (Haworth).

##### Diagnosis.

**Figures 205–214.**
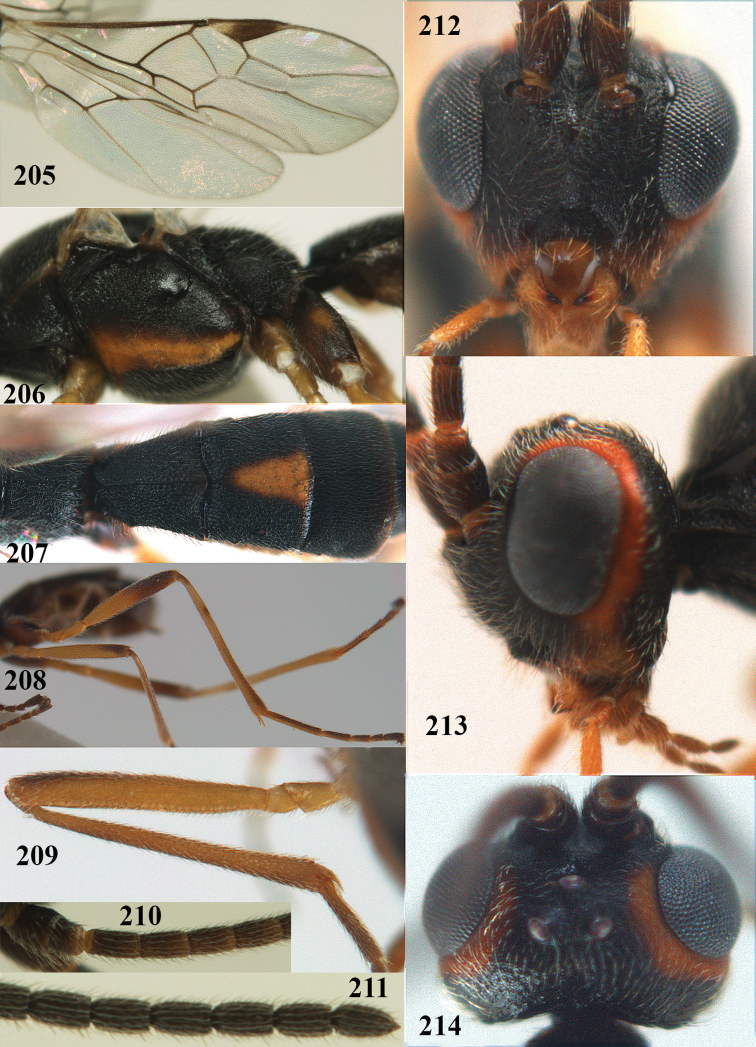
*Aleiodes leptofemur* sp. n., ♀, holotype. **205** wings **206** mesosoma lateral **207** propodeum and metasoma dorsal **208** hind leg lateral **209** fore femur lateral **210** basal segments of antenna **211** apical segments of antenna **212** head anterior **213** head lateral **214** head dorsal.

Length of fore femur 6.4–8.0 × its maximum width (Fig. 209) and hind femur parallel-sided (Fig. 208); mesosternum usually black(ish); face with some weak transverse rugae dorsally; OOL 1.5 × diameter of posterior ocellus; temple roundly narrowed (Fig. 214); scapus ventrally and usually basal half of antenna (dark) brown, rarely yellowish; hind femur slender, basally largely yellowish and frequently infuscate subapically, but remaining nearly always paler than ventral side of scapus; if rarely hind femur is distinctly infuscate (Fig. 208) then often also hind coxa (at least basally) and base of hind tibia infuscate (Fig. 203); face usually black or dark brown medially and near eyes yellowish brown; antennal segments of ♂ 35–40, usually 36–38, less than of ♀, which has usually 37–39 segments; pterostigma tending to be dark brown medially (Fig. 205). Similar to *Aleiodes borealis* (Thomson, 1892) and to species of the *Aleiodes pictus* aggregate. They differ by having the length of the fore femur 5.4–6.4(–7.3) × its maximum width and the hind femur more or less weakly swollen; if more than 6.4 × then the face without transverse rugae dorsally, the hind femur comparatively wide basally, the mesosternum yellowish or the temple comparatively wide, or the scapus ventrally and the basal half of the antenna yellowish brown; if the scapus is dark brown or blackish then the scapus is similarly coloured as the hind femur subapically; colour of the hind femur variable, usually dark brown or blackish subapically; face usually completely black or rarely yellowish; antennal segments of male 37–45, averages about one segment more than of female, which has 36–45 segments (32–34 in *Aleiodes borealis*, of which we have not seen a male with complete antenna); pterostigma is variable, but often yellowish medially; clypeus distinctly transverse and less depressed ventrally, and hind femur rather micro-sculptured. Some dark males of *Aleiodes similis* are very like pale males of *Aleiodes leptofemur* and in extreme cases scarcely separable. The new species shares with the East Palaearctic *Aleiodes angustatus* (Papp, 1971) the elongate and paralle-sided fore and hind femora. *Aleiodes angustatus* has the body entirely yellowish brown, the ocelli larger (POL slightly less than the diameter of the posterior ocellus and OOL about 1.2 times diameter of ocellus), the antenna of ♀ with 47–49 (♂: 46–49) segments, the second metasomal tergite nearly parallel-sided, the precoxal sulcus witrh distinct rugae and the pterostigma yellow.

##### Description.

Holotype, ♀, length of fore wing 4.5 mm, of body 4.4 mm.

*Head.* Antennal segments 39, length of antenna 1.1 × fore wing, its subapical segments about 1.8 × as long as wide (Fig. 211) and basal segments comparatively wide (Fig. 210); frons granulate-coriaceous, with satin sheen; OOL 1.5 × diameter of posterior ocellus and coriaceous; vertex granulate-coriaceous, with satin sheen; clypeus distinctly convex (Fig. 213), coriaceous; ventral margin of clypeus rounded and depressed (Fig. 212); width of hypoclypeal depression 0.4 × minimum width of face and face coriaceous, dorsally somewhat rugulose (Fig. 212); length of eye 2.5 × temple in dorsal view and temple gradually roundly narrowed behind eye (Fig. 214); occiput behind stemmaticum granulate-coriaceous, occipital carina interrupted by somewhat more than width of ocellus (Fig. 214); clypeus partly above lower level of eyes (Fig. 212); length of malar space 0.4 × length of eye in lateral view; eyes moderately protruding (Figs 212–214).

*Mesosoma.* Mesoscutal lobes finely coriaceous, with satin sheen, medio-posteriorly with a few rugulae; notauli complete and narrow, largely smooth and posteriorly reduced; prepectal carina narrow lamelliform, reaching anterior border; precoxal area of mesopleuron granulate-coriaceous, mesopleuron with superficially granulate and shiny speculum and rugose dorso-anteriorly (Fig. 206); metapleuron granulate-coriaceous, matt and posteriorly not tuberculate; mesosternal sulcus narrow and rather deep, shallow medio-posteriorly and no carina; mesosternum angulate posteriorly; scutellum elongate, slightly convex, granulate-coriaceous and laterally largely without carina; propodeum rather flat dorsally, not tuberculate latero-posteriorly, and coriaceous with median carina complete.

*Wings.* Fore wing: r 0.3 × 3-SR (Fig. 205); 1-CU1 horizontal, 0.4 × as long as 2-CU1; r-m 0.6 × 2-SR, and 0.35 × 3-SR; second submarginal cell comparatively large (Fig. 205); cu-a weakly oblique, not parallel with CU1b, straight; 1-M nearly straight posteriorly. Hind wing: apically marginal cell nearly twice as wide as its minimum width; 2-SC+R short and longitudinal; m-cu short and not pigmented (Fig. 205).

*Legs.* Tarsal claws setose; hind coxa finely coriaceous, largely matt; hind trochantellus 2.4 × longer than wide; length of fore and hind femora 6.9 and 6.9 × their width, respectively (Figs 208–209); inner apex of hind tibia without comb; length of inner hind spur 0.3 × hind basitarsus.

*Metasoma.* First tergite 1.1 × as long as wide posteriorly and latero-posteriorly narrowly lamelliform, moderately convex and flattened posteriorly, dorsope medium-sized (Fig. 207); first and second tergites densely and longitudinally rugulose (Fig. 207), with distinct median carina; medio-basal area of second tergite absent; second suture narrow and finely crenulate; third tergite coriaceous and remainder of metasoma largely smooth and shiny; fourth and apical half of third tergite without sharp lateral crease; ovipositor sheath (except dorsally) moderately setose.

*Colour.* Black; antenna dark brown; palpi largely brown; tegulae, malar space ventrally and triangular patch on second tergite pale yellowish (Fig. 207); inner orbita as dark as face centrally; outer orbita posteriorly and dorsally, malar space dorsally and mesoscutum medio-posteriorly brownish yellow (Fig. 213); mesopleuron with ventral brownish yellow stripe (Fig. 206); hind coxa largely, apical half of hind femur, base of hind tibia and tarsi rather fuzzy dark brown (Figs 203, 208) and remainder of legs yellowish brown; veins and pterostigma (except yellow basal 0.3 and slightly apex) dark brown; border between dark and pale part of pterostigma rather sharp, contrasting with each other (Fig. 205); wing membrane subhyaline.

*Variation.* Length of fore wing 4.5–5.0 mm; antennal segments of ♀ 35(3), 36(16), 37(79), 38(98), 39(70), 40(20), 41(1), 42(1), of ♂ 35(22), 36(64), 37(89), 38(60), 39(17), 40(4), 41(1)); mesosoma largely black to largely orange brown; medial length of second tergite 0.8–0.9 × its basal width; OOL of male slightly longer than diameter of posterior ocellus and apical half of antenna dark brown; mesopleuron medially and propodeum rugose or superficially rugulose. Specimens of the summer generation(s) are usually overall paler than those from the overwinter generation. The face usually dark centrally with the inner orbits paler but sometimes face completely black (as in the type, from the overwinter generation), less often completely orange or darkened only near clypeus (males more likely than females to exhibit these extremes). Extent of orange markings on mesoscutum extremely variable, but almost always distinct; metasoma only rarely wholly black or dark brown. Colour of pterostigma very variable, sometimes pale greyish and only faintly darker near posterior margin.

The broad host range, which has (at least in part) been experimentally verified, may contribute to the variability of this species. We have seen a large number of summer-generation female specimens from S. Europe (Portugal, Greece, Turkey and most notably a long series from South Bulgaria from Rodopi in BZL) that consistently differ in colour from summer specimens from Britain in the combination of a slightly darker pterostigma, uniformly pale legs, and the metasomal tergites posterior to the central pale area tending to be reddish brown rather than blackish, and they are also slightly smaller. Because of its relative uniformity in contrast to the variability of what we otherwise regard as *Aleiodes leptofemur*, it seem possible that this material represents a different species and we have not included it in the type series.

##### Etymology.

This common and widely distributed species is named after its slender femora (“leptos” = Greek for “thin”).

##### Distribution.

*Andorra, *Austria, *Belgium, *British Isles (England, Wales, Isle of Man, Scotland, Ireland, Guernsey, Jersey), *Bulgaria, *Cyprus, *Czech Republic, *France, *Finland, *Germany, *Gibraltar (British territory), *Greece, *Hungary, *Italy, *Netherlands, *Norway, *Slovakia, *Spain, *Sweden, *Switzerland. The southern European countries are included provisionally (see above under variation).

##### Notes.

Males have on average about one fewer antennal segments than females. Both authors have left determination labels for this species incorrectly as *Aleiodes borealis* (Thomson) on a large number of specimens in many collections (up until about 2006 for CvA; until 2007 for MRS), which are now impossible to correct.

#### Aleiodes modestus

Taxon classificationAnimaliaHymenopteraBraconidae

(Reinhard, 1863)

Figs 215–216 , 217–229

Rogas modestus Reinhard, 1863: 271; Shenefelt 1975: 1177; Papp 1985a: 160 (lectotype designation); Tobias 1986: 83 (transl.: 138).
Aleiodes modestus ; Papp 1991: 99; Belokobylskij et al. 2003: 398.
Rhogas (Aleiodes) modestus var. piceus Fahringer, 1932: 302–303; Shenefelt 1975: 1177.

##### Type material.

Lectotype ♀ (ZMB) from Germany examined.

##### Additional material.

Widespread in western Europe: *Austria, **British Isles** (**England**: V.C.s 11, 17, 22, 28, 29, 31, 32, 38, 39, 52, 57, 58, 61, 62, 63, 64; **Wales**: V.C.s 48, 52: **Scotland**: V.C.s 88, 96, 97), **Bulgaria**, *Czech Republic, *Denmark, **Finland**, *France, **Germany**, **Italy**, **Netherlands** (DR: Borger; Wijster, GE: Heerde; Brummen (Voorstonden), FR: Terschelling (Midsland-Noord, dunes), LI: Epen, ZH: Asperen; Meijendel (dunes)), **Poland**, *Romania, **Russia**, **Slovakia**, **Sweden**, **Switzerland**. Specimens in NMS, MNHN, BMNH, RMNH, NRSM, BZL, MTMA, USNM, CNC, CMIM, M. Riedel coll., JLC, Delémont, ZSM, MCZ, SDEI, FRAH, WAE, UWIM, ZMC, ZMUO.

##### Molecular data.

MRS282 (Wales JF962850, CO1 + KU682267, 28S).

##### Biology.

A univoltine parasitoid of a wide range of *Eupithecia* species (Geometridae: Larentiinae), mummifying the host in its pupation chamber and overwintering in the mummy. Specimens reared from wild collected hosts determined as follows: *Eupithecia absinthiata* (Clerck) (2 NRS), *Eupithecia exiguata* (Hübner) (1 ZMUH), *Eupithecia gelidata hyperboreata* Staudinger (2 SDEI), *Eupithecia goossensiata* Mabille (2 NMS, 1 OUM; T.H. Ford), *Eupithecia innotata* (Hufnagel) (2 ZMUH, 1 ZMUO); I. Itämes), *Eupithecia lariciata* (Freyer) (1 NMS, 2 Copenhagen, 2 Delémont, 10 FRAH), *Eupithecia nanata* (Hübner) (22 NMS, 5 BMNH, 5 OUM]; T.H. Ford, G.T. Lyle), *Eupithecia satyrata* (Hübner) (1 NRS), *Eupithecia subfuscata* (Haworth) (3 OUM; T.H. Ford), *Eupithecia succenturiata* (Linnaeus) (1 NMS, 1 OUM; T.H. Ford), *Eupithecia vulgata* (Haworth) (6 NMS, 2 OUM; T.H. Ford, M.R. Shaw) and 13 (NMS) from undetermined *Eupithecia* spp on various low plants (including *Artemisia*, *Lotus* and *Pimpinella*). Experimental culture result in *Eupithecia vulgata* 2:16\16\\7+1. While it has been reared particularly from hosts feeding on field layer plants (as is the habit of the majority of European *Eupithecia* species), the rearing records also include a substantial number from *Eupithecia lariciata* on *Larix*; however, it seems probable that many of the specimens seen were reared in a nursery context, in which young *Larix* would have presented as part of the field layer, and it is perhaps significant that *Aleiodes modestus* was not found to be a prominent parasitoid of *Eupithecia lariciata* on mature *Larix* sampled in the Alps (although it was indeed reared from it in small numbers at most sampling sites: Kenis et al. 2005). The host larva is usually fully grown and cocooned in the soil as a prepupa by the time mummification occurs, and the wide and sharp-rimmed clypeus of the adult probably reflects the need to chew its way through the host’s cocoon and soil. The winter is passed in the mummy, which is rather tough and dark, slightly dorso-ventrally flattened but not keeled (Fig. 216), and sometimes weakly upcurled. Typically the thoracic and first two abdominal segments are contracted, with the parasitoid occupying approximately abdominal segments 3–8 in a thin silken lining. The mummy is not stuck down, though probably the usual ventral opening for the expulsion of fluid occurs none the less (however, this is unconfirmed). The adult flight time in Britain is approximately from late June through August.

**Figures 215–216.**
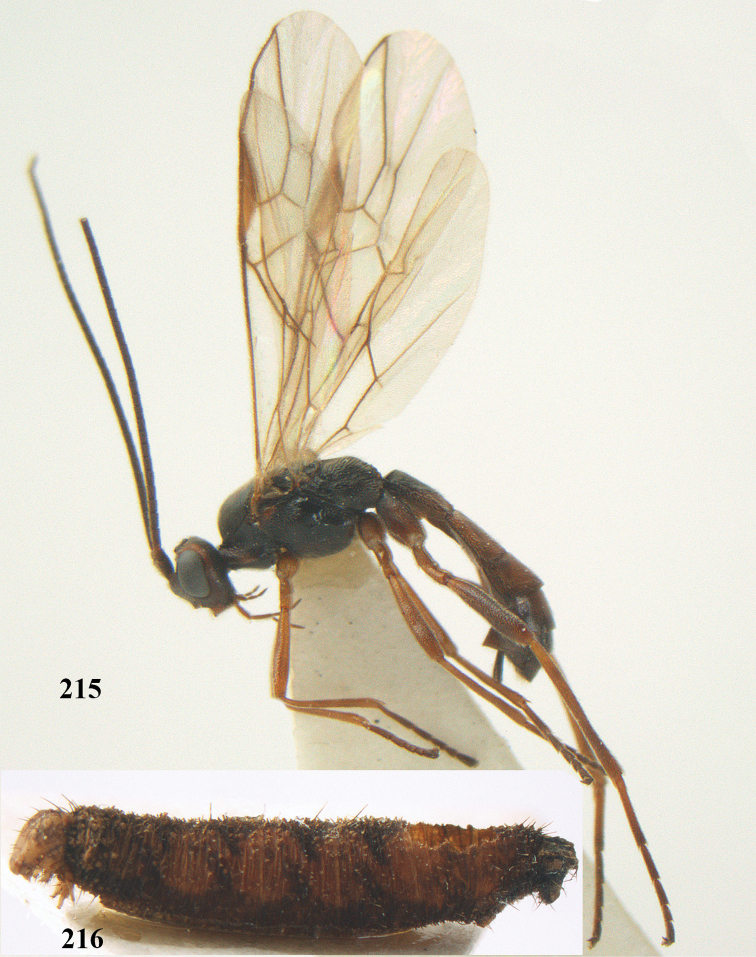
*Aleiodes modestus* (Reinhard), ♀, England, Santon Downham. **215** habitus lateral **216** mummy of *Euphithecia vulgata* (Haworth).

##### Diagnosis.

**Figures 217–229.**
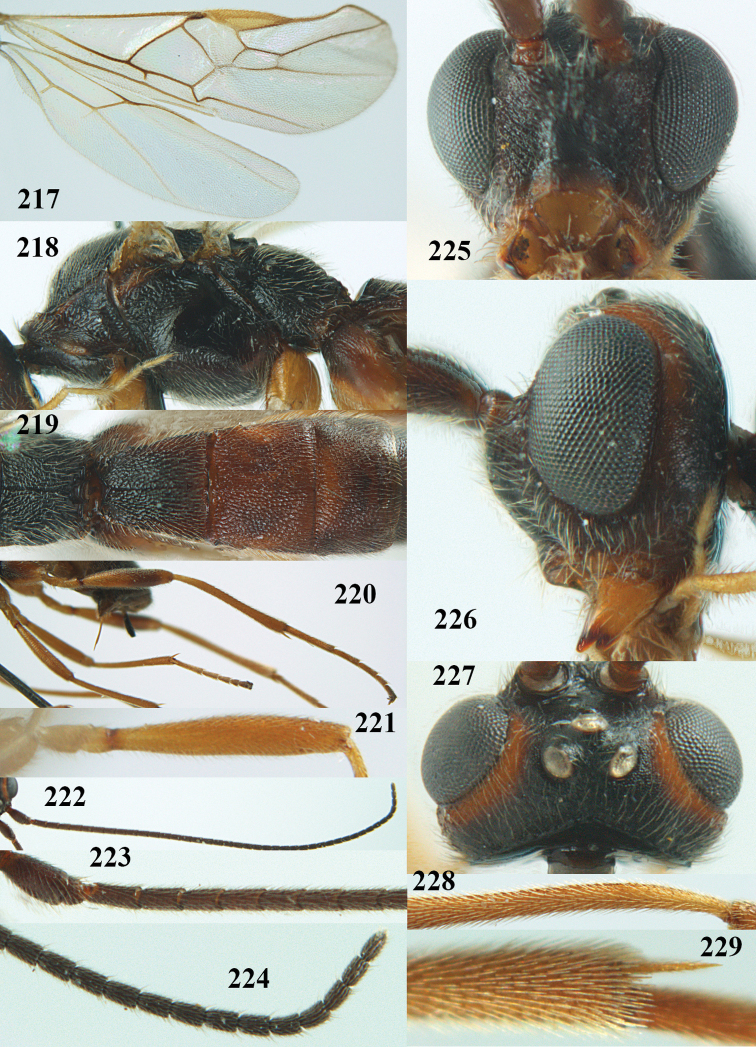
*Aleiodes modestus* (Reinhard), ♀, Netherlands, Wijster. **217** wings **218** mesosoma lateral **219** propodeum and metasoma dorsal **220** hind leg lateral **221** fore femur lateral **222** antenna **223** basal segments of antenna **224** apical segments of antenna **225** head anterior **226** head lateral **227** head dorsal **228** base of hind tibia **229** inner apex of hind tibia.

Antennal segments of both sexes 37–(40–45)–47 and third segment rather slender (Fig. 223); ventral margin of clypeus thin and protruding (Fig. 226); maximum width of hypoclypeal depression about 0.5 × minimum width of face (Fig. 225); length of malar space of female 0.2–0.3 × height of eye in lateral view (Fig. 226); OOL about equal to diameter of posterior ocellus (Fig. 227); area in front of anterior ocellus with a minute smooth tubercle (Fig. 227); vein r of fore wing 0.5–0.6 × vein 3-SR; vein 1-SR of fore wing short (Fig. 217); vein 1r-m of hind wing distinctly shorter than vein 1-M (Fig. 217); mesopleuron above precoxal area strongly shiny and superficially sculptured; third tergite without distinct striae; pterostigma largely yellow or yellowish brown; inner side of basal half of hind tibia yellowish; head and mesosoma largely blackish.

##### Description.

Figured ♀ (RMNH) from Wijster (Netherlands), length of fore wing 5.4 mm, of body 5.6 mm.

*Head*. Antennal segments of ♀ 43, moderately setose, length of antenna 1.2 × fore wing, its subapical segments distinctly longer than wide (Fig. 224); frons rather flat and granulate; OOL equal to diameter of posterior ocellus and finely granulate; vertex finely granulate, with satin sheen; clypeus rather large, micro-granulate; ventral margin of clypeus thin and rather protruding forwards (Fig. 226); width of hypoclypeal depression 0.5 × minimum width of face (Fig. 225); length of eye 1.9 × temple in dorsal view (Fig. 227); occiput behind stemmaticum mainly granulate with some rugulae; clypeus near lower level of eyes; length of malar space 0.25 × length of eye in lateral view; occipital carina complete; with a minute smooth tubercle in front of anterior ocellus.

*Mesosoma*. Mesoscutal lobes densely and finely granulate and with punctulation, matt; prepectal carina complete, distinct; precoxal area of mesopleuron with some rugulae medially; mesopleuron above precoxal area strongly shiny, sparsely punctate and with some superficial micro-granulation (Fig. 218); metapleuron largely granulate; scutellum flat, micro-granulate and without lateral carina; propodeum convex, finely rugose and interspaces micro-granulate (Fig. 219), without tubercles, median carina complete, weak and rather irregular.

*Wings*. Fore wing: r 0.6 × 3-SR (Fig. 217); 1-CU1 horizontal, slender, 0.35 × 2-CU1; r-m 0.5 × 3-SR; second submarginal cell rather long (Fig. 217); cu-a vertical, straight posteriorly; 1-M slightly curved posteriorly. Hind wing: marginal cell subparallel-sided but slightly constricted medially, its apical width equal to width at level of hamuli (Fig. 217); 2-SC+R subquadrate; m-cu faintly indicated; M+CU:1-M = 5:4; 1r-m 0.6 × as long as 1-M.

*Legs*. Tarsal claws yellowish setose; hind coxa granulate; hind trochantellus rather robust; length of fore femur, hind femur and basitarsus 5.7, 5.2 and 8.6 × their width, respectively (Figs 220–221); length of inner hind spur 0.25 × hind basitarsus.

*Metasoma*. First tergite rather robust (Fig. 219); first and second tergites densely and rather finely rugose, with rather weak median carina, reduced posteriorly; medio-basal area of second tergite absent; length of second tergite 0.9 × its basal width; second suture rather deep and distinctly crenulate; third tergite 0.8 × as long as second tergite, anterior two-thirds rugose and remainder of metasoma superficially granulate and punctate, somewhat compressed; fourth and apex of third tergite without sharp lateral crease; ovipositor sheath widened and setose.

*Colour*. Black; palpi, tegulae and pterostigma yellow; veins (except basally) dark brown; malar area ventrally, orbita dorsally and posteriorly, pronotum anteriorly and ventrally, mesopleuron antero-dorsally and postero-ventrally, hind coxa (but basally black), medio-posterior part of mesoscutum, first tergite latero-posteriorly, second and third tergites dark reddish brown; apical half of hind femur largely dark brown, except apically; antenna, telotarsi and clypeus dark brown; remainder of legs yellowish brown or blackish; wing membrane subhyaline.

*Variation.* Antennal segments of ♀ 38(1), 39(0), 40(0), 41(1), 42(18), 43(27), 44(12), 45(4) and of ♂ 40(1), 41(1), 42(2), 43(37), 44(18), 45(2); length of antenna of ♂ 1.3 × fore wing; length of eye of ♀ 1.2–1.9 × temple in dorsal view, of ♂ 1.6–2.5 times; dark specimens have mesosoma, metasoma and hind coxa nearly completely black; pale specimens have scapus, pedicellus, clypeus, entire orbita, notaulic area of mesoscutum, metapleuron posteriorly, first tergite apically and most of second–fourth tergites dark reddish brown; pterostigma sometimes slightly infuscate laterally, but remaining largely yellow; the infuscation of the hind femur is sometimes diffuse; vein cu-a of fore wing vertical or oblique; rugae of precoxal area may be entirely absent.

##### Note.

The number of antennal segments does not differ appreciably between the sexes. The specimens reported from Hungary by Papp (1983, 1985a, 2005) are misidentified *Aleiodes fortipes* (Reinhard).

#### Aleiodes nigriceps

Taxon classificationAnimaliaHymenopteraBraconidae

Wesmael, 1838

Figs 230–231 , 232–242

Aleiodes nigriceps Wesmael, 1838: 109; Papp 1985a: 156 (lectotype designation).
Aleiodes circumscriptus var. nigriceps ; Shenefelt 1975: 1171.

##### Type material.

Lectotype, ♀ (KBIN) from Belgium examined.

##### Additional material.

*Austria, Belgium, British Isles (*England: V.C.s 3, 8, 12, 19, 20, 22, 23, 24, 25, 27, 28, 29, 30, 31, 33, 40, 58, 62, 65; *Wales: V.C.s 41, 45, 52; *Isle of Man: V.C. 71; *Scotland: V.C.s 75, 77, 83, 84, 89, 97, 99, 101, 105; *Ireland: V.C. H5), *Jersey, *Bulgaria, *Czech Republic, *Finland, France, Germany, Hungary, *Italy, *Lithuania, *Netherlands (FR: Terschelling (Formerum), GE: Putten, NB: Geertruidenberg; Oploo; Udenhout (de Brand), NH: Overveen; Muiderberg, LI: Tegelen; St. Pietersberg; Venlo; Maastricht, UT: Harmelen, ZE: Cadzand; Oostkapelle; ZH: Delft; Den Haag; Wassenaar; Leidschendam), *Norway, *Poland, *Portugal, Russia, *Slovakia, Spain, *Sweden and **Switzerland**. Specimens in NMS, BMNH, RMNH, BZL, SDEI, ZSSM, FMNH, MTMA, CNC, P.-N. Libert collection, JLC, WAE, H. Haraldseide collection, and MSC.

##### Molecular data.

MRS075 (England KU682221, CO1), MRS144 (Scotland KU682228, CO1), MRS613 (Wales KU682243, CO1), MRS783 (Austria KU682250, CO1).

##### Biology.

At least partly a plurivoltine parasitoid of larvae of low-feeding noctuine Noctuidae inhabiting grassy sites; most frequent in humid situations. Mummy (Fig. 231) dark brown or brown and slender, only slightly or not swollen, forming low down in concealment. Specimens (in NMS unless stated otherwise) reared from wild-collected Noctuidae identified as *Diarsia rubi* (Vieweg) (10 [2 are ZSSM]; G.M. Haggett, M.R. Shaw), *Lycophotia porphyyrea* (Dennis and Schiffermüller) (1 [ZSSM/Netherlands]), *Noctua fimbriata* (Schreber) (1; J. Connell/Austria), *Xestia c-nigrum* (Linnaeus) (1; J. Connell/Austria), *Xestia sexstrigata* (Haworth) (3; G.M. Haggett), and indet. green noctuid (1). A further specimen was reared from a noctuid mummy collected in a *Carex* tussock (D.G. Notton). Specimens in NMS reared in culture experiments as follows: *Xestia sexstrigata* 6:20\20\\15+2, *Xestia xanthographa* (Denis & Schiffermüller) 4:47\47\\3+30 [several others were retarded but died], *Noctua fimbriata* 6:45\40\\24+0 [several others were retarded but died], *Diarsia rubi* 6:89\83\\74+9, *Phlogophora meticulosa* (Linnaeus) 7:43\19\\0+12 [several others were retarded but died]. No ovipositions resulted from the following culture experiments: *Diarsia mendica* (Fabricius) 1:5\0\\-, *Xanthorhoe fluctuata* (Linnaeus) 3:19\0\\-, *Xanthorhoe montanata* (Denis & Schiffermüller) 3:15\0\\-, *Camptogramma bilineata* (Linnaeus) 6:13\0\\-. While many of its hosts are univoltine with overwintering larvae, in which the parasitoid overwinters as an early instar larva, *Aleiodes nigriceps* has the capacity to be plurivoltine (e.g. in *Diarsia rubi*), and its long summer flight period, including abundant males in late summer, indicates that it regularly is so. Experimental rearings suggest that even within the genera it uses as hosts it specialises narrowly, and these experiments also clearly distinguish between it and both *Aleiodes pictus* and *Aleiodes leptofemur*. The mummy forms low down, more or less in concealment, and is very seldom collected (unlike that of *Aleiodes leptofemur*). The adult emerges soon after. In captivity adult females of this species are long-lived, but have always died off during the winter, and the flight period is ca May–September. Males are sluggish in their courtship, even towards conspecific females which, however, typically offer themselves for mating immediately.

**Figures 230–231.**
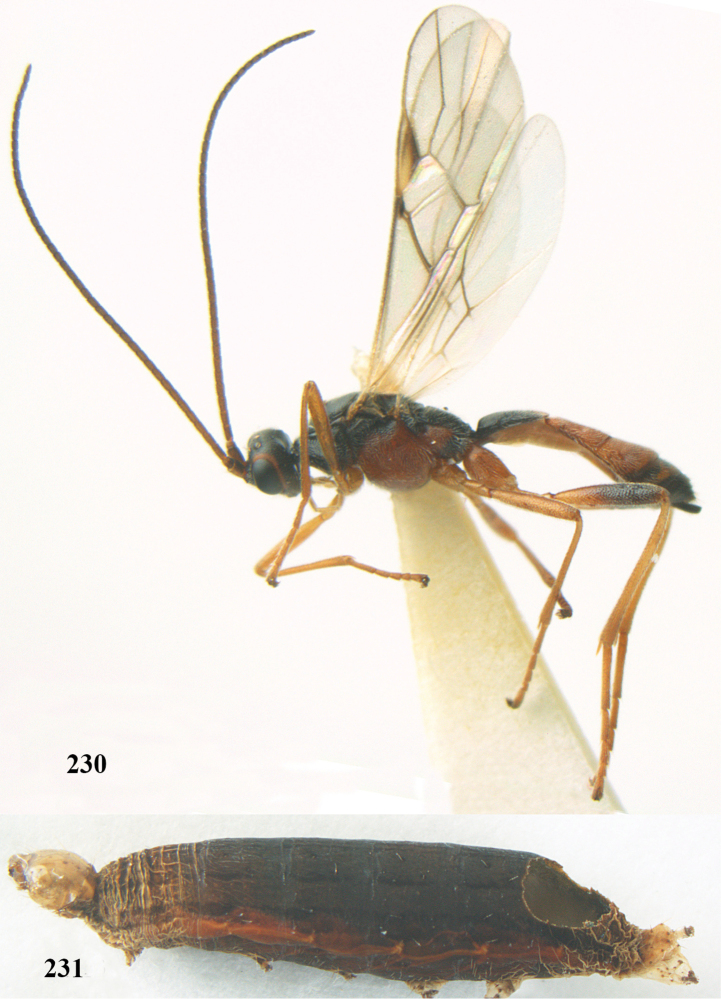
*Aleiodes nigriceps* Wesmael, ♀, Wales, Anglesey. **230** habitus lateral **231** mummy of *Xestia sexstrigata* (Haworth).

##### Diagnosis.

Antennal segments of female (38–)39–43, of male (38–)39–44; pale area of facial (= inner) orbita of female usually narrow or absent (Fig. 239); OOL 1.6 × diameter of posterior ocellus; face usually wholly black or dark brown, rarely wholly yellow brown; mesopleuron without distinct rugulae; mesosternum yellowish or reddish, if darkened then dark area usually not sharply defined; width of hypoclypeal depression 0.4–0.5 × minimum width of face (Fig. 239); mesoscutum antero-laterally and pronotum medio-anteriorly often black or dark brown; face without distinct rugae or only a few rugae medio-dorsally; ventrally mesopleuron usually widely orange brown; malar space partly or completely and temple near eye orange brown; length of first tergite of ♀ 1.1–1.2 × its apical width; second tergite comparatively wide (Fig. 234); fore and hind femora comparatively slender (Figs 235–236); pterostigma partly darkened anteriorly; mummy slightly or not swollen and dark brown or brown; middle third of hind femur partly or entirely dark brown. Very similar to *Aleiodes pictus* (Herrich-Schäffer, 1838), but *Aleiodes pictus* has the antennal segments of ♀ (35–)36–40, of ♂ (36–)37–41, the mesopleuron with some rugulae (at most very weakly evident in *Aleiodes nigriceps*), the mesosternum almost always strongly darkened or black and dark area usually sharply defined, malar space somewhat longer than in *Aleiodes nigriceps*, and the mesoscutum antero-laterally and pronotum medio-anteriorly more often yellowish (but variable in both species). The yellowish colouration is frequently more brownish in *Aleiodes pictus* (tending to orange in *Aleiodes nigriceps*), the legs are on the whole less slender, and the paler area at the extreme apex of the hind femur tends to be more extensive. In practice, the majority of specimens of *Aleiodes nigriceps* have both the mesosternum and third tergite completely yellowish orange, and such specimens are easy to recognise as this combination is rarely approached in *Aleiodes pictus*. In extreme examples of *Aleiodes nigriceps* the mesoscutum and (less often) even the scutellum may be wholly black, and when the tergites and scape are also predominantly dark they may resemble *Aleiodes nigricornis*, but in that species the hind femur is only very rarely extensively darkened, and the number of antennal segments is greater (though with a small overlap).

**Figures 232–242.**
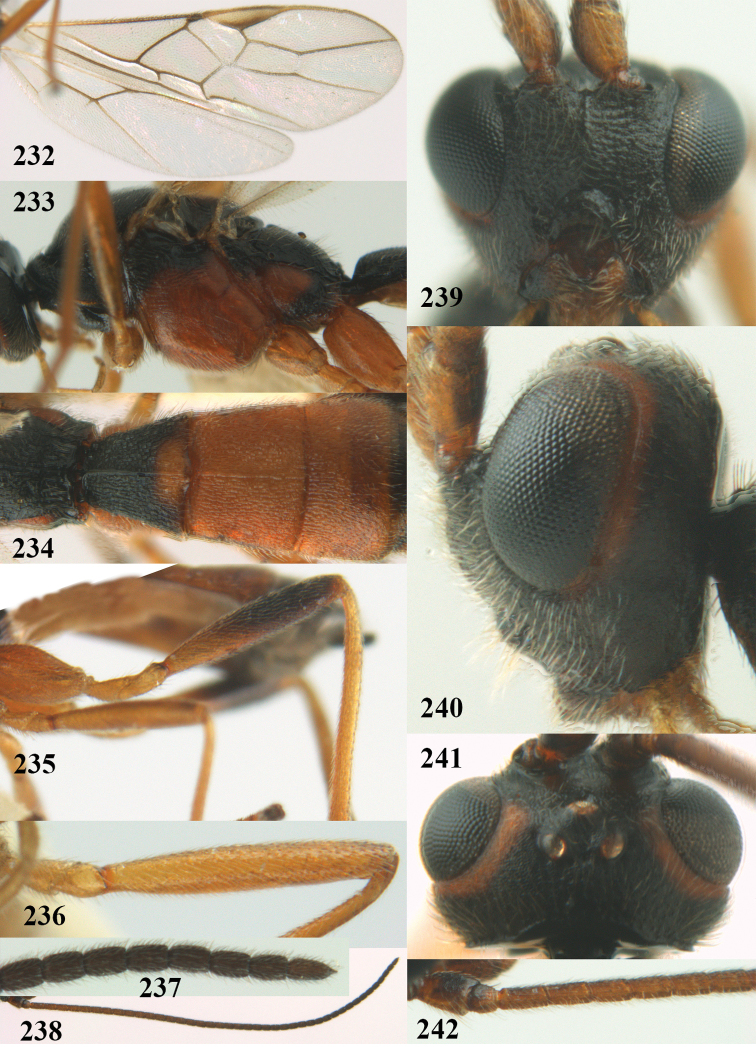
*Aleiodes nigriceps* Wesmael, ♀, Wales, Anglesey. **232** wings **233** mesosoma lateral **234** propodeum and metasoma dorsal **235** hind leg lateral **236** fore femur lateral **237** apical segments of antenna **238** antenna **239** head anterior **240** head lateral **241** head dorsal **242** basal segments of antenna.

##### Description.

Redescribed ♀ (NMS), “Culture: [Wales], Anglesey, Llangristiolus, from ♀ 3:2 [ex] *Diarsia rubi* in *Xestia sexstrigata*, ovip. 28.v.[20]08, mum. 4.iv.[20]09, em. 22.v.2009, died viii.2009, ♀ 2/09, M.R. Shaw”. Length of fore wing 4.4 mm, of body 5.5 mm.

*Head.* Antennal segments 43, length of antenna 1.3 × fore wing, its subapical segments about 1.6 × as long as wide; frons granulate-coriaceous, with satin sheen; OOL 1.6 × diameter of posterior ocellus (POL 1.4 times); vertex granulate-coriaceous, with satin sheen; clypeus distinctly convex and largely smooth; ventral margin of clypeus thick and depressed (Fig. 239); width of hypoclypeal depression 0.4 × minimum width of face (Fig. 239) and face mainly coriaceous with some rugulae medio-dorsally; length of eye 2.3 × temple in dorsal view and temple roundly narrowed behind eye (Fig. 241); occiput behind stemmaticum coriaceous and occipital carina absent medio-dorsally, its interruption slightly less than width of stemmaticum (Fig. 241); clypeus partly above lower level of eyes (Fig. 239); length of malar space 0.4 × height of eye in lateral view; eyes moderately protruding (Figs 239–241).

*Mesosoma.* Mesoscutal lobes finely coriaceous, with satin sheen, but medio-posteriorly longitudinally rugose; notauli narrow, moderately impressed and finely crenulate, but posteriorly lost in rugose area; prepectal carina narrow lamelliform and reaching anterior border; precoxal area of mesopleuron coriaceous and without rugae medially; mesopleuron above precoxal area (except small smooth and shiny speculum) coriaceous, but antero-dorsally rugose; medially metapleuron coriaceous, matt; mesosternal sulcus narrow and moderately deep, but posteriorly shallow and with a carina; mesosternum rather angulate posteriorly; scutellum nearly flat, coriaceous and largely non-carinate laterally; propodeum rather flat and coriaceous but posteriorly with some rugae, median carina complete, without tubercles.

*Wings.* Fore wing: r 0.2 × 3-SR (Fig. 232); 1-CU1 horizontal, 0.5 × as long as 2-CU1; r-m 0.7 × 2-SR, and 0.4 × 3-SR; second submarginal cell medium-sized (Fig. 232); cu-a oblique, but not parallel with CU1b, straight; 1-M nearly straight posteriorly. Hind wing: apical half of marginal cell somewhat widened apically (Fig. 232); 2-SC+R short and longitudinal; short stub of m-cu present, pigmented.

*Legs.* Tarsal claws setose; hind coxa superficially coriaceous, with satin sheen; hind trochantellus 2.3 × longer than wide; length of fore and hind femora 6.3 and 5.6 × their width, respectively (Figs 235–236); inner apex of hind tibia without comb; length of inner hind spur 0.3 × hind basitarsus.

*Metasoma.* First tergite 1.2 × as long as wide posteriorly, convex, but posteriorly flattened; first and second tergites and base of third tergite densely longitudinally rugose; second tergite robust (Fig. 234), with distinct median carina, matt; medio-basal area of second tergite obsolescent; second suture shallow and crenulate; third tergite (except basally) largely coriaceous, remainder of metasoma largely smooth and rather shiny; fourth and apical third of third tergite without sharp lateral crease; ovipositor sheath largely densely setose.

*Colour.* Black or brownish black; antenna brown, but scapus and pedicellus dorsally and laterally and apical third of antenna dark brown; palpi and tegulae pale yellowish (Fig. 230); outer orbita posteriorly and dorsally brownish yellow (Figs 240–241) and remainder of head black; mesopleuron (except dorsally), mesosternum and metapleuron medially orange brown; hind femur (except basally) dark brown (Fig. 235); scutellum, first tergite medio-apically, second and third tergites, part of fourth tergite and remainder of legs brownish yellow; veins (except dark brown veins 1-SR, 1-M, r and CU1) brown; pterostigma yellowish, but anteriorly and posteriorly darkened (Fig. 232); wing membrane subhyaline.

*Variation.* Length of fore wing 4.3–4.4 mm; antennal segments of ♀ 38(1), 39(9), 40(44), 41(44), 42(27), 43(13), 44(2), 45(1); of ♂ 37(1), 38(4), 39(31), 40(82), 41(113), 42(33), 43(12), 44(7), 45(3); vein r of fore wing 0.2–0.5 × vein 3-SR; length of first tergite of ♀ 1.1–1.2 × its apical width; pterostigma medially yellow or dark brown; medio-posteriorly mesoscutum black or brownish yellow; second and third tergites yellowish, infuscate or dark brown laterally, mesosternum orange brown or infuscate.

##### Notes.

The two sexes have about the same number of antennal segments. The lectotype of *Aleiodes nigriceps* has the antenna mutilated; according to the original description it had 40 or 41 segments. *Aleiodes nigriceps* is often considered to be a synonym of *Aleiodes circumscriptus*, e.g. Papp (1985) but the selection of a neotype for *Aleiodes circumscriptus* in the present work resolves that issue (and the two species differ in, among other things, the number of antennal segments).

#### Aleiodes nigricornis

Taxon classificationAnimaliaHymenopteraBraconidae

Wesmael, 1838

Figs 243–244 , 245–256

Aleiodes nigricornis Wesmael, 1838: 105; Shenefelt 1975: 1178; Papp 1991: 112, 1985: 160 (lectotype designation); Belokobylskij et al. 2003: 398.

##### Type material.

Lectotype, ♀ (KBIN) from Belgium examined.

##### Additional material.

*Austria, Belgium, British Isles (**England**: V.C.s 2, 3, 7, 11, 16, 17, 20, 22, 23, 24, 25, 29, 30, 31, 34, 38, 57, 58, 59, 60, 63, 64, 65, 66, 67, 69; **Wales**: V.C.s 35, 44, 48, 52; **Scotland**: V.C.s 72, 75, 78, 79, 83, 85, 86, 87, 88, 89, 90, 95, 96, 97, 98, 99, 101, 102, 105, 107, 108, 111; **Ireland**: V.C.s H21, H28, H29, “Westport”), **Czech Republic**, *Denmark, France, **Finland**, **Germany**, **Italy**, **Netherlands** (GE: Ede; Nunspeet; Heerde; Otterlo, Velp, LI: Grubbenvorst, NB: Geertruidenberg; Helvoirt; Bergen op Zoom; Etten-Leur, NH: Overveen; Muiderberg, ZH: Den Haag; Meijendel; Oegstgeest; Voorschoten; Asperen; Waarder), *Norway, **Poland**, **Russia**, **Slovakia**, **Sweden**, **Switzerland**. Specimens in NMS, BMNH, RMNH, BZL, MTMA, NRS, SDEI, CC, FMNH, ZSSM, OUM, NMI, CMIM, Sheffield Museum, SMNS, ZISP, SYKE, ZMUO, USNM, CNC, UWIM, M. Riedel collection, JLC, MSC, AAC, WAE, I. Kakko collection, H. Haraldseide collection.

##### Molecular data.

MRS216 (Scotland EU979585, CO1 + AJ784934, 28S), MRS373 (Sweden KU682258, CO1), MRS790 (Scotland KU682254, CO1), MRS794 (England KU682255, CO1).

##### Biology.

A plurivoltine parasitoid of Noctuidae, using *Apamea* species in which to overwinter, and (possibly exclusively) *Orthosia gothica* (Linnaeus) in early summer. The mummy is moderately dark brown, rather elongate (Fig. 244) and often found in exposed positions. Specimens of the overwintering generation reared from noctuids identified as *Apamea crenata* (Hufnagel) (2 ZSSM, 1 NMS, 1 NRS; E. Haeselbarth, R.I. Lorimer), *Apamea epomidion* (Haworth) (2 ZSSM, 1 OUM; E. Haeselbarth), Apamea ?monoglypha (Hufnagel) (1 NMS; M.R. Britton), Apamea ?remissa (Hübner) (1 H. Haraldseide/Norway) and ?*Apamea* sp. (11, hosts unidentified or misidentified but with preserved mummies that are consistent with *Apamea* species (det. M.R. Shaw), having at least moderately large shining warts and a well-developed prothoracic plate: 7 (2 as *Xestia xanthographa* (Denis & Schiffermüller)) NMS, 4 (as *Polia* spp.) OUM, 1 (as *Noctua fimbriata* (Schreber)) RMNH, 1 AAC). Specimens of the summer generation reared from *Orthosia gothica* (3 NMS; R.I. Lorimer, M.R. Shaw), and unidentified noctuid mummies compatible with *Orthosia gothica* (8 NMS, 1 ZMUO, 1 ZSSM, 2 H. Haraldseide collection). Other specimens (both generations) with a host recorded (*Mythimna ferrago* (Fabricius) in ZSSM, *Epirrita autumnata* (Borkhausen) in CC) have not been accompanied by mummies and we regard the records as dubious. Experimental results from female ex Apamea ?monoglypha; with 2^nd^ instar hosts: *Orthosia gothica* 1:9\9\\6\3, *Orthosia cerasi* (Fabricius) 1:10\ 0\\-, *Orthosia incerta* (Hufnagel) 1:4\0\\-; with 3^rd^ instar hosts *Orthosia gothica* 1:8\8\\0\8. The single female trialed with hosts undertook considerable non-destructive concurrent host feeding on *Orthosia gothica*. Adult flight time approximately May to August, with females persisting until October.

**Figures 243–244.**
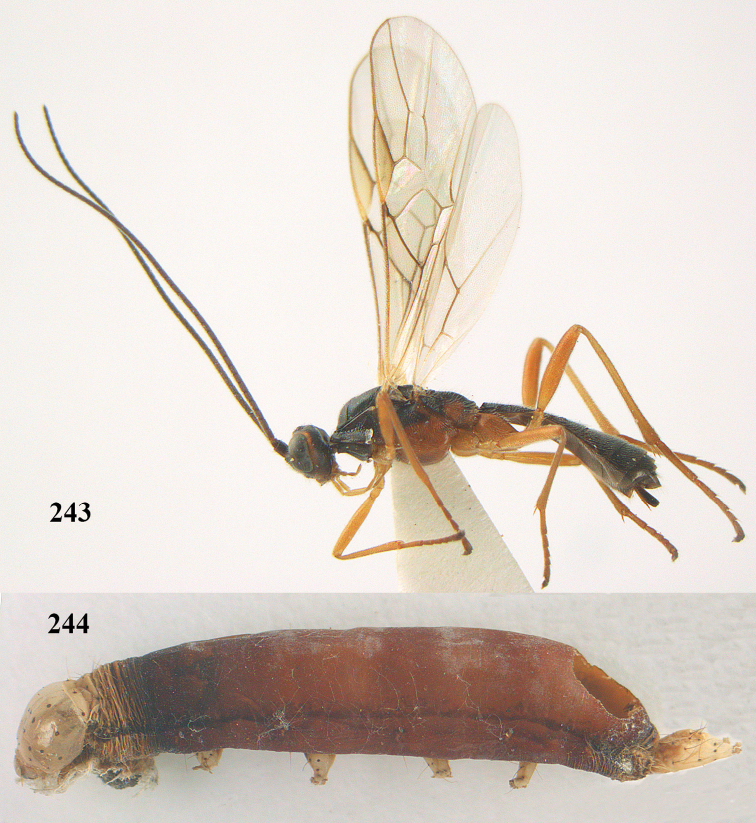
*Aleiodes nigricornis* Wesmael, ♀, England, Gait Barrows. **243** habitus lateral **244** mummy of *Orthosia gothica* (Linnaeus).

##### Diagnosis.

**Figures 245–256.**
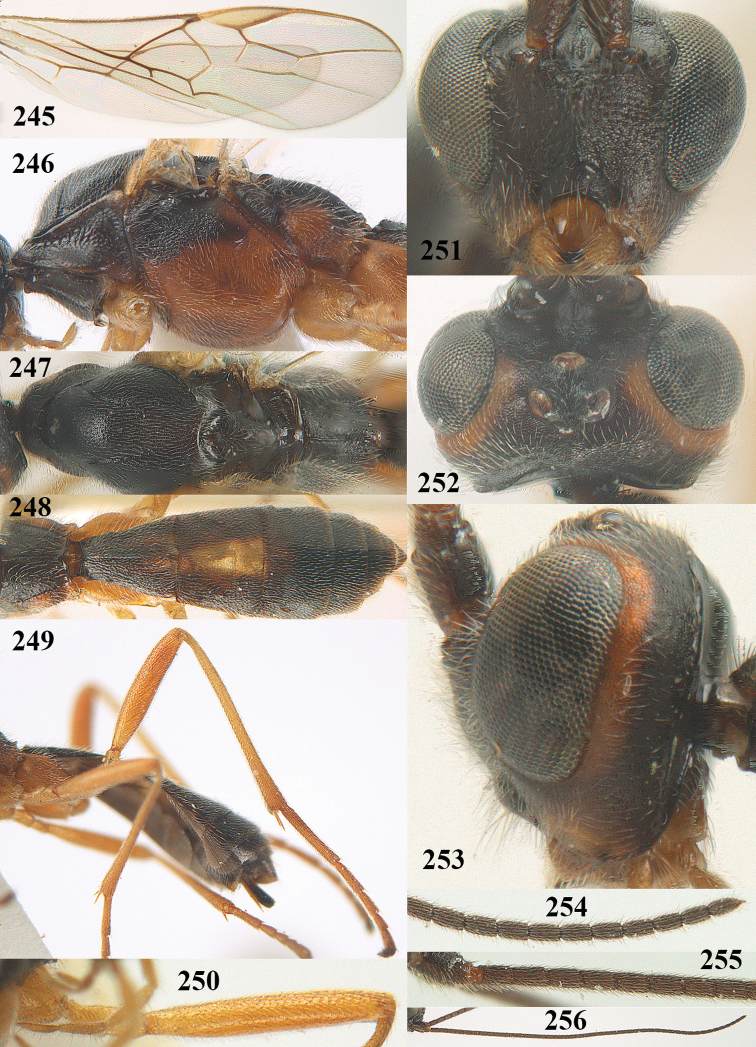
*Aleiodes nigricornis* Wesmael, ♀, England, Gait Barrows. **245** wings **246** mesosoma lateral **247** mesosoma dorsal **248** propodeum and metasoma dorsal **249** hind leg lateral **250** fore femur lateral **251** head anterior **252** head dorsal **253** head lateral **254** apical segments of antenna **255** basal segments of antenna **256** antenna.

Antennal segments of female 44–49, of male 43–47; antenna dark brown or black (also scapus and pedicellus more or less infuscate or black ventrally), rarely completely yellowish brown; temples directly narrowed behind eyes; OOL about equal to diameter of posterior ocellus (Fig. 252); pale yellowish part of malar space usually not reaching clypeus (Figs 251, 253); precoxal area usually without rugae; mesosternum usually reddish or brownish; propodeum largely coriaceous medially and median carina at least anteriorly present on posterior half of propodeum and regular; mesosoma (especially mesoscutum and scutellum) black (or blackish) dorsally, but notaulic area may be brownish posteriorly; fore femur of ♀ 6.7–7.4 × as long as wide (Fig. 250) and very finely sculptured; posterior half of pterostigma of ♀ more or less yellowish, but usually apical third laterally darkened; hind femur of ♀ rather reddish-brown, but may be largely infuscate in males; vein 1-CU1 of fore wing horizontal and vein cu-a short, far postfurcal (Fig. 245); metasoma largely blackish with (pale) yellowish elliptical patch medially (Fig. 248).

##### Description.

Redescribed ♀ (RMNH) from Helvoirt, length of fore wing 5.6 mm, of body 6.2 mm.

*Head.* Antennal segments 46, length of antenna 1.2 × fore wing, its subapical segments about twice as long as wide; frons mainly granulate, posteriorly with some rugulae, rather shiny and mainly flat; OOL 0.8 × diameter of posterior ocellus and granulate-coriaceous; vertex superficially granulate-coriaceous, with satin sheen; clypeus convex, coriaceous; ventral margin of clypeus thick and depressed (Fig. 253); width of hypoclypeal depression 0.4 × minimum width of face (Fig. 251) and face coriaceous and transversely rugose, except ventrally; length of eye 2.2 × temple in dorsal view and temple directly narrowed behind eye; occiput behind stemmaticum coriaceous and occipital carina nearly complete, interrupted dorsally by somewhat less than width of ocellus (Fig. 252); clypeus above lower level of eyes (Fig. 251); length of malar space 0.25 × height of eye in lateral view; eyes moderately protruding (Figs 251–253).

*Mesosoma.* Mesoscutal lobes finely granulate and with satin sheen; notauli narrow, moderately deep and smooth; prepectal carina lamelliform medio-ventrally, reaching anterior border; precoxal area of mesopleuron finely granulate and with some rugulae medially (Fig. 246); mesopleuron above precoxal area (except small and nearly smooth speculum) granulate, but dorsally rugose; medially metapleuron granulate and with satin sheen; mesosternal sulcus narrow and rather deep, without carina posteriorly; mesosternum rather angulate posteriorly (Fig. 246); scutellum nearly flat, granulate and largely non-carinate laterally; propodeum rather flat and granulate but posteriorly with some rugae, median carina complete and regular, evenly convex laterally.

*Wings.* Fore wing: r 0.3 × 3-SR (Fig. 245); 1-CU1 horizontal, 0.5 × as long as 2-CU1; r-m 0.8 × 2-SR, and 0.4 × 3-SR; second submarginal cell rather long (Fig. 245); cu-a slightly oblique, not parallel with CU1b, straight; 1-M straight posteriorly. Hind wing: apical half of marginal cell slightly widened (Fig. 243); 2-SC+R short and longitudinal; m-cu absent, except for a faint trace; M+CU:1-M = 6:5; 1r-m 0.65 × 1-M.

*Legs.* Tarsal claws setose; hind coxa superficially coriaceous, with satin sheen; hind trochantellus 2.3 × longer than wide; length of fore and hind femora 6.2 and 5.0 × their width, respectively (Figs 249–250); hind femur granulate-coriaceous; inner apex of hind tibia without comb; length of inner hind spur 0.3 × hind basitarsus.

*Metasoma.* First tergite 1.2 × as long as wide posteriorly, flattened and latero-posteriorly lamelliform; first–second tergites and base of third tergite coriaceous and finely irregularly longitudinally rugose; second tergite rather robust (Fig. 248), with median carina and rather shiny; medio-basal area of second tergite obsolescent; second suture narrow and distinctly crenulate; remainder of metasoma largely superficially coriaceous and shiny; fourth and apical third of third tergite without sharp lateral crease; ovipositor sheath largely densely setose and apically truncate.

*Colour.* Black or brownish-black; antenna dark brown; palpi, tegulae, tibiae (except apically), medio-apical fifth of first tergite and medially second tergite pale yellowish (Fig. 248); malar space nearly up to eyes, orbita dorsally and posteriorly, mesopleuron (except dorsally), mesosternum, metapleuron largely, mandible and remainder of legs, more or less reddish brown; veins (but of middle third of wing mainly dark brown) and pterostigma (but posterior border somewhat darkened) yellowish brown; wing membrane subhyaline.

*Variation.* Antennal segments of ♀ 42(2), 43(4), 44(10), 45(44), 46(73), 47(59), 48(12), 49(5); of ♂ 42(3), 43(24), 44(20), 45(30), 46(15), 47(9), 48(1); mesosternum reddish brown or partly fuzzy dark brown; precoxal area of mesopleuron medially entirely granulate or rarely with some weak rugulae; scapus entirely dark brown or partly brown.

##### Notes.

The lectotype has 46 antennal segments. On average males have 1–2 fewer antennal segments than females.

#### Aleiodes pallidator

Taxon classificationAnimaliaHymenopteraBraconidae

(Thunberg, 1822)

Figs 257 , 258–259 , 260–271

Ichneumon pallidator Thunberg, 1822: 259.
Aleiodes pallidator ; Shenefelt 1975: 1179; Papp 1991: 101; Belokobylskij et al. 2003: 398.
Rogas pallidator ; Tobias 1986: 81 (transl.: 135).
Rogas ochraceus Curtis, 1834: 512.4; Shenefelt 1975: 1182 (as synonym of Aleiodes testaceus) (examined).
Aleiodes ochraceus ; Papp 1991: 101 (as synonym of Aleiodes pallidator); Belokobylskij et al. 2003: 399 (as synonym of Aleiodes similis).
Aleiodes unicolor Wesmael, 1838: 111; Roman 1912: 271 (synonym of Aleiodes pallidator); Shenefelt 1975: 1179–1180; Papp 1985a: 160 (lectotype designation) (examined).

##### Type material.

Lectotype of *Ichneumon pallidator* here designated, ♀ (ZMUU) from **Sweden** (“α”, “Rhogas (Aleiodes) pallidator Thbg”). Holotype of *Rhogas ochraceus*, ♀ (Melbourne) from England (Regent’s Park), “Type”, “*Rhogas ochraceus* Curtis, type, J.F. Perkins, 1948”. Lectotype of *Aleiodes unicolor*, ♀ (KBIN) from Belgium and 2 paralectotypes examined.

##### Additional material.

**British Isles** (**England**: V.C.s 11, 15, 24, 59, 60), **Bulgaria**, **Netherlands** (FL: Bant; Lelystad (Jagersbos), FR: Ried, GE: Ede (Maanderbroek); Zaltbommel (Kerkwijk), GR: Scheemda, LI: Reuven; Vlodrop, NB: Nederweert; Geffen; Valkenswaard; Heusden, ZH: Rotterdam; Lexmond; Melissant), **Germany**, **Hungary**, *Romania, **Russia**, **Serbia**, **Slovakia**, **Sweden**, **Turkey**. Specimens in NMS, BMNH, RMNH, OUM, ZSSM, MCZ, CC, CNC, UWIM, R. van der Hout collection.

##### Molecular data.

MRS001 (Turkey EU979586, CO1 + EU854333, 28S).

##### Biology.

**Figure 257.**
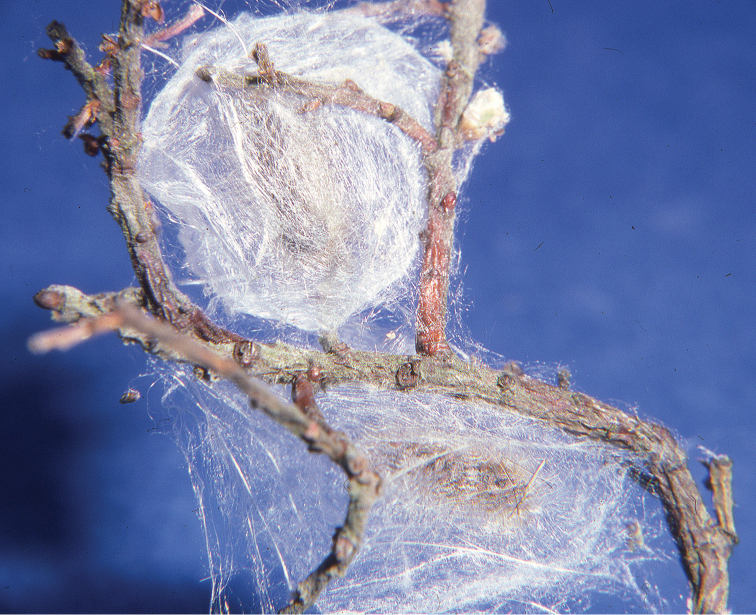
*Aleiodes pallidator* (Thunberg), England, penultimate instar mummies in precocious pseudococoons of *Leucoma salicis* (Linnaeus).

A univoltine, thelytokous specialist parasitoid of the erebid lymantriine *Leucoma salicis* (Linnaeus), overwintering in the host. More than 200 reared specimens seen from *Leucoma salicis* (most in NMS, others in BMNH, RMNH, ZSSM, OUM, CC, MCZ). Its biology has been studied by Dowden (1938) in the course of its attempted introduction to North America for the control of the introduced *Leucoma salicis*: the notes given here supplement rather than repeat his findings. Based on English data the adult flight time is from the last few days of June through to early September. In experimental rearings the females were slow to accept *Leucoma salicis* larvae, but always did so eventually after repeated contact (being especially attracted to traces of silk), and second instars were only marginally more acceptable than firsts. This may suggest some adaptation to the essentially gregarious nature of early stage *Leucoma salicis* larvae. Oviposition was brief, usually taking about 5 but sometimes up to 20 seconds: although there was a pre-oviposition sting the female usually did not wait for paralysis to take effect before ovipositing, but would then avoid superparasitism of hosts thus rendered sluggish for a short period, though not subsequently. Partly because of winter mortality exact quantitative data are not available, but from about 100 observed single ovipositions the success rate in *Leucoma salicis* (partly judged from living established larvae within overwintering hosts) was at least 90%. In one experiment, already briefly reported by Askew & Shaw (1986), a cohort of *Leucoma salicis* larvae (N = 57) parasitised by *Aleiodes pallidator* and kept under outdoor conditions came out of their hibernacula a mean of 9.2 days later than controls (N = 66) from the same host egg batch (t = 9.18, P < 0.001). This delay was interpreted as extending the range of host plants suitable for the development of parasitised hosts, as spring bud burst varies greatly between the Salicaceae present at the English site of origin (Ainsdale, Lancashire); in addition, however, this behaviour by a monophagous parasitoid might be an adaptation to ensure that there is reasonably good synchrony with the next host generation. In experiments to test host range extension, inexperienced females would not oviposit into other species of Lymantriinae, but females that had already oviposited into *Leucoma salicis* often would do so quite readily into both *Euproctis similis* (Fuessly) and *Dicallomera fascelina* (Linnaeus), although attempts were often at least for a time thwarted by the longer setae of the trial hosts. In all cases in which oviposition occurred, hosts were later dissected and found to contain encapsulated parasitoids (usually eggs; possibly in some cases first instar larvae). The results of trials were *Euproctis similis* 2:15\8\\0, *Dicallomera fascelina* 3:12\6\\0, *Orgyia antiqua* (Linnaeus) 4:7\0\\-, *Lymantria dispar* (Linnaeus) 1:5\0\\-. The penultimate (or earlier) instar *Leucoma salicis* larva is induced to prepare a frail cocoon, as though for pupation, within a leaf package just before being mummified, and the mummy forms within that structure with only its setae in contact (Fig. 257). Although the parasitoid larva does make a ventral opening in the host’s thoracic region, through which fluid escapes and dries, the mummy is not thereby stuck down.

##### Diagnosis.

**Figures 258–259.**
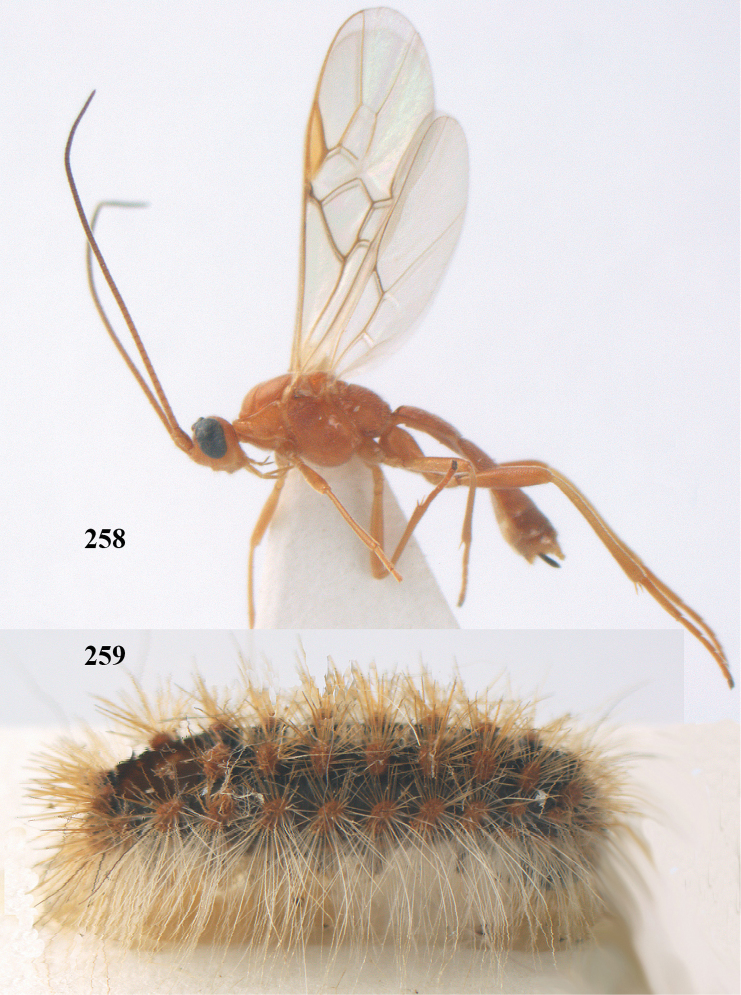
*Aleiodes pallidator* (Thunberg), ♀, Netherlands, Nederweert. **258** habitus lateral **259** mummy of *Leucoma salicis* (Linnaeus).

**Figures 260–271.**
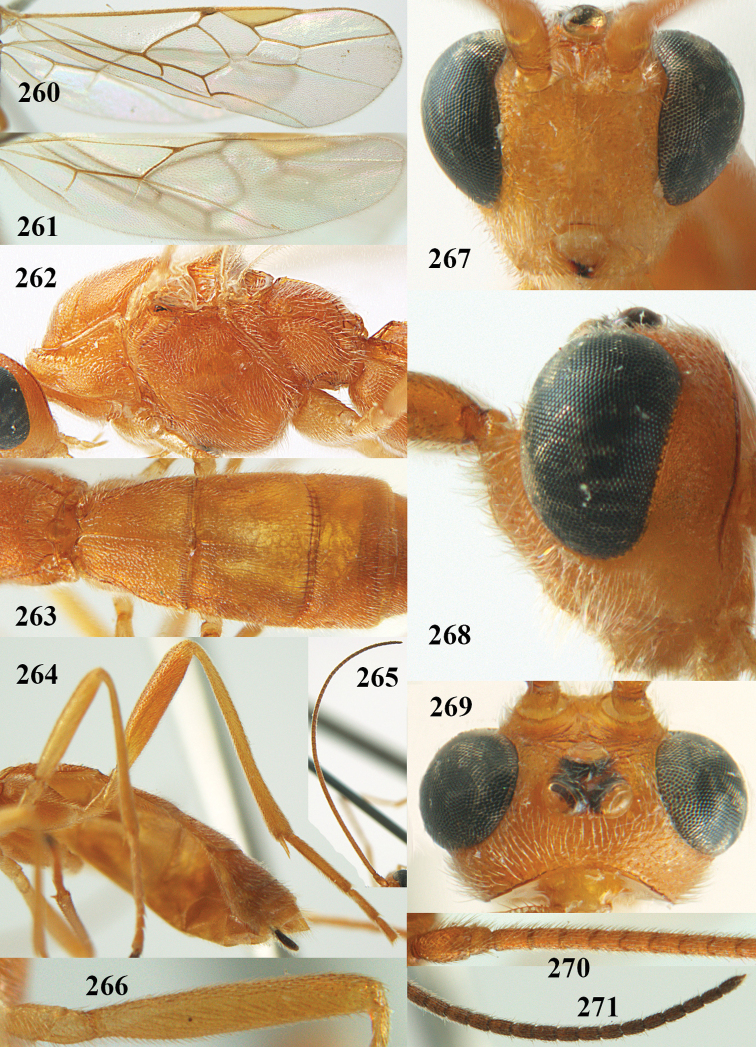
*Aleiodes pallidator* (Thunberg), ♀, Netherlands, Nederweert. **260** fore wing **261** hind wing **262** mesosoma lateral **263** propodeum and metasoma dorsal **264** hind leg lateral **265** antenna **266** fore femur lateral **267** head anterior **268** head lateral **269** head dorsal **270** basal segments of antenna **271** apical segments of antenna.

Antennal segments of ♀ 50–55 **and** head (except stemmaticum) entirely brownish yellow; scapus in lateral view distinctly oblique apically (Fig. 258); OOL 0.6 × diameter of posterior ocellus; occipital carina complete ventrally or nearly so (Fig. 268); vein 2-CU1 of fore wing 2.2–3.0 × vein 1-CU1 (Fig. 260); vein 1-SR linearly connected to vein 1-M and vein 1-M straight or slightly curved (Fig. 260); fourth metasomal tergite largely (superficially) granulate; length of fore wing 5–7 mm. Easily confused with *Aleiodes varius* (Herrich-Schäffer), but this species has more antennal segments (♀: 66–71), malar space 0.6 × as long as height of eye, vein 2-CU1 of fore wing 1.6–1.8 × vein 1-CU1 and the occipital carina is reduced ventrally. Often also confused with *Aleiodes gastritor* (Thunberg) s. lat., but specimens of this species-group have many fewer antennal segments, the segments are more elongate and the pterostigma is often more or less dark brown or infuscate.

##### Description.

Redescribed ♀ (RMNH) from Nederweert, length of fore wing 5.9 mm, of body 7.1 mm.

*Head.* Antennal segments 54, length of antenna 1.2 × fore wing, its subapical segments about 1.8 × as long as wide and scapus in lateral view distinctly oblique apically (Fig. 258); frons superficially granulate and rugose, shiny; OOL 0.6 × diameter of posterior ocellus and rugulose; vertex granulate-rugulose, with satin sheen; clypeus rather high, convex, coriaceous; ventral margin of clypeus thick and gradually depressed (Fig. 268); width of hypoclypeal depression 0.4 × minimum width of face (Fig. 267) and face mainly coriaceous dorsally; length of eye 2.8 × temple in dorsal view and temple directly narrowed behind eye; occiput behind stemmaticum finely rugulose and occipital carina nearly complete medio-dorsally and ventrally (Figs 268–269); clypeus partly above lower level of eyes (Fig. 267); length of malar space 0.3 × height of eye in lateral view; eyes distinctly protruding (Figs 267–269).

*Mesosoma.* Mesoscutal lobes very finely coriaceous, with satin sheen, but medio-posteriorly rugose; notauli narrow, shallow and crenulate; prepectal carina rather lamelliform medio-ventrally, nearly reaching anterior border of mesopleuron and latero-ventrally angulate; precoxal area of mesopleuron with some short rugae medially (Fig. 262); mesopleuron above precoxal area (except large smooth and shiny speculum) superficially granulate, but dorsally extensively rugose; medially metapleuron coriaceous-rugulose, rather shiny; mesosternal sulcus narrow and rather deep, without carina posteriorly; mesosternum rather angulate posteriorly; scutellum finely coriaceous and non-carinate laterally; dorsal face of propodeum rather long and densely finely rugose, posterior face with some carinae and smooth in between, median carina complete, without tubercles, but somewhat angulate postero-laterally.

*Wings.* Fore wing: r 0.3 × 3-SR (Fig. 260); 1-CU1 horizontal, 0.35 × as long as 2-CU1; r-m 0.7 × 2-SR, and 0.4 × 3-SR; second submarginal cell elongate (Fig. 260); 1-SR angled with 1-M; cu-a rather oblique, not parallel with CU1b, slightly curved; 1-M slightly curved posteriorly. Hind wing: apical half of marginal cell parallel-sided or nearly so (Fig. 261); 2-SC+R short and longitudinal; m-cu present as fold, unpigmented; M+CU:1-M = 15:12; 1r-m 0.7 × 1-M.

*Legs.* Tarsal claws yellowish setose; hind coxa superficially finely coriaceous, with satin sheen; hind trochantellus 2.7 × longer ventrally than wide; length of fore and hind femora 6.2 and 4.9 × their width, respectively (Figs 264, 266); inner apex of hind tibia without comb; length of inner hind spur 0.3 × hind basitarsus.

*Metasoma.* First tergite 0.9 × as long as wide posteriorly, flattened and latero-posteriorly lamelliform; first–second tergites and base of third tergite densely finely irregularly rugose and with median carina; second tergite robust and 1.5 × as long as third tergite (Fig. 263); medio-basal area of second tergite absent; second suture deep and distinctly crenulate; apical half of third tergite granulate-coriaceous, remainder of metasoma largely superficially coriaceous and rather shiny; fourth tergite largely with sharp lateral crease; ovipositor sheath largely densely setose and apically rounded.

*Colour.* Yellowish brown; apical third of antenna and stemmaticum (except medially) dark brown; palpi, malar space up to eyes, mandible, tegulae, fore and middle legs, hind trochanter and trochantellus and pterostigma pale yellowish (Fig. 260); medial veins dark brown and other veins brownish yellow; wing membrane subhyaline.

*Variation.* Length of fore wing 5.6–6.5 mm, of body 6.3–7.5 mm; antennal segments of ♀ 50(3), 51(13), 52(43), 53(79), 54(44), 55(7); colour and shape are very uniform in this species, probably because of absence of sexual propagation.

##### Notes.

Apart from a single specimen (reared from *Leucoma salicis* probably in Russia, with its mummy present) in poor condition in NMS, no males of *Aleiodes pallidator* have been seen; examined males identified by J. Papp or V.I. Tobias as *Aleiodes pallidator* belong to *Aleiodes gastritor* (Thunberg) s. lat. or to a species near *Aleiodes abraxanae* with darkened pterostigma, black stemmaticum and widened hind femur (e.g. females reported by Papp and Rezbanyai-Reser (1996, 1997)). The lectotype of *Ichneumon pallidator* has 53 antennal segments and the ocelli somewhat larger than OOL. The holotype of *Rhogas ochraceus* has the ocelli nearly twice OOL. Papp (1985) synonymised *Aleiodes apiculatus* (Fahringer, 1932) with *Aleiodes pallidator*, but we consider it to be a valid species.

#### Aleiodes pictus

Taxon classificationAnimaliaHymenopteraBraconidae

(Herrich-Schäffer, 1838)

Figs 272–273 , 274–284

Rogas pictus Herrich-Schäffer, 1838: 156 (type series lost); Shenefelt 1975: 1171 (as synonym of Aleiodes circumscriptus).
Aleiodes pictus ; Papp 1991: 113 (as synonym of Aleiodes circumscriptus).

##### Type material.

Neotype of *Aleiodes pictus* here designated, ♀ (NMS), “Lower **Austria**, Raglitz, J. Connell, [ex] *Camptogramma bilineata*, [coll.] 29.iii.2011, mum. 4.iv.[20]11, em. 29.iv.[20]11, [J. Connell reference number] XI 2.05.05 ♀4, died ca. 10.vii.2011”. “Voucher: BFW Sparkling Science Schwarzes C”.

##### Additional material.

*Austria, British Isles (*England V.C.s 3, 15, 16, 20, 22, 23, 28, 29, 30, 31, 40, 59, 65, 69; *Scotland: V.C.s 77, 83, 84, 88, 89, 96, 101, 105, 111; **Ireland**: “Westport”), *Bulgaria, Czech Republic, *Finland, *France, Germany, Gibraltar (British territory), *Greece, Hungary, *Iceland, *Italy, Netherlands (DR: Drijber, LI: St. Pietersberg, ZH: Den Haag; Meijendel; Goeree; Ouddorp), *Norway, *Poland, *Portugal, *Romania, *Russia, *Serbia, *Slovakia, Spain, Sweden, Turkey. Specimens in NMS, BMNH, OUM, RMNH, ZSSM, SDEI, BZL, ZISP, CMIM, MSC, CNC, UWIM, JLC, H. Schnee collection, H. Haraldseide collection, World Museum Liverpool.

##### Molecular data.

MRS518 (Austria KU682261, CO1), MRS549 (Austria KU682241, CO1), MRS556 (Austria KU682242, CO1), MRS719 (Austria KU682245, CO1), MRS784 (Austria KU682251, CO1), MRS785 (Austria KU682252, CO1).

##### Biology.

This is a plurivoltine parasitoid, abundant in grassland habitats, of larvae of both geometrids and noctuids feeding in low vegetation, overwintering as a small larva in that of the host. Mummy made low down, more or less in concealment, brown and not swollen (Fig. 273). Specimens (in NMS unless stated otherwise) reared from wild-collected larvae of the larentiine geometrids *Camptogramma bilineata* (Linnaeus) (78:2; J. Connell [77 specimens from Austria resulting from a long term survey], A.R. Cronin), *Epirrhoe alternata* (Müller) (1; G.M. Haggett), *Lithostege griseata* (Denis & Schiffermüller) (3:1; G.M. Haggett), *Xanthorhoe fluctuata* (Linneaus) (3:2; G.M. Haggett, G.E. King), and from the diverse range of noctuids *Hoplodrina ambigua* (Denis & Schiffermüller) (2:1; J. Connell/Austria), *Hoplodrina* sp. (*blanda* (Denis & Schiffermüller), *octogenaria* (Goeze) and *superstes* (Ochsenheimer) co-occurring but indistinguishable when small) (17:1; J. Connell/Austria), *Agrotis exclamationis* (Linnaeus) (1; M.R. Shaw/France); *Noctua fimbriata* (Schreber) (1; J. Connell/Austria); *Xestia xanthographa* (Denis & Schiffermüller) (1; J. Connell/Austria), *Phlogophora meticulosa* (Linnaeus) (1; M.R. Shaw); *Syngrapha interrogationis* (Linnaeus) (1 ZMUO; J. Itämes/Finland). The Austrian material reared by J. Connell resulted from a prolonged survey of grassland caterpillars at a single site. Experimental cultures (specimens in NMS) as follows: *Epirrhoe alternata* 6:27\1\\1+0, *Xanthorhoe fluctuata* 2:45\40\\25+14 [other females even more willing to oviposit (6: 86\84) but cultures lost to disease], *Xanthorhoe montanata* (Denis & Schiffermüller) 3:15\15\\8+2, *Xestia xanthographa* 2:2\0\\-, *Xestia sexstrigata* (Haworth) 2:3\1\\0+1, *Noctua fimbriata* 1:2\0\\- [This female was then boxed with 10 host larvae for 5 days: 6 hosts pupated; the other 4 died and on dissection were found to contain living parasitoid larvae], *Diarsi rubi* (Vieweg) 3:37\18\\0+12, *Phlogophora meticulosa* 2:17\0\\-. The males are aggressive and rather indiscriminate in courtship, seeming as interested in females of *Aleiodes nigriceps* as their own species, but the *Aleiodes nigriceps* females always repelled them successfully. Males of *Aleiodes pictus* are, however, somewhat less interested in females of *Aleiodes leptofemur*. Mating trials between specimens from *Hoplodrina* spp. and *Camptogramma bilineata* (all from the same site in Austria) repeatedly indicated that females from both series were less willing to mate with males from the other than with males reared from the same host as themselves; however, several cooperative matings (that then seemed normal, though of often shorter duration) were obtained and (from very limited trials) no difference was seen in behaviour towards hosts by females of different host origin (but *Hoplodrina* larvae were not available for experiments). However, the possibility that specimens from *Hoplodrina* (which differ slightly in their morphology, particularly colour) are at least some way towards representing a genetic isolate cannot be ruled out, as most of the experimental results involved females from *Camptogramma bilineata* (but also the female from *Noctua fimbriata* whose behaviour was essentially the same). A host range encompassing both Geometridae and Noctuidae is unusual in the genus *Aleiodes*, but the species is evidently rather specialised to a narrow range of taxa in each family. The adult flight period is May to September in Britain.

**Figures 272–273.**
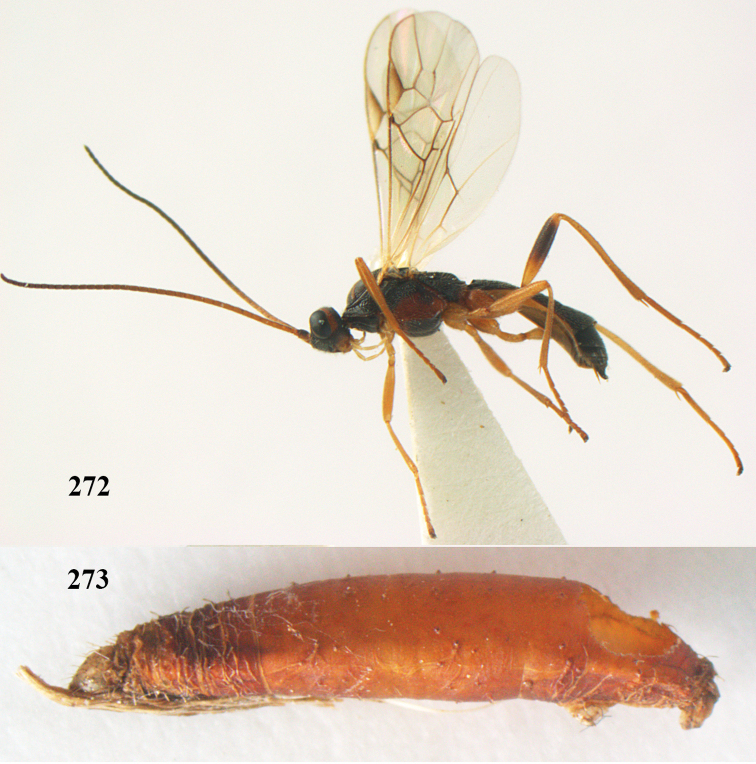
*Aleiodes pictus* (Herrich-Schäffer), ♀, neotype. **272** habitus lateral **273** mummy of *Camptogramma bilineata* (Linnaeus).

##### Diagnosis.

Antennal segments of ♀ (35–)36–40, of ♂ (36–)37–41; pale area of orbita of ♀ rather wide (Figs 281–282); mesopleuron with some rugulae; mesosternum almost always strongly darkened or black and usually sharply defined; width of hypoclypeal depression 0.3–0.4 × minimum width of face (Fig. 280); OOL 1.3 × diameter of posterior ocellus; mesoscutum antero-laterally and pronotum medio-anteriorly very often brownish yellow; length of first tergite of ♀ 1.0–1.1 × its apical width; second tergite often less wide than in *Aleiodes nigriceps* (Fig. 276); fore and hind tarsi comparatively slender (Figs 277–278); pterostigma yellow anteriorly; middle to distal third of hind femur partly or entirely dark brown; mummy not swollen, usually light brown (Fig. 273).

**Figures 274–284.**
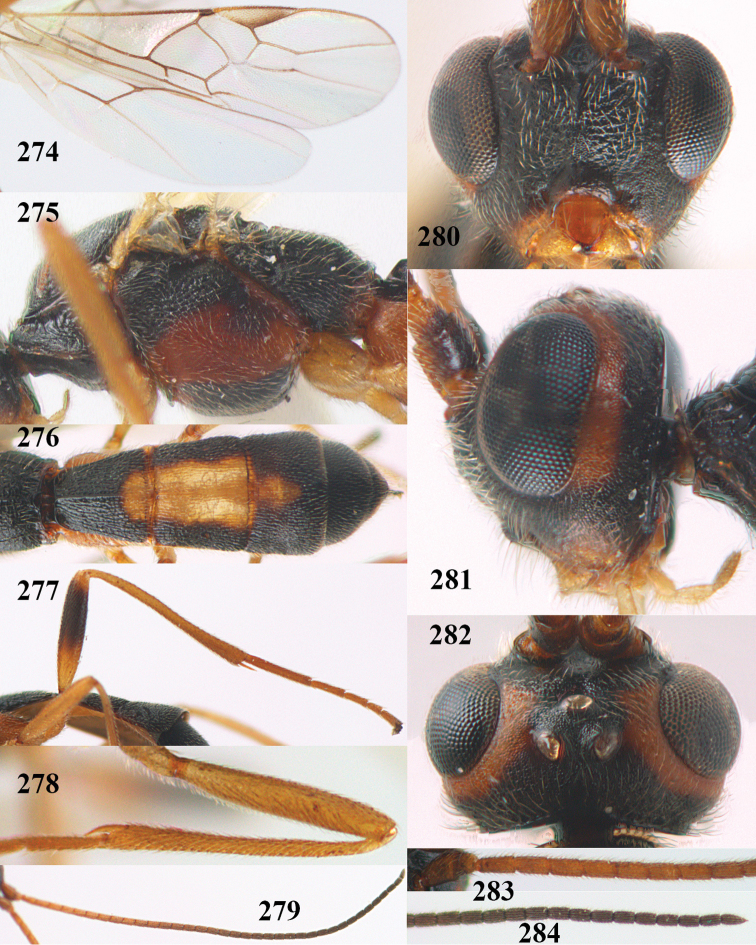
*Aleiodes pictus* (Herrich-Schäffer), ♀, neotype. **274** wings **275** mesosoma lateral **276** propodeum and metasoma dorsal **277** hind leg lateral **278** fore femur lateral **279** antenna **280** head anterior **281** head lateral **282** head dorsal **283** basal segments of antenna **284** apical segments of antenna.

##### Description.

Neotype, ♀, length of fore wing 4.6 mm, of body 4.9 mm.

*Head.* Antennal segments 37 (right) and 38 (left), length of antenna 1.2 × fore wing, its subapical segments about 1.6 × as long as wide; frons granulate and some rugulae, with satin sheen; OOL 1.3 × diameter of posterior ocellus (POL equal to ocellus); vertex superficially granulate, with satin sheen; clypeus distinctly convex and largely nearly smooth; ventral margin of clypeus thick and depressed (Fig. 281); width of hypoclypeal depression 0.3 × minimum width of face (Fig. 280) and face mainly granulate-coriaceous with some rugulae medially; length of eye 2.8 × temple in dorsal view and temple gradually narrowed behind eye (Fig. 282); occiput behind stemmaticum granulate-coriaceous and occipital carina absent medio-dorsally and interruption slightly less than width of stemmaticum (Fig. 282); clypeus partly above lower level of eyes (Fig. 280); length of malar space 0.4 × length of eye in lateral view; eyes moderately protruding (Figs 280–282).

*Mesosoma.* Mesoscutal lobes finely granulate-coriaceous, with satin sheen, but medio-posteriorly rugose; notauli narrow, moderately impressed and finely crenulate, but posteriorly merging in rugose area; prepectal carina narrow lamelliform and reaching anterior border; precoxal area of mesopleuron coriaceous and with some rugae medially (Fig. 275); mesopleuron above precoxal area (except small nearly smooth and shiny speculum) antero-dorsally distinctly rugose and granulate-coriaceous; medially metapleuron granulate, matt; mesosternal sulcus narrow and rather deep, but posteriorly shallow and with a fine carina; mesosternum rather angulate posteriorly; scutellum nearly flat, superficially coriaceous and only antero-laterally carinate; propodeum rather flat and coriaceous with rugae, median carina nearly complete, without tubercles.

*Wings.* Fore wing: r 0.3 × 3-SR (Fig. 274); 1-CU1 horizontal, 0.4 × as long as 2-CU1; r-m 0.7 × 2-SR, and 0.4 × 3-SR; second submarginal cell slender (Fig. 274); cu-a slightly oblique, not parallel with CU1b, straight; 1-M slightly curved posteriorly. Hind wing: apical half of marginal cell somewhat widened apically (Fig. 274); 2-SC+R subquadrate; m-cu medium-sized, unpigmented; M+CU:1-M = 50:39; 1r-m 0.6 × as long as 1-M.

*Legs.* Tarsal claws setose; hind coxa granulate-coriaceous, matt; hind trochantellus 2.5 × longer than wide; length of fore and hind femora 6.1 and 4.9 × their width, respectively (Figs 277–278); hind femur pimply; inner apex of hind tibia without comb; length of inner hind spur 0.3 × hind basitarsus.

*Metasoma.* First tergite 1.1 × as long as wide posteriorly, convex, but posteriorly flattened; first and second tergites densely longitudinally rugose; second tergite slenderer than is usual in *Aleiodes nigriceps* (Fig. 276), with distinct median carina, with satin sheen; medio-basal area of second tergite absent; second suture distinctly impressed and crenulate; third tergite largely coriaceous (but anteriorly rugose) and medially 0.7 × as long as second tergite (Fig. 276); remainder of metasoma largely nearly smooth and rather shiny; fourth and apical half of third tergite without sharp lateral crease; ovipositor sheath moderately setose.

*Colour.* Black or brownish black; antenna brown, but scapus and pedicellus dorsally and laterally and apical half of antenna dark brown; malar space (except near eye), palpi, tegulae, fore and middle coxae, trochanters and trochantelli, first tergite medio-posteriorly, second tergite medially and third tergite narrowly medially pale yellowish (Fig. 276); orbita posteriorly and dorsally widely brownish yellow (Figs 281–282) and remainder of head black; dorso-posteriorly pronotum brown; mesoscutum with notaulic and medio-posterior area brownish yellow; mesosternum narrowly anteriorly behind prepectal carina, ventral half of mesopleuron and posteriorly orange brown; hind femur (except basally and apex) dark brown (Fig. 272); scutellum dark reddish brown medially; veins (except dark brown veins 1-SR, 1-M, r and CU1) brown; pterostigma pale yellowish, but posteriorly somewhat darkened (Figs 272, 274); wing membrane subhyaline.

*Variation.* Length of fore wing 3.8–4.6 mm; antennal segments of ♀ 34(1), 35(5), 36(27), 37(52), 38(64), 39(38), 40(9) and of ♂ 36(9), 37(22), 38(39), 39(34), 40(18), 41(4); vein r of fore wing 0.4 × vein 3-SR; clypeus 0.3–0.4 × as wide as face; length of first tergite of ♀ 1.0–1.1 × its apical width; pterostigma medially and anteriorly yellow; malar space largely dark brown to largely pale yellow; pronotum medio-anteriorly and scutellum brownish yellow or dark brown; medially metapleuron black or orange brown.

##### Note.

Males have on average about one more antennal segments than females. In some populations pale specimens (including hind femur and much of face) occur that superficially resemble *Aleiodes leptofemur*, but can be distinguished by their more robust femora. For further notes see *Aleiodes nigriceps*.

#### Aleiodes praetor

Taxon classificationAnimaliaHymenopteraBraconidae

(Reinhard, 1863)

Figs 285 , 286–287 , 288–301

Rogas praetor Reinhard, 1863: 264; Shenefelt 1975: 1244; Tobias 1986: 78 (transl.: 128).
Aleiodes praetor ; Papp 1991: 73; Belokobylskij et al. 2003: 398.
Neorhogas luteus Szépligeti, 1906: 606; Shenefelt 1975: 1205; Papp 1977: 115 (as synonym of Aleiodes praetor); 1991: 73; 2004: 215 (lectotype designation); Chen and He 1997: 37.

##### Type material.

Holotype of *Aleiodes praetor* ♀ (MNHN) from France (“Mout. [= Moutiers, Savoie]”, “Moutiers”, “Muséum Paris, 1867, Coll. O. Sichel”, “*Rogas praetor* Rhd.”) and lectotype of *Neorhogas luteus* ♂ (MTMA) from Serbia examined.

##### Additional material.

**Austria, Belgium, British Isles** (**England**: V.C.s 5, 11, 16, 17, 19, 20, 21, 22, 24, 30, 31, 34, 38, 62, 64), *Bulgaria, Croatia, Finland, **France, Germany**, **Hungary**, **Netherlands** (GE: Heerde; LI: Stein, Epen, Tegelen; NH: Naardermeer; UT: Bilthoven, Leersum; ZH: Melissant, Oostkapelle), **Serbia, Spain, Sweden, Switzerland**. Specimens in NMS, MNHN, BMNH, CMIM, OUM, RMNH, FMNH, NRSM, MTMA, S. Dodd collection, P. McMullen collection, WAE, UWIM.

##### Molecular data.

MRS067 (England KM067256/KU682219, CO1 + EU854334, 28S), MRS654 (Bulgaria HQ551265/KU682244, CO1).

##### Biology.

**Figure 285.**
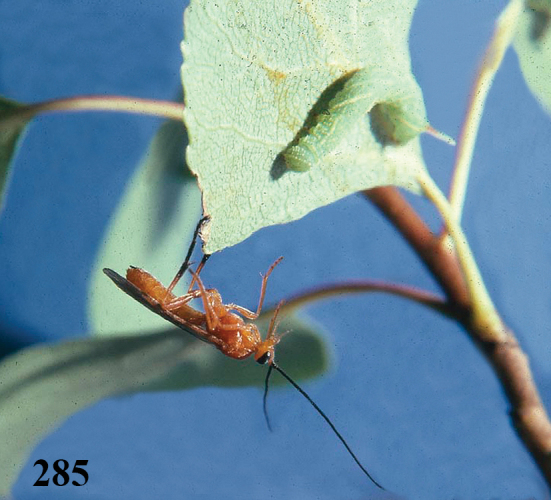
*Aleiodes praetor* (Reinhard), ♀, hanging from a leaf edge.

*Aleiodes praetor* is a univoltine parasitoid of at least some arboreal Sphingidae, and overwinters in the host mummy. Reared specimens seen were from *Lathoe populi* (Linnaeus) (1 CMIM; C. Morley), *Mimas tiliae* (Linnaeus) (2 OUM, 1 NMS, 1 BMNH, 1 RMNH; J.C. Fraser, J. Koorneef, R.A. Softly). In Britain the flight period is from late June through August. A series of males was reared in culture in both *Lathoe populi* and *Mimas tiliae* parented by a virgin female from *Mimas tiliae*. The female was often seen hanging from a leaf edge by only a few of her legs (Fig. 285). Most ovipositions, into late first instar and more particularly second instar hosts, occurred in a rearing cage overnight and were unobserved; however two of these remarkable occasions were witnessed (into late first instar *Lathoe populi*). The host, which rests and feeds from the under surface of the leaf, was approached and repeatedly touched with the outstretched extreme tips of the antennae, causing the host to twitch more or less violently from side to side. It is noteworthy that the apical segment of the antenna in this species has a well-pronounced nipple-like tip (Fig. 297). When this reaction wore out, the parasitoid suddenly jumped on the host and rapidly inserted her ovipositor, with the metasoma scarcely curled; she then immediately straightened her body and released her footing completely so that she hung from the host with only her ovipositor touching it, and all legs completely free of any support. After 30 seconds she jerked free, and took flight as she fell from the host, which was apparently not paralysed to any extent although it was quiescent during oviposition. The extraordinary oviposition behaviour is clearly facilitated by the unusual flange and teeth at the apex of the ovipositor (Fig. 295), and may be completely constraining: otherwise suitable hosts on the floor of containers (i.e. lacking a drop) were consistently just walked over or otherwise ignored. The lack of paralysis ensures that the host maintains its footing, without risk that it would fall and be unable to rediscover its food source. The mummy appears to be highly adapted for a lengthy persistence in crevices in tree bark. It is very hard, matt, and predominantly light greyish brown in colour but with darker transverse variegation and sometimes small dark grey dorsal patches (Fig. 287). The parasitoid occupies abdominal segments (4–)5–8 which become thinly lined with silk and weakly arched. This structure is strongly stuck down ventrally at about the fourth abdominal segment, with the anterior part of the host becoming physically detached at an oblique angle by the action of the parasitoid larva. In captivity the stricken hosts sought refuge in paper tissues at the base of the rearing boxes, where mummies were made glued firmly in surface folds, and, despite Morley’s (1916) finding a mummy on a *Populus* leaf, it is clear that the penultimate instar host larva is normally induced to descend and find a crevice before perishing; indeed, a partly grown *Mimas tiliae* larva which was collected on the bark of a *Tilia* tree was mummified within a few hours (R.A. Softly, personal communication). Before the widespread use of UV lights by lepidopterists *Aleiodes praetor* was rarely collected in Britain; now, however, specimens turn up quite regularly in light traps.

**Figures 286–287.**
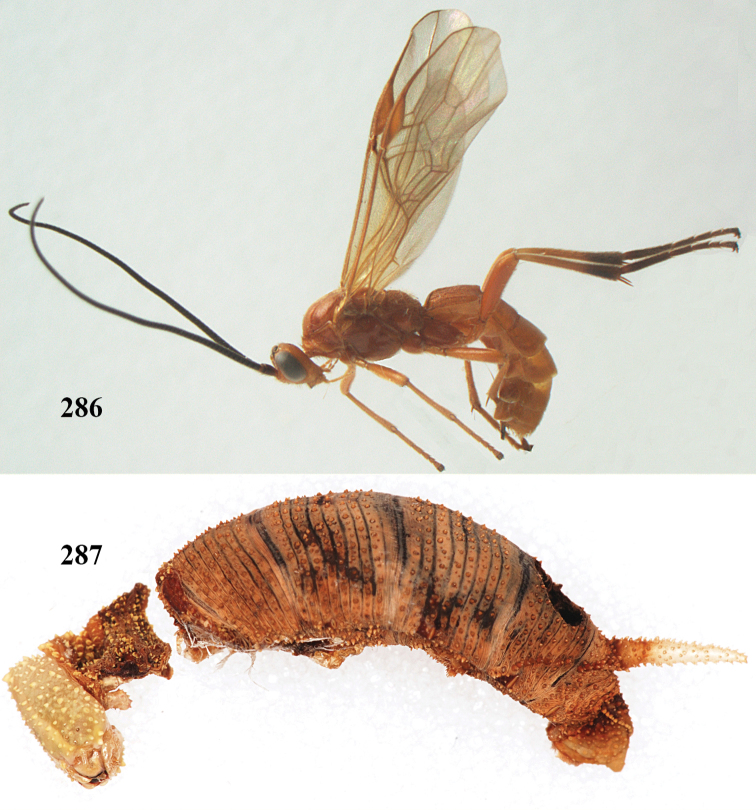
*Aleiodes praetor* (Reinhard), ♀, Netherlands, Epen. **286** habitus lateral **287** mummy of *Mimas tiliae* (Linnaeus) from England.

**Figures 288–301.**
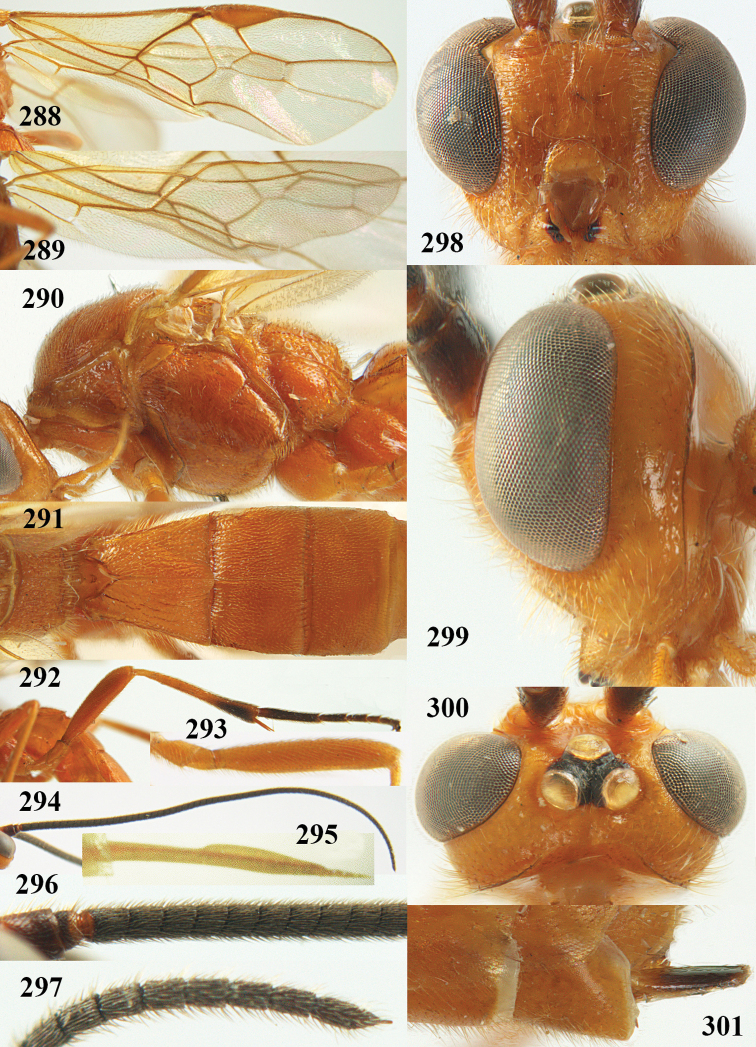
*Aleiodes praetor* (Reinhard), ♀, Netherlands, Epen. **288** fore wing **289** hind wing **290** mesosoma lateral **291** propodeum and metasoma dorsal **292** hind leg lateral **293** fore femur lateral **294** antenna **295** ovipositor lateral **296** basal segments of antenna **297** apical segments of antenna **298** head anterior **299** head lateral **300** head dorsal **301** hypopygium and ovipositor sheath lateral.

##### Diagnosis.

Large yellowish brown species with antennal segments of female 67–77 and of male 62–75; OOL 0.3 × diameter of posterior ocellus; lateral carina of scutellum strong and lunula rather narrow; marginal cell of hind wing narrowed near basal 0.6 and slightly widened apically (Fig. 289); inner apex of hind tibia with weak and indistinct comb; tarsal claws yellowish setose; ovipositor sheath largely glabrous (except apically and ventrally; Fig. 301); ovipositor with small teeth ventrally and with wide dorsal flange (Fig. 295); length of fore wing 8–10 mm; parasitoid of Sphingidae.

##### Description.

Redescribed ♀ (RMNH) from Naardermeer, length of fore wing 8.7 mm, of body 8.8 mm.

*Head*. Antennal segments of ♀ 72, with many tyloids and apex of subbasal segments oblique (Fig. 296), length of antenna 1.2 × fore wing, its subapical segments distinctly longer than wide and apical segment with long spine (Fig. 297); frons narrow, rather flat and largely micro-granulate; OOL 0.3 × diameter of posterior ocellus and micro-sculptured; vertex flat, micro-sculptured and shiny; clypeus convex, micro-granulate and near lower level of eyes; ventral margin of clypeus not differentiated (Fig. 299); width of hypoclypeal depression 0.5 × minimum width of face (Fig. 298); face micro-sculptured and partly transversely rugulose; length of eye 3.2 × temple in dorsal view (Fig. 300); occiput behind stemmaticum superficially sculptured; length of malar space 0.2 × length of eye in lateral view; occipital carina strong, but medio-dorsally absent; eyes distinctly protruding (Figs 298–300).

*Mesosoma*. Mesoscutal lobes densely punctate, micro-sculptured and shiny; prosternum rather large and distinctly concave; prepectal carina complete, distinct; precoxal area of mesopleuron with some striae medially; mesopleuron above precoxal area strongly shiny, punctate medio-posteriorly and remainder smooth (Fig. 290); metapleuron punctate dorsally and rugose ventrally; scutellar sulcus wide, deep and with 7 carinae; scutellum flat, densely punctate, with striae medio-posteriorly and lateral carina largely present and lunula narrow; propodeum convex, dorsal face about as long as posterior face, densely rugose, tuberculate protruding latero-dorsally (Fig. 290), propodeal spiracle large and median carina of propodeum complete and regular.

*Wings*. Fore wing: r 0.6 × 3-SR (Fig. 288); 1-CU1 nearly horizontal, slender, 0.2 × 2-CU1; r-m 0.4 × 3-SR and not pigmented; second submarginal cell rather long (Fig. 288); cu-a inclivous, curved posteriorly; 1-M straight posteriorly. Hind wing: marginal cell subparallel-sided basally, constricted near basal 0.7 and its apical width nearly equal to width at level of hamuli (Fig. 289); 2-SC+R short; m-cu absent; M+CU:1-M = 33:16; 1r-m 1.1 × as long as 1-M.

*Legs*. Tarsal claws yellowish setose; hind coxa punctate and micro-sculptured dorso-basally and remainder largely smooth and punctulate; hind trochantellus ventrally twice as long as wide; length of fore femur, hind femur and basitarsus 6.3, 4.2 and 7.8 × their width, respectively (Figs 292–293); length of inner hind spur 0.35 × hind basitarsus; inner apex of hind tibia with indistinct weak comb.

*Metasoma*. First tergite as long as wide apically (Fig. 291); first and second tergites densely and coarsely longitudinally rugose, with distinct median carina, reduced near apex of second tergite; medio-basal area of second tergite absent; length of second tergite 0.7 × its basal width; second suture deep and distinctly crenulate; third tergite 0.9 × as long as second tergite, anterior half largely densely and finely punctate and remainder of metasoma largely smooth and depressed; fourth and apical half of third tergite without sharp lateral crease; ovipositor sheath largely glabrous (except apically and ventrally; Fig. 301); ovipositor with small teeth ventrally and with wide dorsal flange (Fig. 295).

*Colour*. Yellowish brown; antenna (but scapus brownish basally), stemmaticum, apical third of hind tibia (except spurs) and hind tarsus largely, black; base of hind tibia pale yellowish; pterostigma and veins brownish yellow; wing membrane largely subhyaline, but basally slightly pigmented and near veins 1-SR and 1-M slightly infuscate.

*Variation.* Antennal segments of European ♀ 67(2), 68(7), 69(7), 70(7), 71(3), 72(4), 73(4); of ♂ 62(3), 63(5), 64(5), 65(5), 66(1), 67(1); males have fifth–seventh tergites moderately setose; vein m-cu of fore wing sometimes slightly curved and gradually merging into 3-CU1; precoxal sulcus entirely smooth or with some striae; scapus and pedicellus partly yellowish brown or entirely black.

##### Notes.

European males have approximately four fewer antennal segments than females. Antenna of possibly conspecific Chinese and Japanese females consists of 70–77 segments and of males 62–75 segments and they have the pterostigma darker compared to the veins below it.

#### Aleiodes reticulatus

Taxon classificationAnimaliaHymenopteraBraconidae

(Noskiewicz, 1956) stat. rev.

Figs 302–304 , 305–315

Rhogas reticulatus Noskiewicz, 1956: 176 (examined).
Aleiodes reticulatus ; Shenefelt 1975: 1181; Papp 1991: 96 (as synonym of Aleiodes arcticus).

##### Type material.

Holotype, ♀ (PAN), “[**Poland**], 15/1 [19]48 *Itame fulvaria* Vill. 1.II.[19]49”, “*Rhogas* - ♂ *reticularis* [sic] Nosk.”, “Holotyp. (lgz. pnedui)”, “Holotypus ♀ % *Rhogas reticulatus* Nosk. 1956. Papp 1983”. Paratypes: 1 ♀ (glued on same card as holotype, with one emerged mummy of geometrid (compatible with *Macaria* sp.) with same labels and “*Aleiodes* ♀ *arcticus* Th. det. Papp J., 1983”, “Syntypus *Rhogas reticulatus* Noskiewicz 1954”; 2 ♂ “15/1/ [19]48 Karczewski Jędrzejów 15.xii.48”, “*Rhogas* - ♂ *reticularis* [sic] Nk”, “Allotypus *Rhogas* ♂ *reticulatus* Nosk. 1956. Papp 1983”, “*Aleiodes* ♂ *arcticus* Th. det. Papp J. 1983”, “Syntypus *Rhogas reticulatus* Noskiewicz, 1954”.

##### Additional material.

2 ♀, 2 ♂ (3 ZISP, 1 NMS), *Belarus, Zubky, 120 km W of Minsk, ex *Macaria wauaria* on *Ribes nigrum*, em. 1–8.v.1984 (Silvanovich); 1 ♀ (SDEI), *Germany, Mecklenburg, Fürstenberg, 21.v.1888 (Konow); 1 ♂ (SDEI) Germany, Mecklenberg, Kalkhof, 30.iv.1890 (F.W. Konow); 1 ♀ (NMS) Germany, Saxony, Tharandt, Fichtenwald, 18.v.1980 (Walter); 2 ♂ (NMS), **Poland**, Pomeranian, Czarne, ex *Macaria brunneata* on *Vaccinium myrtillus*, coll. 24.v.2014, mummies 27.v.2014, em. 27.iii.2015 (M.R. Shaw); 1 ♂ (ZISP) *Russia, Voronezezhskij zapovednik ex geometrid [host mummy compatible with *Macaria wauaria*] on *Ribes nigrum*, 18.iv.1950 (Donvar); 1 ♀ (ZISP) Russia, Rostchino, NW of St Petersburg, 15.vi.1966 (V.I. Tobias). In addition several mummies were obtained from *Macaria brunneata* larvae collected from *Vaccinium myrtillus* in Polish conifer forests in v. 2014, both at the type locality (Kielce: Jędrzejów, Lasków forest), and also at Ruciane-Nida (Warmian-Masurian) and Biebrza (Podalaskie), but the adults failed to develop (mummies in NMS).

##### Molecular data.

MRS808 (Poland KU682262, CO1).

##### Biology.

The type series was reared in Poland from the ennomine gemetrid *Macaria brunneata* (Thunberg) (3 ♀, 3 ♂) feeding on *Vaccinius myrtillus* - and supposedly also *Arctia caja* (Linnaeus) (1 ♂) collected from the same plant, but we discount that as a presumed error (the specimen can no longer be found in PAN, its supposed depository). It is not surprising to add *Macaria wauaria* (Linnaeus) (4:1 [Belarus]; Silvanovich) and another possibly from this host (Russia) to the known host range, especially as these two *Macaria* species both overwinter in the egg stage (unlike many others). From material recently obtained from *Macaria brunneata* in the type locality and five other sites in Poland (M.R. Shaw), it is clear that *Aleiodes reticulatus* is a regular univoltine parasitoid of *Macaria brunneata* feeding on *Vaccinium myrtillus* growing as understory in conifer forests especially on infertile sandy soils (on one of the German specimens “Fichtenwald” translates as spruce forest), flying in early spring (April and May) which is no doubt why it has remained poorly understood until now. The small mummy (Fig. 303) forms at about the end of May and is firmly glued to a twig of the foodplant well below the crown, or frequently on twigs and conifer needles in the litter. It is brownish grey, with a dark brown posterodorsal patch corresponding to the site of eventual adult emergence, and rather short, broad and dorsally elevated. After summer diapause *Aleiodes reticulatus* overwinters as a prepupa (without defaecation but with well-formed eyes: ascertained by opening a mummy with living contents in December).

**Figures 302–304.**
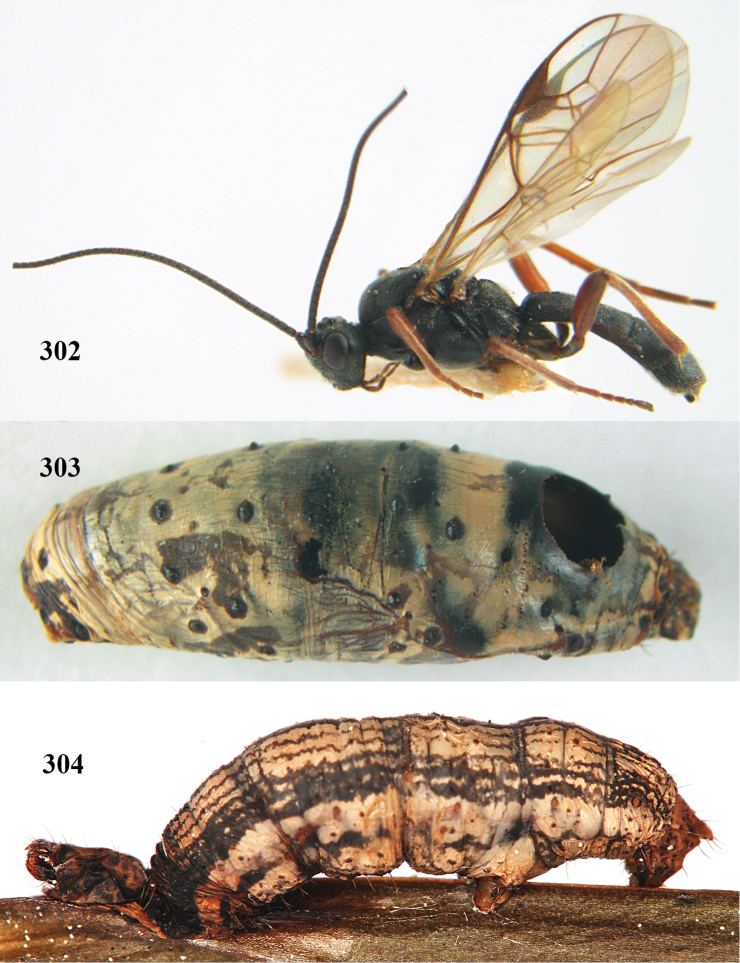
*Aleiodes reticulatus* (Noskiewicz), ♀, Russia, Rostchino, but **304** from Poland. **302** habitus lateral **303** mummy of cf. *Macaria wauaria* (Linnaeus) **304** mummy of *Macaria brunneata* (Thunberg) forming, at end will resemble 303.

##### Diagnosis.

**Figures 305–315.**
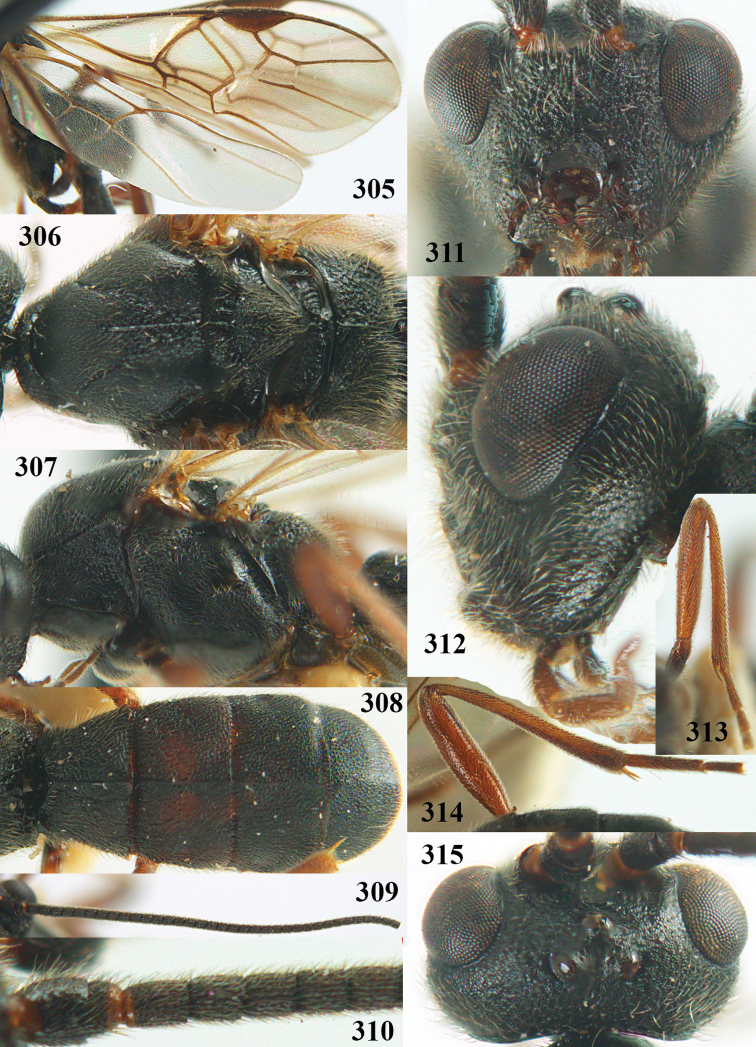
*Aleiodes reticulatus* (Noskiewicz), ♀, Russia, Rostchino. **305** wings **306** mesosoma dorsal **307** mesosoma lateral **308** metasoma dorsal **309** antenna (tip absent) **310** basal segments of antenna **311** head anterior **312** head lateral **313** fore femur lateral **314** hind leg lateral **315** head dorsal.

Maximum width of hypoclypeal depression 0.4 × minimum width of face (Fig. 311); OOL twice diameter of posterior ocellus; mesoscutum, orbita and malar space black; precoxal sulcus granulate; trochanters, trochantelli and pterostigma largely black(ish); mesoscutum with a fine longitudinal carina on mesoscutum medio-posteriorly and more or less anteriorly, but sometimes absent; apical half of marginal cell of hind wing parallel-sided or slightly widened; vein M+CU1 of fore wing apically above level of vein 2-CU1 (Fig. 305); vein r of fore wing 0.9–1.1 × as long as vein 3-SR (Fig. 305); vein 1-SR of fore wing slightly angled with vein 1-M; all femora and tibiae dark reddish brown; fore and hind femora moderately stout (Figs 313–314); fourth metasomal tergite curved posteriorly in dorsal view (Fig. 308), its lateral crease distinct and following tergites more or less retracted (Fig. 302); length of fore wing 3.9–4.7 mm.

##### Description.

Redescribed ♀ (NMS) from Zubky (Belarus), length of fore wing 4.7 mm, of body 5.1 mm.

*Head.* Apical antennal segments missing, remaining 37 segments, length of antenna about as long as fore wing, its subbasal segments about 1.2 × as long as wide; frons mainly superficially granulate and with some rugae anteriorly, weakly shiny; OOL twice diameter of posterior ocellus and granulate as is vertex, with satin sheen; clypeus weakly convex, narrow and coriaceous; ventral margin of clypeus thick and depressed (Fig. 312); width of hypoclypeal depression 0.4 × minimum width of face (Fig. 311) and face mainly coriaceous with some rugulae dorsally and long setae; length of eye 1.1 × temple in dorsal view and temple sub-parallel-sided behind eye (Fig. 315); occiput behind stemmaticum granulate and occipital carina nearly complete ventrally and narrowly interrupted dorsally; clypeus near lower level of eyes (Fig. 311); length of malar space 0.6 × height of eye in lateral view; eyes somewhat protruding (Figs 311–312, 315).

*Mesosoma.* Mesoscutal lobes largely granulate-coriaceous, matt and medio-posteriorly rugose, middle lobe with a complete longitudinal carina, but weakly developed anteriorly (Fig. 306); notauli narrow, rather shallow and finely crenulate; prepectal carina narrow lamelliform medio-ventrally, not reaching anterior border of mesopleuron; precoxal area of mesopleuron granulate; mesopleuron above precoxal area (except large smooth and shiny speculum) granulate, but dorsally finely rugose; medially metapleuron granulate and with some rugae, rather shiny; mesosternal sulcus narrow and rather shallow; mesosternum rounded posteriorly; scutellum moderately convex, shallowly impressed medio-anteriorly, mainly granulate and non-carinate laterally; propodeum rather directly lowered posteriorly and granulate-rugose, median carina complete, without tubercles.

*Wings.* Fore wing: r nearly as long as 3-SR (Fig. 305); 1-CU1 oblique, 0.3 × as long as 2-CU1; r-m 0.8 × 2-SR, and 0.9 × 3-SR; second submarginal cell rather short (Fig. 305); vein M+CU1 of fore wing apically above level of vein 2-CU1 (Fig. 305); vein 1-SR of fore wing slightly angled with vein 1-M; cu-a slightly oblique, not parallel with CU1b; 1-M nearly straight posteriorly. Hind wing: apical half of marginal cell slightly widened apically (Fig. 305); 2-SC+R short and longitudinal; m-cu present, pigmented; M+CU:1-M = 25:14; 1r-m slightly oblique and 0.6 × as long as 1-M.

*Legs.* Tarsal claws setose; hind coxa granulate-coriaceous, with satin sheen and nearly reaching apex of first tergite; hind trochantellus 1.8 × longer ventrally than wide; length of fore and hind femora 5.3 and 4.5 × their width, respectively (Figs 313–314); inner apex of hind tibia without comb; length of inner hind spur 0.4 × hind basitarsus.

*Metasoma.* First tergite 0.7 × as long as wide posteriorly, convex and latero-posteriorly non-lamelliform; first–second tergites finely and densely irregularly rugulose and with median carina (Fig. 308); medio-basal area of second tergite absent; second suture medium-sized, deep and distinctly crenulate; third tergite with median carina (but obsolescent posteriorly), third–fourth tergites very finely rugulose-coriaceous; fourth tergite convex medially and apically; fourth tergite with sharp lateral crease; remainder of metasoma largely retracted; ovipositor sheath truncate apically and moderately setose.

*Colour.* Black (including coxae); palpi basally, tegulae, pterostigma, veins, trochanters, middle and hind femora dorso-apically and more or less trochantelli dark brown; remainder of palpi and legs brown; wing membrane slightly infuscate, especially near basal veins.

*Variation*. Antennal segments of ♀ 40(1), 41(1), 43(1), 44(1); of ♂ 40(1), 42(1), 43(3), 44(3); male has shape of head just like the examined specimens of true *Aleiodes arcticus* but females have the temple slightly longer and more narrowed; mesoscutum black but one ♀ vaguely brownish near origin of notauli; pale parts of legs brown or orange brown; mesopleuron black or more or less brownish.

##### Note.

This species is very close to *Aleiodes arcticus* but, in addition to small morphological differences, the fact that *Aleiodes reticulatus* is a lowland species while *Aleiodes arcticus* is boreo-alpine is also regarded as significant.

#### Aleiodes ryrholmi sp. n.

Taxon classificationAnimaliaHymenopteraBraconidae

http://zoobank.org/2ED2733A-AA4C-4ECD-9A56-983CA62F5B10

Figs 316 , 317–327

##### Type material.

Holotype, ♀ (NMS, Edinburgh), “**Sweden**: Hr., Sveg, Duvberg, 16.vii.–12.viii.2004, N. Ryrholm, NMSZ 2004.167”, “MRS *Aleiodes* DNA 395”.

##### Molecular data.

MRS395 (Sweden JF962792, CO1).

##### Biology.

Unknown.

##### Diagnosis.

Antennal segments of ♀ about 40, of ♂ unknown; head strongly directly narrowed behind eyes (Fig. 324); OOL 0.6 × diameter of posterior ocellus; length of malar space of ♀ 0.35 × height of eye in lateral view (Fig. 325); palpi mainly dark brown; face superficially rugulose (Fig. 323); occipital carina interrupted dorsally and complete ventrally (Figs 324–325); eye about 4.5 × as long as temple in dorsal view (Fig. 324); face black; POL 0.6 × as wide as diameter of posterior ocellus (Fig. 324); mesoscutum entirely black (Fig. 319); medio-posterior depression of metanotum rather narrow (Fig. 319); mesosternum and precoxal sulcus superficially granulate and with satin sheen; vein 2-CU1 of fore wing about twice vein 1-CU1 (Fig. 317); vein cu-a of fore wing inclivous; vein 1-SR narrow and linearly connected to vein 1-M and vein 1-M straight (Fig. 317); hind femur stout (Fig. 321); pale area of second metasomal tergite narrow (Fig. 320); fourth tergite largely (superficially) coriaceous and shiny; length of fore wing about 4.5 mm. Very similar to *Aleiodes cantherius* (Lyle), but the new species has a much darker body, larger ocelli and superficially sculptured face.

**Figure 316.**
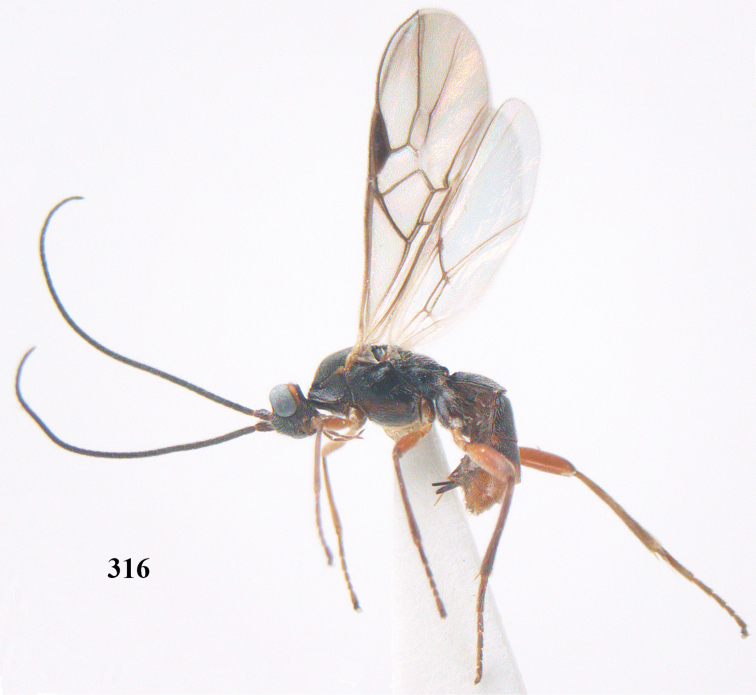
*Aleiodes ryrholmi* sp. n., ♀, holotype, habitus lateral.

**Figures 317–327.**
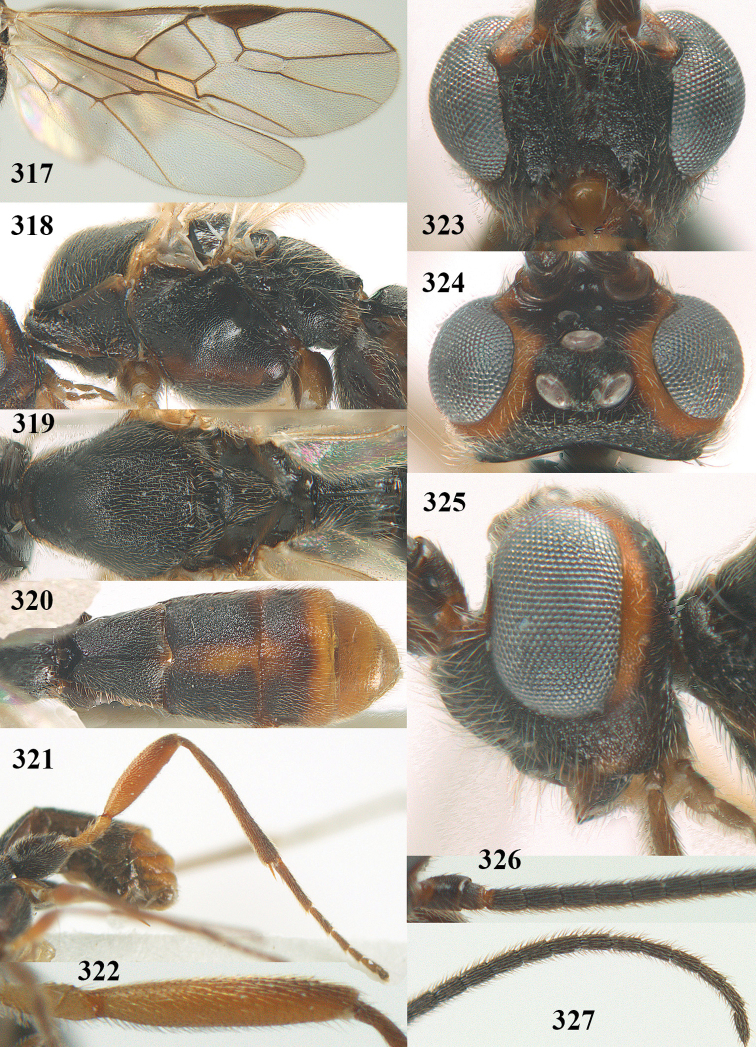
*Aleiodes ryrholmi* sp. n., ♀, holotype. **317** wings **318** mesosoma lateral **319** mesosoma dorsal **320** propodeum and metasoma dorsal **321** hind leg lateral **322** fore femur lateral **323** head anterior **324** head dorsal **325** head lateral **326** basal segments of antenna **327** apical segments of antenna.

##### Description.

Holotype, ♀, length of fore wing 4.4 mm, of body 4.9 mm.

*Head.* Antennal segments 40, length of antenna 1.2 × fore wing, basal segments rather robust (Fig. 326), its subapical segments about 2.3 × as long as wide (Fig. 327) and scapus in lateral view oblique apically; frons granulate and rather shiny; OOL and POL 0.6 and 0.6 × diameter of posterior ocellus, respectively, and granulate; vertex coriaceous, with satin sheen; clypeus rather high, convex, coriaceous, 0.4 × as wide as face; ventral margin of clypeus thick (Fig. 325); width of hypoclypeal depression 0.35 × minimum width of face (Fig. 323) and face mainly granulate, dorsally superficially rugulose; length of eye 4.5 × temple in dorsal view and temple directly narrowed behind eye; occiput behind stemmaticum mainly granulate-coriaceous and occipital carina widely interrupted medio-dorsally and nearly complete ventrally (Fig. 325); clypeus above lower level of eyes (Fig. 323); length of malar space 0.35 × height of eye in lateral view; eyes protuberant (Figs 323–325).

*Mesosoma.* Mesoscutal lobes very finely granulate-coriaceous, with satin sheen, but medio-posteriorly rugulose; notauli narrow, shallow and anterior half largely smooth; prepectal carina narrow lamelliform medio-ventrally, nearly reaching anterior border of mesopleuron and latero-ventrally curved; precoxal area of mesopleuron granulate and shiny; mesopleuron above precoxal area (except large smooth and shiny speculum) superficially granulate, but dorsally rugose; medially metapleuron nearly smooth, superficially granulate, shiny; mesosternal sulcus narrow and rather deep, smooth, without carina posteriorly; mesosternum rather angulate posteriorly; scutellum finely coriaceous and non-carinate laterally; lunula (= smooth lateral part of scutellum) wide medially; medio-posterior depression of metanotum rather narrow (Fig. 319); dorsal face of propodeum medium-sized, convex and coriaceous, but posteriorly with some carinae and smooth in between, without tubercles, median carina complete.

*Wings.* Fore wing: r 0.55 × 3-SR (Fig. 317); 1-CU1 horizontal and slender, 0.55 × as long as 2-CU1; r-m 0.8 × 2-SR, and 0.5 × 3-SR; second submarginal cell elongate (Fig. 317); 1-SR slightly angled to 1-M and slender; cu-a inclivous, subparallel with CU1b, straight; 1-M slightly curved. Hind wing: apical half of marginal cell parallel-sided or nearly so (Fig. 317); 2-SC+R longitudinal; m-cu present as weakly pigmented fold; M+CU:1-M = 10:7; 1r-m 0.7 × 1-M.

*Legs.* Tarsal claws yellowish setose; hind coxa superficially finely coriaceous, rather shiny; hind trochantellus 2.2 × longer ventrally than wide; length of fore and hind femora 5.2 and 3.9 × their width, respectively (Figs 321–322); inner apex of hind tibia without distinct comb; length of inner hind spur 0.4 × hind basitarsus.

*Metasoma.* First tergite as long as wide posteriorly, flattened and latero-anteriorly lamelliform near dorsope; first–second tergites and base of third tergite mainly coriaceous with superficial rugulosity, and with median carina weakly developed on apical half of first tergite and middle of second tergite; second tergite 0.9 × longer than wide basally and 1.3 × as long as third tergite (Fig. 320); medio-basal area of second tergite minute; second suture rather deep and distinctly crenulate; remainder of metasoma largely smooth and shiny; fourth tergite without sharp lateral crease; ovipositor sheath largely densely setose and apically truncate.

*Colour.* Black; antenna, palpi, basal two-thirds of third tergite, apices of femora, bases of tibiae and hind coxa dark brown; pronotum postero-dorsally, trochanters, trochantelli and tegulae pale yellowish; fore and middle coxae, remainder of tibiae and tarsi brown; orbita (except ventrally and sides of face), femora (except apically), first tergite medio-apically, second tergite narrowly medially, apical third of third and following tergites mainly orange brown (Fig. 320); pterostigma and veins dark brown; wing membrane slightly infuscate.

##### Etymology.

This species from Sweden is named after the collector of the type specimen, Swedish lepidopterist Nils Ryrholm, whose generous donations of Swedish parasitic wasps to the NMS collection have enriched it greatly.

##### Distribution.

*Sweden.

#### “Aleiodes seriatus (Herrich-Schäffer, 1838)” sensu lato

Taxon classificationAnimaliaHymenopteraBraconidae

Figs 328 , 329–340

Rogas seriatus Herrich-Schäffer, 1838: 156–12, Fig. [type series lost].
Aleiodes seriatus ; Papp 1991: 107; Belokobylskij et al. 2003: 399.
Aleiodes vittiger Wesmael, 1838: 112; Shenefelt 1975: 1185; Papp 1991: 107; Belokobylskij et al. 2003: 399 (as synonym of Aleiodes seriatus) [examined].
Rogas kuslitzkyi Tobias, 1976: 88, 223–224; 1986: 83 (transl.: 137).
Aleiodes kuslitzkyi ; Belokobylskij et al. 2003: 399 (as synonym of Aleiodes seriatus).

##### Diagnosis.

**Figure 328.**
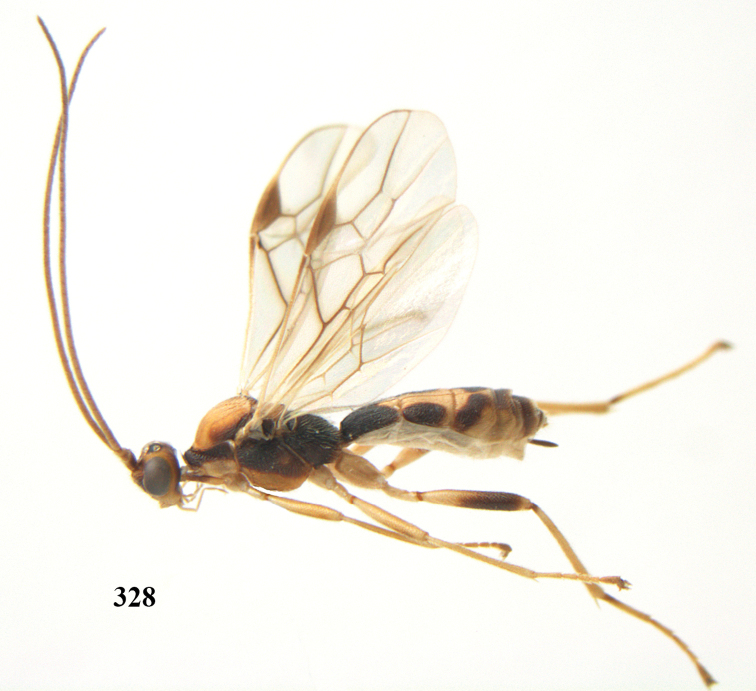
*Aleiodes seriatus* (Herrich-Schäffer) s.l., ♀, Netherlands, Wijster, habitus lateral.

**Figures 329–340.**
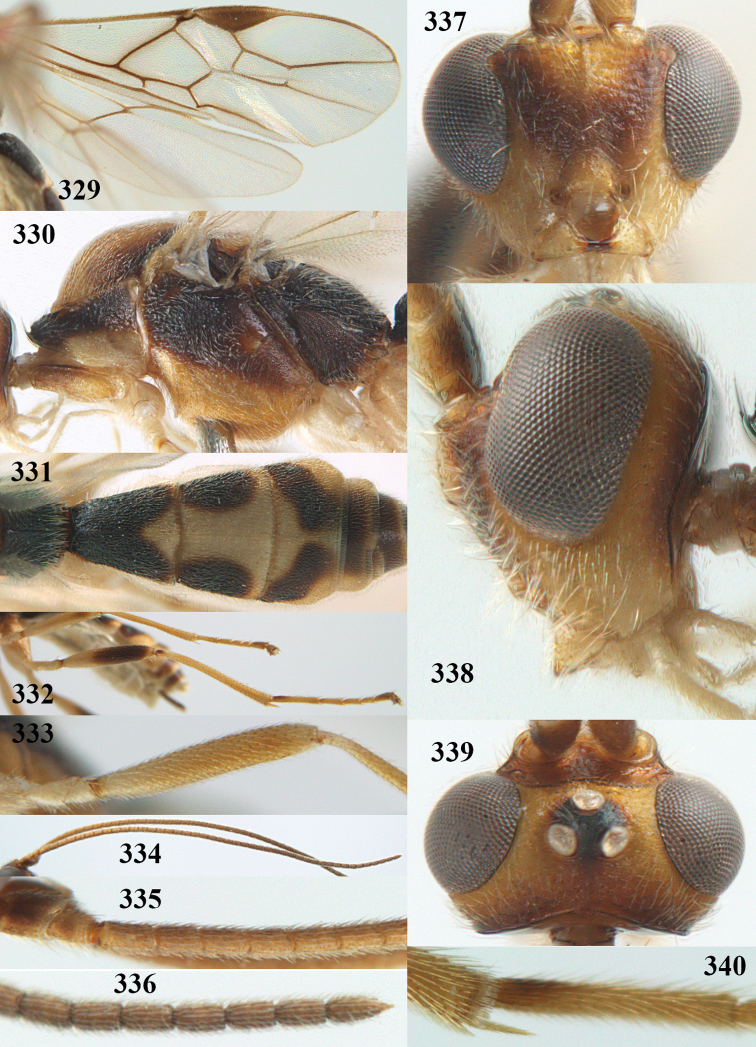
*Aleiodes seriatus* (Herrich-Schäffer) s.l., ♀, Netherlands, Wijster. **329** wings **330** mesosoma lateral **331** propodeum and metasoma dorsal **332** hind leg lateral **333** fore femur lateral **334** antenna **335** basal segments of antenna **336** apical segments of antenna **337** head anterior **338** head lateral **339** head dorsal **340** inner apex of hind tibia and basitarsus lateral.

Antennal segments of ♀ (35–)44–50, of ♂ (42–)48–53; length of malar space of ♀ 0.3–0.4 × (of ♂ 0.25 times) height of eye in lateral view (Fig. 338); OOL 0.8 × diameter of posterior ocellus; temple narrow (Fig. 339); surroundings of veins 1-M and 1-SR of fore wing more or less infuscate (Fig. 329); inner apex of hind tibia with comb (Fig. 340); metasoma of ♀ maculate (Fig. 331; less so in males); fourth tergite of ♀ pale (ivory) yellowish latero-posteriorly, in ♂ usually infuscate or this tergite uniformly pale brown; base of hind tibia usually narrowly dark brown; length of hind femur of ♀ 5.1–6.5 × its width (of ♂ up to 8 x); fourth tergite gently folded laterally, without acute lateral crease or this only anteriorly developed, although rarely present as a simple, non-lamelliform crease to apex of tergite; precoxal area, epicnemial area and propodeum laterally, rugose; fourth tergite superficially transversely rugulose or aciculate; setose part of ovipositor sheath 0.6 × as long as hind basitarsus. The patterning of the metasoma is characteristic but very variable in extent (Figs 328, 331).

##### Notes.

We include this taxon only in outline, because DNA evidence (Quicke & Shaw, unpublished) suggest that an aggregate of two species in Europe and another in the Russian Far East currently going under this name remains unresolved. This will be addressed in a future paper (van Achterberg, Shaw & Quicke, in prep.), but here we include *Aleiodes seriatus* sensu lato in the key because the aggregate is morphologically isolated (within the region covered), easily recognised and does not fall logically into any of the species groups represented. The aggregate is very widespread in Europe, and at least one segregate is associated with Lithosiini (Erebidae: Arctiinae).

#### Aleiodes testaceus

Taxon classificationAnimaliaHymenopteraBraconidae

(Telenga, 1941)

Figs 341–342 , 343–352

Heterogamus testaceus Telenga, 1941: 134; Shenefelt 1975: 1202; Tobias 1986: 85 (transl.: 142; lectotype designation); Papp 1991: 115 (examined).
Aleiodes testaceus Bergamesco et al. 1995: 5; Papp and Rezbanyai-Reser 1996: 83, 87, 1997: 116; Fortier and Shaw 1999: 221; van Achterberg and Aguiar 2009: 793–794.

##### Type material.

Lectotype ♀ (ZISP) from southern **Russia** (Astrakhanskaya Oblast) examined.

##### Additional material.

*Austria, **British Isles** (*England: V.C.s 2, 3, 5, 17, 21, 22, 23, 28, 29, 31, 32, 38, 39, 57, 58, 63; *Wales: V.C.s 48, 52; *Ireland: H5, H30 and Co. Cork), **Bulgaria**, *Cyprus, **France** (*mainland and *Corsica), **Greece** (mainland), **Italy**, **Morocco**, *Netherlands (NB: Tilburg (Kaaistoep)), **Portugal** (Madeira), **Spain** (mainland, *Mallorca and *Canary Islands (Gomera; Gran Canaria; Tenerife)). Specimens in NMS, BMNH, RMNH, CNC, BZL, OUM, ZISP, ZSSM, CMIM, UWIM, AAC.

##### Molecular data.

MRS057 (Corsica JF973344, CO1) MRS084 (England JF962864, CO1), MRS086 (England KU682223, CO1), MRS103 (Corsica KU682226, CO1), MRS106 (Corsica KU682227, CO1), MRS261 (France JF962863, CO1).

##### Biology.

This is a plurivoltine parasitoid of *Eupithecia* (Geometridae: Larentiinae) and close relatives feeding on flowers, overwintering as an adult. Specimens in NMS reared from wild collected larvae identified as *Eupithecia dodoneata* Guenée (10; T.H. Ford/France, M.R. Shaw/France), *Eupithecia* sp. (6), *Chloroclystis v-ata* (Haworth) (9; G.M. Haggett, M.R. Shaw), *Gymnoscelis rufifasciata* (Haworth) (2; G.M. Haggett, M.R. Shaw). Hosts were always associated with flowers, especially of trees (*Quercus*) and shrubby plants (*Ligustrum*, *Clematis*), but including field layer plants (*Solidago*, *Torilis*). Adults always emerge in the year of formation of the mummy, and females of this species overwinter as adults. In addition to a torpid specimen having been beaten from conifers in late December and another taken in early March (both M.W.R. de Vere Graham, southern England), this has been confirmed experimentally. However, in captivity the females tend to become active and require food above about 4 °C, indicating that the species is best adapted to areas where the winter is relatively short, as in southern Europe. Dissection of 5 (out of 51) female specimens collected by fogging *Quercus* canopy in mid September (southern England, N.E. Stork) revealed neither mature nor developing eggs, only moderately extensive fat bodies. As the species had been virtually absent from other fortnightly samples fogged from the same trees, it seems that they were feeding, perhaps on honeydew, prior to overwintering elsewhere. Because males are found late in the summer as well as early, it appears to be a plurivoltine species; however, it is unclear whether the autumnal specimens lacking mature eggs had resorbed them or refrained from developing them. Light trapped specimens tend to be female, but this is probably just a consequence of the much longer life span of the female sex, and in reared series the sex ratio is more equal. The light brown, banana-shaped, apically attenuated and short-lived mummy is interesting in not being stuck down at the thoracic area; instead (at least when forming from relatively elongate and fully exposed *Eupithecia* larvae such as those of *Eupithecia dodoneata*) it projects outwards at an angle similar to that normally adopted by the host at rest. None the less, a ventral opening is made by the parasitoid larva in the host’s thoracic region at the time of mummification, through which a small quantity of fluid is expelled and quickly dries.

##### Diagnosis.

Antennal segments of female 30–35 (of male 34–37); third antennal segment rather slender (Fig. 348); OOL about equal to diameter of posterior ocellus (Fig. 351); area in front of anterior ocellus with a minute smooth tubercle; ventral margin of clypeus thick (Fig. 350); maximum width of hypoclypeal depression 0.30–0.35 × minimum width of face; length of malar space of female 0.3–0.4 × height of eye in lateral view (Fig. 350); occipital carina very widely effaced dorsally (Fig. 351); vein r of fore wing 0.7–0.9 × vein 3-SR; vein 1-SR of fore wing rather long; vein 1r-m of hind wing distinctly shorter than vein 1-M (Fig. 343); marginal cell of hind wing somewhat widened apically; inner hind tibial spur 0.25–0.30 × as long as hind basitarsus; tarsal claws setose; pterostigma, inner side of basal half of hind tibia, head and mesosoma largely yellowish, but mesopleuron dorsally and propodeum usually more or less dark brown; third metasomal tergite with more or less developed diverging striae laterally

**Figures 341–342.**
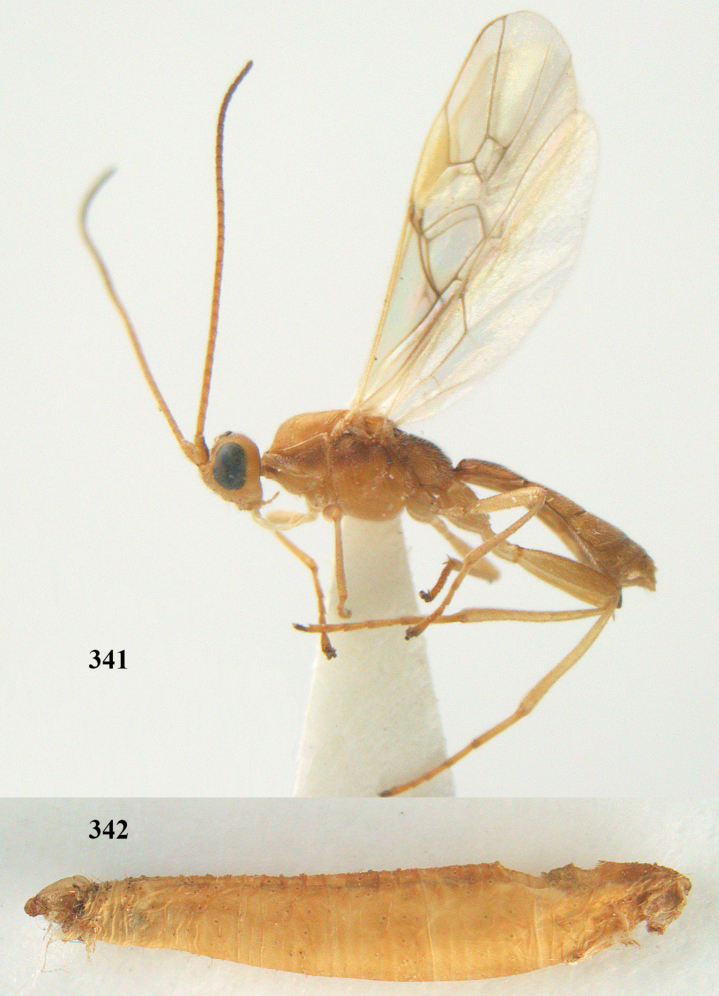
*Aleiodes testaceus* (Telenga), ♀, England, Bodney. **341** habitus lateral **342** mummy of *Gymnoscelis rufofasciata* (Haworth).

**Figures 343–352.**
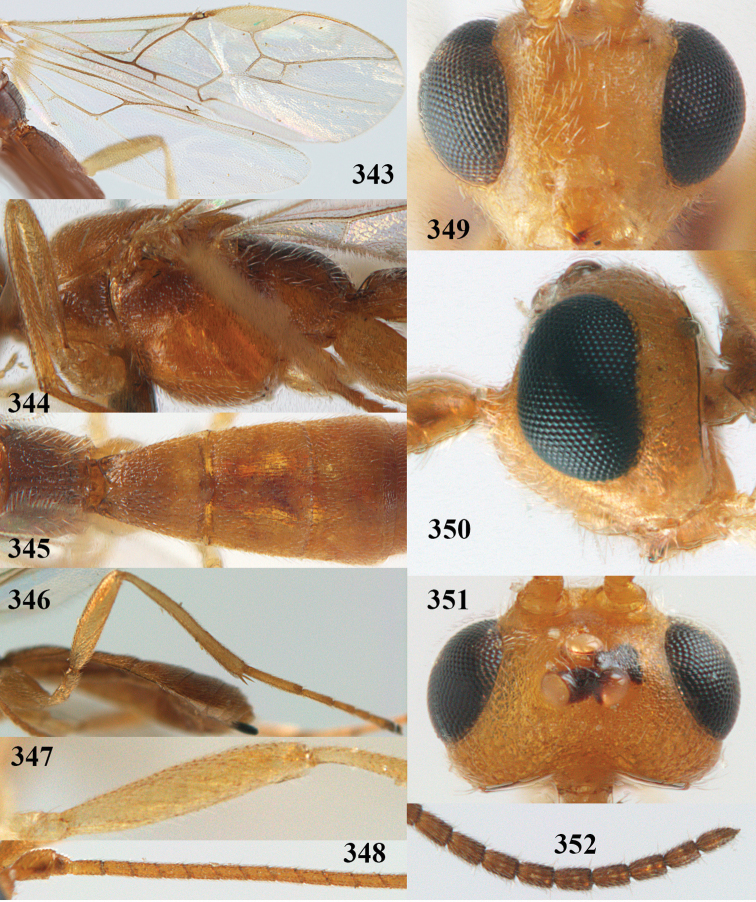
*Aleiodes testaceus* (Telenga), ♀, France, Var, Garde Freinet. **343** wings **344** mesosoma lateral **345** propodeum and metasoma dorsal **346** hind leg lateral **347** fore femur lateral **348** basal segments of antenna **349** head anterior **350** head lateral **351** head dorsal **352** apical segments of antenna.

##### Description.

Redescribed ♀ (RMNH) from Garde Freinet (France: Var), length of fore wing 3.3 mm, of body 3.7 mm.

*Head.* Antennal segments 32, antenna as long as fore wing, its subbasal and subapical segments about 2.4 and 1.7 × as long as wide, respectively; frons mainly granulate and flat; OOL equal to diameter of posterior ocellus and very finely granulate as vertex, with satin sheen; clypeus weakly convex, medium-sized and granulate; ventral margin of clypeus thick and depressed (Fig. 350); width of hypoclypeal depression 0.3 × minimum width of face (Fig. 349) and face granulate and with medium-sized setae; length of eye 3.2 × temple in dorsal view and temple gradually narrowed behind eye; occiput behind stemmaticum very finely granulate and occipital carina complete ventrally and widely interrupted dorsally; clypeus near lower level of eyes (Fig. 349); length of malar space 0.35 × height of eye in lateral view; eyes rather protruding (Figs 349–351).

*Mesosoma.* Mesoscutal lobes finely granulate, matt and medio-posteriorly rugose; anterior half of notauli narrow, shallow and smooth and posterior half obsolescent; prepectal carina narrow lamelliform medio-ventrally, not reaching anterior border of mesopleuron; precoxal area of mesopleuron finely rugulose medially (Fig. 344); remainder of mesopleuron above precoxal area nearly smooth or superficially shiny granulate, but dorsally finely rugose; medially metapleuron distinctly granulate and with some rugae, rather shiny; mesosternal sulcus narrow and shallow; mesosternum rounded posteriorly; scutellum nearly flat, densely granulate and laterally non-carinate; propodeum convex, and rugulose-granulate, median carina complete, without tubercles.

*Wings.* Fore wing: r nearly as long as 3-SR (Fig. 343); 1-CU1 horizontal, 0.5 × as long as 2-CU1; r-m 0.8 × 2-SR, and 0.7 × 3-SR; second submarginal cell short (Fig. 343); vein M+CU1 of fore wing apically at level of vein 2-CU1; vein 1-SR of fore wing rather long and nearly linear with vein 1-M (Fig. 343); cu-a short and vertical, first subdiscal cell narrow (Fig. 343); 1-M nearly straight posteriorly. Hind wing: apical half of marginal cell slightly widened apically; 2-SC+R short and longitudinal; m-cu present as unpigmented fold; M+CU:1-M = 7:5; 1r-m moderately oblique and 0.7 × as long as 1-M.

*Legs.* Tarsal claws yellowish setose; hind coxa granulate and with satin sheen; hind trochantellus 2.2 × longer ventrally than wide; length of fore and hind femora 6.4 and 4.7 × their width, respectively (Figs 346–347); inner apex of hind tibia without comb; length of inner hind spur 0.3 × hind basitarsus.

*Metasoma.* First tergite 1.1 × as long as wide posteriorly, convex and latero-posteriorly narrowly lamelliform; first–second tergites and basal 0.6 of third tergite finely longitudinally rugose, interspaces granulate and with median carina (Fig. 345); remainder of metasoma superficially micro-sculptured; medio-basal area of second tergite absent; second suture medium-sized, rather shallow and crenulate; third tergite 0.8 × as long as second tergite; fourth tergite without sharp lateral crease; remainder of metasoma partly retracted; ovipositor sheath truncate apically and moderately setose.

*Colour.* Brownish yellow; stemmaticum, patch on outer side of scapus and pedicellus, apical third of antenna, pronotum dorsally, mesopleuron dorsally, metanotum, metapleuron dorsally, propodeum largely and veins around 1-M of fore wing dark brown; remainder of veins brown or yellowish; palpi, tegulae and pterostigma pale yellowish; wing membrane subhyaline; first tergite somewhat infuscate basally; ovipositor sheath black.

*Variation.* Antennal segments of ♀ 30(1), 31(9), 32(24), 33(78), 34(30), 35(6); of ♂ 34(7), 35(16), 36(18), 37(9); side of pronotum and metasoma of both sexes sometimes largely dark brown or brown except ivory patch of second and third tergites and yellowish brown apex of metasoma.

##### Notes.

This species is distinctive and (with the wide use of UV light traps by lepidopterists) proving to be rather common in southern England, but it seems to have been very rarely collected and generally overlooked as a British species until quite recently. However, it has probably been present for a long time; a British specimen in the Dale collection (OUM) is dated 1892. In The Netherlands known since 2006 but only from one locality in the southern province of Noord-Brabant. On average the males have 2–3 antennal segments more than females.

The name “*testaceus*” (or the invalid emendation “*testaceator*” by Thunberg (1822)) has been used for 3 different taxa or groups of taxa in the genera *Rogas/Rhogas*, *Aleiodes* or *Heterogamus* (Braconidae: Rogadinae). Most taxa included in the past in *Rogas/Rhogas* are now included in the genus *Aleiodes* Wesmael, 1838 s. l. (Yu et al. 2012) as well as some taxa formerly included in *Heterogamus* Wesmael, 1838 (including *Heterogamus testaceus* Telenga, 1941).

The oldest name is *Ichneumon testaceus* Fabricius, 1798, not Gmelin, 1790. As a junior homonym *Ichneumon testaceus* Fabricius is unavailable, and the oldest available name for this taxon is *Rogas luteus* Nees, 1834 (see van Achterberg 1982). In 1822 Thunberg emended the Fabrician name to “*testaceator*”, but this emendation is invalid. The senior homonym *Ichneumon testaceus* Gmelin is a synonym of *Monoblastus brachyacanthus* (Gmelin, 1790) and belongs to the Ichneumonidae: Tryphoninae (Yu & Horstmann 1997).

Second is “*Aleiodes testaceus*” of Spinola (1808) of which the original combination is *Bracon testaceus*. It has so often been considered to be a valid taxon that it is included as such in Taxapad (Yu et al. 2012). However, in the supposed description by Spinola (1808), the author refers to *Ichneumon testaceus* Fabricius, 1798, and it is, therefore, not a valid description of a new taxon. Nomenclaturally it is the same as *Rogas luteus* Nees, 1834 (see above), but subsequent authors have incorrectly interpreted it as another (or other) species. *Rogas luteus* Nees is a valid name and refers to the type species of the genus *Rogas* Nees, 1819, and its (unjustified) emendation *Rhogas* Agassiz, 1849. “*Aleiodes testaceus* (Spinola)” of authors usually refers to one or another of several brownish yellow (“testaceous”) species of *Aleiodes*, most often the taxon which we consider to be correctly named as *Aleiodes similis* (Curtis, 1834), or taxa included in Taxapad under the species-aggregate of *Aleiodes gastritor* (Thunberg, 1822) s. l. (Yu et al. 2012). According to Art. 11.5 of the ICZN Code (1999), **t**o be available a name must be used as valid for a taxon when proposed, unless it was first published as a junior synonym and subsequently made available under the provisions of Art. 11.6.1. The status of a previously unavailable name is not changed by its mere citation as a valid taxon (Art. 11.5.2) and *Bracon testaceus* was not described by Spinola as a new taxon. A name could become available according to Art. 11.10, if it was deliberately employed for a misidentified type species, but this does not apply to the *Aleiodes testaceus* of Spinola. In principle, to maintain prevailing usage of a misinterpreted name, the case could be brought to the Commission for a ruling, but in our opinion there are no good reasons to do this because the intention of Spinola (1808) is unambiguous, the name has been used for several taxa, and it is not an iconic name that should be preserved because of many unambiguous published citations outside of the field of taxonomy.

Finally, *Heterogamus testaceus* Telenga, 1941, was (correctly in our view) included in the genus *Aleiodes* by several authors, including Shenefelt (1975) and Bergamesco et al. (1995). Thus *Aleiodes testaceus* (Telenga) could be confused, nomenclaturally, with “*Aleiodes testaceus* (Spinola)” of authors (belonging mainly to the *Aleiodes gastritor*-aggregate or other taxa of what we refer to in this paper as the *Aleiodes circumscriptus*-group). The two are only distantly related according to Fortier and Shaw (1999), a conclusion with which we concur (including from unpublished molecular data; Quicke et al. in prep.). Included among the purposes of this paper is to clarify the differences between these taxa and to suppress the incorrect usage of the name “*Aleiodes testaceus* (Spinola)” of authors not (Telenga 1941), as for example by Quicke (2015).

#### Aleiodes ungularis

Taxon classificationAnimaliaHymenopteraBraconidae

(Thomson, 1892)

Figs 353–354 , 355–365

Rogas (Aleiodes) ungularis Thomson, 1892: 1677 (examined).
Aleiodes ungularis ; Shenefelt 1975: 1184; Papp 1991: 98, 1985: 163 (lectotype designation).

##### Type material.

Lectotype ♀ (ZIL) from Sweden (“Pål”, “*ungularis*”, “Funnen vid Pålsjo I Skåne, Sverige, teste Papp, 1983”).

##### Additional material.

**British Isles** (*England: V.C. 18; *Wales: V.C. 49; *Ireland: V.C. H19) *Finland, **France**, **Germany**, **Greece**, **Hungary**, *Romania, **Slovakia**, **Sweden**, **Switzerland**. Specimens in NMS, BMNH, RMNH, FMNH, Doncaster Museum, MTMA, ZSSM, JLC.

##### Molecular data.

MRS604 (France JF962867, CO1 + KU682268, 28S).

##### Biology.

A probably monophagous and at least partly plurivoltine parasitoid of *Pseudopis prasinana* (Linnaeus) (Nolidae: Chloephorinae), overwintering as a mummy. The mummy is cylindrical and dark brown (Fig. 354). This is evidently a rare species because its common host is quite often collected in the larval stage, yet we have seen little material. Reared specimens seen from *Pseudoips prasinana* (5:2; M.R. Shaw/France, T. Weber/Germany). From four host larvae collected in late July (France: Haute-Marne, Fayl Billot) mummification took place over the next few days and all adults emerged in August. Only incomplete observations of its performance with its host in culture could be made, but there was no period of post-oviposition association in the otherwise normal oviposition sequences observed. Two mummies resulted in September from ovipositions obtained in August, the adults emerging the following May and June.

##### Diagnosis.

Maximum width of hypoclypeal depression 0.4 × minimum width of face (Fig. 361); OOL 0.6 × diameter of posterior ocellus; mesopleuron largely (and often strongly) shiny; mesosoma (except propodeum and metapleuron) largely yellowish (or orange); precoxal area impressed medially and crenulate (Fig. 356); inner side of hind tibia with whitish comb apically; metasoma dark brown or blackish medially and largely pale yellow laterally (Fig. 357). A distinctive species because of the combination of the shiny mesopleuron with the presence of the hind tibial comb.

**Figures 353–354.**
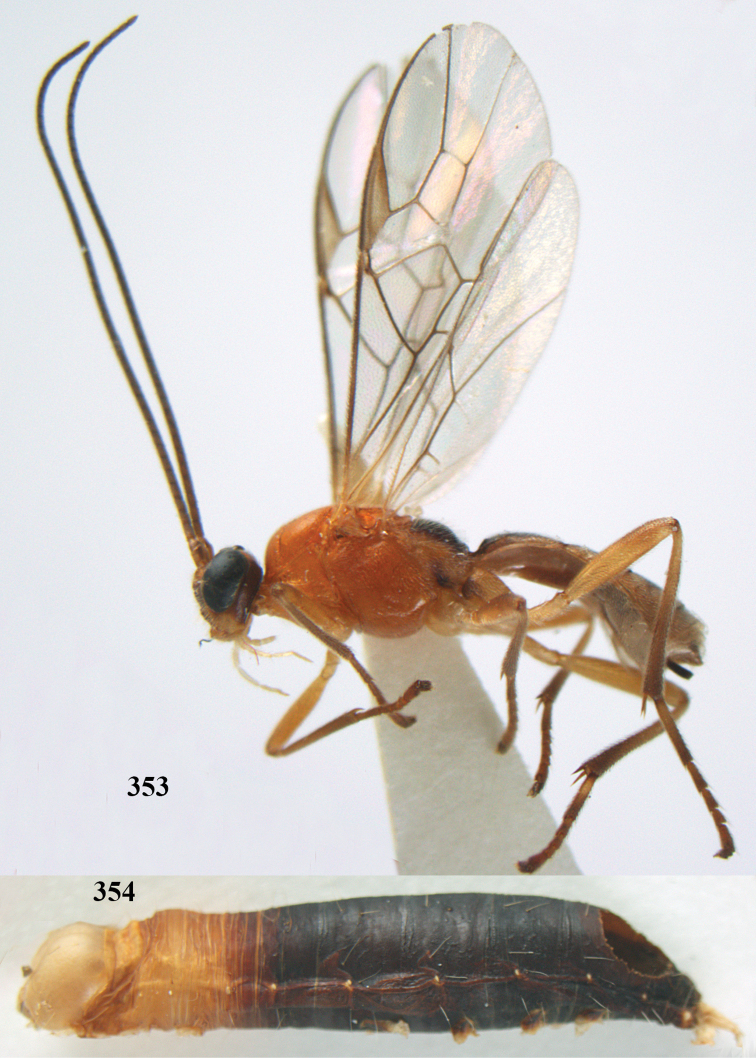
*Aleiodes ungularis* (Thomson), ♀, France, Fayl Billot. **353** habitus lateral **354** mummy of *Pseudopis prasinana* (Linnaeus).

**Figures 355–365.**
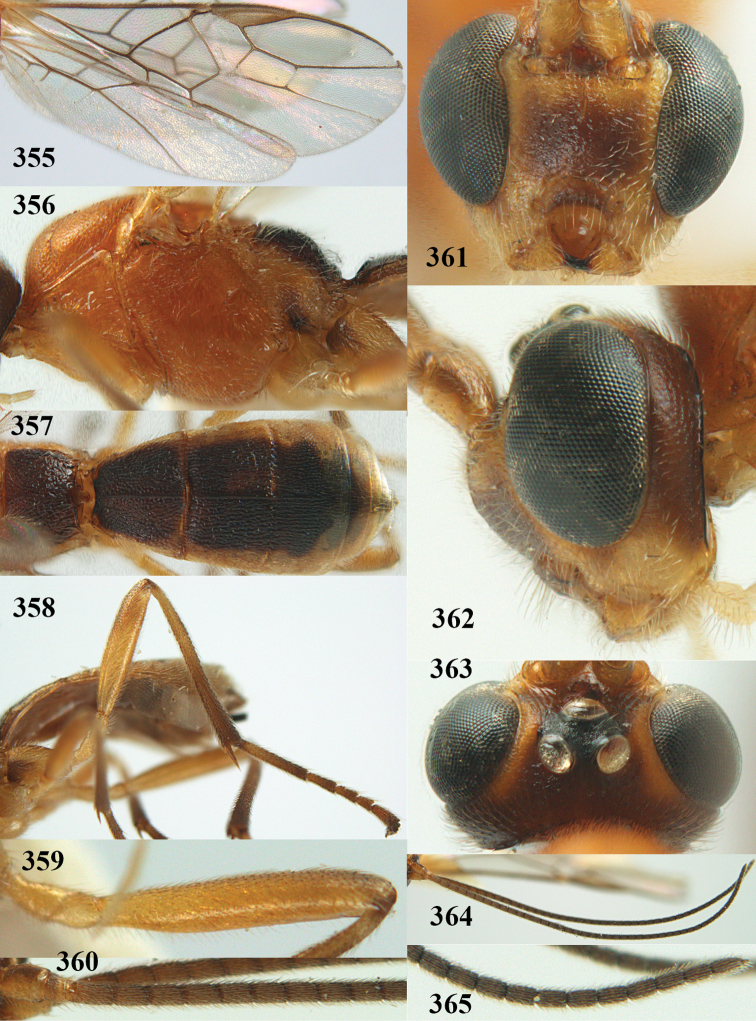
*Aleiodes ungularis* (Thomson), ♀, France, Fayl Billot. **355** wings **356** mesosoma lateral **357** propodeum and metasoma dorsal **358** hind leg lateral **359** fore femur lateral **360** basal segments of antenna **361** head anterior **362** head lateral **363** head dorsal **364** antenna **365** apical segments of antenna.

##### Description.

Redescribed ♀ (NMS) from Ardscull Moat (Ireland), length of fore wing 5.5 mm, of body 5.5 mm.

*Head*. Antennal segments of ♀ 46, with medium-sized setae, length of antenna 1.2 × fore wing, its subapical segments distinctly longer than wide (Fig. 365); frons weakly depressed and with coarse oblique rugae; OOL 0.6 × diameter of posterior ocellus and superficially granulate; vertex granulate, rather dull; clypeus convex, finely granulate and punctate; ventral margin of clypeus thick and not protruding forwards (Fig. 362); width of hypoclypeal depression 0.4 × minimum width of face (Fig. 361); length of eye 3.2 × temple in dorsal view and temple directly narrowed behind eye (Fig. 363); occiput behind stemmaticum superficially granulate, narrow; clypeus largely above lower level of eyes; length of malar space 0.25 × length of eye in lateral view (Fig. 362); occipital carina nearly complete medio-dorsally, complete and strong ventrally.

*Mesosoma*. Mesoscutal lobes finely and densely granulate and with fine punctation, matt; prepectal carina strongly developed, lamelliform, not reaching anterior border of mesopleuron; precoxal area of mesopleuron medially impressed and crenulate, remainder of mesopleuron (except dorsal rugose area) superficially granulate, shiny and with some superficial punctures (but in other specimens largely smooth); metapleuron granulate-punctate; scutellum finely granulate and rather flat, only antero-laterally with distinct carina; propodeum convex, rather short, distinctly rugose and its median carina complete, without tubercles.

*Wings*. Fore wing: r 0.5 × 3-SR (Fig. 355); 1-CU1 horizontal, 0.45 × 2-CU1; r-m 0.7 × 3-SR; second submarginal cell rather large (Fig. 355); cu-a slightly inclivous, straight; 1-M nearly straight posteriorly. Hind wing: marginal cell subparallel-sided, its apical width 1.6 × width at level of hamuli (Fig. 355); 2-SC+R shortly longitudinal; m-cu obsolescent; M+CU:1-M = 10:7; 1r-m long (Fig. 355) and nearly as long as 1-M.

*Legs*. Tarsal claws yellowish setose; telotarsi 1.5 × wider than fourth tarsal segment in dorsal view; hind coxa superficially granulate; hind trochantellus 1.8 × longer ventrally than wide; length of fore femur, hind femur and basitarsus 5.5, 5.1 and 6.8 × their width, respectively (Figs 358–359); length of inner hind spur 0.4 × hind basitarsus, as long as outer spur; inner apex of hind tibia with whitish comb.

*Metasoma*. First tergite robust; first and second tergites and basal half of third tergite finely and densely rugose, with distinct median carina; medio-basal area of second tergite minute; second suture narrow and rather shallow; third tergite nearly as long as second tergite; remainder of metasoma micro-sculptured, depressed; fourth and apical half of third tergite without sharp lateral crease; ovipositor sheath shiny, setose and apically truncate.

*Colour*. Yellowish brown; head (except orbita dorsally and part of malar space), metapleuron, propodeum and metasoma dorsally (except lateral margins) blackish; antenna, pterostigma, most veins, tibiae (except narrow pale basal ring), hind coxa largely, apical half of middle and hind femora largely, narrowly base of tibiae and telotarsi dark brown; palpi and tegulae yellowish brown; wing membrane subhyaline.

*Variation.* Antennal segments of ♀ 44(1), 45(1), 46(4), 47(2); of ♂ 41(1), 42(2), 43(5), 44(2); head mainly blackish or dark brown; metapleuron blackish or largely yellowish brown; hind coxa and tarsi nearly entirely dark brown or largely (except telotarsus) yellowish brown. In males the metasoma is sometimes entirely dark (without a paler lateral margin) and the hind coxae are dark brown.

##### Note.

Males have about three fewer antennal segments than females. In MTMA is a ♀ from Korea with 43 antennal segments and the apical half of hind coxa, hind and middle femora and tibiae dark brown, similar to males from Switzerland.

#### Aleiodes varius

Taxon classificationAnimaliaHymenopteraBraconidae

(Herrich-Schäffer, 1838)

Figs 366–367 , 368–378

Rogas varius Herrich-Schäffer, 1838: 156-7, fig.; Shenefelt 1975: 1244–1245 (as synonym of Aleiodes procerus).
Aleiodes varius ; Belokobylskij et al. 2003: 399.
Aleiodes procerus Wesmael, 1838: 104; Papp 1985a: 161 (lectotype designation), 1991: 93 (examined).
Rogas procerus ; Shenefelt 1975: 1244–1245; Tobias 1986: 81 (transl.: 135).

##### Type material.

Type series of *Rogas varius* is lost. Lectotype of *Aleiodes procerus* ♀ (KBIN), “**Belgique**, Charleroi/teste Papp J. 1983”, “*Aleiodes procerus*”, “dét. C. Wesmael”, “Lectotypus”, “*Aleiodes procerus* Wesm. 1838, ♀, Papp, 1983”. One ♀ paralectotype with same label data.

##### Additional material.

*Austria, *Finland, *Netherlands (Oisterwijk), **Russia**. There is a specimen in BMNH labelled “British Isles: Devignes Coll. B.M. 1868–52” but we have seen no other evidence of its occurrence in Britain, and it is probably extinct if indeed it ever occurred. Specimens in NMS, BMNH, FMNH, MSC, I. Kakko collection.

##### Molecular data.

MRS446 (Russia HQ551275, CO1).

##### Biology.

The only reared specimen seen is from *Euthrix potatoria* (Linnaeus) (Lasiocampidae) (E.O. Peltonen/Finland). The single date (6.vii.1987) on the label does not suggest voltinism or how the winter is passed, but the specimen is accompanied by a mummy (Fig. 367) whose elongate shape is very different from the relatively short stout mummies of the common *Aleiodes alternator* (Nees) (in the *Aleiodes bicolor*-group, not treated here) which parasitises the same host (among others).

**Figures 366–367.**
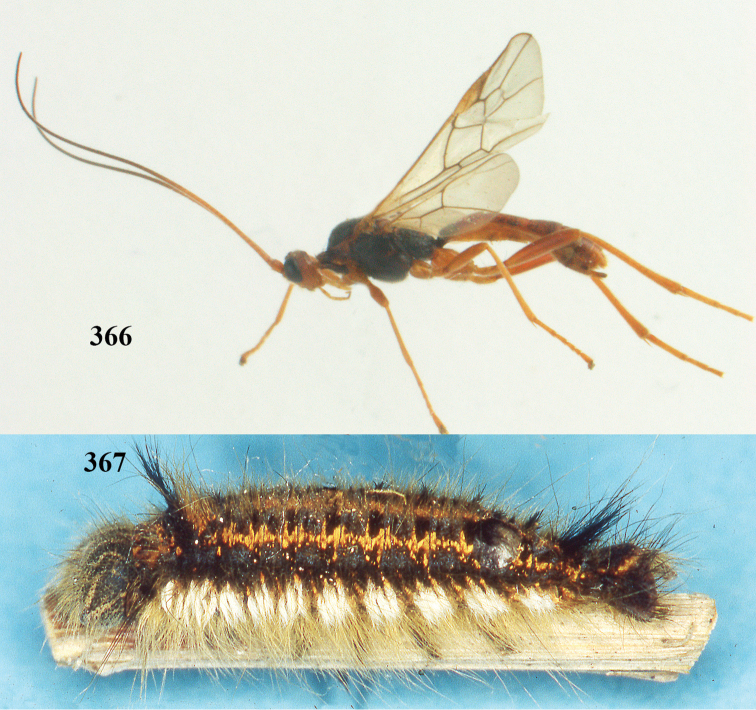
*Aleiodes varius* (Herrich-Schäffer), ♀, Finland, Lappeenranta. **366** habitus lateral **367** mummy of *Euthrix potatoria* (Linnaeus).

##### Diagnosis.

**Figures 368–378.**
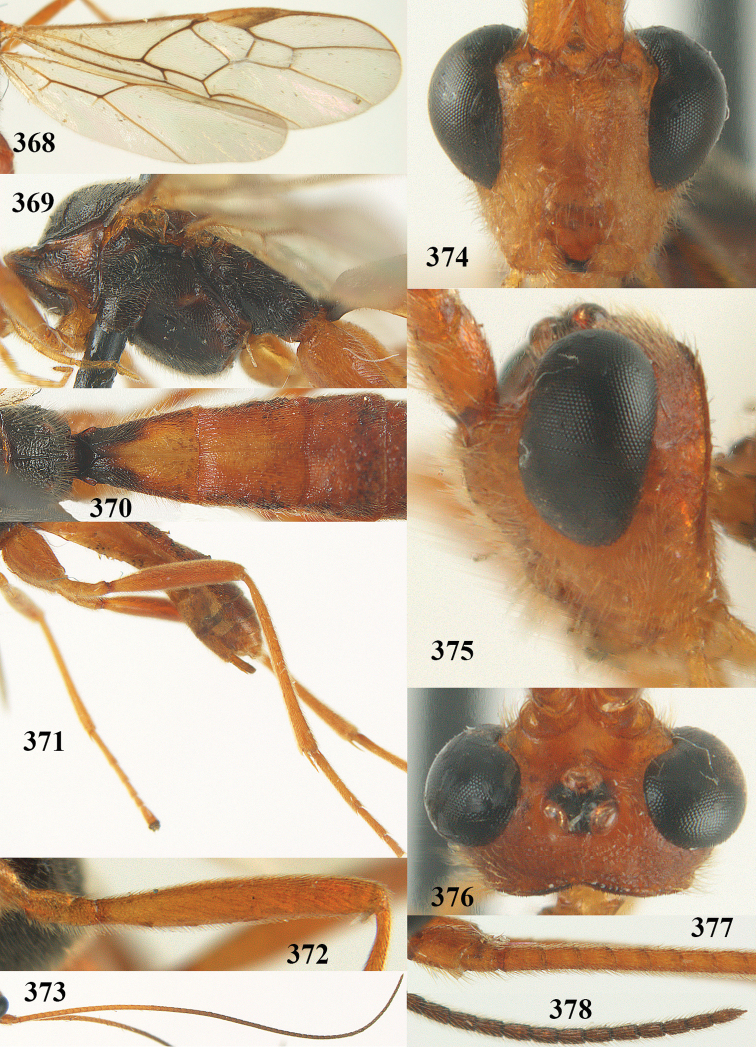
*Aleiodes varius* (Herrich-Schäffer), ♀, Finland, Lappeenranta. **368** wings **369** mesosoma lateral **370** propodeum and metasoma dorsal **371** hind leg lateral **372** fore femur lateral **373** antenna **374** head anterior **375** head lateral **376** head dorsal **377** basal segments of antenna **378** apical segments of antenna.

Antennal segments of ♀ 66–71; head (except stemmaticum) entirely brownish yellow; scapus in lateral view distinctly oblique apically; occipital carina reduced ventrally (Fig. 375); length of malar space 0.4 × height of eye in lateral view; vein 2-CU1 of fore wing 1.6–1.8 × vein 1-CU1 (Fig. 368); vein 1-SR slightly angled to vein 1-M and vein 1-M slightly curved (Fig. 368); vein r of fore wing gradually merging into vein 3-SR; fourth metasomal tergite superficially coriaceous; length of fore wing 8.0–8.5 mm.

##### Description.

Redescribed ♀ (NMS) from Lappeenranta (Finland), length of fore wing 8.2 mm, of body 10.1 mm.

*Head.* Antennal segments 69, length of antenna 1.4 × fore wing, its subapical segments about 1.9 × as long as wide and scapus in lateral view distinctly oblique apically (Figs 377–378); frons superficially granulate and with oblique rugae, rather shiny; OOL 0.7 × diameter of posterior ocellus and granulate; vertex granulate-rugulose, with satin sheen; clypeus rather high, convex dorsally but flattened ventrally, coriaceous and with long setae; ventral margin of clypeus thick and gradually depressed (Fig. 375); width of hypoclypeal depression 0.5 × minimum width of face (Fig. 374) and face mainly coriaceous dorsally; length of eye 2.4 × temple in dorsal view and temple directly narrowed behind eye (Fig. 376); occiput behind stemmaticum finely rugose and occipital carina arched and complete medio-dorsally and reduced ventrally (Figs 375–376); clypeus partly above lower level of eyes (Fig. 374); length of malar space 0.6 × height of eye in lateral view; eyes distinctly protruding (Figs 374–376).

*Mesosoma.* Mesoscutal lobes very finely coriaceous, with satin sheen, but medio-posteriorly with some rugae; notauli narrow, shallow and mainly coriaceous; prepectal carina rather lamelliform medio-ventrally, reaching anterior border of mesopleuron and latero-ventrally angulate; precoxal area of mesopleuron with some fine rugae medially (Fig. 369); mesopleuron above precoxal area (including hardly shiny speculum) superficially coriaceous, but dorsally coarsely rugose; medially metapleuron superficially granulate, rather shiny; mesosternal sulcus narrow and rather deep, without carina posteriorly; mesosternum angulate posteriorly; scutellum coriaceous-rugulose and carinate antero-laterally; dorsal face of propodeum rather long and densely moderately rugose, posterior face with some carinae and smooth in between, median carina complete, without tubercles, but somewhat angulate postero-laterally.

*Wings.* Fore wing: r 0.3 × 3-SR (Fig. 368); 1-CU1 horizontal, 0.55 × as long as 2-CU1; r-m 0.8 × 2-SR, and 0.5 × 3-SR; second submarginal cell stout (Fig. 368); 1-SR slightly angled to 1-M; cu-a oblique, not parallel with CU1b, straight; 1-M slightly curved posteriorly. Hind wing: marginal cell slightly narrowed submedially and slightly widened apically (Fig. 368); 2-SC+R short and longitudinal; m-cu present as slightly pigmented vein; M+CU:1-M = 17:15; 1r-m 0.6 × 1-M.

*Legs.* Tarsal claws with fine brownish pecten; hind coxa superficially finely coriaceous, with satin sheen; hind trochantellus 2.2 × longer ventrally than wide; length of fore and hind femora 7.5 and 5.3 × their width, respectively (Figs 371–372); inner apex of hind tibia without comb; length of inner hind spur 0.35 × hind basitarsus.

*Metasoma.* First tergite 1.3 × as long as wide posteriorly, flattened and latero-anteriorly distinctly lamelliform; first–second tergites and base of third tergite densely finely regularly rugose and with median carina; second tergite slender, 1.1 × longer than wide basally and 1.4 × as long as third tergite (Fig. 370); medio-basal area of second tergite absent; second suture rather deep and distinctly crenulate; apical half of third tergite mainly rugulose-coriaceous, remainder of metasoma superficially coriaceous and rather shiny; fourth tergite largely without sharp lateral crease; ovipositor sheath largely densely setose and apically truncate.

*Colour.* Yellowish brown; apical 0.6 of antenna dark brown; stemmaticum, mesosoma (except largely brownish pronotum, mesoscutum medio-posteriorly, scutellum, metanotum posteriorly) and base of first tergite black or nearly so; pterostigma pale yellowish but apical third infuscate (Fig. 368); medial veins dark brown and other veins brownish yellow; wing membrane subhyaline.

*Variation.* Length of fore wing 8.0–8.5 mm, of body 10.1–10.3 mm; antennal segments of ♀ 67(1), 68(1), 69(1), 70(1), 71(2), of ♂ 65(2), 66(1), 68(2), 69(1); marginal cell of hind wing parallel-sided or slightly narrowed submedially.

##### Note.

Females have on average 1–2 more antennal segments than males.

## Supplementary Material

XML Treatment for Aleiodes

XML Treatment for Aleiodes abraxanae

XML Treatment for Aleiodes albitibia

XML Treatment for Aleiodes angustipterus

XML Treatment for Aleiodes apiculatus

XML Treatment for Aleiodes arcticus

XML Treatment for Aleiodes artesiariae

XML Treatment for Aleiodes bistrigatus

XML Treatment for Aleiodes cantherius

XML Treatment for Aleiodes carminatus

XML Treatment for Aleiodes circumscriptus

XML Treatment for Aleiodes curticornis

XML Treatment for Aleiodes diarsianae

XML Treatment for Aleiodes esenbeckii

XML Treatment for Aleiodes jakowlewi

XML Treatment for Aleiodes leptofemur

XML Treatment for Aleiodes modestus

XML Treatment for Aleiodes nigriceps

XML Treatment for Aleiodes nigricornis

XML Treatment for Aleiodes pallidator

XML Treatment for Aleiodes pictus

XML Treatment for Aleiodes praetor

XML Treatment for Aleiodes reticulatus

XML Treatment for Aleiodes ryrholmi

XML Treatment for “Aleiodes seriatus (Herrich-Schäffer, 1838)” sensu lato

XML Treatment for Aleiodes testaceus

XML Treatment for Aleiodes ungularis

XML Treatment for Aleiodes varius
